# Pectate Lyase Pollen Allergens: Sensitization Profiles and Cross-Reactivity Pattern

**DOI:** 10.1371/journal.pone.0120038

**Published:** 2015-05-15

**Authors:** Ulrike Pichler, Michael Hauser, Martin Wolf, Maria Livia Bernardi, Gabriele Gadermaier, Richard Weiss, Christof Ebner, Hidenori Yokoi, Toshiro Takai, Alain Didierlaurent, Chiara Rafaiani, Peter Briza, Adriano Mari, Heidrun Behrendt, Michael Wallner, Fátima Ferreira

**Affiliations:** 1 Christian Doppler Laboratory for Allergy Diagnosis and Therapy, Department of Molecular Biology, University of Salzburg, Salzburg, Austria; 2 Associated Centers for Molecular Allergology, Rome, Italy; 3 Centro di Allergologia Molecolare, IDI-IRCCS, Rome, Italy; 4 Department of Molecular Biology, University of Salzburg, Salzburg, Austria; 5 Allergieambulatorium am Reumanplatz, Vienna, Austria; 6 Department of Otolaryngology, Kyorin University, School of Medicine, Tokyo, Japan; 7 Atopy (Allergy) Research Center, Juntendo University, Graduate School of Medicine, Tokyo, Japan; 8 Stallergenes S.A., Antony, France; 9 ZAUM, Center for Allergy and Environment, Munich, Germany; Mie University Graduate School of Medicine, JAPAN

## Abstract

**Background:**

Pollen released by allergenic members of the botanically unrelated families of Asteraceae and Cupressaceae represent potent elicitors of respiratory allergies in regions where these plants are present. As main allergen sources the Asteraceae species ragweed and mugwort, as well as the Cupressaceae species, cypress, mountain cedar, and Japanese cedar have been identified. The major allergens of all species belong to the pectate lyase enzyme family. Thus, we thought to investigate cross-reactivity pattern as well as sensitization capacities of pectate lyase pollen allergens in cohorts from distinct geographic regions.

**Methods:**

The clinically relevant pectate lyase pollen allergens Amb a 1, Art v 6, Cup a 1, Jun a 1, and Cry j 1 were purified from aqueous pollen extracts, and patients´ sensitization pattern of cohorts from Austria, Canada, Italy, and Japan were determined by IgE ELISA and cross-inhibition experiments. Moreover, we performed microarray experiments and established a mouse model of sensitization.

**Results:**

In ELISA and ELISA inhibition experiments specific sensitization pattern were discovered for each geographic region, which reflected the natural allergen exposure of the patients. We found significant cross-reactivity within Asteraceae and Cupressaceae pectate lyase pollen allergens, which was however limited between the orders. Animal experiments showed that immunization with Asteraceae allergens mainly induced antibodies reactive within the order, the same was observed for the Cupressaceae allergens. Cross-reactivity between orders was minimal. Moreover, Amb a 1, Art v 6, and Cry j 1 showed in general higher immunogenicity.

**Conclusion:**

We could cluster pectate lyase allergens in four categories, Amb a 1, Art v 6, Cup a 1/Jun a 1, and Cry j 1, respectively, at which each category has the potential to sensitize predisposed individuals. The sensitization pattern of different cohorts correlated with pollen exposure, which should be considered for future allergy diagnosis and therapy.

## Introduction

Understanding the process of allergic sensitization to a *per se* innocuous environmental antigen is considered the holy grail of allergology. The complex interplay between the allergen source, the allergen itself, the circumstances of exposure, and host immune factors are some aspects to be considered. For inhalant allergens, the airway mucosa seems to be the logical site of allergen entry; however, alternative models also suggest a very early mode of allergic sensitization for airborne allergens via the penetration of disrupted or injured skin [1, 2]. After allergen entry, a cascade of immunologic events is initiated leading to TH2 polarization and the production of specific IgE but only a limited number of proteins are capable of triggering the allergic cascade.

**Fig 1:**
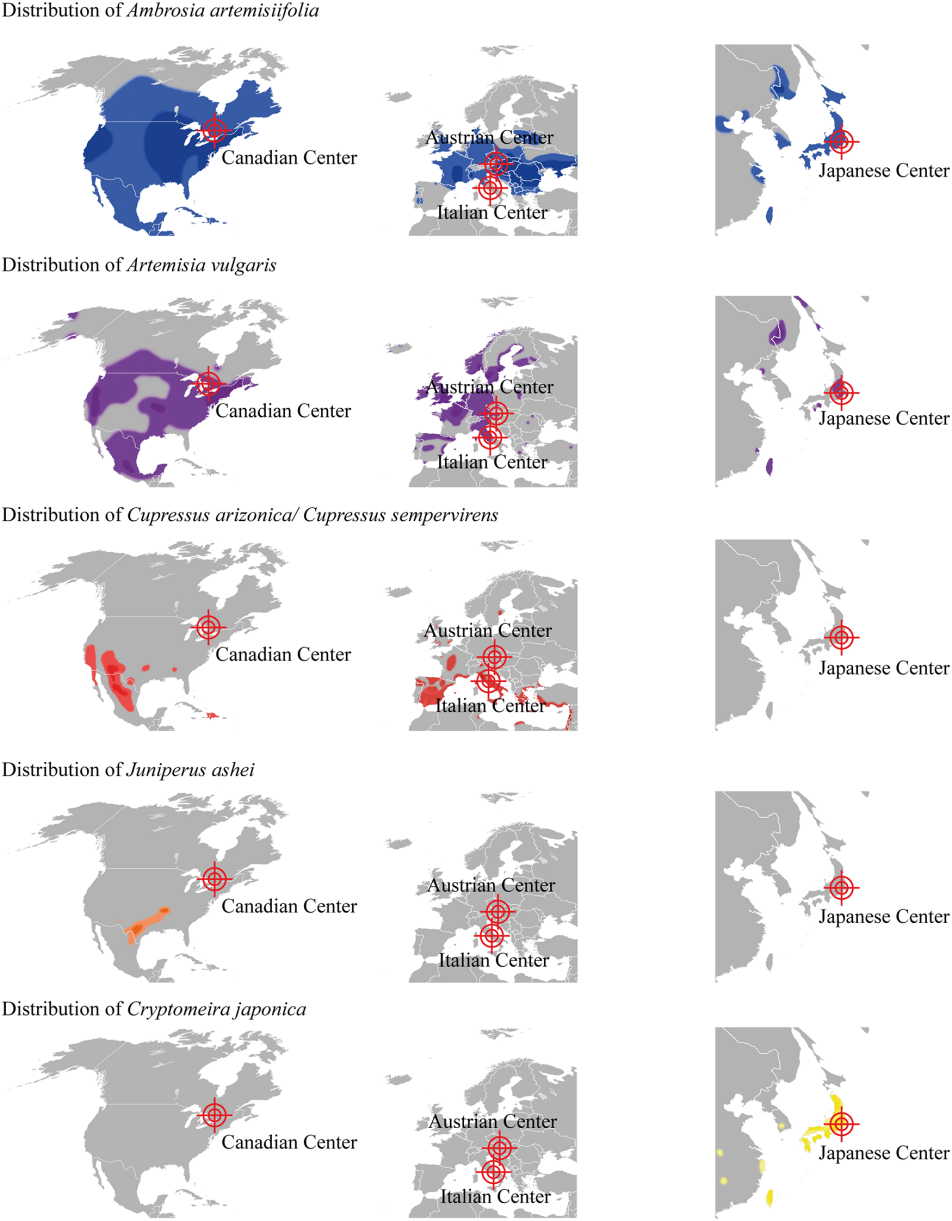
Geographical distribution of plants producing pollen containing clinically relevant allergenic pectate lyases. Light colored zones represent areas with low to medium distribution, dark colored zone areas with high distribution of the respective species. *Ambrosia artemisiifolia* (ragweed) is presented in blue, *Artemisia vulgaris* (mugwort) in violet, *Cupressus arizonica / Cupressus sempervirens* (cypress) in red, *Juniperus ashei* (Mountain cedar) in orange, and *Cryptomeria japonica* (Japanese cedar) in yellow. The 4 clinical centers of the study are depict as cross lines (www.gbif.org; www.pollenlibrary.com; eol.org) [3, 4].

Pollen of plants belonging to the botanically unrelated families of Asteraceae and Cupressaceae are an emerging cause of pollinosis predominately in the Northern hemisphere. The typical distribution of these allergenic plants indicates a geographic separation according to plant families but also according to different species (Fig 1) (www.gbif.org; www.pollenlibrary.com; eol.org) [3, 4]. In brief, the allergenic Cupressaceae species *Juniperus* as well as *Cupressus* populate the southern part of North America, whereas ragweed (*Ambrosia artemisiifolia*) belonging to the Asteraceae family is endemic in the northern regions of the continent. In Europe, cypress species populate the Mediterranean area, while the allergenic Asteraceae species mugwort (*Artemisia vulgaris*) as well as the neophyte ragweed form large populations in central European areas. Moreover, mugwort but not ragweed can be found in Mediterranean areas, whereas *Cupressus* species have also been introduced into the flora of more northern parts of Europe for ornamental purposes. In the eastern parts of Asia including Japan, ragweed as well as mugwort populate the temperate climate zones, nevertheless the Japanese island is considered a hot spot for the appearance of the Cupressaceae *Cryptomeria* and *Chamaecyparis*.

**Fig 2:**
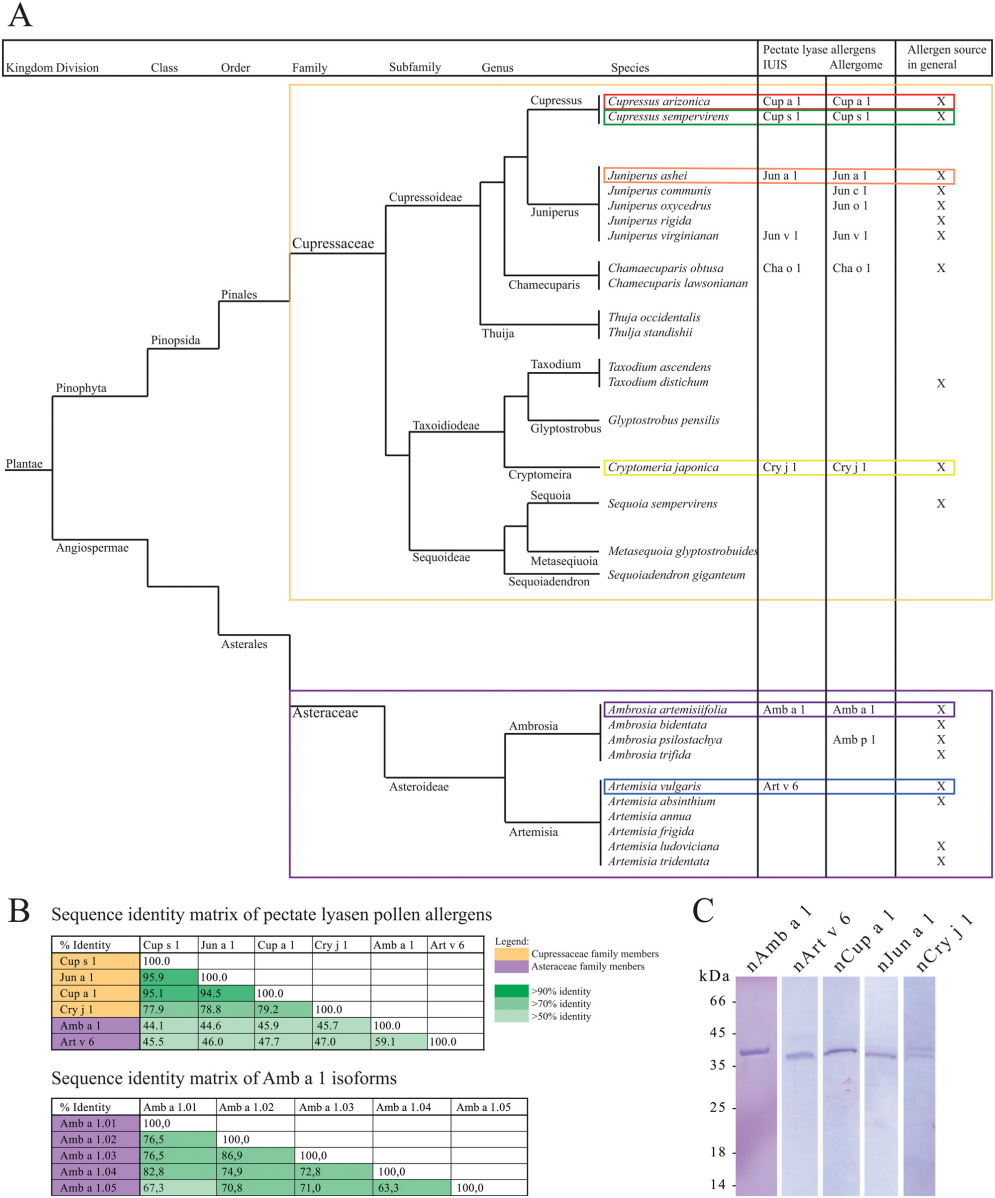
Taxonomy of Cupressaceae and Asteraceae and SDS-PAGE of purified allergenic proteins. **(A)** Phylogenetic tree of Cupressaceae and Asteraceae species. **(B)** Identity matrix of pectate lyase pollen allergens and, in case of Amb a 1, allergen isoforms. **(C)** SDS-PAGE of allergenic pectate lyases purified from pollen extracts by anion-exchange chromatography and/or size exclusion chromatography. The gel was stained with Coomassie Brilliant Blue R-250. Natural Cry j 1 migrates as visible double band.

The major Cupressaceae allergens Cup s 1 from the Mediterranean cypress (*Cupressus sempervirens*) [5], Cup a 1 from the Arizona cypress (*Cupressus arizonica*) [6], Cry j 1 from Japanese cedar (*Cryptomeria japonica*) [7], and Jun a 1 from Mountain cedar (*Juniperus ashei*) [8], are all members of the protein family of pectate lyases. Moreover, the Asteraceae allergens Amb a 1 from short ragweed (*Ambrosia artemisiifolia*) [9] and Art v 6 from mugwort (*Artemisia vulgaris*) [10] also belong to this allergen family. Whereas pectate lyases represent major allergens in almost all allergenic pollen where they have been identified, sensitization rates of only 26% have been reported for Art v 6 in mugwort allergic patients [11], which might be a consequence of the very low expression levels of Art v 6 in the pollen [12]. A protein sequence alignment is provided in Fig 2A, a sequence identity matrix in Fig 2B.

Pectate lyase allergens are classified as polysaccharide lyases, a class of enzymes which cleave galacturonic acid-containing polysaccharide chains. In general, polysaccharide lyases are divided into 23 families, at which allergenic pectate lyases are clustered in family 1 (PL1). PL1 enzymes can be found in all kingdoms; however, most lyases are of microbial origin (www.cazy.org). In higher plants pectate lyases are involved in fruit ripening [13–15], whereas in pollen they may play a role in tissue remodeling and in pollen tube outgrowth [16, 17]. So far allergenic pectate lyases have been identified and acknowledged in the pollen of 11 plant species and 2 fungi (www.allergome.org; 9 are listed in the IUIS allergen nomenclature database www.allergen.org). Nevertheless, to our knowledge the cross-reactivity of these allergens across plant orders as well as their allergenic potential has not been elucidated so far. Thus, the aim of the present study was to identify cross-reactivity pattern of allergenic pectate lyases from Astereaceae weeds and Cupressaceae trees, and to define the key players of pectate lyase-driven allergies. Therefore, patient cohorts from 4 geographically distinct areas (Austria, Canada, Italy, and Japan) were recruited and systematically screened using purified natural allergenic pectate lyases (Amb a 1, Art v 6, Cup a 1, Jun a 1, and Cry j 1). In a murine model we aimed to reveal the sensitizing capacity of the different proteins.

## Methods

### Patients and sera

Patients with Asteraceae and/or Cupressaceae pollen allergy were selected on basis of case history, positive *in vivo* skin prick test, and *in vitro* IgE detection using the CAP system (Phadia AB, Uppsala, Sweden). To determine allergen-specific IgE ImmunoCAPs w1 (Common ragweed *Ambrosia artemisiifolia* [A. elatior]), t17 (Japanese cedar *Cryptomeria japonica*), w6 (mugwort *Artemisia vulgaris*), or ImmunoCAP ISAC nCup a 1 were used (Thermo Fisher Scientific, Vienna, Austria) (see Table A in S1 File). The cohorts included in the ELISA and ELISA inhibition testing showed the following mean IgE values ± SD: Austrian patients to common ragweed w1 40.92 kU/L ± 25.27 kU/L and to mugwort w6 18.18 kU/L ± 17.28 kU/L; Canadian patients to common ragweed w1 38.70 kU/L ± 24.50 kU/L; Italian patients to ImmunoCAP ISAC nCup a 1 8.77 kU/L ± 5.97 kU/L; Japanese patients to Japanese cedar t17 22.23 kU/L ± 23.52 kU/L. The Italian cohort included in ImmunoCAP ISAC microarray study showed a mean IgE values ± SD to nAmb a 1 of 0.28 kUA/L ± 2.83 kUA/L, to nCry j 1 2.03 kUA/L ± 4.53 kUA/L, and to nCup a 1 6.58 kUA/L ± 10.28 kUA/L.

### Ethics statement

Experiments using anonymous blood samples from respective patients were approved by the local ethics committees of the respective institutions (Canadian samples: Research was approved by the ethics committee IRB—Institutional Review Board Services, Aurora, Ontario, Canada; Austrian samples: Research was approved by the ethics committee of the Medical University of Vienna, ethics committee number 497/2005, Vienna, Austria; Italian samples: Research was approved by the ethics committee at IDI-IRCCS (Istituto Dermopatico dell’Immacolata—Istituto di Ricovero e Cura a Carattere Scientifico), ethics committee number 106/CE/2005, Rome, Italy; Japanese samples: Research was approved by the ethics committee at Juntendo University, Tokyo, Japan). Informed written consent was obtained from all subjects included in the study.

### Pollen extracts

*Ambrosia artemisiifolia* (formerly *A*. *eliator*) (batch number 02051201), *Artemisia vulgaris* (batch number 010111102), *Cupressus arizonica* (batch number 054709201), *Juniperus ashei* (batch number 042009207), and *Cryptomeria japonica* pollen (batch number 064309701) were purchased from Allergon AB (Ängelholm, Sweden). Cupressaceae pollen extracts were prepared by incubation with 20 mM Tris HCl, pH 7.4–8 over night at 4°C. Extraction of Astereaceae pollen was performed with 100 mM Tris HCl pH 9.5 for 2 hours at room temperature. Extracts were then centrifuged at 4°C for 20 min at 15.000 x g. The centrifugation step was repeated with the collected supernatant. Resulting supernatant was concentrated by ultra-filtration using a 30 kDa MWCO filter (Vivacell, Sartorius, Göttingen, Germany).

### Purification of the allergenic pectate lyases

Pollen extracts were filtered with a 0.45 μm syringe filter (GE Healthcare, Little Chalfont, United Kingdom). *Ambrosia artemisiifolia* and *Artemisia vulgaris* extracts were thereafter directly applied on a 5 ml Q Sepharose column (GE Healthcare). After loading and washing the column was eluted using a pH followed by a sodium chloride gradient. Fractions containing purified protein were pooled and dialyzed against 20 mM sodium phosphate pH 7.4. Other pollen extracts (*Juniperus ashei*, *Cupressus arizonica*, and *Cryptomeria japonica*) were precipitated with ammonium sulfate at 80% saturation and thereafter re-suspended in extraction buffers (20 mM Tris HCl, pH 7.4–8), followed by size exclusion chromatography using a Superdex 75 10/300 GL (GE Healthcare). Purity of the proteins was analyzed by sodium dodecyl sulfate polyacrylamide gel electrophoresis (SDS-PAGE).

### Amino acid analysis

Amino acid analysis was performed using the Pico Tag method (Waters, Eschborn, Germany) as described [18].

### Mass spectrometry

Tryptic digests of purified natural allergens were performed with a ProteoExtract Trypsin Digestion kit (Calbiochem, Gibbstown, NJ, USA). Peptides were separated by reversed phase-capillary HPLC and analyzed with an ESI-QTOF mass spectrometer (Waters, Milford, Massachusetts) [19].

### ELISA experiments

NUNC Maxisorp plates (Thermo Fisher Scientific, Waltham, USA) were coated with allergen (200 ng/well in 50μl of PBS pH 7.4) overnight at 4°C. Plates were blocked with TBS pH 7.4, 0.05% (v/v) Tween, 0.5% (w/v) BSA and incubated with patients’ sera diluted either 1:8 or 1:3, respectively, followed by an incubation step overnight at 4°C. For inhibition experiments, patients’ sera (dilution 1:10 or 1:3) were pre-incubated with 20 μg/ml antigen overnight at 4°C, thereafter 50 μl aliquots were transferred to the antigen-coated ELISA plate and incubated overnight at 4°C. Bound IgE was detected using alkaline phosphatase-conjugated monoclonal anti-human IgE antibodies (BD Biosciences, Franklin Lakes, USA) incubated for 1h at 37°C and 1h at 4°C. After washing a colorimetric detection was performed using 10 mM 4-nitrophenyl phosphate (Sigma-Aldrich, St. Louis, USA) as substrate. Plates were measured at OD 405/492 nm. Specific serum IgE levels were determined based on a quantitative sandwich ELISA using goat anti-human IgE (KPL, Gaithersburg, MD, USA) as catching antibody and different concentrations of purified natural human IgE (Serotec, Düsseldorf, Germany). Values were calculated based on a regression curve obtained with the IgE standard and signals were considered positive when exceeding three times SD of background controls. For inhibition assays, sera were pre-incubated overnight with 20 μg/ml of purified allergen, and results are presented as percentage of inhibition comparing incubated with buffer-treated samples.

### ISAC testing

Immuno solid-phase allergen chip (ISAC) assays were performed as reported in previous studies [20]. ISAC 103 and ISAC 112 microarrays (Thermo Fisher Scientific) bearing 103 and 112 allergenic molecules, respectively, were used to screen sera. IgE results obtained on Amb a 1, Cup a 1, and Cry j 1 have been extracted and are herein reported. The three allergen preparations were all natural, highly purified compounds as available on both microarrays from the manufacturer (Thermo Fisher Scientific). Cut off positive value was set at 0.01 kU_A_/L. Results from ISAC testing were collected during routine diagnostic testing and results used under the ethical committee approvals at IDI-IRCCS, Rome, Italy, ethics committee numbers 106/CE/2005 and 63/CE/2007.

### Animal experiments

Female BALB/c mice (Charles River Laboratories, Sulzfeld, Germany) were purchased at 8 to 10 weeks of age and were used for experiments 7 days after arrival. Animals were immunized subcutaneously with 5 μg allergen adsorbed to Alu-Gel-S (Serva, Heidelberg, Germany) given as two 50 μl injections administered bilaterally in the lumbar regions. Mice were boosted on days 14, 28, and 42. Sera were collected on days 0 and 49. Per group, 5 animals were tested, at total 25 animals were included in the study. No adverse events have been observed. Animal experiments were conducted in accordance with EU guidelines 86/609/EWG and national legal regulations (TVG 2012). This study was approved by the Austrian Ministry of Science, Ref. II/3b (Gentechnik und Tierversuche), permission number BMWF-66.012/0011-II/10b/2010. Additional details on animal welfare are given in the Methods Section of the Online Supporting Information (S1 File). Antigen specific IgG1 and IgG2a were analyzed by ELISA. Therefore, NUNC Maxisorp plates (Thermo Fisher Scientific) were coated with 2 μg/ml antigen solution over night at 4°C. Murine sera were applied in serial dilutions followed by 2 h incubation at room temperature. After a washing step, bound IgG was detected with appropriate alkaline phosphatase-conjugated secondary antibodies (all from SouthernBiotech, Birmingham, USA) followed by chromogenic substrate development. Measurements were done in triplicates and titers were calculated by endpoint titrations. After isolation of splenocytes from immunized animals 2 x 10^5^ cells were added to each well and stimulation with 20 μg/ml protein antigen in 100 μl MEM (minimum essential medium Eagle) medium (Sigma-Aldrich, St. Louis, MO, USA) supplemented with 1% fetal bovine serum (FCS), 2 mM L-glutamine, 250 U/ml penicillin, 0.25 mg/ml streptomycin, 1 mM Na-pyruvate, 50 mM Hepes solution, 1x non-essential amino acids (all from PAN-Biotech GmbH, Aidenbach, Germany), and 2 μM β-mercaptoethanol (Sigma-Aldrich), at 37°C with 7% CO_2_. Antigen-specific IL-2 secreting splenic lymphocytes were analyzed by ELISpot assay using appropriate matched-pair antibodies (eBiosciences, San Diego, CA, USA) as described elsewhere [21]. Each measurement was performed in triplicates.

### Mediator release assays

IgE triggered mediator release assays were performed using the cell line RBL-2H3 (ATCC number CRL-2256) as described elsewhere [22]. Briefly, 4 x 10^4^ cells / well were incubated in RPMI 1640, 5% FCS, 4 mM L-glutamine, 2 mM Na-pyruvate, 10 mM Hepes, 100 U/ml penicillin, 0.1 mg/ml streptomycin (all from PAN-Biotech) and 100 μM β-mercaptoethanol (Sigma-Aldrich), overnight at 37°C with 7% CO_2_. Thereafter, cells were passively sensitized with murine immune sera at a dilution of 1:20, followed by 1 h incubation at 37°C, 7% CO_2_. After a washing step with Tyrode´s buffer (Sigma-Aldrich) supplemented with 0.1% w/v NaHCO_3_, 0.1% bovine serum albumin, pH 7.2 (all from AppliChem GmbH, Darmstadt, Germany), antigen was added to a final concentration of 300 ng/ml and mediator release was determined by enzymatic cleavage of the fluorogenic substrate 4-methyl-umbelliferyl-N-acetyl-b-glucosaminide (Sigma Aldrich). The measurement was performed at an excitation wavelength of 360 nm and an emission wavelength of 465 nm. Relative β-hexosaminidase activity is expressed as a percentage of the total enzyme activity of Triton X-100-treated cells. All experiments were performed in duplicates.

### Statistical Analysis

Statistical analysis was performed with either Graphpad Prism 6 or PASW Statistics 18 using appropriate test systems as indicated directly in the results section and in the figure legends. *P*-values < .05 were considered statistically significant.

## Results

### Purification and physico-chemical characterization of natural pectate lyase allergens

Five clinically relevant allergenic pectate lyases (ragweed Amb a 1, mugwort Art v 6, cypress Cup a 1, mountain cedar Jun a 1, and Japanese cedar Cry j 1) were purified from aqueous pollen extracts. Homogeneity of each preparation was verified by SDS-PAGE (Fig 2C), protein concentration was determined by amino acid analysis and protein identity was confirmed by mass spectrometry (MS). Protein preparations digested with trypsin were used for peptide mapping and peptide assignments were performed based on published allergen sequences. For Amb a 1, isoform specific peptides were found for all 5 isoforms listed in the IUIS allergen nomenclature database. The sequence coverage for Amb a 1.01 was 68.43%, for Amb a 1.02 31.66%, for Amb a 1.03 44.84%, for Amb a 1.04 18.88%, and for Amb a 1.05 64.99%. We found that natural Amb a 1 was composed of 47.0% Amb a 1.01, 7.4% Amb a 1.02, 11.5% Amb a 1.3, 22.6% Amb a 1.04, and 11.5% Amb a 1.05, respectively. The corresponding sequence identity matrix in is provided in Fig 2B. For Art v 6.01 the sequence coverage was 40.65%, for Cup a 1.01 48.27%, for Jun a 1.01 48.77%, and for Cry j 1.01 43.26%, respectively. Cry j 1 formed a double-band in gel electrophoresis, most likely as a result of heterogeneous glycosylation [23]. Both bands were identified as Cry j 1 by mass spectrometry after tryptic digest, as well as immunoblot analysis. The immunoblot experiment was performed with an in-house produced murine monoclonal anti-Cry j 1 antibody (data not shown).

### Patients from different geographic areas show distinct IgE reactivity pattern

**Fig 3:**
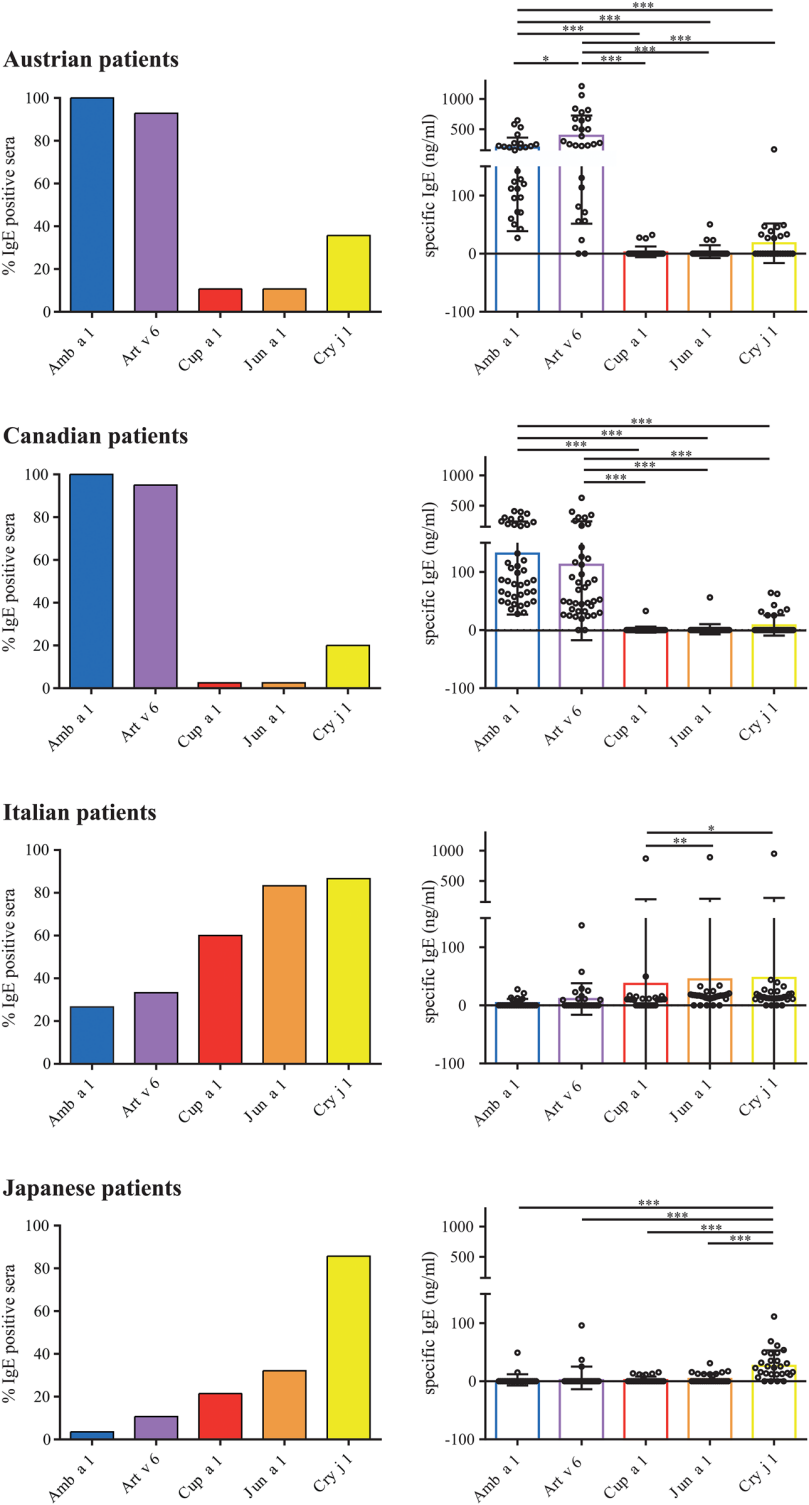
Patients´ serum IgE levels determined by ELISA. IgE binding of sera from Austrian (n = 28), Canadian (n = 40), Italian (n = 30), and Japanese (n = 28) Asteraceae and Cupressaceae pollen allergic patients were tested on natural Amb a 1, Art v 6, Cup a 1, Jun a 1, and Cry j 1. % values for IgE positive sera are given on the left, IgE levels are presented as mean ± SD on the right. Statistical analysis was performed with Repeated Measures ANOVA and Tukey´s Multiple Comparison Test (* *P*< .05, ** *P*< .01, *** *P*< .001).

The percentage of IgE positive sera and the mean IgE reactivity to different pectate lyase allergens were determined by ELISA using serum samples from four different geographic regions (Austria, Canada, Italy, and Japan, respectively) (Fig 3). In Austria 100% of the tested patients reacted to Amb a 1 with a mean IgE reactivity of 200 ng/ml. Of note, only 93% reacted with Art v 6; however the mean reactivity was 389.3 ng/ml. 10% of the Austrian patients had Cup a 1 reactive IgE (3.1 ng/ml), 10% also reacted with Jun a 1 (3.5 ng/ml), but 36% reacted with Cry j 1; however with low IgE reactivity (17.7 ng/ml). 100% of the Canadian sera reacted with Amb a 1 (131.5 ng/ml) and 95% with Art v 6 (111.1 ng/ml), whereas IgE binding to pectate lyases from Cupressaceae however was relatively low compared to Amb a 1 (2.5% reacted with Cup a 1 with a mean of 0.8 ng/ml, 2.5% with Jun a 1 with a mean of 1.4 ng/ml, and 20% with Cry j 1 with a mean of 7.9 ng/ml). With values lower than 50 ng/ml the pectate lyase specific IgE levels of Italian patients were generally low; nevertheless there was a clear trend towards increased binding of Cupressaceae pectate lyases compared to pectate lyase allergens from Asteraceae. 83.3% of the Italian patients reacted with Jun a 1, 60% with Cup a 1, 86.6% with Cry j 1, 26.6% with Amb a 1, and 33.3% with Art v 6. Similar as for the Italian cohort, in Japanese patients the IgE levels towards pectate lyases were generally low (between 2.3 ng/ml for Amb a 1 and 5.6 ng/ml for Art v 6) whereas the reactivity towards Cry j 1 was found to be significantly higher (28 ng/ml). We found 86% Cry j 1 positive sera, 32% were positive on Jun a 1, 21% on Cry j 1, 11% reacted with Art v 6, and 4% with Amb a 1.

### Patients´ sensitization profiles were analyzed by inhibition ELISA

**Fig 4:**
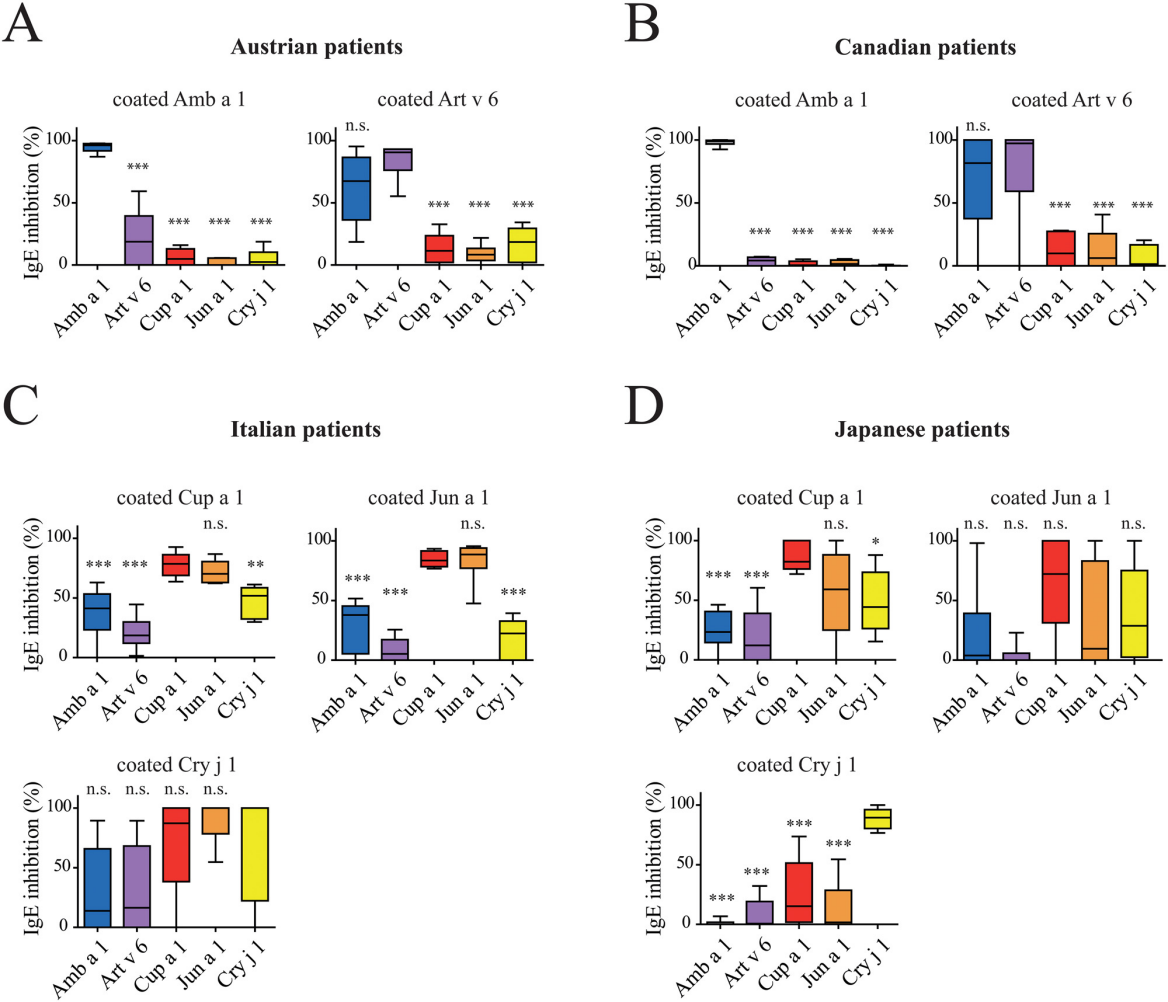
Inhibition ELISA of allergic patients´ sera. IgE binding of sera of pectate lyase pollen allergic patients from Austria (n = 6) and Canada (n = 6) were tested with coated Amb a 1 and Art v 6, respectively, sera from Italian (n = 6) and Japanese (n = 6) patients were tested with coated Cup a 1, Jun a 1, and Cry j 1. Prior to the ELISA all sera were pre-incubated with either 1 μg/ well of Amb a 1, Art v 6, Cup a 1, Jun a 1, or Cry j 1. Inhibitions are presented as box plots, median values are indicated as lines, whiskers indicate the 5–95 percentile. Results are presented as % inhibition compared to maximum IgE of untreated sera. Statistical analysis was performed with Repeated Measures ANOVA and Tukey´s Multiple Comparison Test (* *P*< .05, ** *P*< .01, *** *P*< .001).

For inhibition ELISA, sera were pre-incubated with each of the five pectate lyase allergens Amb a 1, Art v 6, Cup a 1, Jun a 1, and Cry j 1, respectively. Thereafter, IgE binding of the pre-incubated sera was tested using the allergens, which were found to be the strongest IgE binders in a distinct cohort as determined by direct ELISA experiments (Fig 4). In brief, Canadian and Austrian sera were tested with coated Amb a 1 and Art v 6, Italian and Japanese sera with the three Cupressaceae allergens Cup a 1, Jun a 1, and Cry j 1, respectively. All data are provided as median inhibition values.

For the Austrian patients we found that Amb a 1 inhibited IgE binding to coated Amb a 1 by 96.2%, whereas Art v 6 inhibited IgE binding by 18.8%, and the cypress allergens demonstrated very low levels of antibody inhibition (0–4.9%). However, when Art v 6 was coated, Amb a 1 could inhibit IgE binding only by 67.4%, Art v 6 by 90.6%, and the cypress allergens by 8.3–18.5%.

For the Canadian cohort we found that only Amb a 1 pre-incubation was able to fully inhibit IgE binding to Amb a 1 (98.8%) whereas all other pectate lyases inhibited IgE binding to Amb a 1 between 0–4.2%. When Art v 6 was coated and the inhibitions were performed with the 5 candidate allergens, we found that Amb a 1 (81.6%) and Art v 6 (97.3%) strongly inhibited IgE binding; inhibitions with Cupressaceae allergens ranged from 1.4–10%.

When analyzing the cohorts of Central Italy and Japan, all three members of the cypress pectate lyases were used for coating. For Italian patients we saw that the highly homologous proteins Cup a 1 and Jun a 1 could inhibit IgE binding to themselves by 78.6 and 88.5%, to each other by 70.2 and 83.5%. Cry j 1 inhibited IgE binding to Cup a 1 by 51.8%, Amb a 1 by 41.2%, and Art v 6 by 18.6%. IgE binding to coated Jun a 1 was inhibited by Cry j 1 with 22.4%, by Amb a 1 by 37.9%, and by Art v 6 by 5.2%. The IgE binding to coated Cry j 1 was fully inhibited with Cry j 1 as well as Jun a 1, Cup a 1 inhibition resulted was 87.2%, whereas Amb a 1 and Art v 6 inhibited binding to 13.9 and 16%, respectively. However, Italian sera showed very weak IgE binding to Cry j 1.

For Japanese patients the IgE signals towards coated Cup a 1 as well as Jun a 1 were generally very low. Cup a 1 could inhibit IgE binding to itself with 82.3%, Jun a 1 with 59.1%, Cry j 1 with 44.3%, Amb a 1 with 23.4%, and Art v 6 with 12.2%. IgE binding to Jun a 1 was inhibited by Cup a 1 with 72.2%, with Jun a 1 itself by 9.6%, a value that is very likely a result of the extremely low overall IgE binding to this allergen, with Cry j 1 by 28.8%, and between 0–3.9 with Amb a 1 and Art v 6, respectively. The more interesting part was the IgE reactivity towards Cry j 1. Here IgE signals were higher and the Japanese sera showed inhibition rates of 89.4% with Cry j 1 itself, 15.2% with Cup a 1, 1.6% with Jun a 1, 0% with Amb a 1, and 0.6% with Art v 6.

In summary, we conclude that Canadian patients are sensitized by Amb a 1, which shows cross-reactivity with Art v 6. Austrian patients are sensitized to both Amb a 1 and Art v 6. Patients from Central Italy are sensitized to cypress group 1 allergens, whereas sensitization of Japanese patients occurred mainly via Cry j 1. However, we also found some exclusive IgE to cypress allergens (mainly Cup a 1), which is most likely a result of sensitization to Cha o 1, the pectate lyase allergen from Japanese cypress (*Chamaecyparis obtusa*), which has been described as highly cross-reactivity with Jun a 1 and Cup a 1 [5].

### Microarray testing of 32,802 allergic Italian patients for pectate lyase allergies

**Fig 5:**
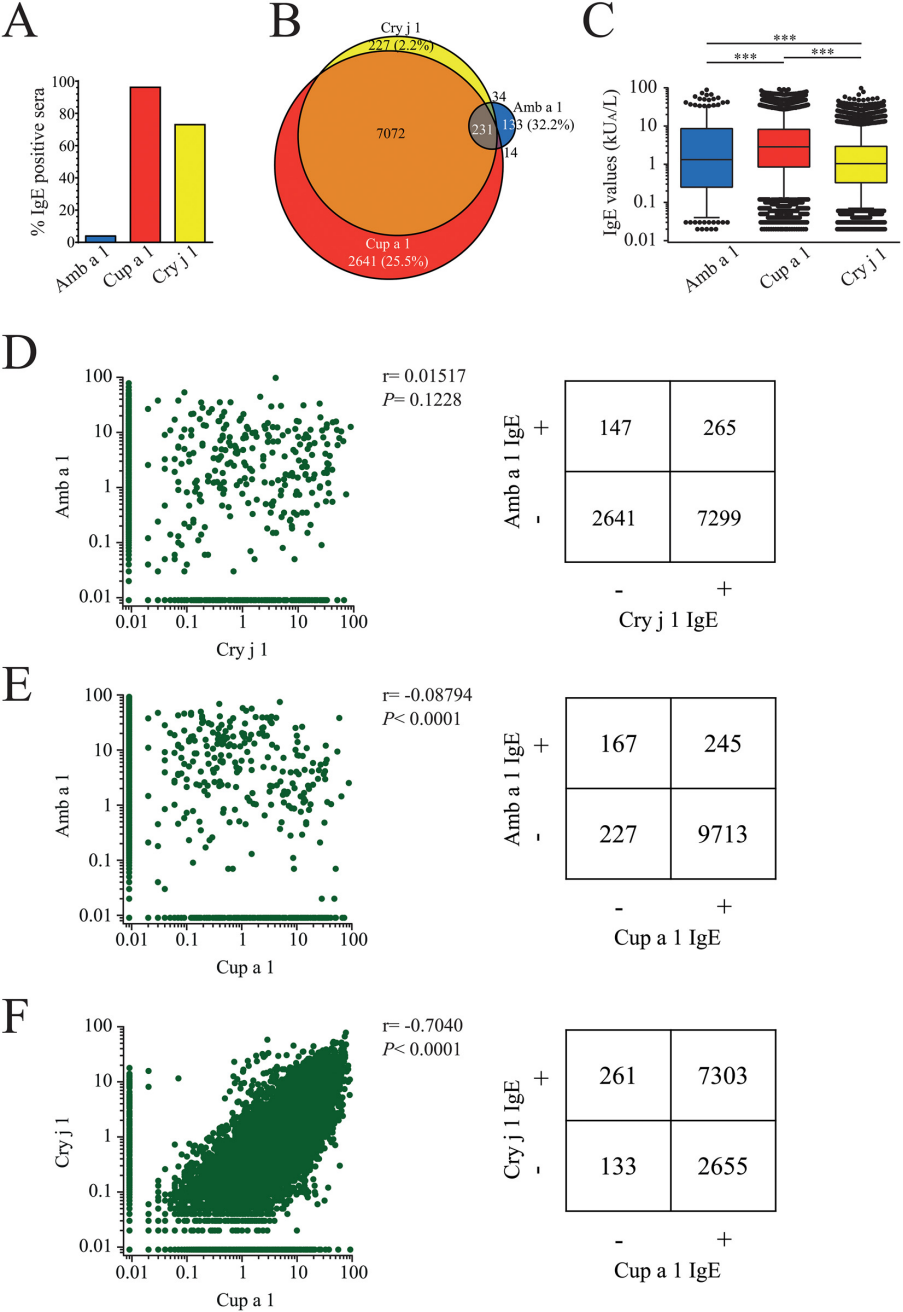
Sensitization patter of 10,352 allergic patients from Italy. **(A)** Immuno solid-phase allergen chip (ISAC) assays were performed to determine sensitization to pectate lyase allergens. **(B)** Venn diagram of 10,352 pectate lyase positive patients´ sera. **(C)** IgE values are presented as kU_A_/L, the cut off value was set at 0.01 kU_A_/L. Results are presented as box plots, medians are indicated as solid lines, 25–75 percentiles are boxed, whiskers are set at 5–95%, statistics were calculated with Repeated Measures ANOVA and Tukey´s Multiple Comparison Test (*** *P*< .001). **(D-F)** Correlations of pectate lyase sensitizations are presented as dot blot and in matrix format. Spearman´s rank correlation coefficients were calculated.

To investigate the IgE binding to pectate lyase allergens in a large cohort, microarray assays using the ISAC test system were performed with 32,802 (58.7% female) allergic patients, all from Italy. The age range was between 1 and 97 years old (see Table B in S1 File). 10,352 ISAC tests (31.56%) were IgE positive to at least one of the three pectate lyases. The vast majority of the Italian cohort reacted with Cup a 1 (96.18%), whereas 73.06% of the sera were IgE positive to Cry j 1 and 3.98 to Amb a 1 (Fig 5A). 25.5% of the sensitized individuals reacted exclusively with Cup a 1, whereas 70.5% reacted with Cup a 1 and Cry j 1, only 2.2% reacted only with Cry j 1. 1.3% of the tested patients reacted exclusively with Amb a 1 and 2.2% reacted with all three tested pectate lyase allergens (Fig 5B). The median IgE values of patients reactive to Amb a 1 were 1.32 kU_A_/L, whereas the median reactivity against Cup a 1 was 2.84 kU_A_/L and 1.04 kU_A_/L against Cry j 1 (Fig 5C). We found a significant correlation (*P*< .0001) of IgE binding only with Cup a 1 and Cry j 1, whereas there was no correlation of IgE reactivity between Amb a 1 and Cup a 1 or Amb a 1 and Cry j 1 (Fig 5D–5F). Thus, the majority of Italian patients is primarily sensitized to Cupressaceae pectate lyase allergens, which are cross-reactive with Cry j 1 from Japanese cedar. In this population sensitization to Amb a 1 is generally rare and does not correlate with sensitization to Cup a 1.

### Animal immunization with Asteraceae and Cupressaceae allergens induced no cross-reactivity between the two families

**Fig 6:**
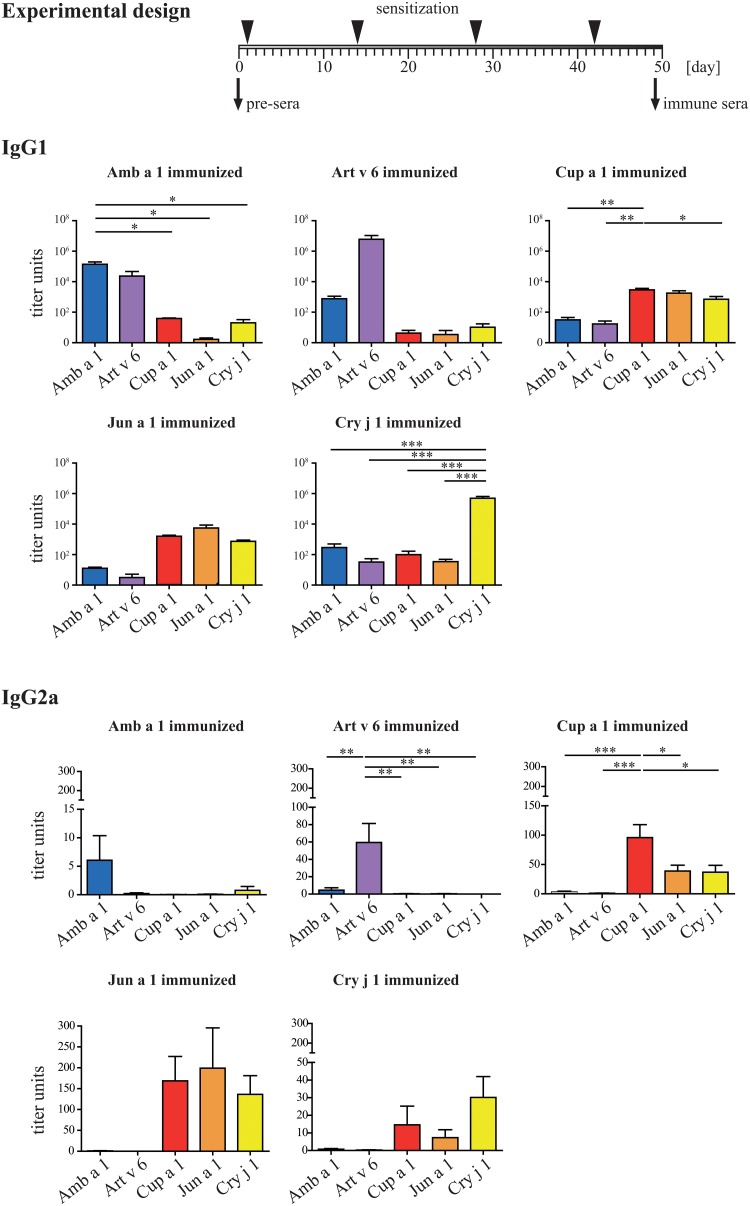
Humoral analysis of murine immune sera. The immunization scheme is presented as timeline, at each time point animals were immunized with 5 μg antigen adsorbed to Alu-Gel-S. Sera were drawn at day 0 and 49, respectively. Levels of allergen-specific IgG1 and IgG2a of mice (n = 5/ group) immunized with either Amb a 1, Art v 6, Cup a 1, Jun a 1, or Cry j 1 at day 49 were analyzed by ELISA. Titers are displayed as bars ± SEM. Statistical analysis was performed with One-way ANOVA and Tukey´s Multiple Comparison Test (* *P*< .05, ** *P*< .01, *** *P*< .001).

To further compare cross-reactivity pattern induced by Asteraceae and Cupressaceae allergens, mice were immunized four times by s.c. injection of Amb a 1, Art v 6, Cup a 1, Jun a 1, and Cry j 1, respectively, all adsorbed to Alu-Gel-S. Antibody responses and cross-reactivity of IgG (Fig 6) were monitored and are presented as mean titer units per group. Animals immunized with Amb a 1 showed strong IgG1 titers against Amb a 1 (1.4*10^5^) and Art v 6 (2.4*10^4^), however the recognition of Cup a 1, Jun a 1, and Cry j 1 was low (titers below 1*10^2^). Art v 6 immunized animals had very high titers against Art v 6 (6*10^6^), but the antibodies showed hardly any cross-reactivity (all titers below 1*10^3^). The murine IgG1 response triggered by Cup a 1 immunization was generally very low. The highest titers were induced against Cup a 1 itself (2.9*10^3^), Jun a 1 (1.8*10^3^), and Cry j 1(7.1*10^2^), whereas almost no reactivity was observed with Amb a 1 and Art v 6 (titers of 31 and 17). Jun a 1 immunization led to a similar picture, the titers against Jun a 1 itself were 5.5*10^3^, followed by 1.6*10^3^ against Cup a 1, and 7.5*10^2^ against Cry j 1. Amb a 1 and Art v 6 showed titers of 12 and 3, which resembles background values. Interestingly, the immunization with Cry j 1 led to high specific IgG levels (5*10^5^), whereas the recognition of all other pectate lyases including Cup a 1 and Jun a 1 was very low (between 32 and 289 titer units). Though, IgG2a levels were low, the IgG2a binding pattern was interesting. The cross-reactivity was low between Astereaceae and Cupressaceae allergens, and also the cross-reactivity between Amb a 1 and Art v 6 was low. However, we found some cross-reactivity within the Cupressaceae allergens. Of note, the allergens which elicited robust IgG1 titers had hardly detectable IgG2a (Amb a 1, Art v 6, and Cry j 1), whereas the allergens with low IgG1 titers had more IgG2a (Cup a 1 and Jun a 1).

**Fig 7:**
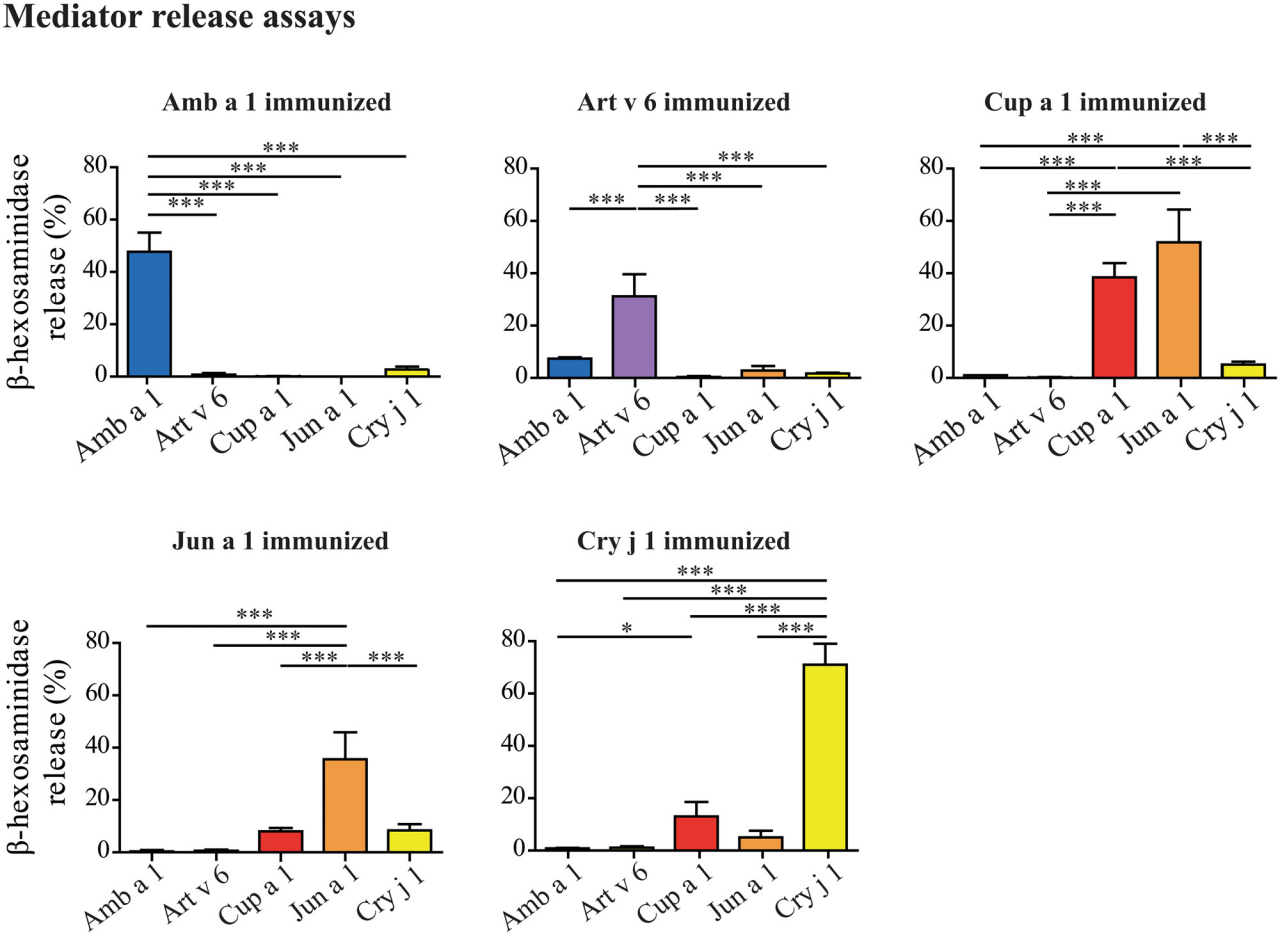
Biologically functional IgE of immunized mice was analyzed by mediator release assays. RBL-2H3 cells were passively sensitized with pooled immune sera (n = 5 animals/ group) withdrawn at day 49, and mediator release was triggered by addition of 0.3 μg/ml allergen. Relative β-hexosaminidase activity was calculated compared to Triton X-100 treated cells. Results are presented as mean of duplicates ± SEM. Statistical analysis was performed with One-way ANOVA and Tukey´s Multiple Comparison Test (* *P*< .05, ** *P*< .01, *** *P*< .001).

Biological active IgE was detected by degranulation of rat basophilic leukemia cells (RBL 2H3) (Fig 7). Sera of Amb a 1 immunized animals elicited a strong mediator release upon reactivation with Amb a 1, whereas Art v 6 immunization induced IgE against Art v 6 showing low reactivity with Amb a 1. IgE induced by Cup a 1 was highly cross-reactive with Jun a 1, and minor reactivity with Cry j 1 was observed. Interestingly, IgE induced by Jun a 1 did not show this extensive cross-reactivity with Cup a 1. Cry j 1 was also weakly recognized. Immunizations with Cry j 1 induced the highest mediator release. Still the antibodies reacted very weakly with the other Cupressaceae allergens and no cross-reactivity with Asteraceae pectate lyases was observed.

### Proliferative responses induced by immunization with pectate lyase allergens

**Fig 8:**
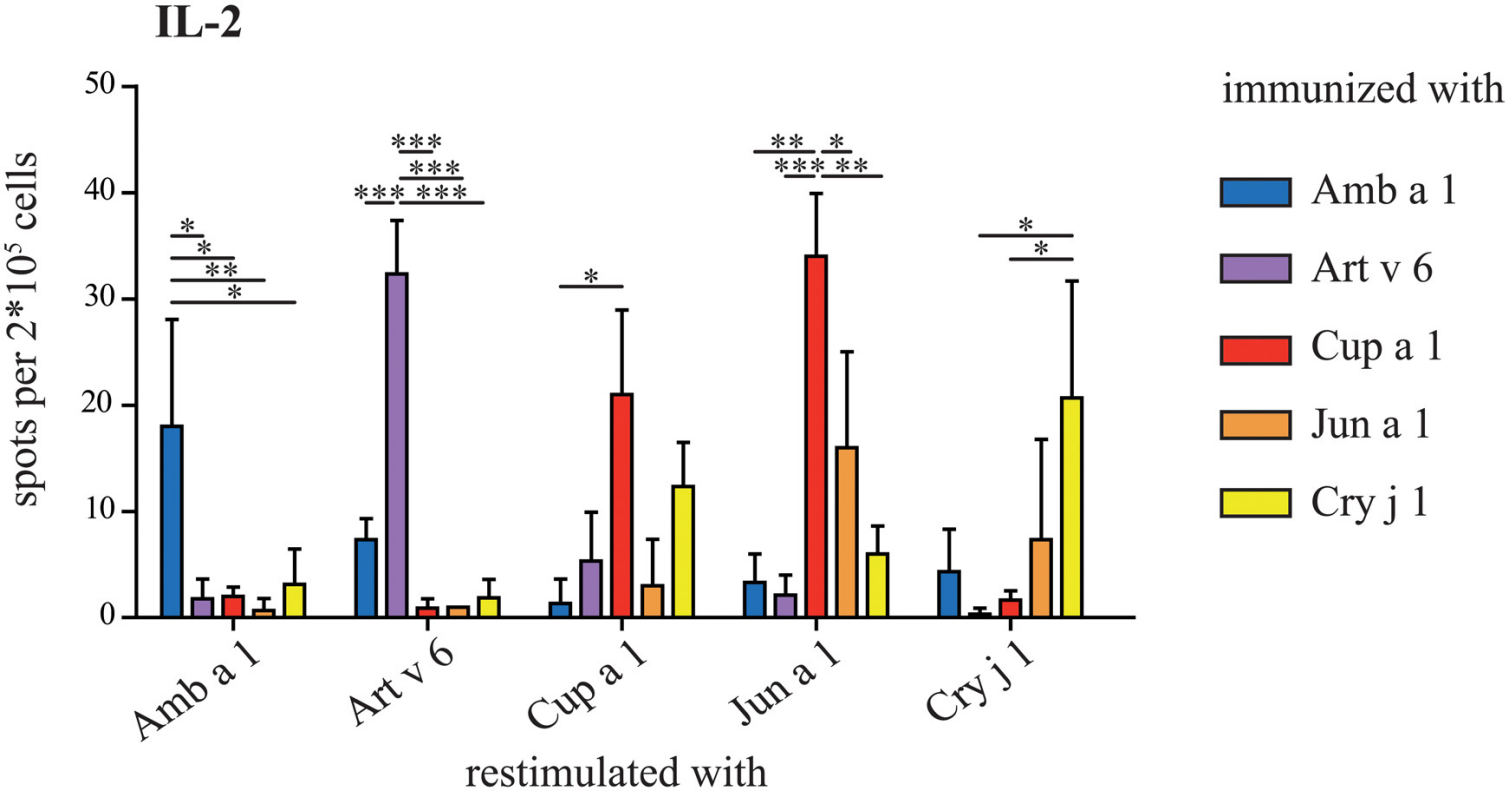
Proliferation of re-stimulated splenocytes. Activation of T cells was observed by the induction of IL-2 secreting cells. Therefore, splenocytes of immunized animals (n = 5 animals/ group) were re-stimulation with each of the five pectate lyase allergens. IL-2 secreting cells were detected by ELISpot. Results are presented as mean ± SD. Statistical analysis was performed with One-way ANOVA and Tukey´s Multiple Comparison Test (* *P*< .05, ** *P*< .01, *** *P*< .001).

Proliferation of splenocytes derived from immunized mice was induced by re-stimulation with pectate lyase allergens (Fig 8). We analyzed the number of IL-2 secreting cells in ELISpot assays and found that mainly the immunogen was able to induce significantly stronger proliferation. Re-stimulation of splenocytes from Amb a 1 immunized mice was only efficient with Amb a 1, whereas for Art v 6 immunized mice splenocytes could at least partially be reactivated with Amb a 1. For Cup a 1 immunized animals we surprisingly found that Jun a 1 failed to reactivate the cells, whereas Cry j 1 could partially induce IL-2 secretion. Splenocytes obtained from Jun a 1 immunized mice could be reactivated with Jun a 1. However, re-stimulation with Cup a 1 resulted in significantly more IL-2 producing T cells. The re-stimulation of splenocytes from Cry j 1 immunized animals was most efficient with Cry j 1.

## Discussion

Due to their broad distribution, Asteraceae as well as Cupressaceae species are among the most potent elicitors of pollen allergy in many regions including North America, Europe, and vast parts of Asia. Though not botanically related, the major allergens of these plant families have been identified as pectate lyases. In general, the most abundant proteins in allergenic pollen extracts represent the major allergens of that source [24]. For pectate lyases this is true for ragweed, cypress, mountain cedar, and Japanese cedar, whereas in mugwort the most abundant protein is Art v 1, which has been described as a member of the defensin-like allergen family [12]. Nevertheless, also mugwort pollen contains an allergenic pectate lyase (Art v 6), which is recognized by approximately 20–26% of mugwort allergic patients [10]. Though IgE reactivity to Art v 6 was found to be a consequence of Amb a 1 sensitization in previous studies, it has also been reported that Art v 6 can act as a primary sensitizer in areas with high mugwort pollen exposure [25, 26].

To identify cross-reactive pattern of pectate lyase pollen allergens, ragweed Amb a 1, mugwort Art v 6, cypress Cup a 1, mountain cedar Jun a 1, and Japanese cedar Cry j 1 were purified from aqueous pollen extracts. Sera from patients of four geographic regions (Austria, Canada, Italy, and Japan) showing different exposure to pectate lyase allergens were collected and used for cross-reactivity profiling. As inclusion criterion for the study typical pollen allergy-associated symptoms and positive specific IgE values to the respective weed or tree pollen extracts and/or purified allergens were defined.

In Austria, exposure to mugwort as well as ragweed has been reported, while in Canada, ragweed pollen has been described as the main source of pectate lyases allergens. Patients from the southern and central parts of the Italy are primarily exposed to cypress pollen, whereas in Japan, Japanese cedar pollen represents the main source of allergenic pectate lyases (www.gbif.org; www.pollenlibrary.com; eol.org) [3, 4], though there are environmental and epidemiological reports about pollen dispersal and low sensitization prevalence to ragweed and mugwort in Japan as well [27].

Direct ELISA experiments revealed that more Austrian patients reacted with Amb a 1; however mean IgE levels towards Art v 6 were higher than to Amb a 1. This was surprising since almost all Austrian sera had higher CAP values to ragweed than to mugwort, but may be explained by the low levels of Art v 6 in mugwort pollen extracts. Canadian sera reacted strongly with Amb a 1, followed by Art v 6, whereas cross-reactivity with cypress allergens was minimal. Italian sera showed almost equal specific IgE binding to all three Cupressaceae pectate lyases, whereas Japanese sera reacted significantly stronger with Cry j 1 than with any other pectate lyase allergen. These observations were confirmed by inhibition ELISA.

For each region the prevalent allergens were used for coating (i.e. for Austrian patients Amb a 1 and Art v 6, the same for Canadian patients, for Italian and Japanese patients Cup a 1, Jun a 1, and Cry j 1). Thereafter, sera were pre-incubated with each of the allergens and residual IgE binding was determined. For Austrian patients we found that Amb a 1 most efficiently inhibited IgE binding to itself, whereas when Art v 6 was coated, Art v 6 was the most efficient inhibitor. In that case Amb a 1 could inhibit IgE binding to approx. 70%. Cupressaceae pectate lyases showed almost no inhibitory capacities. Therefore, we conclude that within the Austrian population we find both, sensitization to Amb a 1 as well as Art v 6. A recent study demonstrated that Amb a 1 and Art v 6 are widely cross-reactive in an Italian cohort. Both Amb a 1 as well as Art v 6 have the capacity to sensitize patients, and that IgE reactivity to the major mugwort pollen allergen Art v 1 can be used to distinguish between ragweed and mugwort sensitized individuals [26]. We could observe that in Austria sensitization to both allergens is prevalent, which correlates with the natural exposure to both, ragweed as well as mugwort pollen, in that region.

For Canadian sera only Amb a 1 could effectively inhibit IgE binding to coated Amb a 1, whereas the inhibition to coated Art v 6 was almost equal with Art v 6 as well as Amb a 1. Almost no inhibition could be observed with the Cupressaceae pectate lyases. This indicates that Canadian patients are sensitized by Amb a 1 and cross-react to Art v 6.

For Central Italy it has been reported that 62.9% of atopic patients showed sensitization to cypress pollen [28]. We found that Cup a 1 and Jun a 1 could almost equally well inhibit IgE binding to coated Cupressaceae pectate lyases, indicating a primary sensitization with cypress pollen-derived allergens. The high sequence homology between Cup a 1 and Jun a 1 (91%) explains the high level of IgE cross-reactivity [5]. The cross-reactivity with Cry j 1 is however limited—the sequence identity between Cup a 1 and Cry j 1 is only 75%—and cross-reactivity with Asteraceae pectate lyases is insignificant. The Japanese population is mainly exposed to the pollen of Japanese cedar, however also Japanese cypress (*Chamaecyparis obtusa*) pollen contains an allergenic pectate lyase (Cha o 1) showing high cross-reactivity with Jun a 1 and Cup a 1 [5]. We found that Cup a 1 efficiently inhibited IgE binding to itself. In general, Japanese sera had very low levels of IgE to Jun a 1 therefore the inhibition ELISA gave low signals and was hard to interpret. Nevertheless, Cup a 1 and Jun a 1 inhibited IgE binding to coated Jun a 1 most efficiently. When Cry j 1 was used as coating antigen, this allergen was the only pectate lyase that could efficiently inhibit IgE binding. Therefore, we found that there is only limited IgE cross-reactivity between Cry j 1 and the Cup a 1/Jun a 1 group. Japanese atopics seem to be primarily sensitized by Japanese cedar pollen; however there is relevant co-sensitization with cypress pollen-derived pectate lyasese—most likely with Cha o 1.

We further investigated pectate lyase cross-reactivity in a large cohort from Italy, which are mainly exposed to cypress pollen as major allergen source by testing IgE reactivity to Amb a 1, Cup a 1, and Cry j 1. Thereby, we found essentially the same picture as with ELISA experiments. The vast majority of the patients had strong IgE levels against Cup a 1 which were to a certain degree cross-reactive with Cry j 1. Sensitization to Amb a 1 was rare and did not correlate with IgE reactivity to either Cup a 1 or Cry j 1.

Despite the extremely low specific IgE levels of Italian patients towards Cry j 1 and Japanese patients towards Cup a 1 we could observe that IgE cross-reactivity was mainly restricted to botanical orders. We did not exclude this data because even the variable inhibition results of the two data sets reflected the picture observed in all the other inhibition ELISA experiments—a clustering of IgE cross-reactivity within plant orders, families, and sub-families.

To get a more detailed picture about the sensitizing capacity of each pectate lyase pollen allergen, an animal model of allergic sensitization was established. When looking at IgG1, Amb a 1, Art v 6, and Cry j 1 showed high immunogenicity, whereas Cup a 1 and Jun a 1 were weak immunogens. The cross-reactivity of Amb a 1-induced IgG1 with Art v 6 was high, vice versa it was lower, indicating that antibody epitopes of Amb a 1 and Art v 6 are slightly divergent. Between Cup a 1 and Jun a 1 we found extremely high levels of cross-reactivity, whereas Cry j 1 had a rather independent antibody profile. Of note, despite low immunogenicity at the IgG1 level, Cup a 1 and Jun a 1 were most efficient in inducing allergen-specific IgG2a antibodies. At the IgE level the picture was slightly different. Amb a 1 induced high levels of biologically active IgE levels only against itself. Art v 6-specific IgE were slightly cross-reactive with Amb a 1. Cup a 1 induced cross-reactive IgE with Jun a 1 and—though weaker—this was also observed vice versa, and Cry j 1 induced a very strong IgE response only against itself. In proliferation assays we found essentially the same pattern for cross-reactivity as we saw in ELISA or microarray experiments; however cross-reactivity was limited in general.

## Conclusion

In summary, we could cluster our pectate lyase allergens in four categories, which were Amb a 1, Art v 6, Cup a 1/Jun a 1, and Cry j 1. We found hardly any cross-reactivity between Asteraceae as well as Cupressaceae pectate lyases. However, within the plant orders the allergens were cross-reactive. We could further demonstrate that members of each allergen category can induce sensitization in humans, which raises the question on how to simplify the diagnosis and therapy for pectate lyase sensitized patients. Based on our findings, we consider that it will be necessary to include a member of each of the four categories for effective diagnosis and, considering individual exposure pattern, also for therapeutic applications. In the clinic, the allergenic potential of the minor mugwort pollen allergen Art v 6 seems to be dampened by the low expression levels in pollen extracts.

## Supporting Information

S1 File **Table A.** Demographic and diagnostic data of pectate lyase allergic patients included in the study. **Table B.** Demographic and diagnostic data of Italian pectate lyase allergic patients involved in the microarray survey.
(DOCX)
