# Supplementary material for: Pectate Lyase Pollen Allergens: Sensitization Profiles and Cross-Reactivity Pattern
Source: PLoS One. 2015 May 15;10(5):e0120038. doi: 10.1371/journal.pone.0120038 (PMC4433284; doi:10.1371/journal.pone.0120038)
Supplement: S1 File — Table A. Demographic and diagnostic data of pectate lyase allergic patients included in the study. Table B. Demographic and diagnostic data of Italian pectate lyase allergic patients involved in the microarray survey. (DOCX) [file pone.0120038.s001.docx]

**SUPPORTING INFORMATION**

**Pectate lyase pollen allergens: Sensitization profiles and cross-reactivity pattern**

Ulrike Pichler^1*^, Michael Hauser^1*^, Martin Wolf^1*^, Maria Livia Bernardi^2,3^, Gabriele Gadermaier^1^, Richard Weiss^4^, Christof Ebner^5^, Hidenori Yokoi^6^, Toshiro Takai^7^, Alain Didierlaurent^8^, Chiara Rafaiani^2,3^, Peter Briza^4^, Adriano Mari^2,3^, Heidrun Behrendt^9^, Michael Wallner^1^, Fátima Ferreira^1^

1 Christian Doppler Laboratory for Allergy Diagnosis and Therapy, Department of Molecular Biology, University of Salzburg, Salzburg, Austria

2 Associated Centers for Molecular Allergology, Rome, Italy

3 Centro di Allergologia Molecolare, IDI-IRCCS, Rome, Italy

4 Department of Molecular Biology, University of Salzburg, Salzburg, Austria

5 Allergieambulatorium am Reumanplatz, Vienna, Austria

6 Department of Otolaryngology, Kyorin University, School of Medicine, Tokyo, Japan

7 Atopy (Allergy) Research Center, Juntendo University, Graduate School of Medicine, Tokyo, Japan

8 Stallergenes S.A., Antony, France

9 ZAUM, Center for Allergy and Environment, Munich, Germany

* the authors contributed equally to the work

Corresponding author:

Dr. Fatima Ferreira

Department of Molecular Biology

University of Salzburg

Hellbrunnerstrasse 34

5020 Salzburg, Austria

[fatima.ferreira@sbg.ac.at](mailto:fatima.ferreira@sbg.ac.at)

phone: +43 662 8044 5016

fax: +43 662 8044 745011

**Material and Methods**

**Animal welfare and animal housing:**

Mice were kept in individually vented IVC modules in the SPF (special pathogen free) animal facilities at the University of Salzburg. Food and water was given *ad libitum*. Animals were kept in groups of five per IVC module at 20-24°C and a relative humidity of 55%. Bedding and water was changed weekly. The light/dark cycles were set in 12 h intervals with a 30 min dim period from 0-200 lux and *vice versa*. Blood samples were taken by punctuation of the *vena facialis*. For analyses of splenocytes animals were sacrificed via cervical dislocation.

**Table A.** Demographic and diagnostic data of pectate lyase allergic patients included in the study.

**Table B.** Demographic and diagnostic data of Italian pectate lyase allergic patients involved in the microarray survey.

**Table A.**

|  | Patient Code | Country | Sex | Age | CAP w1 (ragweed) kU/L | ISAC nCup a 1 kU/L | CAP t17 (Japanese cedar) kU/L | CAP w6 (mugwort) kU/L | Used for inhibition |
| --- | --- | --- | --- | --- | --- | --- | --- | --- | --- |
|  |  |  |  |  |  |  |  |  |  |
|  |  |  |  |  |  |  |  |  |  |
| 1 | C1-1 | Canada |  |  | 3,74 |  |  |  |  |
| 2 | C1-3 | Canada |  |  | 29 |  |  |  | X |
| 3 | C1-6 | Canada |  |  | 47,7 |  |  |  |  |
| 4 | C1-8 | Canada |  |  | 24,6 |  |  |  |  |
| 5 | C1-9 | Canada |  |  | 23,9 |  |  |  | X |
| 6 | C1-11 | Canada |  |  | 27,5 |  |  |  |  |
| 7 | C1-12 | Canada |  |  | >100 |  |  |  |  |
| 8 | C1-13 | Canada |  |  | 68,9 |  |  |  |  |
| 9 | C2-19 | Canada |  |  | 40,8 |  |  |  | X |
| 10 | C3-26 | Canada |  |  | 99,4 |  |  |  |  |
| 11 | C3-27 | Canada |  |  | 89,6 |  |  |  |  |
| 12 | C4-29 | Canada |  |  | 41,3 |  |  |  |  |
| 13 | C4-31 | Canada |  |  | 65,3 |  |  |  |  |
| 14 | C5-36 | Canada |  |  | **86,6** |  |  |  |  |
| 15 | C5-38 | Canada |  |  | 81,1 |  |  |  | X |
| 16 | C5-42 | Canada |  |  | 25,6 |  |  |  |  |
| 17 | C5-48 | Canada |  |  | 63,6 |  |  |  |  |
| 18 | C5-54 | Canada |  |  | 22,8 |  |  |  |  |
| 19 | C5-55 | Canada |  |  | >100 |  |  |  |  |
| 20 | C6-58 | Canada |  |  | 32,5 |  |  |  | X |
| 21 | C6-59 | Canada |  |  | >100 |  |  |  |  |
| 22 | C6-60 | Canada |  |  | 20,4 |  |  |  |  |
| 23 | C6-61 | Canada |  |  | 24,8 |  |  |  |  |
| 24 | C6-66 | Canada |  |  | 34,8 |  |  |  |  |
| 25 | C6-68 | Canada |  |  | 27,8 |  |  |  |  |
| 26 | C6-69 | Canada |  |  | 63,2 |  |  |  | X |
| 27 | C7-72 | Canada |  |  | 62,6 |  |  |  |  |
| 28 | C7-73 | Canada |  |  | 50,1 |  |  |  |  |
| 29 | C7-74 | Canada |  |  | 24,6 |  |  |  |  |
| 30 | C11-76 | Canada |  |  | 30,1 |  |  |  |  |
| 31 | C11-78 | Canada |  |  | >100 |  |  |  |  |
| 32 | C11-80 | Canada |  |  | 42,3 |  |  |  |  |
| 33 | C11-82 | Canada |  |  | 16,7 |  |  |  |  |
| 34 | C8-75 | Canada |  |  | 17,6 |  |  |  |  |
| 35 | C5-47 | Canada |  |  | 17,5 |  |  |  |  |
| 36 | C5-50 | Canada |  |  | 19,9 |  |  |  |  |
| 37 | C5-49 | Canada |  |  | 18,4 |  |  |  |  |
| 38 | C5-35 | Canada |  |  | 13,3 |  |  |  |  |
| 39 | C4-32 | Canada |  |  | 23,1 |  |  |  |  |
| 40 | C3-23 | Canada |  |  | 12,1 |  |  |  |  |
|  |  |  |  |  |  |  |  |  |  |
| 1 | I-01 | Italy | F | 22 |  | 5,48 |  |  |  |
| 2 | I-02 | Italy | F | 44 |  | 8,20 |  |  |  |
| 3 | I-03 | Italy | F | 59 |  | 7,62 |  |  |  |
| 4 | I-04 | Italy | M | 44 |  | 5,08 |  |  |  |
| 5 | I-05 | Italy | M | 55 |  | 5,07 |  |  |  |
| 6 | I-06 | Italy | F | 35 |  | 13,96 |  |  |  |
| 7 | I-07 | Italy | F | 64 |  | 6,73 |  |  |  |
| 8 | I-08 | Italy | M | 42 |  | 4,62 |  |  |  |
| 9 | I-09 | Italy | M | 25 |  | 8,37 |  |  |  |
| 10 | I-10 | Italy | F | 45 |  | 26,20 |  |  |  |
| 11 | I-11 | Italy | F | 28 |  | 3,56 |  |  |  |
| 12 | I-12 | Italy | F | 40 |  | 7,35 |  |  |  |
| 13 | I-13 | Italy | M | 25 |  | 5,82 |  |  | X |
| 14 | I-14 | Italy | F | 31 |  | 6,03 |  |  | X |
| 15 | I-15 | Italy | M | 35 |  | 3,59 |  |  |  |
| 16 | I-16 | Italy | M | 55 |  | 17,93 |  |  | X |
| 17 | I-17 | Italy | F | 18 |  | 21,49 |  |  | X |
| 18 | I-18 | Italy | F | 34 |  | 3,76 |  |  | X |
| 19 | I-19 | Italy | F | 69 |  | 9,99 |  |  | X |
| 20 | I-20 | Italy | F | 35 |  | 7,14 |  |  |  |
| 21 | I-21 | Italy | F | 59 |  | 13,86 |  |  |  |
| 22 | I-22 | Italy | F | 46 |  | 4,17 |  |  |  |
| 23 | I-23 | Italy | F | 24 |  | 3,72 |  |  |  |
| 24 | I-24 | Italy | F | 49 |  | 18,44 |  |  |  |
| 25 | I-25 | Italy | F | 36 |  | 16,69 |  |  |  |
| 26 | I-26 | Italy | F | 30 |  | 4,78 |  |  |  |
| 27 | I-27 | Italy | F | 43 |  | 4,47 |  |  |  |
| 28 | I-28 | Italy | M | 38 |  | 9,15 |  |  |  |
| 29 | I-29 | Italy | M | 18 |  | 5,59 |  |  |  |
| 30 | I-30 | Italy | M | 49 |  | 4,23 |  |  |  |
|  |  |  |  |  |  |  |  |  |  |
| 1 | A-02 | Austria | f | 69 | 33,6 |  |  | 19,8 |  |
| 2 | A-03 | Austria | m | 32 | 21,3 |  |  | 3,05 | X |
| 3 | A-04 | Austria | f | 12 | 17,8 |  |  |  |  |
| 4 | A-06 | Austria | f | 42 | 20,4 |  |  | 21,9 |  |
| 5 | A-07 | Austria | f | 44 | 19 |  |  | 1,04 |  |
| 6 | A-09 | Austria | f | 42 | 40,6 |  |  | 7,35 | X |
| 7 | A-11 | Austria | m | 33 | 49,8 |  |  | 3,12 |  |
| 8 | A-12 | Austria | m | 30 | 27,9 |  |  |  |  |
| 9 | A-13 | Austria | m | 13 | >100 |  |  | 32 |  |
| 10 | A-14 | Austria | m | 35 | 33,4 |  |  | 3,24 |  |
| 11 | A-15 | Austria | m | 25 | 91,6 |  |  | 18,4 | X |
| 12 | A-16 | Austria | m | 18 | 21,9 |  |  |  |  |
| 13 | A-18 | Austria | f | 66 | 21,3 |  |  | 26,6 |  |
| 14 | A-19 | Austria | m | 20 | 57,5 |  |  | 31,1 | X |
| 15 | A-20 | Austria | f | 35 | 34 |  |  | 6,61 |  |
| 16 | A-21 | Austria | f | 15 | 95,3 |  |  | 14,5 |  |
| 17 | A-23 | Austria | f | 46 | 32,7 |  |  | 72,5 |  |
| 18 | A-25 | Austria | m | 14 | 25,4 |  |  | 0,59 |  |
| 19 | A-26 | Austria | m | 42 | 30,6 |  |  | 24,9 |  |
| 20 | A-28 | Austria | m | 57 | 43,5 |  |  | 4,48 | X |
| 21 | A-30 | Austria | m | 13 | 28,7 |  |  | 0,49 |  |
| 22 | A-31 | Austria | f | 12 | 51,1 |  |  | 44,1 |  |
| 23 | A-32 | Austria | f | 44 | 34,2 |  |  | 25 |  |
| 24 | A-33 | Austria | f | 47 | 28,7 |  |  | 12,4 |  |
| 25 | A-34 | Austria | m | 9 | 36 |  |  |  |  |
| 26 | A-36 | Austria | f | 44 | 19,2 |  |  | 31,7 |  |
| 27 | A-37 | Austria | m | 34 | >100 |  |  | 30,4 |  |
| 28 | A-38 | Austria | f | 19 | 30,2 |  |  | 1,08 | X |
|  |  |  |  |  |  |  |  |  |  |
| 1 | J-01 | Japan | m | 42 |  |  | 2,52 |  |  |
| 2 | J-02 | Japan | f | 33 |  |  | 14,4 |  |  |
| 4 | J-04 | Japan | m | 18 |  |  | 12,4 |  |  |
| 5 | J-05 | Japan | f | 37 |  |  | 1,78 |  |  |
| 8 | J-09 | Japan | f | 46 |  |  | 13,7 |  |  |
| 9 | J-10 | Japan | m | 23 |  |  | 85,8 |  | X |
| 11 | J-13 | Japan | m | 62 |  |  | 26,3 |  |  |
| 12 | J-14 | Japan | m | 37 |  |  | 7,82 |  |  |
| 17 | J-23 | Japan | f | 73 |  |  | 52,9 |  | X |
| 19 | J-25 | Japan | m | 30 |  |  | 1,43 |  |  |
| 22 | J-28 | Japan | m | 16 |  |  | 0,39 |  |  |
| 23 | J-29 | Japan | f | 72 |  |  | 11 |  |  |
| 25 | J-31 | Japan | f | 34 |  |  | 2,43 |  |  |
| 26 | J-33 | Japan | f | 21 |  |  | 26,3 |  |  |
| 27 | J-34 | Japan | m | 22 |  |  | 11,6 |  |  |
| 28 | J-35 | Japan | f | 22 |  |  | 13,9 |  |  |
| 29 | J-36 | Japan | m | 29 |  |  | 53 |  |  |
| 30 | J-37 | Japan | m | 30 |  |  | 0,35 |  |  |
| 31 | J-40 | Japan | f | 27 |  |  | 67,2 |  | X |
| 32 | J-41 | Japan | f | 42 |  |  | 0,37 |  |  |
| 33 | J-42 | Japan | m | 63 |  |  | 0,65 |  |  |
| 34 | J-43 | Japan | f | 24 |  |  | 32 |  |  |
| 35 | J-44 | Japan | f | 39 |  |  | 44,2 |  | X |
| 36 | J-47 | Japan | f | 26 |  |  | 61,3 |  | X |
| 37 | J-51 | Japan | m | 44 |  |  | 3,54 |  |  |
| 38 | J-52 | Japan | m | 58 |  |  | 26,3 |  |  |
| 39 | J-53 | Japan | f | 25 |  |  | 34,3 |  |  |
| 40 | J-56 | Japan | f | 15 |  |  | 14,6 |  | X |

**Table B.**

| Code | Gender | Age | Allergen | IgE (kUA/L) | Allergen | IgE (kUA/L) | Allergen | IgE (kUA/L) |
| --- | --- | --- | --- | --- | --- | --- | --- | --- |
| Italy - CAAM 1 | M | 33 | Amb a 1 | 88,41 | Cry j 1 | 12,62 | Cup a 1 | 2,52 |
| Italy - CAAM 2 | M | 8 | Amb a 1 | 73,13 | Cry j 1 | 0,75 | Cup a 1 | 0 |
| Italy - CAAM 3 | F | 33 | Amb a 1 | 67,19 | Cry j 1 | 0 | Cup a 1 | 0 |
| Italy - CAAM 4 | F | 37 | Amb a 1 | 64,83 | Cry j 1 | 6,95 | Cup a 1 | 1,45 |
| Italy - CAAM 5 | F | 72 | Amb a 1 | 59,43 | Cry j 1 | 10,42 | Cup a 1 | 38,38 |
| Italy - CAAM 6 | M | 17 | Amb a 1 | 54,89 | Cry j 1 | 7,87 | Cup a 1 | 9,44 |
| Italy - CAAM 7 | M | 29 | Amb a 1 | 51,41 | Cry j 1 | 5,87 | Cup a 1 | 0 |
| Italy - CAAM 8 | F | 44 | Amb a 1 | 50,9 | Cry j 1 | 0 | Cup a 1 | 0,07 |
| Italy - CAAM 9 | M | 5 | Amb a 1 | 49,94 | Cry j 1 | 11,09 | Cup a 1 | 0 |
| Italy - CAAM 10 | M | 20 | Amb a 1 | 48,1 | Cry j 1 | 15,85 | Cup a 1 | 0,02 |
| Italy - CAAM 11 | M | 19 | Amb a 1 | 42,27 | Cry j 1 | 15,31 | Cup a 1 | 1,04 |
| Italy - CAAM 12 | M | 47 | Amb a 1 | 41,43 | Cry j 1 | 0,55 | Cup a 1 | 0 |
| Italy - CAAM 13 | M | 45 | Amb a 1 | 41,06 | Cry j 1 | 2,1 | Cup a 1 | 0 |
| Italy - CAAM 14 | M | 32 | Amb a 1 | 37,02 | Cry j 1 | 3,76 | Cup a 1 | 0 |
| Italy - CAAM 15 | F | 16 | Amb a 1 | 36 | Cry j 1 | 0,79 | Cup a 1 | 1,14 |
| Italy - CAAM 16 | M | 38 | Amb a 1 | 35,54 | Cry j 1 | 2,64 | Cup a 1 | 0 |
| Italy - CAAM 17 | M | 28 | Amb a 1 | 34,97 | Cry j 1 | 1,2 | Cup a 1 | 6,65 |
| Italy - CAAM 18 | M | 73 | Amb a 1 | 33,74 | Cry j 1 | 2,17 | Cup a 1 | 0 |
| Italy - CAAM 19 | F | 14 | Amb a 1 | 33,62 | Cry j 1 | 6,12 | Cup a 1 | 12,05 |
| Italy - CAAM 20 | M | 11 | Amb a 1 | 33,53 | Cry j 1 | 2,75 | Cup a 1 | 5,79 |
| Italy - CAAM 21 | F | 34 | Amb a 1 | 33,08 | Cry j 1 | 0 | Cup a 1 | 0 |
| Italy - CAAM 22 | F | 41 | Amb a 1 | 32,95 | Cry j 1 | 13,99 | Cup a 1 | 0 |
| Italy - CAAM 23 | M | 12 | Amb a 1 | 32,86 | Cry j 1 | 1,84 | Cup a 1 | 2,96 |
| Italy - CAAM 24 | M | 16 | Amb a 1 | 32,54 | Cry j 1 | 12,21 | Cup a 1 | 4,14 |
| Italy - CAAM 25 | M | 16 | Amb a 1 | 31,79 | Cry j 1 | 0,56 | Cup a 1 | 0,48 |
| Italy - CAAM 26 | F | 11 | Amb a 1 | 31,5 | Cry j 1 | 0 | Cup a 1 | 0 |
| Italy - CAAM 27 | M | 42 | Amb a 1 | 30,93 | Cry j 1 | 4,12 | Cup a 1 | 3,63 |
| Italy - CAAM 28 | F | 56 | Amb a 1 | 30,32 | Cry j 1 | 0 | Cup a 1 | 0 |
| Italy - CAAM 29 | M | 10 | Amb a 1 | 29,34 | Cry j 1 | 1,85 | Cup a 1 | 0 |
| Italy - CAAM 30 | F | 54 | Amb a 1 | 28,7 | Cry j 1 | 0 | Cup a 1 | 0 |
| Italy - CAAM 31 | F | 65 | Amb a 1 | 28,47 | Cry j 1 | 8,14 | Cup a 1 | 0,02 |
| Italy - CAAM 32 | F | 38 | Amb a 1 | 27,38 | Cry j 1 | 0 | Cup a 1 | 0 |
| Italy - CAAM 33 | M | 56 | Amb a 1 | 27,05 | Cry j 1 | 10,89 | Cup a 1 | 5,43 |
| Italy - CAAM 34 | M | 33 | Amb a 1 | 26,53 | Cry j 1 | 0,09 | Cup a 1 | 0,21 |
| Italy - CAAM 35 | F | 56 | Amb a 1 | 25,8 | Cry j 1 | 0 | Cup a 1 | 0 |
| Italy - CAAM 36 | F | 32 | Amb a 1 | 25,32 | Cry j 1 | 18,88 | Cup a 1 | 4,29 |
| Italy - CAAM 37 | M | 25 | Amb a 1 | 24,75 | Cry j 1 | 1,05 | Cup a 1 | 0 |
| Italy - CAAM 38 | F | 28 | Amb a 1 | 24,74 | Cry j 1 | 0 | Cup a 1 | 0 |
| Italy - CAAM 39 | M | 31 | Amb a 1 | 24,61 | Cry j 1 | 0,99 | Cup a 1 | 0 |
| Italy - CAAM 40 | F | 45 | Amb a 1 | 24,37 | Cry j 1 | 33,35 | Cup a 1 | 3,01 |
| Italy - CAAM 41 | F | 34 | Amb a 1 | 21,93 | Cry j 1 | 0,48 | Cup a 1 | 0 |
| Italy - CAAM 42 | M | 6 | Amb a 1 | 21,82 | Cry j 1 | 0 | Cup a 1 | 0 |
| Italy - CAAM 43 | F | 34 | Amb a 1 | 21,68 | Cry j 1 | 0 | Cup a 1 | 0 |
| Italy - CAAM 44 | M | 46 | Amb a 1 | 21,3 | Cry j 1 | 0 | Cup a 1 | 0 |
| Italy - CAAM 45 | F | 38 | Amb a 1 | 21,13 | Cry j 1 | 0 | Cup a 1 | 0 |
| Italy - CAAM 46 | M | 33 | Amb a 1 | 20,51 | Cry j 1 | 0 | Cup a 1 | 0 |
| Italy - CAAM 47 | M | 13 | Amb a 1 | 20,4 | Cry j 1 | 0 | Cup a 1 | 0 |
| Italy - CAAM 48 | F | 32 | Amb a 1 | 19,8 | Cry j 1 | 0 | Cup a 1 | 0 |
| Italy - CAAM 49 | F | 12 | Amb a 1 | 19,75 | Cry j 1 | 6,41 | Cup a 1 | 2,35 |
| Italy - CAAM 50 | F | 19 | Amb a 1 | 19,68 | Cry j 1 | 1,72 | Cup a 1 | 5,83 |
| Italy - CAAM 51 | M | 37 | Amb a 1 | 19,57 | Cry j 1 | 0 | Cup a 1 | 0 |
| Italy - CAAM 52 | F | 9 | Amb a 1 | 19,25 | Cry j 1 | 0 | Cup a 1 | 0 |
| Italy - CAAM 53 | F | 59 | Amb a 1 | 18,77 | Cry j 1 | 1,47 | Cup a 1 | 4,1 |
| Italy - CAAM 54 | F | 37 | Amb a 1 | 17,84 | Cry j 1 | 0 | Cup a 1 | 0 |
| Italy - CAAM 55 | F | 10 | Amb a 1 | 17,79 | Cry j 1 | 3,9 | Cup a 1 | 1,03 |
| Italy - CAAM 56 | M | 11 | Amb a 1 | 17,76 | Cry j 1 | 0,8 | Cup a 1 | 0 |
| Italy - CAAM 57 | M | 4 | Amb a 1 | 16,74 | Cry j 1 | 0,21 | Cup a 1 | 1,97 |
| Italy - CAAM 58 | F | 17 | Amb a 1 | 16,69 | Cry j 1 | 11,23 | Cup a 1 | 0,82 |
| Italy - CAAM 59 | M | 15 | Amb a 1 | 16,11 | Cry j 1 | 5,93 | Cup a 1 | 2,26 |
| Italy - CAAM 60 | M | 43 | Amb a 1 | 16,1 | Cry j 1 | 0 | Cup a 1 | 0 |
| Italy - CAAM 61 | M | 11 | Amb a 1 | 15,46 | Cry j 1 | 1,39 | Cup a 1 | 0 |
| Italy - CAAM 62 | F | 30 | Amb a 1 | 15,18 | Cry j 1 | 8,13 | Cup a 1 | 10,86 |
| Italy - CAAM 63 | F | 67 | Amb a 1 | 14,99 | Cry j 1 | 0 | Cup a 1 | 0 |
| Italy - CAAM 64 | M | 48 | Amb a 1 | 14,86 | Cry j 1 | 5 | Cup a 1 | 0,49 |
| Italy - CAAM 65 | F | 40 | Amb a 1 | 14,76 | Cry j 1 | 0,15 | Cup a 1 | 0,45 |
| Italy - CAAM 66 | F | 14 | Amb a 1 | 14,31 | Cry j 1 | 2,39 | Cup a 1 | 0 |
| Italy - CAAM 67 | F | 17 | Amb a 1 | 14,29 | Cry j 1 | 0,35 | Cup a 1 | 1,19 |
| Italy - CAAM 68 | M | 16 | Amb a 1 | 13,89 | Cry j 1 | 9,17 | Cup a 1 | 4,36 |
| Italy - CAAM 69 | M | 21 | Amb a 1 | 13,62 | Cry j 1 | 0 | Cup a 1 | 0 |
| Italy - CAAM 70 | F | 9 | Amb a 1 | 13,59 | Cry j 1 | 1,75 | Cup a 1 | 1,67 |
| Italy - CAAM 71 | M | 3 | Amb a 1 | 13,47 | Cry j 1 | 1,7 | Cup a 1 | 1,31 |
| Italy - CAAM 72 | F | 29 | Amb a 1 | 13,21 | Cry j 1 | 2,92 | Cup a 1 | 1,12 |
| Italy - CAAM 73 | M | 2 | Amb a 1 | 12,99 | Cry j 1 | 0 | Cup a 1 | 0 |
| Italy - CAAM 74 | F | 75 | Amb a 1 | 12,89 | Cry j 1 | 0 | Cup a 1 | 0 |
| Italy - CAAM 75 | M | 9 | Amb a 1 | 12,81 | Cry j 1 | 7,82 | Cup a 1 | 7,8 |
| Italy - CAAM 76 | M | 5 | Amb a 1 | 12,53 | Cry j 1 | 0 | Cup a 1 | 0,29 |
| Italy - CAAM 77 | F | 29 | Amb a 1 | 12,5 | Cry j 1 | 31,01 | Cup a 1 | 26,58 |
| Italy - CAAM 78 | F | 31 | Amb a 1 | 12,26 | Cry j 1 | 0 | Cup a 1 | 0 |
| Italy - CAAM 79 | M | 11 | Amb a 1 | 11,89 | Cry j 1 | 0,35 | Cup a 1 | 0,76 |
| Italy - CAAM 80 | F | 54 | Amb a 1 | 11,79 | Cry j 1 | 2,35 | Cup a 1 | 1,45 |
| Italy - CAAM 81 | F | 33 | Amb a 1 | 11,78 | Cry j 1 | 0 | Cup a 1 | 0 |
| Italy - CAAM 82 | F | 39 | Amb a 1 | 10,82 | Cry j 1 | 0 | Cup a 1 | 3,65 |
| Italy - CAAM 83 | M | 26 | Amb a 1 | 10,82 | Cry j 1 | 6,06 | Cup a 1 | 7,94 |
| Italy - CAAM 84 | M | 46 | Amb a 1 | 10,63 | Cry j 1 | 0 | Cup a 1 | 0 |
| Italy - CAAM 85 | F | 36 | Amb a 1 | 10,63 | Cry j 1 | 0 | Cup a 1 | 0 |
| Italy - CAAM 86 | M | 39 | Amb a 1 | 10,53 | Cry j 1 | 0,74 | Cup a 1 | 0 |
| Italy - CAAM 87 | M | 38 | Amb a 1 | 10,31 | Cry j 1 | 3,11 | Cup a 1 | 13,99 |
| Italy - CAAM 88 | F | 17 | Amb a 1 | 10,25 | Cry j 1 | 15,74 | Cup a 1 | 8,08 |
| Italy - CAAM 89 | F | 40 | Amb a 1 | 10,17 | Cry j 1 | 0 | Cup a 1 | 0 |
| Italy - CAAM 90 | F | 17 | Amb a 1 | 10,01 | Cry j 1 | 8,32 | Cup a 1 | 18,22 |
| Italy - CAAM 91 | F | 6 | Amb a 1 | 9,91 | Cry j 1 | 0 | Cup a 1 | 0 |
| Italy - CAAM 92 | F | 35 | Amb a 1 | 9,84 | Cry j 1 | 1,51 | Cup a 1 | 4,03 |
| Italy - CAAM 93 | M | 30 | Amb a 1 | 9,51 | Cry j 1 | 16,12 | Cup a 1 | 25,19 |
| Italy - CAAM 94 | F | 33 | Amb a 1 | 9,5 | Cry j 1 | 0,32 | Cup a 1 | 0 |
| Italy - CAAM 95 | F | 28 | Amb a 1 | 9,46 | Cry j 1 | 0,85 | Cup a 1 | 10,08 |
| Italy - CAAM 96 | F | 11 | Amb a 1 | 9,26 | Cry j 1 | 0 | Cup a 1 | 0 |
| Italy - CAAM 97 | F | 34 | Amb a 1 | 9,08 | Cry j 1 | 0,74 | Cup a 1 | 2,03 |
| Italy - CAAM 98 | M | 44 | Amb a 1 | 9,06 | Cry j 1 | 0 | Cup a 1 | 0 |
| Italy - CAAM 99 | F | 41 | Amb a 1 | 8,95 | Cry j 1 | 0 | Cup a 1 | 0 |
| Italy - CAAM 100 | F | 41 | Amb a 1 | 8,95 | Cry j 1 | 4,4 | Cup a 1 | 0,87 |
| Italy - CAAM 101 | F | 43 | Amb a 1 | 8,87 | Cry j 1 | 11,6 | Cup a 1 | 0,07 |
| Italy - CAAM 102 | F | 34 | Amb a 1 | 8,59 | Cry j 1 | 0 | Cup a 1 | 0 |
| Italy - CAAM 103 | F | 33 | Amb a 1 | 8,59 | Cry j 1 | 0,05 | Cup a 1 | 0,11 |
| Italy - CAAM 104 | F | 66 | Amb a 1 | 8,39 | Cry j 1 | 0,35 | Cup a 1 | 2,49 |
| Italy - CAAM 105 | M | 56 | Amb a 1 | 8,24 | Cry j 1 | 4,83 | Cup a 1 | 0 |
| Italy - CAAM 106 | M | 22 | Amb a 1 | 8,11 | Cry j 1 | 0 | Cup a 1 | 0 |
| Italy - CAAM 107 | M | 48 | Amb a 1 | 8,01 | Cry j 1 | 1,62 | Cup a 1 | 0,88 |
| Italy - CAAM 108 | F | 28 | Amb a 1 | 7,83 | Cry j 1 | 0 | Cup a 1 | 0 |
| Italy - CAAM 109 | F | 19 | Amb a 1 | 7,78 | Cry j 1 | 0,18 | Cup a 1 | 3,96 |
| Italy - CAAM 110 | M | 17 | Amb a 1 | 7,78 | Cry j 1 | 1,83 | Cup a 1 | 0 |
| Italy - CAAM 111 | M | 8 | Amb a 1 | 7,71 | Cry j 1 | 0,2 | Cup a 1 | 0,28 |
| Italy - CAAM 112 | F | 13 | Amb a 1 | 7,62 | Cry j 1 | 0 | Cup a 1 | 0 |
| Italy - CAAM 113 | F | 16 | Amb a 1 | 7,32 | Cry j 1 | 0,26 | Cup a 1 | 0 |
| Italy - CAAM 114 | M | 22 | Amb a 1 | 7,15 | Cry j 1 | 0,25 | Cup a 1 | 0,97 |
| Italy - CAAM 115 | F | 35 | Amb a 1 | 6,9 | Cry j 1 | 1,67 | Cup a 1 | 1,73 |
| Italy - CAAM 116 | F | 18 | Amb a 1 | 6,57 | Cry j 1 | 10,58 | Cup a 1 | 1,6 |
| Italy - CAAM 117 | F | 31 | Amb a 1 | 6,24 | Cry j 1 | 0 | Cup a 1 | 0 |
| Italy - CAAM 118 | M | 72 | Amb a 1 | 6,23 | Cry j 1 | 0 | Cup a 1 | 0 |
| Italy - CAAM 119 | M | 38 | Amb a 1 | 5,93 | Cry j 1 | 1,73 | Cup a 1 | 1,78 |
| Italy - CAAM 120 | F | 42 | Amb a 1 | 5,92 | Cry j 1 | 0 | Cup a 1 | 0 |
| Italy - CAAM 121 | F | 70 | Amb a 1 | 5,81 | Cry j 1 | 1,11 | Cup a 1 | 1,12 |
| Italy - CAAM 122 | M | 62 | Amb a 1 | 5,74 | Cry j 1 | 3,66 | Cup a 1 | 7,25 |
| Italy - CAAM 123 | F | 38 | Amb a 1 | 5,72 | Cry j 1 | 0 | Cup a 1 | 0 |
| Italy - CAAM 124 | M | 19 | Amb a 1 | 5,6 | Cry j 1 | 1,41 | Cup a 1 | 1,54 |
| Italy - CAAM 125 | M | 40 | Amb a 1 | 5,57 | Cry j 1 | 0,35 | Cup a 1 | 4,06 |
| Italy - CAAM 126 | M | 11 | Amb a 1 | 5,55 | Cry j 1 | 0 | Cup a 1 | 0 |
| Italy - CAAM 127 | M | 53 | Amb a 1 | 5,3 | Cry j 1 | 8,9 | Cup a 1 | 101 |
| Italy - CAAM 128 | F | 28 | Amb a 1 | 5,28 | Cry j 1 | 0,08 | Cup a 1 | 0,49 |
| Italy - CAAM 129 | F | 24 | Amb a 1 | 5,04 | Cry j 1 | 0,29 | Cup a 1 | 2,68 |
| Italy - CAAM 130 | M | 12 | Amb a 1 | 4,97 | Cry j 1 | 0,88 | Cup a 1 | 0,37 |
| Italy - CAAM 131 | M | 22 | Amb a 1 | 4,93 | Cry j 1 | 30,37 | Cup a 1 | 74,73 |
| Italy - CAAM 132 | M | 47 | Amb a 1 | 4,88 | Cry j 1 | 0,72 | Cup a 1 | 0 |
| Italy - CAAM 133 | M | 44 | Amb a 1 | 4,71 | Cry j 1 | 0 | Cup a 1 | 0 |
| Italy - CAAM 134 | M | 52 | Amb a 1 | 4,61 | Cry j 1 | 0 | Cup a 1 | 0 |
| Italy - CAAM 135 | M | 46 | Amb a 1 | 4,58 | Cry j 1 | 0 | Cup a 1 | 0 |
| Italy - CAAM 136 | F | 24 | Amb a 1 | 4,53 | Cry j 1 | 0 | Cup a 1 | 0 |
| Italy - CAAM 137 | F | 43 | Amb a 1 | 4,37 | Cry j 1 | 1,36 | Cup a 1 | 0 |
| Italy - CAAM 138 | M | 47 | Amb a 1 | 4,36 | Cry j 1 | 0 | Cup a 1 | 0 |
| Italy - CAAM 139 | F | 27 | Amb a 1 | 4,34 | Cry j 1 | 0 | Cup a 1 | 2,29 |
| Italy - CAAM 140 | F | 34 | Amb a 1 | 4,33 | Cry j 1 | 6,91 | Cup a 1 | 11,02 |
| Italy - CAAM 141 | F | 18 | Amb a 1 | 4,23 | Cry j 1 | 12,35 | Cup a 1 | 38,71 |
| Italy - CAAM 142 | F | 29 | Amb a 1 | 4,22 | Cry j 1 | 0 | Cup a 1 | 0 |
| Italy - CAAM 143 | M | 35 | Amb a 1 | 4,02 | Cry j 1 | 0,15 | Cup a 1 | 0 |
| Italy - CAAM 144 | F | 31 | Amb a 1 | 3,94 | Cry j 1 | 97,76 | Cup a 1 | 104,64 |
| Italy - CAAM 145 | F | 44 | Amb a 1 | 3,84 | Cry j 1 | 0 | Cup a 1 | 4,7 |
| Italy - CAAM 146 | F | 14 | Amb a 1 | 3,83 | Cry j 1 | 0 | Cup a 1 | 0 |
| Italy - CAAM 147 | M | 30 | Amb a 1 | 3,58 | Cry j 1 | 0,56 | Cup a 1 | 2,21 |
| Italy - CAAM 148 | F | 21 | Amb a 1 | 3,58 | Cry j 1 | 0 | Cup a 1 | 0 |
| Italy - CAAM 149 | M | 13 | Amb a 1 | 3,56 | Cry j 1 | 6,83 | Cup a 1 | 12,49 |
| Italy - CAAM 150 | F | 54 | Amb a 1 | 3,54 | Cry j 1 | 0,15 | Cup a 1 | 0 |
| Italy - CAAM 151 | M | 28 | Amb a 1 | 3,53 | Cry j 1 | 0 | Cup a 1 | 0 |
| Italy - CAAM 152 | F | 14 | Amb a 1 | 3,51 | Cry j 1 | 0 | Cup a 1 | 0 |
| Italy - CAAM 153 | M | 13 | Amb a 1 | 3,5 | Cry j 1 | 12,55 | Cup a 1 | 30,75 |
| Italy - CAAM 154 | M | 10 | Amb a 1 | 3,46 | Cry j 1 | 4,32 | Cup a 1 | 40,48 |
| Italy - CAAM 155 | F | 43 | Amb a 1 | 3,42 | Cry j 1 | 0,47 | Cup a 1 | 1,02 |
| Italy - CAAM 156 | F | 35 | Amb a 1 | 3,36 | Cry j 1 | 1,2 | Cup a 1 | 0 |
| Italy - CAAM 157 | M | 12 | Amb a 1 | 3,34 | Cry j 1 | 0 | Cup a 1 | 0 |
| Italy - CAAM 158 | F | 83 | Amb a 1 | 3,32 | Cry j 1 | 0 | Cup a 1 | 0 |
| Italy - CAAM 159 | M | 17 | Amb a 1 | 3,3 | Cry j 1 | 29,63 | Cup a 1 | 33,42 |
| Italy - CAAM 160 | M | 30 | Amb a 1 | 3,28 | Cry j 1 | 0,5 | Cup a 1 | 2,25 |
| Italy - CAAM 161 | F | 58 | Amb a 1 | 3,21 | Cry j 1 | 0,35 | Cup a 1 | 1,77 |
| Italy - CAAM 162 | F | 47 | Amb a 1 | 3,05 | Cry j 1 | 0 | Cup a 1 | 0 |
| Italy - CAAM 163 | M | 43 | Amb a 1 | 2,99 | Cry j 1 | 8,43 | Cup a 1 | 7,43 |
| Italy - CAAM 164 | M | 40 | Amb a 1 | 2,87 | Cry j 1 | 9,05 | Cup a 1 | 0 |
| Italy - CAAM 165 | M | 26 | Amb a 1 | 2,86 | Cry j 1 | 14,79 | Cup a 1 | 18,28 |
| Italy - CAAM 166 | M | 16 | Amb a 1 | 2,86 | Cry j 1 | 6,72 | Cup a 1 | 39,03 |
| Italy - CAAM 167 | M | 47 | Amb a 1 | 2,81 | Cry j 1 | 0 | Cup a 1 | 0 |
| Italy - CAAM 168 | F | 23 | Amb a 1 | 2,78 | Cry j 1 | 0,18 | Cup a 1 | 0 |
| Italy - CAAM 169 | M | 18 | Amb a 1 | 2,73 | Cry j 1 | 0 | Cup a 1 | 0 |
| Italy - CAAM 170 | F | 30 | Amb a 1 | 2,71 | Cry j 1 | 0 | Cup a 1 | 0 |
| Italy - CAAM 171 | F | 31 | Amb a 1 | 2,7 | Cry j 1 | 0 | Cup a 1 | 0 |
| Italy - CAAM 172 | M | 10 | Amb a 1 | 2,61 | Cry j 1 | 0,5 | Cup a 1 | 0,65 |
| Italy - CAAM 173 | F | 58 | Amb a 1 | 2,58 | Cry j 1 | 0 | Cup a 1 | 0 |
| Italy - CAAM 174 | F | 9 | Amb a 1 | 2,57 | Cry j 1 | 11,64 | Cup a 1 | 18,14 |
| Italy - CAAM 175 | F | 31 | Amb a 1 | 2,49 | Cry j 1 | 1,24 | Cup a 1 | 4,94 |
| Italy - CAAM 176 | F | 34 | Amb a 1 | 2,48 | Cry j 1 | 13,17 | Cup a 1 | 25,98 |
| Italy - CAAM 177 | M | 39 | Amb a 1 | 2,45 | Cry j 1 | 0 | Cup a 1 | 0 |
| Italy - CAAM 178 | F | 29 | Amb a 1 | 2,44 | Cry j 1 | 0 | Cup a 1 | 0 |
| Italy - CAAM 179 | F | 8 | Amb a 1 | 2,4 | Cry j 1 | 0 | Cup a 1 | 0 |
| Italy - CAAM 180 | F | 42 | Amb a 1 | 2,37 | Cry j 1 | 3,71 | Cup a 1 | 39,04 |
| Italy - CAAM 181 | M | 17 | Amb a 1 | 2,36 | Cry j 1 | 3,25 | Cup a 1 | 21,03 |
| Italy - CAAM 182 | M | 88 | Amb a 1 | 2,34 | Cry j 1 | 2,45 | Cup a 1 | 24,98 |
| Italy - CAAM 183 | F | 12 | Amb a 1 | 2,22 | Cry j 1 | 0 | Cup a 1 | 0 |
| Italy - CAAM 184 | M | 79 | Amb a 1 | 2,21 | Cry j 1 | 0 | Cup a 1 | 0 |
| Italy - CAAM 185 | M | 28 | Amb a 1 | 2,16 | Cry j 1 | 0 | Cup a 1 | 0 |
| Italy - CAAM 186 | M | 70 | Amb a 1 | 2,08 | Cry j 1 | 44,48 | Cup a 1 | 19,9 |
| Italy - CAAM 187 | F | 56 | Amb a 1 | 1,97 | Cry j 1 | 0 | Cup a 1 | 0 |
| Italy - CAAM 188 | F | 36 | Amb a 1 | 1,96 | Cry j 1 | 0 | Cup a 1 | 0 |
| Italy - CAAM 189 | F | 30 | Amb a 1 | 1,81 | Cry j 1 | 0,6 | Cup a 1 | 1,03 |
| Italy - CAAM 190 | M | 16 | Amb a 1 | 1,79 | Cry j 1 | 8,35 | Cup a 1 | 39,92 |
| Italy - CAAM 191 | M | 47 | Amb a 1 | 1,72 | Cry j 1 | 0 | Cup a 1 | 0 |
| Italy - CAAM 192 | M | 25 | Amb a 1 | 1,69 | Cry j 1 | 10,02 | Cup a 1 | 13,17 |
| Italy - CAAM 193 | M | 76 | Amb a 1 | 1,68 | Cry j 1 | 0 | Cup a 1 | 0 |
| Italy - CAAM 194 | M | 42 | Amb a 1 | 1,64 | Cry j 1 | 0,05 | Cup a 1 | 1,06 |
| Italy - CAAM 195 | M | 34 | Amb a 1 | 1,59 | Cry j 1 | 0 | Cup a 1 | 0 |
| Italy - CAAM 196 | F | 36 | Amb a 1 | 1,57 | Cry j 1 | 0 | Cup a 1 | 1,65 |
| Italy - CAAM 197 | F | 26 | Amb a 1 | 1,52 | Cry j 1 | 0,33 | Cup a 1 | 0,13 |
| Italy - CAAM 198 | F | 63 | Amb a 1 | 1,51 | Cry j 1 | 5,18 | Cup a 1 | 4,33 |
| Italy - CAAM 199 | M | 30 | Amb a 1 | 1,51 | Cry j 1 | 9,76 | Cup a 1 | 56,57 |
| Italy - CAAM 200 | F | 38 | Amb a 1 | 1,48 | Cry j 1 | 2,01 | Cup a 1 | 19,01 |
| Italy - CAAM 201 | M | 7 | Amb a 1 | 1,48 | Cry j 1 | 1,41 | Cup a 1 | 6,34 |
| Italy - CAAM 202 | F | 28 | Amb a 1 | 1,44 | Cry j 1 | 0 | Cup a 1 | 0 |
| Italy - CAAM 203 | F | 38 | Amb a 1 | 1,39 | Cry j 1 | 0,07 | Cup a 1 | 1,19 |
| Italy - CAAM 204 | M | 28 | Amb a 1 | 1,39 | Cry j 1 | 0 | Cup a 1 | 0 |
| Italy - CAAM 205 | M | 18 | Amb a 1 | 1,39 | Cry j 1 | 0 | Cup a 1 | 0 |
| Italy - CAAM 206 | F | 34 | Amb a 1 | 1,37 | Cry j 1 | 2,19 | Cup a 1 | 15,81 |
| Italy - CAAM 207 | F | 52 | Amb a 1 | 1,27 | Cry j 1 | 0,52 | Cup a 1 | 1,37 |
| Italy - CAAM 208 | M | 4 | Amb a 1 | 1,26 | Cry j 1 | 11,54 | Cup a 1 | 9,26 |
| Italy - CAAM 209 | F | 69 | Amb a 1 | 1,24 | Cry j 1 | 0 | Cup a 1 | 0 |
| Italy - CAAM 210 | M | 46 | Amb a 1 | 1,2 | Cry j 1 | 0 | Cup a 1 | 0 |
| Italy - CAAM 211 | M | 21 | Amb a 1 | 1,2 | Cry j 1 | 12,61 | Cup a 1 | 51,65 |
| Italy - CAAM 212 | F | 31 | Amb a 1 | 1,17 | Cry j 1 | 1,15 | Cup a 1 | 0,45 |
| Italy - CAAM 213 | M | 26 | Amb a 1 | 1,15 | Cry j 1 | 8,08 | Cup a 1 | 30,48 |
| Italy - CAAM 214 | M | 13 | Amb a 1 | 1,13 | Cry j 1 | 2,52 | Cup a 1 | 13,47 |
| Italy - CAAM 215 | F | 41 | Amb a 1 | 1,12 | Cry j 1 | 0 | Cup a 1 | 0 |
| Italy - CAAM 216 | M | 19 | Amb a 1 | 1,12 | Cry j 1 | 9,07 | Cup a 1 | 17,28 |
| Italy - CAAM 217 | M | 81 | Amb a 1 | 1,1 | Cry j 1 | 1,48 | Cup a 1 | 19,71 |
| Italy - CAAM 218 | F | 14 | Amb a 1 | 1,1 | Cry j 1 | 0 | Cup a 1 | 0 |
| Italy - CAAM 219 | F | 32 | Amb a 1 | 1,09 | Cry j 1 | 0 | Cup a 1 | 0 |
| Italy - CAAM 220 | M | 61 | Amb a 1 | 1,07 | Cry j 1 | 0 | Cup a 1 | 0 |
| Italy - CAAM 221 | M | 59 | Amb a 1 | 1,07 | Cry j 1 | 2,84 | Cup a 1 | 18,32 |
| Italy - CAAM 222 | M | 22 | Amb a 1 | 1,04 | Cry j 1 | 21,49 | Cup a 1 | 57,84 |
| Italy - CAAM 223 | F | 20 | Amb a 1 | 1,04 | Cry j 1 | 0 | Cup a 1 | 0 |
| Italy - CAAM 224 | F | 62 | Amb a 1 | 1,03 | Cry j 1 | 7,81 | Cup a 1 | 0 |
| Italy - CAAM 225 | F | 30 | Amb a 1 | 1,02 | Cry j 1 | 4,82 | Cup a 1 | 20,46 |
| Italy - CAAM 226 | F | 49 | Amb a 1 | 0,99 | Cry j 1 | 0 | Cup a 1 | 0 |
| Italy - CAAM 227 | M | 41 | Amb a 1 | 0,99 | Cry j 1 | 0,77 | Cup a 1 | 6,4 |
| Italy - CAAM 228 | M | 16 | Amb a 1 | 0,94 | Cry j 1 | 18,01 | Cup a 1 | 11,71 |
| Italy - CAAM 229 | M | 21 | Amb a 1 | 0,91 | Cry j 1 | 0 | Cup a 1 | 4,55 |
| Italy - CAAM 230 | M | 12 | Amb a 1 | 0,9 | Cry j 1 | 1,3 | Cup a 1 | 0,51 |
| Italy - CAAM 231 | M | 70 | Amb a 1 | 0,89 | Cry j 1 | 16,47 | Cup a 1 | 4,3 |
| Italy - CAAM 232 | F | 31 | Amb a 1 | 0,88 | Cry j 1 | 0 | Cup a 1 | 0 |
| Italy - CAAM 233 | F | 46 | Amb a 1 | 0,84 | Cry j 1 | 0 | Cup a 1 | 0 |
| Italy - CAAM 234 | F | 35 | Amb a 1 | 0,82 | Cry j 1 | 4,56 | Cup a 1 | 3,96 |
| Italy - CAAM 235 | F | 19 | Amb a 1 | 0,82 | Cry j 1 | 0 | Cup a 1 | 0 |
| Italy - CAAM 236 | M | 48 | Amb a 1 | 0,8 | Cry j 1 | 4,15 | Cup a 1 | 4,89 |
| Italy - CAAM 237 | F | 46 | Amb a 1 | 0,79 | Cry j 1 | 1,1 | Cup a 1 | 2,14 |
| Italy - CAAM 238 | M | 16 | Amb a 1 | 0,77 | Cry j 1 | 0 | Cup a 1 | 0 |
| Italy - CAAM 239 | M | 35 | Amb a 1 | 0,76 | Cry j 1 | 5,29 | Cup a 1 | 5,98 |
| Italy - CAAM 240 | M | 28 | Amb a 1 | 0,75 | Cry j 1 | 4,27 | Cup a 1 | 0,83 |
| Italy - CAAM 241 | F | 5 | Amb a 1 | 0,75 | Cry j 1 | 0 | Cup a 1 | 0 |
| Italy - CAAM 242 | M | 1 | Amb a 1 | 0,73 | Cry j 1 | 5,72 | Cup a 1 | 11,78 |
| Italy - CAAM 243 | F | 7 | Amb a 1 | 0,72 | Cry j 1 | 0 | Cup a 1 | 0 |
| Italy - CAAM 244 | M | 44 | Amb a 1 | 0,7 | Cry j 1 | 2,49 | Cup a 1 | 17,69 |
| Italy - CAAM 245 | M | 7 | Amb a 1 | 0,7 | Cry j 1 | 2,4 | Cup a 1 | 38 |
| Italy - CAAM 246 | F | 27 | Amb a 1 | 0,69 | Cry j 1 | 0,03 | Cup a 1 | 0,07 |
| Italy - CAAM 247 | F | 36 | Amb a 1 | 0,63 | Cry j 1 | 0 | Cup a 1 | 0 |
| Italy - CAAM 248 | M | 70 | Amb a 1 | 0,61 | Cry j 1 | 35,38 | Cup a 1 | 12,71 |
| Italy - CAAM 249 | M | 52 | Amb a 1 | 0,61 | Cry j 1 | 2,84 | Cup a 1 | 26,24 |
| Italy - CAAM 250 | M | 26 | Amb a 1 | 0,61 | Cry j 1 | 5,8 | Cup a 1 | 33,1 |
| Italy - CAAM 251 | F | 43 | Amb a 1 | 0,6 | Cry j 1 | 0 | Cup a 1 | 0 |
| Italy - CAAM 252 | F | 24 | Amb a 1 | 0,6 | Cry j 1 | 0,3 | Cup a 1 | 0,49 |
| Italy - CAAM 253 | F | 17 | Amb a 1 | 0,59 | Cry j 1 | 8,22 | Cup a 1 | 7,98 |
| Italy - CAAM 254 | M | 66 | Amb a 1 | 0,57 | Cry j 1 | 0 | Cup a 1 | 0,07 |
| Italy - CAAM 255 | F | 19 | Amb a 1 | 0,57 | Cry j 1 | 0 | Cup a 1 | 0 |
| Italy - CAAM 256 | M | 18 | Amb a 1 | 0,57 | Cry j 1 | 6,63 | Cup a 1 | 11,83 |
| Italy - CAAM 257 | M | 33 | Amb a 1 | 0,56 | Cry j 1 | 0,44 | Cup a 1 | 2,3 |
| Italy - CAAM 258 | M | 39 | Amb a 1 | 0,54 | Cry j 1 | 2,66 | Cup a 1 | 16,64 |
| Italy - CAAM 259 | M | 13 | Amb a 1 | 0,51 | Cry j 1 | 13,79 | Cup a 1 | 22,16 |
| Italy - CAAM 260 | M | 14 | Amb a 1 | 0,49 | Cry j 1 | 4,79 | Cup a 1 | 15,4 |
| Italy - CAAM 261 | M | 10 | Amb a 1 | 0,48 | Cry j 1 | 0 | Cup a 1 | 0 |
| Italy - CAAM 262 | M | 40 | Amb a 1 | 0,47 | Cry j 1 | 0,91 | Cup a 1 | 0,85 |
| Italy - CAAM 263 | M | 7 | Amb a 1 | 0,47 | Cry j 1 | 23,45 | Cup a 1 | 17,83 |
| Italy - CAAM 264 | F | 18 | Amb a 1 | 0,46 | Cry j 1 | 11,76 | Cup a 1 | 13,1 |
| Italy - CAAM 265 | M | 5 | Amb a 1 | 0,46 | Cry j 1 | 1,25 | Cup a 1 | 0,73 |
| Italy - CAAM 266 | F | 52 | Amb a 1 | 0,45 | Cry j 1 | 0,77 | Cup a 1 | 5,98 |
| Italy - CAAM 267 | M | 8 | Amb a 1 | 0,45 | Cry j 1 | 0,93 | Cup a 1 | 6,24 |
| Italy - CAAM 268 | F | 45 | Amb a 1 | 0,44 | Cry j 1 | 8,76 | Cup a 1 | 14,72 |
| Italy - CAAM 269 | F | 27 | Amb a 1 | 0,44 | Cry j 1 | 8,11 | Cup a 1 | 12,78 |
| Italy - CAAM 270 | M | 27 | Amb a 1 | 0,44 | Cry j 1 | 0 | Cup a 1 | 0 |
| Italy - CAAM 271 | F | 25 | Amb a 1 | 0,44 | Cry j 1 | 1,84 | Cup a 1 | 14,37 |
| Italy - CAAM 272 | F | 39 | Amb a 1 | 0,43 | Cry j 1 | 0,47 | Cup a 1 | 2,35 |
| Italy - CAAM 273 | M | 7 | Amb a 1 | 0,43 | Cry j 1 | 19,53 | Cup a 1 | 28,64 |
| Italy - CAAM 274 | M | 33 | Amb a 1 | 0,42 | Cry j 1 | 4,94 | Cup a 1 | 8,1 |
| Italy - CAAM 275 | M | 39 | Amb a 1 | 0,41 | Cry j 1 | 3,71 | Cup a 1 | 6,32 |
| Italy - CAAM 276 | M | 11 | Amb a 1 | 0,41 | Cry j 1 | 0 | Cup a 1 | 0 |
| Italy - CAAM 277 | F | 68 | Amb a 1 | 0,4 | Cry j 1 | 0 | Cup a 1 | 0 |
| Italy - CAAM 278 | F | 32 | Amb a 1 | 0,4 | Cry j 1 | 0 | Cup a 1 | 0 |
| Italy - CAAM 279 | M | 11 | Amb a 1 | 0,4 | Cry j 1 | 1,12 | Cup a 1 | 2,7 |
| Italy - CAAM 280 | M | 7 | Amb a 1 | 0,4 | Cry j 1 | 7,2 | Cup a 1 | 41 |
| Italy - CAAM 281 | F | 57 | Amb a 1 | 0,39 | Cry j 1 | 3,47 | Cup a 1 | 15,78 |
| Italy - CAAM 282 | M | 22 | Amb a 1 | 0,39 | Cry j 1 | 35,4 | Cup a 1 | 69,17 |
| Italy - CAAM 283 | F | 11 | Amb a 1 | 0,39 | Cry j 1 | 0,98 | Cup a 1 | 2,23 |
| Italy - CAAM 284 | F | 22 | Amb a 1 | 0,38 | Cry j 1 | 16,01 | Cup a 1 | 24,62 |
| Italy - CAAM 285 | M | 17 | Amb a 1 | 0,38 | Cry j 1 | 0 | Cup a 1 | 0 |
| Italy - CAAM 286 | M | 46 | Amb a 1 | 0,37 | Cry j 1 | 0 | Cup a 1 | 0 |
| Italy - CAAM 287 | F | 24 | Amb a 1 | 0,37 | Cry j 1 | 3,21 | Cup a 1 | 19,24 |
| Italy - CAAM 288 | M | 10 | Amb a 1 | 0,37 | Cry j 1 | 16,05 | Cup a 1 | 15,84 |
| Italy - CAAM 289 | M | 5 | Amb a 1 | 0,37 | Cry j 1 | 0 | Cup a 1 | 0 |
| Italy - CAAM 290 | F | 25 | Amb a 1 | 0,36 | Cry j 1 | 0 | Cup a 1 | 0 |
| Italy - CAAM 291 | F | 10 | Amb a 1 | 0,36 | Cry j 1 | 3,39 | Cup a 1 | 12,92 |
| Italy - CAAM 292 | F | 26 | Amb a 1 | 0,35 | Cry j 1 | 1,26 | Cup a 1 | 4,74 |
| Italy - CAAM 293 | M | 11 | Amb a 1 | 0,35 | Cry j 1 | 0 | Cup a 1 | 0 |
| Italy - CAAM 294 | F | 14 | Amb a 1 | 0,33 | Cry j 1 | 0 | Cup a 1 | 0 |
| Italy - CAAM 295 | F | 13 | Amb a 1 | 0,33 | Cry j 1 | 0 | Cup a 1 | 0 |
| Italy - CAAM 296 | M | 28 | Amb a 1 | 0,32 | Cry j 1 | 5,07 | Cup a 1 | 7,22 |
| Italy - CAAM 297 | M | 9 | Amb a 1 | 0,32 | Cry j 1 | 2,02 | Cup a 1 | 2,62 |
| Italy - CAAM 298 | F | 41 | Amb a 1 | 0,31 | Cry j 1 | 1,24 | Cup a 1 | 3,9 |
| Italy - CAAM 299 | M | 27 | Amb a 1 | 0,3 | Cry j 1 | 3,41 | Cup a 1 | 20,82 |
| Italy - CAAM 300 | M | 23 | Amb a 1 | 0,3 | Cry j 1 | 0 | Cup a 1 | 0 |
| Italy - CAAM 301 | F | 16 | Amb a 1 | 0,3 | Cry j 1 | 1,22 | Cup a 1 | 4,37 |
| Italy - CAAM 302 | M | 11 | Amb a 1 | 0,3 | Cry j 1 | 0 | Cup a 1 | 0 |
| Italy - CAAM 303 | M | 9 | Amb a 1 | 0,3 | Cry j 1 | 1,2 | Cup a 1 | 10,64 |
| Italy - CAAM 304 | M | 9 | Amb a 1 | 0,29 | Cry j 1 | 0 | Cup a 1 | 0,26 |
| Italy - CAAM 305 | M | 61 | Amb a 1 | 0,28 | Cry j 1 | 26,46 | Cup a 1 | 29,14 |
| Italy - CAAM 306 | F | 50 | Amb a 1 | 0,26 | Cry j 1 | 0 | Cup a 1 | 0 |
| Italy - CAAM 307 | M | 42 | Amb a 1 | 0,26 | Cry j 1 | 0 | Cup a 1 | 0,23 |
| Italy - CAAM 308 | F | 5 | Amb a 1 | 0,26 | Cry j 1 | 0 | Cup a 1 | 0 |
| Italy - CAAM 309 | F | 42 | Amb a 1 | 0,25 | Cry j 1 | 1,6 | Cup a 1 | 5,63 |
| Italy - CAAM 310 | M | 25 | Amb a 1 | 0,25 | Cry j 1 | 0 | Cup a 1 | 0 |
| Italy - CAAM 311 | M | 10 | Amb a 1 | 0,25 | Cry j 1 | 23,03 | Cup a 1 | 30,84 |
| Italy - CAAM 312 | F | 65 | Amb a 1 | 0,24 | Cry j 1 | 1,63 | Cup a 1 | 8,42 |
| Italy - CAAM 313 | M | 43 | Amb a 1 | 0,24 | Cry j 1 | 0,06 | Cup a 1 | 1,53 |
| Italy - CAAM 314 | F | 20 | Amb a 1 | 0,24 | Cry j 1 | 1,29 | Cup a 1 | 2,26 |
| Italy - CAAM 315 | F | 49 | Amb a 1 | 0,23 | Cry j 1 | 0 | Cup a 1 | 11,89 |
| Italy - CAAM 316 | M | 33 | Amb a 1 | 0,23 | Cry j 1 | 0 | Cup a 1 | 0 |
| Italy - CAAM 317 | F | 7 | Amb a 1 | 0,22 | Cry j 1 | 0 | Cup a 1 | 0,17 |
| Italy - CAAM 318 | M | 6 | Amb a 1 | 0,22 | Cry j 1 | 13,21 | Cup a 1 | 15,4 |
| Italy - CAAM 319 | F | 63 | Amb a 1 | 0,21 | Cry j 1 | 0,19 | Cup a 1 | 1,81 |
| Italy - CAAM 320 | M | 55 | Amb a 1 | 0,21 | Cry j 1 | 0 | Cup a 1 | 0 |
| Italy - CAAM 321 | F | 14 | Amb a 1 | 0,21 | Cry j 1 | 0,06 | Cup a 1 | 2,6 |
| Italy - CAAM 322 | M | 19 | Amb a 1 | 0,2 | Cry j 1 | 13,59 | Cup a 1 | 33,46 |
| Italy - CAAM 323 | M | 38 | Amb a 1 | 0,19 | Cry j 1 | 5,78 | Cup a 1 | 37,55 |
| Italy - CAAM 324 | M | 36 | Amb a 1 | 0,19 | Cry j 1 | 6,28 | Cup a 1 | 41,09 |
| Italy - CAAM 325 | M | 26 | Amb a 1 | 0,19 | Cry j 1 | 28,04 | Cup a 1 | 31,19 |
| Italy - CAAM 326 | M | 45 | Amb a 1 | 0,18 | Cry j 1 | 0,14 | Cup a 1 | 4,06 |
| Italy - CAAM 327 | F | 40 | Amb a 1 | 0,18 | Cry j 1 | 0 | Cup a 1 | 0 |
| Italy - CAAM 328 | M | 30 | Amb a 1 | 0,18 | Cry j 1 | 35,17 | Cup a 1 | 49,64 |
| Italy - CAAM 329 | F | 6 | Amb a 1 | 0,18 | Cry j 1 | 0,56 | Cup a 1 | 0,35 |
| Italy - CAAM 330 | M | 24 | Amb a 1 | 0,17 | Cry j 1 | 17,49 | Cup a 1 | 23,93 |
| Italy - CAAM 331 | F | 21 | Amb a 1 | 0,17 | Cry j 1 | 0,74 | Cup a 1 | 2,3 |
| Italy - CAAM 332 | M | 16 | Amb a 1 | 0,17 | Cry j 1 | 0,59 | Cup a 1 | 0,5 |
| Italy - CAAM 333 | M | 10 | Amb a 1 | 0,17 | Cry j 1 | 0 | Cup a 1 | 0 |
| Italy - CAAM 334 | F | 55 | Amb a 1 | 0,16 | Cry j 1 | 0 | Cup a 1 | 0 |
| Italy - CAAM 335 | F | 52 | Amb a 1 | 0,16 | Cry j 1 | 0,11 | Cup a 1 | 0 |
| Italy - CAAM 336 | F | 45 | Amb a 1 | 0,15 | Cry j 1 | 1,33 | Cup a 1 | 2,39 |
| Italy - CAAM 337 | M | 30 | Amb a 1 | 0,15 | Cry j 1 | 0 | Cup a 1 | 0 |
| Italy - CAAM 338 | F | 17 | Amb a 1 | 0,15 | Cry j 1 | 0,99 | Cup a 1 | 8,66 |
| Italy - CAAM 339 | F | 43 | Amb a 1 | 0,14 | Cry j 1 | 1,21 | Cup a 1 | 8,68 |
| Italy - CAAM 340 | F | 10 | Amb a 1 | 0,14 | Cry j 1 | 0 | Cup a 1 | 0 |
| Italy - CAAM 341 | M | 5 | Amb a 1 | 0,14 | Cry j 1 | 0,84 | Cup a 1 | 14,87 |
| Italy - CAAM 342 | M | 54 | Amb a 1 | 0,13 | Cry j 1 | 0,26 | Cup a 1 | 0,78 |
| Italy - CAAM 343 | F | 31 | Amb a 1 | 0,13 | Cry j 1 | 0,23 | Cup a 1 | 0,09 |
| Italy - CAAM 344 | F | 28 | Amb a 1 | 0,13 | Cry j 1 | 0 | Cup a 1 | 0 |
| Italy - CAAM 345 | F | 27 | Amb a 1 | 0,13 | Cry j 1 | 21,91 | Cup a 1 | 17,81 |
| Italy - CAAM 346 | M | 16 | Amb a 1 | 0,13 | Cry j 1 | 4,23 | Cup a 1 | 6,19 |
| Italy - CAAM 347 | F | 60 | Amb a 1 | 0,12 | Cry j 1 | 0 | Cup a 1 | 0 |
| Italy - CAAM 348 | F | 45 | Amb a 1 | 0,12 | Cry j 1 | 11,04 | Cup a 1 | 47,39 |
| Italy - CAAM 349 | M | 33 | Amb a 1 | 0,12 | Cry j 1 | 21,51 | Cup a 1 | 17,39 |
| Italy - CAAM 350 | M | 25 | Amb a 1 | 0,12 | Cry j 1 | 8,8 | Cup a 1 | 43,99 |
| Italy - CAAM 351 | F | 41 | Amb a 1 | 0,11 | Cry j 1 | 2,97 | Cup a 1 | 15,29 |
| Italy - CAAM 352 | F | 28 | Amb a 1 | 0,11 | Cry j 1 | 3,99 | Cup a 1 | 14,32 |
| Italy - CAAM 353 | F | 20 | Amb a 1 | 0,11 | Cry j 1 | 0,47 | Cup a 1 | 5,07 |
| Italy - CAAM 354 | F | 6 | Amb a 1 | 0,11 | Cry j 1 | 0,19 | Cup a 1 | 0,23 |
| Italy - CAAM 355 | M | 27 | Amb a 1 | 0,1 | Cry j 1 | 0 | Cup a 1 | 0,73 |
| Italy - CAAM 356 | F | 22 | Amb a 1 | 0,1 | Cry j 1 | 0,04 | Cup a 1 | 2,14 |
| Italy - CAAM 357 | F | 16 | Amb a 1 | 0,1 | Cry j 1 | 0,82 | Cup a 1 | 2,93 |
| Italy - CAAM 358 | M | 63 | Amb a 1 | 0,09 | Cry j 1 | 0 | Cup a 1 | 0 |
| Italy - CAAM 359 | F | 63 | Amb a 1 | 0,09 | Cry j 1 | 0,22 | Cup a 1 | 0,58 |
| Italy - CAAM 360 | F | 38 | Amb a 1 | 0,09 | Cry j 1 | 53,54 | Cup a 1 | 45,81 |
| Italy - CAAM 361 | M | 30 | Amb a 1 | 0,09 | Cry j 1 | 11,33 | Cup a 1 | 22,69 |
| Italy - CAAM 362 | F | 25 | Amb a 1 | 0,09 | Cry j 1 | 0,03 | Cup a 1 | 0 |
| Italy - CAAM 363 | F | 25 | Amb a 1 | 0,09 | Cry j 1 | 5,02 | Cup a 1 | 5,62 |
| Italy - CAAM 364 | F | 69 | Amb a 1 | 0,08 | Cry j 1 | 2,68 | Cup a 1 | 3,75 |
| Italy - CAAM 365 | F | 47 | Amb a 1 | 0,08 | Cry j 1 | 0 | Cup a 1 | 0 |
| Italy - CAAM 366 | F | 40 | Amb a 1 | 0,08 | Cry j 1 | 0 | Cup a 1 | 0 |
| Italy - CAAM 367 | F | 37 | Amb a 1 | 0,08 | Cry j 1 | 8,27 | Cup a 1 | 10,82 |
| Italy - CAAM 368 | M | 35 | Amb a 1 | 0,08 | Cry j 1 | 0,09 | Cup a 1 | 7,22 |
| Italy - CAAM 369 | M | 11 | Amb a 1 | 0,08 | Cry j 1 | 0 | Cup a 1 | 0 |
| Italy - CAAM 370 | F | 4 | Amb a 1 | 0,08 | Cry j 1 | 0 | Cup a 1 | 0 |
| Italy - CAAM 371 | F | 43 | Amb a 1 | 0,07 | Cry j 1 | 0,13 | Cup a 1 | 0 |
| Italy - CAAM 372 | F | 39 | Amb a 1 | 0,07 | Cry j 1 | 0,13 | Cup a 1 | 3,52 |
| Italy - CAAM 373 | M | 34 | Amb a 1 | 0,07 | Cry j 1 | 37,9 | Cup a 1 | 42,62 |
| Italy - CAAM 374 | M | 31 | Amb a 1 | 0,07 | Cry j 1 | 1,26 | Cup a 1 | 3,69 |
| Italy - CAAM 375 | F | 27 | Amb a 1 | 0,07 | Cry j 1 | 5,31 | Cup a 1 | 12,57 |
| Italy - CAAM 376 | M | 23 | Amb a 1 | 0,07 | Cry j 1 | 0,9 | Cup a 1 | 11,95 |
| Italy - CAAM 377 | M | 17 | Amb a 1 | 0,07 | Cry j 1 | 0,1 | Cup a 1 | 0,46 |
| Italy - CAAM 378 | M | 13 | Amb a 1 | 0,07 | Cry j 1 | 5,57 | Cup a 1 | 11,96 |
| Italy - CAAM 379 | M | 5 | Amb a 1 | 0,07 | Cry j 1 | 0 | Cup a 1 | 0 |
| Italy - CAAM 380 | M | 67 | Amb a 1 | 0,06 | Cry j 1 | 0,06 | Cup a 1 | 2,77 |
| Italy - CAAM 381 | F | 52 | Amb a 1 | 0,06 | Cry j 1 | 1,93 | Cup a 1 | 2,59 |
| Italy - CAAM 382 | M | 50 | Amb a 1 | 0,06 | Cry j 1 | 3,29 | Cup a 1 | 8,93 |
| Italy - CAAM 383 | M | 36 | Amb a 1 | 0,06 | Cry j 1 | 0,05 | Cup a 1 | 0 |
| Italy - CAAM 384 | F | 27 | Amb a 1 | 0,06 | Cry j 1 | 0 | Cup a 1 | 0 |
| Italy - CAAM 385 | F | 24 | Amb a 1 | 0,06 | Cry j 1 | 17,12 | Cup a 1 | 18,62 |
| Italy - CAAM 386 | M | 14 | Amb a 1 | 0,06 | Cry j 1 | 0,06 | Cup a 1 | 1,02 |
| Italy - CAAM 387 | F | 58 | Amb a 1 | 0,05 | Cry j 1 | 0 | Cup a 1 | 0 |
| Italy - CAAM 388 | F | 40 | Amb a 1 | 0,05 | Cry j 1 | 10,92 | Cup a 1 | 20,35 |
| Italy - CAAM 389 | F | 38 | Amb a 1 | 0,05 | Cry j 1 | 0 | Cup a 1 | 0 |
| Italy - CAAM 390 | F | 36 | Amb a 1 | 0,05 | Cry j 1 | 0,14 | Cup a 1 | 0 |
| Italy - CAAM 391 | F | 56 | Amb a 1 | 0,04 | Cry j 1 | 0,47 | Cup a 1 | 6,5 |
| Italy - CAAM 392 | F | 28 | Amb a 1 | 0,04 | Cry j 1 | 2,18 | Cup a 1 | 3,96 |
| Italy - CAAM 393 | M | 25 | Amb a 1 | 0,04 | Cry j 1 | 9,01 | Cup a 1 | 28,33 |
| Italy - CAAM 394 | F | 18 | Amb a 1 | 0,04 | Cry j 1 | 0 | Cup a 1 | 0 |
| Italy - CAAM 395 | M | 18 | Amb a 1 | 0,04 | Cry j 1 | 0,05 | Cup a 1 | 0,03 |
| Italy - CAAM 396 | M | 4 | Amb a 1 | 0,04 | Cry j 1 | 0,05 | Cup a 1 | 0,85 |
| Italy - CAAM 397 | M | 4 | Amb a 1 | 0,04 | Cry j 1 | 3,25 | Cup a 1 | 9,21 |
| Italy - CAAM 398 | F | 52 | Amb a 1 | 0,03 | Cry j 1 | 0,03 | Cup a 1 | 0,04 |
| Italy - CAAM 399 | F | 46 | Amb a 1 | 0,03 | Cry j 1 | 0,24 | Cup a 1 | 0,45 |
| Italy - CAAM 400 | F | 29 | Amb a 1 | 0,03 | Cry j 1 | 0 | Cup a 1 | 0 |
| Italy - CAAM 401 | F | 27 | Amb a 1 | 0,03 | Cry j 1 | 0 | Cup a 1 | 0 |
| Italy - CAAM 402 | F | 15 | Amb a 1 | 0,03 | Cry j 1 | 0 | Cup a 1 | 0 |
| Italy - CAAM 403 | F | 11 | Amb a 1 | 0,03 | Cry j 1 | 0 | Cup a 1 | 0,18 |
| Italy - CAAM 404 | M | 9 | Amb a 1 | 0,03 | Cry j 1 | 37,85 | Cup a 1 | 47,47 |
| Italy - CAAM 405 | F | 8 | Amb a 1 | 0,03 | Cry j 1 | 0 | Cup a 1 | 0 |
| Italy - CAAM 406 | M | 8 | Amb a 1 | 0,03 | Cry j 1 | 0 | Cup a 1 | 0 |
| Italy - CAAM 407 | F | 3 | Amb a 1 | 0,03 | Cry j 1 | 0 | Cup a 1 | 0 |
| Italy - CAAM 408 | F | 44 | Amb a 1 | 0,02 | Cry j 1 | 0,12 | Cup a 1 | 0,21 |
| Italy - CAAM 409 | F | 31 | Amb a 1 | 0,02 | Cry j 1 | 0 | Cup a 1 | 0 |
| Italy - CAAM 410 | F | 27 | Amb a 1 | 0,02 | Cry j 1 | 26,68 | Cup a 1 | 37,75 |
| Italy - CAAM 411 | F | 26 | Amb a 1 | 0,02 | Cry j 1 | 2,57 | Cup a 1 | 11,01 |
| Italy - CAAM 412 | F | 13 | Amb a 1 | 0,02 | Cry j 1 | 0,04 | Cup a 1 | 1,47 |
| Italy - CAAM 413 | M | 97 | Amb a 1 | 0 | Cry j 1 | 0,03 | Cup a 1 | 0 |
| Italy - CAAM 414 | M | 97 | Amb a 1 | 0 | Cry j 1 | 0,08 | Cup a 1 | 0,71 |
| Italy - CAAM 415 | F | 95 | Amb a 1 | 0 | Cry j 1 | 8,15 | Cup a 1 | 0 |
| Italy - CAAM 416 | F | 94 | Amb a 1 | 0 | Cry j 1 | 0,07 | Cup a 1 | 0,04 |
| Italy - CAAM 417 | F | 89 | Amb a 1 | 0 | Cry j 1 | 0,2 | Cup a 1 | 1,18 |
| Italy - CAAM 418 | M | 88 | Amb a 1 | 0 | Cry j 1 | 10,76 | Cup a 1 | 14,09 |
| Italy - CAAM 419 | F | 87 | Amb a 1 | 0 | Cry j 1 | 0,92 | Cup a 1 | 13,24 |
| Italy - CAAM 420 | M | 86 | Amb a 1 | 0 | Cry j 1 | 0,17 | Cup a 1 | 0,57 |
| Italy - CAAM 421 | M | 85 | Amb a 1 | 0 | Cry j 1 | 0,58 | Cup a 1 | 8,44 |
| Italy - CAAM 422 | M | 84 | Amb a 1 | 0 | Cry j 1 | 0 | Cup a 1 | 0,03 |
| Italy - CAAM 423 | F | 84 | Amb a 1 | 0 | Cry j 1 | 0,06 | Cup a 1 | 0,07 |
| Italy - CAAM 424 | M | 84 | Amb a 1 | 0 | Cry j 1 | 1,23 | Cup a 1 | 4,92 |
| Italy - CAAM 425 | F | 84 | Amb a 1 | 0 | Cry j 1 | 0 | Cup a 1 | 0,05 |
| Italy - CAAM 426 | M | 83 | Amb a 1 | 0 | Cry j 1 | 3,77 | Cup a 1 | 4,31 |
| Italy - CAAM 427 | M | 83 | Amb a 1 | 0 | Cry j 1 | 0,1 | Cup a 1 | 0 |
| Italy - CAAM 428 | M | 82 | Amb a 1 | 0 | Cry j 1 | 0,38 | Cup a 1 | 2,07 |
| Italy - CAAM 429 | M | 82 | Amb a 1 | 0 | Cry j 1 | 0 | Cup a 1 | 0,17 |
| Italy - CAAM 430 | M | 82 | Amb a 1 | 0 | Cry j 1 | 0,04 | Cup a 1 | 0 |
| Italy - CAAM 431 | F | 82 | Amb a 1 | 0 | Cry j 1 | 0 | Cup a 1 | 1,1 |
| Italy - CAAM 432 | M | 81 | Amb a 1 | 0 | Cry j 1 | 0,23 | Cup a 1 | 0,15 |
| Italy - CAAM 433 | F | 81 | Amb a 1 | 0 | Cry j 1 | 0 | Cup a 1 | 0,15 |
| Italy - CAAM 434 | F | 81 | Amb a 1 | 0 | Cry j 1 | 1,07 | Cup a 1 | 5,19 |
| Italy - CAAM 435 | F | 80 | Amb a 1 | 0 | Cry j 1 | 0,11 | Cup a 1 | 0,42 |
| Italy - CAAM 436 | F | 80 | Amb a 1 | 0 | Cry j 1 | 0,07 | Cup a 1 | 1,36 |
| Italy - CAAM 437 | M | 80 | Amb a 1 | 0 | Cry j 1 | 0,44 | Cup a 1 | 0,59 |
| Italy - CAAM 438 | F | 80 | Amb a 1 | 0 | Cry j 1 | 1,9 | Cup a 1 | 7,81 |
| Italy - CAAM 439 | M | 80 | Amb a 1 | 0 | Cry j 1 | 0 | Cup a 1 | 5,35 |
| Italy - CAAM 440 | F | 79 | Amb a 1 | 0 | Cry j 1 | 1,16 | Cup a 1 | 5,54 |
| Italy - CAAM 441 | M | 79 | Amb a 1 | 0 | Cry j 1 | 8,27 | Cup a 1 | 10,78 |
| Italy - CAAM 442 | F | 79 | Amb a 1 | 0 | Cry j 1 | 1,15 | Cup a 1 | 2,8 |
| Italy - CAAM 443 | M | 79 | Amb a 1 | 0 | Cry j 1 | 0,05 | Cup a 1 | 0,05 |
| Italy - CAAM 444 | M | 79 | Amb a 1 | 0 | Cry j 1 | 4,4 | Cup a 1 | 10,15 |
| Italy - CAAM 445 | F | 79 | Amb a 1 | 0 | Cry j 1 | 0,26 | Cup a 1 | 1,21 |
| Italy - CAAM 446 | F | 79 | Amb a 1 | 0 | Cry j 1 | 0,04 | Cup a 1 | 0,55 |
| Italy - CAAM 447 | F | 79 | Amb a 1 | 0 | Cry j 1 | 3,23 | Cup a 1 | 13,21 |
| Italy - CAAM 448 | M | 79 | Amb a 1 | 0 | Cry j 1 | 0,26 | Cup a 1 | 4,16 |
| Italy - CAAM 449 | M | 79 | Amb a 1 | 0 | Cry j 1 | 6,94 | Cup a 1 | 30,28 |
| Italy - CAAM 450 | M | 78 | Amb a 1 | 0 | Cry j 1 | 0 | Cup a 1 | 0,06 |
| Italy - CAAM 451 | F | 78 | Amb a 1 | 0 | Cry j 1 | 0 | Cup a 1 | 1,26 |
| Italy - CAAM 452 | M | 78 | Amb a 1 | 0 | Cry j 1 | 3,48 | Cup a 1 | 8,89 |
| Italy - CAAM 453 | F | 78 | Amb a 1 | 0 | Cry j 1 | 0,22 | Cup a 1 | 1,64 |
| Italy - CAAM 454 | M | 78 | Amb a 1 | 0 | Cry j 1 | 1,01 | Cup a 1 | 6,62 |
| Italy - CAAM 455 | M | 78 | Amb a 1 | 0 | Cry j 1 | 1,22 | Cup a 1 | 1,33 |
| Italy - CAAM 456 | F | 78 | Amb a 1 | 0 | Cry j 1 | 0 | Cup a 1 | 1,1 |
| Italy - CAAM 457 | M | 78 | Amb a 1 | 0 | Cry j 1 | 2,35 | Cup a 1 | 4,67 |
| Italy - CAAM 458 | F | 78 | Amb a 1 | 0 | Cry j 1 | 0,17 | Cup a 1 | 1,43 |
| Italy - CAAM 459 | M | 78 | Amb a 1 | 0 | Cry j 1 | 0 | Cup a 1 | 1,88 |
| Italy - CAAM 460 | M | 78 | Amb a 1 | 0 | Cry j 1 | 0,1 | Cup a 1 | 11,22 |
| Italy - CAAM 461 | F | 77 | Amb a 1 | 0 | Cry j 1 | 8,1 | Cup a 1 | 9,6 |
| Italy - CAAM 462 | M | 77 | Amb a 1 | 0 | Cry j 1 | 0,19 | Cup a 1 | 0,85 |
| Italy - CAAM 463 | M | 77 | Amb a 1 | 0 | Cry j 1 | 0,26 | Cup a 1 | 1,17 |
| Italy - CAAM 464 | F | 77 | Amb a 1 | 0 | Cry j 1 | 0,42 | Cup a 1 | 0,53 |
| Italy - CAAM 465 | M | 76 | Amb a 1 | 0 | Cry j 1 | 0 | Cup a 1 | 11,96 |
| Italy - CAAM 466 | F | 76 | Amb a 1 | 0 | Cry j 1 | 0,38 | Cup a 1 | 6,5 |
| Italy - CAAM 467 | F | 76 | Amb a 1 | 0 | Cry j 1 | 0,07 | Cup a 1 | 0 |
| Italy - CAAM 468 | F | 76 | Amb a 1 | 0 | Cry j 1 | 0,11 | Cup a 1 | 0,26 |
| Italy - CAAM 469 | F | 76 | Amb a 1 | 0 | Cry j 1 | 0,15 | Cup a 1 | 0,36 |
| Italy - CAAM 470 | F | 76 | Amb a 1 | 0 | Cry j 1 | 0,7 | Cup a 1 | 6,95 |
| Italy - CAAM 471 | F | 76 | Amb a 1 | 0 | Cry j 1 | 0 | Cup a 1 | 0,13 |
| Italy - CAAM 472 | F | 76 | Amb a 1 | 0 | Cry j 1 | 0,13 | Cup a 1 | 0,69 |
| Italy - CAAM 473 | F | 76 | Amb a 1 | 0 | Cry j 1 | 1,02 | Cup a 1 | 25,65 |
| Italy - CAAM 474 | F | 76 | Amb a 1 | 0 | Cry j 1 | 0 | Cup a 1 | 13 |
| Italy - CAAM 475 | F | 76 | Amb a 1 | 0 | Cry j 1 | 0 | Cup a 1 | 9,99 |
| Italy - CAAM 476 | M | 75 | Amb a 1 | 0 | Cry j 1 | 0 | Cup a 1 | 0,34 |
| Italy - CAAM 477 | F | 75 | Amb a 1 | 0 | Cry j 1 | 1,81 | Cup a 1 | 2,06 |
| Italy - CAAM 478 | F | 75 | Amb a 1 | 0 | Cry j 1 | 3,64 | Cup a 1 | 17,82 |
| Italy - CAAM 479 | M | 75 | Amb a 1 | 0 | Cry j 1 | 0,43 | Cup a 1 | 3,41 |
| Italy - CAAM 480 | M | 75 | Amb a 1 | 0 | Cry j 1 | 1,75 | Cup a 1 | 3,48 |
| Italy - CAAM 481 | M | 75 | Amb a 1 | 0 | Cry j 1 | 0,91 | Cup a 1 | 2,98 |
| Italy - CAAM 482 | F | 75 | Amb a 1 | 0 | Cry j 1 | 11,25 | Cup a 1 | 16,1 |
| Italy - CAAM 483 | M | 75 | Amb a 1 | 0 | Cry j 1 | 1,28 | Cup a 1 | 1,66 |
| Italy - CAAM 484 | M | 75 | Amb a 1 | 0 | Cry j 1 | 0 | Cup a 1 | 3,5 |
| Italy - CAAM 485 | F | 75 | Amb a 1 | 0 | Cry j 1 | 0 | Cup a 1 | 4,38 |
| Italy - CAAM 486 | M | 74 | Amb a 1 | 0 | Cry j 1 | 0,37 | Cup a 1 | 1,09 |
| Italy - CAAM 487 | F | 74 | Amb a 1 | 0 | Cry j 1 | 0 | Cup a 1 | 1,33 |
| Italy - CAAM 488 | F | 74 | Amb a 1 | 0 | Cry j 1 | 0,28 | Cup a 1 | 0,12 |
| Italy - CAAM 489 | F | 74 | Amb a 1 | 0 | Cry j 1 | 1,27 | Cup a 1 | 0,39 |
| Italy - CAAM 490 | M | 74 | Amb a 1 | 0 | Cry j 1 | 1,92 | Cup a 1 | 2,18 |
| Italy - CAAM 491 | F | 74 | Amb a 1 | 0 | Cry j 1 | 2,06 | Cup a 1 | 0 |
| Italy - CAAM 492 | M | 74 | Amb a 1 | 0 | Cry j 1 | 0,32 | Cup a 1 | 0,84 |
| Italy - CAAM 493 | F | 74 | Amb a 1 | 0 | Cry j 1 | 4,77 | Cup a 1 | 30,69 |
| Italy - CAAM 494 | F | 74 | Amb a 1 | 0 | Cry j 1 | 5,33 | Cup a 1 | 10,77 |
| Italy - CAAM 495 | M | 74 | Amb a 1 | 0 | Cry j 1 | 0,04 | Cup a 1 | 0,45 |
| Italy - CAAM 496 | M | 74 | Amb a 1 | 0 | Cry j 1 | 0 | Cup a 1 | 0,29 |
| Italy - CAAM 497 | M | 74 | Amb a 1 | 0 | Cry j 1 | 0,09 | Cup a 1 | 0,19 |
| Italy - CAAM 498 | F | 74 | Amb a 1 | 0 | Cry j 1 | 2,61 | Cup a 1 | 3,44 |
| Italy - CAAM 499 | M | 74 | Amb a 1 | 0 | Cry j 1 | 0 | Cup a 1 | 0,45 |
| Italy - CAAM 500 | M | 74 | Amb a 1 | 0 | Cry j 1 | 0,16 | Cup a 1 | 0,13 |
| Italy - CAAM 501 | F | 74 | Amb a 1 | 0 | Cry j 1 | 0,81 | Cup a 1 | 3,23 |
| Italy - CAAM 502 | F | 74 | Amb a 1 | 0 | Cry j 1 | 0 | Cup a 1 | 0,15 |
| Italy - CAAM 503 | M | 74 | Amb a 1 | 0 | Cry j 1 | 0 | Cup a 1 | 27 |
| Italy - CAAM 504 | M | 74 | Amb a 1 | 0 | Cry j 1 | 2,59 | Cup a 1 | 9,78 |
| Italy - CAAM 505 | F | 74 | Amb a 1 | 0 | Cry j 1 | 0,12 | Cup a 1 | 2,27 |
| Italy - CAAM 506 | M | 74 | Amb a 1 | 0 | Cry j 1 | 2,74 | Cup a 1 | 14,09 |
| Italy - CAAM 507 | F | 74 | Amb a 1 | 0 | Cry j 1 | 0 | Cup a 1 | 1,89 |
| Italy - CAAM 508 | M | 74 | Amb a 1 | 0 | Cry j 1 | 1,17 | Cup a 1 | 15,05 |
| Italy - CAAM 509 | F | 74 | Amb a 1 | 0 | Cry j 1 | 0 | Cup a 1 | 1,64 |
| Italy - CAAM 510 | M | 74 | Amb a 1 | 0 | Cry j 1 | 0,63 | Cup a 1 | 4,94 |
| Italy - CAAM 511 | M | 73 | Amb a 1 | 0 | Cry j 1 | 0,14 | Cup a 1 | 0 |
| Italy - CAAM 512 | F | 73 | Amb a 1 | 0 | Cry j 1 | 0,38 | Cup a 1 | 5,54 |
| Italy - CAAM 513 | F | 73 | Amb a 1 | 0 | Cry j 1 | 1,81 | Cup a 1 | 12,99 |
| Italy - CAAM 514 | M | 73 | Amb a 1 | 0 | Cry j 1 | 0 | Cup a 1 | 0,07 |
| Italy - CAAM 515 | M | 73 | Amb a 1 | 0 | Cry j 1 | 0,08 | Cup a 1 | 0,4 |
| Italy - CAAM 516 | F | 73 | Amb a 1 | 0 | Cry j 1 | 0,05 | Cup a 1 | 0,11 |
| Italy - CAAM 517 | F | 73 | Amb a 1 | 0 | Cry j 1 | 0,05 | Cup a 1 | 0,16 |
| Italy - CAAM 518 | F | 73 | Amb a 1 | 0 | Cry j 1 | 0,2 | Cup a 1 | 0,87 |
| Italy - CAAM 519 | M | 73 | Amb a 1 | 0 | Cry j 1 | 1,69 | Cup a 1 | 2,78 |
| Italy - CAAM 520 | F | 73 | Amb a 1 | 0 | Cry j 1 | 0,06 | Cup a 1 | 0,31 |
| Italy - CAAM 521 | M | 73 | Amb a 1 | 0 | Cry j 1 | 2,3 | Cup a 1 | 4,49 |
| Italy - CAAM 522 | F | 73 | Amb a 1 | 0 | Cry j 1 | 5,12 | Cup a 1 | 31,6 |
| Italy - CAAM 523 | M | 73 | Amb a 1 | 0 | Cry j 1 | 0,34 | Cup a 1 | 3,97 |
| Italy - CAAM 524 | F | 73 | Amb a 1 | 0 | Cry j 1 | 7,71 | Cup a 1 | 19,9 |
| Italy - CAAM 525 | F | 73 | Amb a 1 | 0 | Cry j 1 | 0,79 | Cup a 1 | 11,46 |
| Italy - CAAM 526 | M | 73 | Amb a 1 | 0 | Cry j 1 | 8,74 | Cup a 1 | 36,97 |
| Italy - CAAM 527 | F | 73 | Amb a 1 | 0 | Cry j 1 | 0,11 | Cup a 1 | 0,13 |
| Italy - CAAM 528 | M | 73 | Amb a 1 | 0 | Cry j 1 | 0,54 | Cup a 1 | 8,77 |
| Italy - CAAM 529 | M | 73 | Amb a 1 | 0 | Cry j 1 | 0,12 | Cup a 1 | 1,9 |
| Italy - CAAM 530 | M | 72 | Amb a 1 | 0 | Cry j 1 | 0,14 | Cup a 1 | 0,13 |
| Italy - CAAM 531 | F | 72 | Amb a 1 | 0 | Cry j 1 | 0 | Cup a 1 | 1,32 |
| Italy - CAAM 532 | M | 72 | Amb a 1 | 0 | Cry j 1 | 0 | Cup a 1 | 0,86 |
| Italy - CAAM 533 | M | 72 | Amb a 1 | 0 | Cry j 1 | 0,52 | Cup a 1 | 3,42 |
| Italy - CAAM 534 | F | 72 | Amb a 1 | 0 | Cry j 1 | 5,12 | Cup a 1 | 0 |
| Italy - CAAM 535 | M | 72 | Amb a 1 | 0 | Cry j 1 | 0,23 | Cup a 1 | 0,96 |
| Italy - CAAM 536 | M | 72 | Amb a 1 | 0 | Cry j 1 | 4,03 | Cup a 1 | 0 |
| Italy - CAAM 537 | M | 72 | Amb a 1 | 0 | Cry j 1 | 0,58 | Cup a 1 | 0,93 |
| Italy - CAAM 538 | M | 72 | Amb a 1 | 0 | Cry j 1 | 0 | Cup a 1 | 0,15 |
| Italy - CAAM 539 | F | 72 | Amb a 1 | 0 | Cry j 1 | 0 | Cup a 1 | 0,2 |
| Italy - CAAM 540 | F | 72 | Amb a 1 | 0 | Cry j 1 | 7,37 | Cup a 1 | 16,75 |
| Italy - CAAM 541 | M | 72 | Amb a 1 | 0 | Cry j 1 | 1,66 | Cup a 1 | 11,78 |
| Italy - CAAM 542 | F | 72 | Amb a 1 | 0 | Cry j 1 | 0,95 | Cup a 1 | 3,74 |
| Italy - CAAM 543 | F | 72 | Amb a 1 | 0 | Cry j 1 | 0 | Cup a 1 | 0,14 |
| Italy - CAAM 544 | M | 72 | Amb a 1 | 0 | Cry j 1 | 7,77 | Cup a 1 | 42,93 |
| Italy - CAAM 545 | F | 72 | Amb a 1 | 0 | Cry j 1 | 0 | Cup a 1 | 0,09 |
| Italy - CAAM 546 | M | 72 | Amb a 1 | 0 | Cry j 1 | 0,82 | Cup a 1 | 4,61 |
| Italy - CAAM 547 | M | 72 | Amb a 1 | 0 | Cry j 1 | 0,16 | Cup a 1 | 0,27 |
| Italy - CAAM 548 | F | 72 | Amb a 1 | 0 | Cry j 1 | 1,75 | Cup a 1 | 3,42 |
| Italy - CAAM 549 | F | 72 | Amb a 1 | 0 | Cry j 1 | 0,05 | Cup a 1 | 0,16 |
| Italy - CAAM 550 | F | 72 | Amb a 1 | 0 | Cry j 1 | 0 | Cup a 1 | 0,32 |
| Italy - CAAM 551 | M | 72 | Amb a 1 | 0 | Cry j 1 | 0 | Cup a 1 | 0,32 |
| Italy - CAAM 552 | F | 72 | Amb a 1 | 0 | Cry j 1 | 0,13 | Cup a 1 | 3,29 |
| Italy - CAAM 553 | M | 72 | Amb a 1 | 0 | Cry j 1 | 0 | Cup a 1 | 14,72 |
| Italy - CAAM 554 | M | 72 | Amb a 1 | 0 | Cry j 1 | 0 | Cup a 1 | 0,16 |
| Italy - CAAM 555 | F | 72 | Amb a 1 | 0 | Cry j 1 | 0,17 | Cup a 1 | 1,53 |
| Italy - CAAM 556 | F | 72 | Amb a 1 | 0 | Cry j 1 | 0 | Cup a 1 | 0,36 |
| Italy - CAAM 557 | F | 72 | Amb a 1 | 0 | Cry j 1 | 0,17 | Cup a 1 | 2,8 |
| Italy - CAAM 558 | F | 72 | Amb a 1 | 0 | Cry j 1 | 0 | Cup a 1 | 4,03 |
| Italy - CAAM 559 | F | 72 | Amb a 1 | 0 | Cry j 1 | 0 | Cup a 1 | 1,51 |
| Italy - CAAM 560 | F | 72 | Amb a 1 | 0 | Cry j 1 | 4,16 | Cup a 1 | 9,98 |
| Italy - CAAM 561 | F | 71 | Amb a 1 | 0 | Cry j 1 | 0 | Cup a 1 | 0,27 |
| Italy - CAAM 562 | F | 71 | Amb a 1 | 0 | Cry j 1 | 0 | Cup a 1 | 0,09 |
| Italy - CAAM 563 | F | 71 | Amb a 1 | 0 | Cry j 1 | 0,15 | Cup a 1 | 0,67 |
| Italy - CAAM 564 | F | 71 | Amb a 1 | 0 | Cry j 1 | 0,22 | Cup a 1 | 2,3 |
| Italy - CAAM 565 | M | 71 | Amb a 1 | 0 | Cry j 1 | 6,58 | Cup a 1 | 0 |
| Italy - CAAM 566 | F | 71 | Amb a 1 | 0 | Cry j 1 | 1,48 | Cup a 1 | 2,85 |
| Italy - CAAM 567 | M | 71 | Amb a 1 | 0 | Cry j 1 | 1,32 | Cup a 1 | 6,41 |
| Italy - CAAM 568 | F | 71 | Amb a 1 | 0 | Cry j 1 | 0 | Cup a 1 | 1,38 |
| Italy - CAAM 569 | M | 71 | Amb a 1 | 0 | Cry j 1 | 0,06 | Cup a 1 | 0 |
| Italy - CAAM 570 | F | 71 | Amb a 1 | 0 | Cry j 1 | 4,41 | Cup a 1 | 12,89 |
| Italy - CAAM 571 | M | 71 | Amb a 1 | 0 | Cry j 1 | 0,58 | Cup a 1 | 1,52 |
| Italy - CAAM 572 | M | 71 | Amb a 1 | 0 | Cry j 1 | 2,74 | Cup a 1 | 15,08 |
| Italy - CAAM 573 | M | 71 | Amb a 1 | 0 | Cry j 1 | 4,31 | Cup a 1 | 24,07 |
| Italy - CAAM 574 | F | 71 | Amb a 1 | 0 | Cry j 1 | 14,41 | Cup a 1 | 20,56 |
| Italy - CAAM 575 | F | 71 | Amb a 1 | 0 | Cry j 1 | 0 | Cup a 1 | 0,72 |
| Italy - CAAM 576 | M | 71 | Amb a 1 | 0 | Cry j 1 | 0,26 | Cup a 1 | 0,72 |
| Italy - CAAM 577 | M | 71 | Amb a 1 | 0 | Cry j 1 | 0,14 | Cup a 1 | 0,87 |
| Italy - CAAM 578 | M | 71 | Amb a 1 | 0 | Cry j 1 | 0 | Cup a 1 | 0,23 |
| Italy - CAAM 579 | M | 71 | Amb a 1 | 0 | Cry j 1 | 0 | Cup a 1 | 0,16 |
| Italy - CAAM 580 | F | 71 | Amb a 1 | 0 | Cry j 1 | 0,17 | Cup a 1 | 1,7 |
| Italy - CAAM 581 | F | 71 | Amb a 1 | 0 | Cry j 1 | 0 | Cup a 1 | 0,15 |
| Italy - CAAM 582 | F | 71 | Amb a 1 | 0 | Cry j 1 | 1,48 | Cup a 1 | 8,42 |
| Italy - CAAM 583 | M | 71 | Amb a 1 | 0 | Cry j 1 | 0 | Cup a 1 | 6,6 |
| Italy - CAAM 584 | M | 70 | Amb a 1 | 0 | Cry j 1 | 0 | Cup a 1 | 3,24 |
| Italy - CAAM 585 | M | 70 | Amb a 1 | 0 | Cry j 1 | 0 | Cup a 1 | 0,06 |
| Italy - CAAM 586 | F | 70 | Amb a 1 | 0 | Cry j 1 | 0 | Cup a 1 | 0,6 |
| Italy - CAAM 587 | M | 70 | Amb a 1 | 0 | Cry j 1 | 0,72 | Cup a 1 | 2,13 |
| Italy - CAAM 588 | M | 70 | Amb a 1 | 0 | Cry j 1 | 10,18 | Cup a 1 | 0 |
| Italy - CAAM 589 | F | 70 | Amb a 1 | 0 | Cry j 1 | 1,04 | Cup a 1 | 1,34 |
| Italy - CAAM 590 | F | 70 | Amb a 1 | 0 | Cry j 1 | 1,92 | Cup a 1 | 3,49 |
| Italy - CAAM 591 | F | 70 | Amb a 1 | 0 | Cry j 1 | 0,09 | Cup a 1 | 0,07 |
| Italy - CAAM 592 | M | 70 | Amb a 1 | 0 | Cry j 1 | 1,06 | Cup a 1 | 5,51 |
| Italy - CAAM 593 | M | 70 | Amb a 1 | 0 | Cry j 1 | 1,51 | Cup a 1 | 8,56 |
| Italy - CAAM 594 | M | 70 | Amb a 1 | 0 | Cry j 1 | 0 | Cup a 1 | 0,24 |
| Italy - CAAM 595 | M | 70 | Amb a 1 | 0 | Cry j 1 | 0,11 | Cup a 1 | 0 |
| Italy - CAAM 596 | F | 70 | Amb a 1 | 0 | Cry j 1 | 0 | Cup a 1 | 0,11 |
| Italy - CAAM 597 | F | 70 | Amb a 1 | 0 | Cry j 1 | 0,05 | Cup a 1 | 0,85 |
| Italy - CAAM 598 | F | 70 | Amb a 1 | 0 | Cry j 1 | 0,03 | Cup a 1 | 2,6 |
| Italy - CAAM 599 | F | 70 | Amb a 1 | 0 | Cry j 1 | 7,95 | Cup a 1 | 26,61 |
| Italy - CAAM 600 | F | 70 | Amb a 1 | 0 | Cry j 1 | 0 | Cup a 1 | 1,64 |
| Italy - CAAM 601 | M | 70 | Amb a 1 | 0 | Cry j 1 | 1,55 | Cup a 1 | 11,93 |
| Italy - CAAM 602 | F | 70 | Amb a 1 | 0 | Cry j 1 | 0 | Cup a 1 | 0,52 |
| Italy - CAAM 603 | M | 70 | Amb a 1 | 0 | Cry j 1 | 0,12 | Cup a 1 | 3,12 |
| Italy - CAAM 604 | F | 69 | Amb a 1 | 0 | Cry j 1 | 0 | Cup a 1 | 3,15 |
| Italy - CAAM 605 | F | 69 | Amb a 1 | 0 | Cry j 1 | 0 | Cup a 1 | 5,55 |
| Italy - CAAM 606 | M | 69 | Amb a 1 | 0 | Cry j 1 | 0,85 | Cup a 1 | 2,43 |
| Italy - CAAM 607 | M | 69 | Amb a 1 | 0 | Cry j 1 | 3,1 | Cup a 1 | 32 |
| Italy - CAAM 608 | M | 69 | Amb a 1 | 0 | Cry j 1 | 0,2 | Cup a 1 | 3,17 |
| Italy - CAAM 609 | M | 69 | Amb a 1 | 0 | Cry j 1 | 2,1 | Cup a 1 | 5,53 |
| Italy - CAAM 610 | M | 69 | Amb a 1 | 0 | Cry j 1 | 0,23 | Cup a 1 | 0,71 |
| Italy - CAAM 611 | F | 69 | Amb a 1 | 0 | Cry j 1 | 0,77 | Cup a 1 | 1,02 |
| Italy - CAAM 612 | M | 69 | Amb a 1 | 0 | Cry j 1 | 1,73 | Cup a 1 | 0 |
| Italy - CAAM 613 | F | 69 | Amb a 1 | 0 | Cry j 1 | 0,88 | Cup a 1 | 0 |
| Italy - CAAM 614 | M | 69 | Amb a 1 | 0 | Cry j 1 | 2,41 | Cup a 1 | 0 |
| Italy - CAAM 615 | M | 69 | Amb a 1 | 0 | Cry j 1 | 0,18 | Cup a 1 | 0,12 |
| Italy - CAAM 616 | M | 69 | Amb a 1 | 0 | Cry j 1 | 0,26 | Cup a 1 | 0,29 |
| Italy - CAAM 617 | M | 69 | Amb a 1 | 0 | Cry j 1 | 0,12 | Cup a 1 | 0,35 |
| Italy - CAAM 618 | F | 69 | Amb a 1 | 0 | Cry j 1 | 0,34 | Cup a 1 | 17,49 |
| Italy - CAAM 619 | F | 69 | Amb a 1 | 0 | Cry j 1 | 0,06 | Cup a 1 | 0,15 |
| Italy - CAAM 620 | F | 69 | Amb a 1 | 0 | Cry j 1 | 0,19 | Cup a 1 | 1,65 |
| Italy - CAAM 621 | F | 69 | Amb a 1 | 0 | Cry j 1 | 0,32 | Cup a 1 | 0,48 |
| Italy - CAAM 622 | F | 69 | Amb a 1 | 0 | Cry j 1 | 5,99 | Cup a 1 | 12,16 |
| Italy - CAAM 623 | M | 69 | Amb a 1 | 0 | Cry j 1 | 0,4 | Cup a 1 | 1,96 |
| Italy - CAAM 624 | M | 69 | Amb a 1 | 0 | Cry j 1 | 0,39 | Cup a 1 | 4,49 |
| Italy - CAAM 625 | F | 69 | Amb a 1 | 0 | Cry j 1 | 0,98 | Cup a 1 | 4,33 |
| Italy - CAAM 626 | F | 69 | Amb a 1 | 0 | Cry j 1 | 0,06 | Cup a 1 | 0,64 |
| Italy - CAAM 627 | M | 69 | Amb a 1 | 0 | Cry j 1 | 5,35 | Cup a 1 | 9,88 |
| Italy - CAAM 628 | F | 69 | Amb a 1 | 0 | Cry j 1 | 0 | Cup a 1 | 0,37 |
| Italy - CAAM 629 | F | 69 | Amb a 1 | 0 | Cry j 1 | 3,16 | Cup a 1 | 13,17 |
| Italy - CAAM 630 | M | 69 | Amb a 1 | 0 | Cry j 1 | 0,96 | Cup a 1 | 1,19 |
| Italy - CAAM 631 | M | 69 | Amb a 1 | 0 | Cry j 1 | 0 | Cup a 1 | 1,26 |
| Italy - CAAM 632 | F | 69 | Amb a 1 | 0 | Cry j 1 | 0,19 | Cup a 1 | 2,26 |
| Italy - CAAM 633 | M | 69 | Amb a 1 | 0 | Cry j 1 | 0 | Cup a 1 | 1 |
| Italy - CAAM 634 | M | 69 | Amb a 1 | 0 | Cry j 1 | 0,92 | Cup a 1 | 12,6 |
| Italy - CAAM 635 | F | 68 | Amb a 1 | 0 | Cry j 1 | 3,3 | Cup a 1 | 22 |
| Italy - CAAM 636 | F | 68 | Amb a 1 | 0 | Cry j 1 | 0,45 | Cup a 1 | 7,22 |
| Italy - CAAM 637 | F | 68 | Amb a 1 | 0 | Cry j 1 | 0,21 | Cup a 1 | 0 |
| Italy - CAAM 638 | F | 68 | Amb a 1 | 0 | Cry j 1 | 10,04 | Cup a 1 | 3,78 |
| Italy - CAAM 639 | F | 68 | Amb a 1 | 0 | Cry j 1 | 5,35 | Cup a 1 | 0 |
| Italy - CAAM 640 | M | 68 | Amb a 1 | 0 | Cry j 1 | 2,74 | Cup a 1 | 0,67 |
| Italy - CAAM 641 | F | 68 | Amb a 1 | 0 | Cry j 1 | 0 | Cup a 1 | 0,31 |
| Italy - CAAM 642 | F | 68 | Amb a 1 | 0 | Cry j 1 | 4,9 | Cup a 1 | 53 |
| Italy - CAAM 643 | M | 68 | Amb a 1 | 0 | Cry j 1 | 0 | Cup a 1 | 1,15 |
| Italy - CAAM 644 | M | 68 | Amb a 1 | 0 | Cry j 1 | 2,7 | Cup a 1 | 12,32 |
| Italy - CAAM 645 | F | 68 | Amb a 1 | 0 | Cry j 1 | 0,65 | Cup a 1 | 3,04 |
| Italy - CAAM 646 | M | 68 | Amb a 1 | 0 | Cry j 1 | 0 | Cup a 1 | 0,11 |
| Italy - CAAM 647 | M | 68 | Amb a 1 | 0 | Cry j 1 | 0,2 | Cup a 1 | 0 |
| Italy - CAAM 648 | M | 68 | Amb a 1 | 0 | Cry j 1 | 0,09 | Cup a 1 | 0 |
| Italy - CAAM 649 | F | 68 | Amb a 1 | 0 | Cry j 1 | 0,24 | Cup a 1 | 2,13 |
| Italy - CAAM 650 | F | 68 | Amb a 1 | 0 | Cry j 1 | 0,05 | Cup a 1 | 0,68 |
| Italy - CAAM 651 | M | 68 | Amb a 1 | 0 | Cry j 1 | 3,46 | Cup a 1 | 5,29 |
| Italy - CAAM 652 | M | 68 | Amb a 1 | 0 | Cry j 1 | 1,89 | Cup a 1 | 4,49 |
| Italy - CAAM 653 | M | 68 | Amb a 1 | 0 | Cry j 1 | 7,33 | Cup a 1 | 18,97 |
| Italy - CAAM 654 | M | 68 | Amb a 1 | 0 | Cry j 1 | 7,55 | Cup a 1 | 17,68 |
| Italy - CAAM 655 | F | 68 | Amb a 1 | 0 | Cry j 1 | 0,12 | Cup a 1 | 0,46 |
| Italy - CAAM 656 | M | 68 | Amb a 1 | 0 | Cry j 1 | 2,42 | Cup a 1 | 2,55 |
| Italy - CAAM 657 | M | 68 | Amb a 1 | 0 | Cry j 1 | 0,54 | Cup a 1 | 0,79 |
| Italy - CAAM 658 | F | 68 | Amb a 1 | 0 | Cry j 1 | 9 | Cup a 1 | 16,67 |
| Italy - CAAM 659 | F | 68 | Amb a 1 | 0 | Cry j 1 | 2,15 | Cup a 1 | 14,59 |
| Italy - CAAM 660 | F | 68 | Amb a 1 | 0 | Cry j 1 | 0 | Cup a 1 | 0,23 |
| Italy - CAAM 661 | M | 68 | Amb a 1 | 0 | Cry j 1 | 0,16 | Cup a 1 | 1,11 |
| Italy - CAAM 662 | M | 67 | Amb a 1 | 0 | Cry j 1 | 0,77 | Cup a 1 | 11,3 |
| Italy - CAAM 663 | F | 67 | Amb a 1 | 0 | Cry j 1 | 0 | Cup a 1 | 5,15 |
| Italy - CAAM 664 | F | 67 | Amb a 1 | 0 | Cry j 1 | 0 | Cup a 1 | 0,32 |
| Italy - CAAM 665 | F | 67 | Amb a 1 | 0 | Cry j 1 | 0,84 | Cup a 1 | 2,69 |
| Italy - CAAM 666 | F | 67 | Amb a 1 | 0 | Cry j 1 | 2,94 | Cup a 1 | 5,52 |
| Italy - CAAM 667 | F | 67 | Amb a 1 | 0 | Cry j 1 | 0 | Cup a 1 | 0,09 |
| Italy - CAAM 668 | F | 67 | Amb a 1 | 0 | Cry j 1 | 0,05 | Cup a 1 | 0,06 |
| Italy - CAAM 669 | F | 67 | Amb a 1 | 0 | Cry j 1 | 0,9 | Cup a 1 | 1,03 |
| Italy - CAAM 670 | M | 67 | Amb a 1 | 0 | Cry j 1 | 0 | Cup a 1 | 11,84 |
| Italy - CAAM 671 | M | 67 | Amb a 1 | 0 | Cry j 1 | 0,43 | Cup a 1 | 0,43 |
| Italy - CAAM 672 | M | 67 | Amb a 1 | 0 | Cry j 1 | 0,13 | Cup a 1 | 0,14 |
| Italy - CAAM 673 | F | 67 | Amb a 1 | 0 | Cry j 1 | 9,28 | Cup a 1 | 39,05 |
| Italy - CAAM 674 | F | 67 | Amb a 1 | 0 | Cry j 1 | 0,12 | Cup a 1 | 0,23 |
| Italy - CAAM 675 | F | 67 | Amb a 1 | 0 | Cry j 1 | 3,55 | Cup a 1 | 23,09 |
| Italy - CAAM 676 | M | 67 | Amb a 1 | 0 | Cry j 1 | 0,17 | Cup a 1 | 0,65 |
| Italy - CAAM 677 | F | 67 | Amb a 1 | 0 | Cry j 1 | 0,06 | Cup a 1 | 0,47 |
| Italy - CAAM 678 | F | 67 | Amb a 1 | 0 | Cry j 1 | 0,02 | Cup a 1 | 0,36 |
| Italy - CAAM 679 | M | 67 | Amb a 1 | 0 | Cry j 1 | 0,18 | Cup a 1 | 1,24 |
| Italy - CAAM 680 | M | 67 | Amb a 1 | 0 | Cry j 1 | 0 | Cup a 1 | 0,02 |
| Italy - CAAM 681 | M | 67 | Amb a 1 | 0 | Cry j 1 | 0,22 | Cup a 1 | 1,91 |
| Italy - CAAM 682 | F | 67 | Amb a 1 | 0 | Cry j 1 | 0,03 | Cup a 1 | 0 |
| Italy - CAAM 683 | F | 67 | Amb a 1 | 0 | Cry j 1 | 0 | Cup a 1 | 0,52 |
| Italy - CAAM 684 | M | 67 | Amb a 1 | 0 | Cry j 1 | 0 | Cup a 1 | 0,25 |
| Italy - CAAM 685 | F | 67 | Amb a 1 | 0 | Cry j 1 | 0 | Cup a 1 | 0,8 |
| Italy - CAAM 686 | F | 67 | Amb a 1 | 0 | Cry j 1 | 11,34 | Cup a 1 | 10,27 |
| Italy - CAAM 687 | M | 67 | Amb a 1 | 0 | Cry j 1 | 1,49 | Cup a 1 | 8,22 |
| Italy - CAAM 688 | F | 67 | Amb a 1 | 0 | Cry j 1 | 0,26 | Cup a 1 | 2,97 |
| Italy - CAAM 689 | F | 67 | Amb a 1 | 0 | Cry j 1 | 0 | Cup a 1 | 0,3 |
| Italy - CAAM 690 | M | 67 | Amb a 1 | 0 | Cry j 1 | 0,46 | Cup a 1 | 2,15 |
| Italy - CAAM 691 | F | 67 | Amb a 1 | 0 | Cry j 1 | 19,68 | Cup a 1 | 46,8 |
| Italy - CAAM 692 | F | 67 | Amb a 1 | 0 | Cry j 1 | 0 | Cup a 1 | 2,22 |
| Italy - CAAM 693 | F | 67 | Amb a 1 | 0 | Cry j 1 | 1,96 | Cup a 1 | 11,48 |
| Italy - CAAM 694 | M | 67 | Amb a 1 | 0 | Cry j 1 | 0,4 | Cup a 1 | 0,53 |
| Italy - CAAM 695 | M | 67 | Amb a 1 | 0 | Cry j 1 | 0,58 | Cup a 1 | 9,77 |
| Italy - CAAM 696 | F | 67 | Amb a 1 | 0 | Cry j 1 | 5,5 | Cup a 1 | 15,08 |
| Italy - CAAM 697 | F | 67 | Amb a 1 | 0 | Cry j 1 | 0 | Cup a 1 | 1,2 |
| Italy - CAAM 698 | F | 67 | Amb a 1 | 0 | Cry j 1 | 0 | Cup a 1 | 2 |
| Italy - CAAM 699 | F | 67 | Amb a 1 | 0 | Cry j 1 | 0 | Cup a 1 | 0,59 |
| Italy - CAAM 700 | F | 67 | Amb a 1 | 0 | Cry j 1 | 0 | Cup a 1 | 2,58 |
| Italy - CAAM 701 | M | 67 | Amb a 1 | 0 | Cry j 1 | 2,36 | Cup a 1 | 11,84 |
| Italy - CAAM 702 | F | 66 | Amb a 1 | 0 | Cry j 1 | 0,39 | Cup a 1 | 8,79 |
| Italy - CAAM 703 | F | 66 | Amb a 1 | 0 | Cry j 1 | 1,34 | Cup a 1 | 14,23 |
| Italy - CAAM 704 | F | 66 | Amb a 1 | 0 | Cry j 1 | 2,59 | Cup a 1 | 0 |
| Italy - CAAM 705 | M | 66 | Amb a 1 | 0 | Cry j 1 | 2,41 | Cup a 1 | 21,58 |
| Italy - CAAM 706 | M | 66 | Amb a 1 | 0 | Cry j 1 | 3,89 | Cup a 1 | 20,71 |
| Italy - CAAM 707 | F | 66 | Amb a 1 | 0 | Cry j 1 | 0 | Cup a 1 | 0,16 |
| Italy - CAAM 708 | F | 66 | Amb a 1 | 0 | Cry j 1 | 1,91 | Cup a 1 | 3,94 |
| Italy - CAAM 709 | M | 66 | Amb a 1 | 0 | Cry j 1 | 2 | Cup a 1 | 4,38 |
| Italy - CAAM 710 | F | 66 | Amb a 1 | 0 | Cry j 1 | 0,39 | Cup a 1 | 0,28 |
| Italy - CAAM 711 | M | 66 | Amb a 1 | 0 | Cry j 1 | 0,56 | Cup a 1 | 0 |
| Italy - CAAM 712 | M | 66 | Amb a 1 | 0 | Cry j 1 | 15,84 | Cup a 1 | 36,05 |
| Italy - CAAM 713 | M | 66 | Amb a 1 | 0 | Cry j 1 | 5,67 | Cup a 1 | 24,25 |
| Italy - CAAM 714 | M | 66 | Amb a 1 | 0 | Cry j 1 | 0,11 | Cup a 1 | 0,24 |
| Italy - CAAM 715 | M | 66 | Amb a 1 | 0 | Cry j 1 | 1,7 | Cup a 1 | 8,11 |
| Italy - CAAM 716 | F | 66 | Amb a 1 | 0 | Cry j 1 | 4,83 | Cup a 1 | 8,96 |
| Italy - CAAM 717 | M | 66 | Amb a 1 | 0 | Cry j 1 | 0,5 | Cup a 1 | 2,35 |
| Italy - CAAM 718 | F | 66 | Amb a 1 | 0 | Cry j 1 | 0,83 | Cup a 1 | 1,61 |
| Italy - CAAM 719 | F | 66 | Amb a 1 | 0 | Cry j 1 | 1,13 | Cup a 1 | 0,82 |
| Italy - CAAM 720 | F | 66 | Amb a 1 | 0 | Cry j 1 | 0 | Cup a 1 | 0,5 |
| Italy - CAAM 721 | M | 66 | Amb a 1 | 0 | Cry j 1 | 0,18 | Cup a 1 | 1,31 |
| Italy - CAAM 722 | F | 66 | Amb a 1 | 0 | Cry j 1 | 0 | Cup a 1 | 0,06 |
| Italy - CAAM 723 | F | 66 | Amb a 1 | 0 | Cry j 1 | 0 | Cup a 1 | 0,13 |
| Italy - CAAM 724 | M | 66 | Amb a 1 | 0 | Cry j 1 | 4,91 | Cup a 1 | 16,39 |
| Italy - CAAM 725 | M | 66 | Amb a 1 | 0 | Cry j 1 | 0,51 | Cup a 1 | 19,11 |
| Italy - CAAM 726 | F | 66 | Amb a 1 | 0 | Cry j 1 | 0,15 | Cup a 1 | 2,71 |
| Italy - CAAM 727 | F | 66 | Amb a 1 | 0 | Cry j 1 | 1,57 | Cup a 1 | 8,01 |
| Italy - CAAM 728 | M | 66 | Amb a 1 | 0 | Cry j 1 | 0 | Cup a 1 | 1,25 |
| Italy - CAAM 729 | M | 66 | Amb a 1 | 0 | Cry j 1 | 2,48 | Cup a 1 | 5,18 |
| Italy - CAAM 730 | F | 66 | Amb a 1 | 0 | Cry j 1 | 5,93 | Cup a 1 | 17,28 |
| Italy - CAAM 731 | F | 66 | Amb a 1 | 0 | Cry j 1 | 0,48 | Cup a 1 | 5,29 |
| Italy - CAAM 732 | M | 66 | Amb a 1 | 0 | Cry j 1 | 0,04 | Cup a 1 | 0,08 |
| Italy - CAAM 733 | F | 66 | Amb a 1 | 0 | Cry j 1 | 2,48 | Cup a 1 | 3,5 |
| Italy - CAAM 734 | M | 66 | Amb a 1 | 0 | Cry j 1 | 1,94 | Cup a 1 | 3,01 |
| Italy - CAAM 735 | M | 66 | Amb a 1 | 0 | Cry j 1 | 0 | Cup a 1 | 0,29 |
| Italy - CAAM 736 | M | 66 | Amb a 1 | 0 | Cry j 1 | 0,17 | Cup a 1 | 0,15 |
| Italy - CAAM 737 | F | 66 | Amb a 1 | 0 | Cry j 1 | 0,2 | Cup a 1 | 0 |
| Italy - CAAM 738 | M | 66 | Amb a 1 | 0 | Cry j 1 | 4,06 | Cup a 1 | 15,25 |
| Italy - CAAM 739 | F | 66 | Amb a 1 | 0 | Cry j 1 | 0,68 | Cup a 1 | 1,85 |
| Italy - CAAM 740 | F | 66 | Amb a 1 | 0 | Cry j 1 | 0 | Cup a 1 | 1,43 |
| Italy - CAAM 741 | F | 66 | Amb a 1 | 0 | Cry j 1 | 0 | Cup a 1 | 0,32 |
| Italy - CAAM 742 | M | 66 | Amb a 1 | 0 | Cry j 1 | 0,88 | Cup a 1 | 15,06 |
| Italy - CAAM 743 | F | 66 | Amb a 1 | 0 | Cry j 1 | 0,35 | Cup a 1 | 4,02 |
| Italy - CAAM 744 | M | 66 | Amb a 1 | 0 | Cry j 1 | 2,3 | Cup a 1 | 29 |
| Italy - CAAM 745 | M | 65 | Amb a 1 | 0 | Cry j 1 | 0,17 | Cup a 1 | 0,95 |
| Italy - CAAM 746 | M | 65 | Amb a 1 | 0 | Cry j 1 | 1,02 | Cup a 1 | 2,33 |
| Italy - CAAM 747 | F | 65 | Amb a 1 | 0 | Cry j 1 | 0,71 | Cup a 1 | 0 |
| Italy - CAAM 748 | F | 65 | Amb a 1 | 0 | Cry j 1 | 0,62 | Cup a 1 | 5,75 |
| Italy - CAAM 749 | M | 65 | Amb a 1 | 0 | Cry j 1 | 2,78 | Cup a 1 | 13,2 |
| Italy - CAAM 750 | F | 65 | Amb a 1 | 0 | Cry j 1 | 0,11 | Cup a 1 | 0,92 |
| Italy - CAAM 751 | F | 65 | Amb a 1 | 0 | Cry j 1 | 5,49 | Cup a 1 | 25,63 |
| Italy - CAAM 752 | F | 65 | Amb a 1 | 0 | Cry j 1 | 0,1 | Cup a 1 | 0,16 |
| Italy - CAAM 753 | F | 65 | Amb a 1 | 0 | Cry j 1 | 0,63 | Cup a 1 | 8,67 |
| Italy - CAAM 754 | M | 65 | Amb a 1 | 0 | Cry j 1 | 29,91 | Cup a 1 | 1,81 |
| Italy - CAAM 755 | M | 65 | Amb a 1 | 0 | Cry j 1 | 4,04 | Cup a 1 | 6,24 |
| Italy - CAAM 756 | F | 65 | Amb a 1 | 0 | Cry j 1 | 0,5 | Cup a 1 | 2,36 |
| Italy - CAAM 757 | F | 65 | Amb a 1 | 0 | Cry j 1 | 0 | Cup a 1 | 0,27 |
| Italy - CAAM 758 | F | 65 | Amb a 1 | 0 | Cry j 1 | 0,19 | Cup a 1 | 0,55 |
| Italy - CAAM 759 | F | 65 | Amb a 1 | 0 | Cry j 1 | 1 | Cup a 1 | 5,98 |
| Italy - CAAM 760 | F | 65 | Amb a 1 | 0 | Cry j 1 | 4,83 | Cup a 1 | 9,21 |
| Italy - CAAM 761 | F | 65 | Amb a 1 | 0 | Cry j 1 | 5,38 | Cup a 1 | 27,39 |
| Italy - CAAM 762 | M | 65 | Amb a 1 | 0 | Cry j 1 | 1,2 | Cup a 1 | 3,55 |
| Italy - CAAM 763 | M | 65 | Amb a 1 | 0 | Cry j 1 | 5,05 | Cup a 1 | 9,35 |
| Italy - CAAM 764 | M | 65 | Amb a 1 | 0 | Cry j 1 | 0 | Cup a 1 | 0,03 |
| Italy - CAAM 765 | F | 65 | Amb a 1 | 0 | Cry j 1 | 0 | Cup a 1 | 0,42 |
| Italy - CAAM 766 | M | 65 | Amb a 1 | 0 | Cry j 1 | 0,07 | Cup a 1 | 0,25 |
| Italy - CAAM 767 | F | 65 | Amb a 1 | 0 | Cry j 1 | 3,59 | Cup a 1 | 5,31 |
| Italy - CAAM 768 | F | 65 | Amb a 1 | 0 | Cry j 1 | 3,16 | Cup a 1 | 2,78 |
| Italy - CAAM 769 | M | 65 | Amb a 1 | 0 | Cry j 1 | 0 | Cup a 1 | 2,61 |
| Italy - CAAM 770 | F | 65 | Amb a 1 | 0 | Cry j 1 | 1,71 | Cup a 1 | 5,58 |
| Italy - CAAM 771 | M | 65 | Amb a 1 | 0 | Cry j 1 | 0,55 | Cup a 1 | 0 |
| Italy - CAAM 772 | F | 65 | Amb a 1 | 0 | Cry j 1 | 0,07 | Cup a 1 | 0,55 |
| Italy - CAAM 773 | F | 65 | Amb a 1 | 0 | Cry j 1 | 0,12 | Cup a 1 | 1,05 |
| Italy - CAAM 774 | F | 65 | Amb a 1 | 0 | Cry j 1 | 0 | Cup a 1 | 0,46 |
| Italy - CAAM 775 | M | 65 | Amb a 1 | 0 | Cry j 1 | 0,84 | Cup a 1 | 1,69 |
| Italy - CAAM 776 | F | 65 | Amb a 1 | 0 | Cry j 1 | 1,16 | Cup a 1 | 14,75 |
| Italy - CAAM 777 | M | 65 | Amb a 1 | 0 | Cry j 1 | 2,28 | Cup a 1 | 6,03 |
| Italy - CAAM 778 | F | 65 | Amb a 1 | 0 | Cry j 1 | 0,38 | Cup a 1 | 1,28 |
| Italy - CAAM 779 | F | 65 | Amb a 1 | 0 | Cry j 1 | 13,35 | Cup a 1 | 12,92 |
| Italy - CAAM 780 | F | 65 | Amb a 1 | 0 | Cry j 1 | 0,35 | Cup a 1 | 2,55 |
| Italy - CAAM 781 | M | 65 | Amb a 1 | 0 | Cry j 1 | 3,91 | Cup a 1 | 15,34 |
| Italy - CAAM 782 | F | 65 | Amb a 1 | 0 | Cry j 1 | 0 | Cup a 1 | 0,46 |
| Italy - CAAM 783 | F | 65 | Amb a 1 | 0 | Cry j 1 | 0,19 | Cup a 1 | 1,07 |
| Italy - CAAM 784 | M | 65 | Amb a 1 | 0 | Cry j 1 | 0 | Cup a 1 | 9,2 |
| Italy - CAAM 785 | F | 64 | Amb a 1 | 0 | Cry j 1 | 0 | Cup a 1 | 1,19 |
| Italy - CAAM 786 | F | 64 | Amb a 1 | 0 | Cry j 1 | 1,81 | Cup a 1 | 8,61 |
| Italy - CAAM 787 | F | 64 | Amb a 1 | 0 | Cry j 1 | 0,27 | Cup a 1 | 2,45 |
| Italy - CAAM 788 | M | 64 | Amb a 1 | 0 | Cry j 1 | 0 | Cup a 1 | 2,14 |
| Italy - CAAM 789 | F | 64 | Amb a 1 | 0 | Cry j 1 | 0,91 | Cup a 1 | 2,55 |
| Italy - CAAM 790 | F | 64 | Amb a 1 | 0 | Cry j 1 | 0 | Cup a 1 | 0,06 |
| Italy - CAAM 791 | F | 64 | Amb a 1 | 0 | Cry j 1 | 0,38 | Cup a 1 | 3,23 |
| Italy - CAAM 792 | F | 64 | Amb a 1 | 0 | Cry j 1 | 0,37 | Cup a 1 | 0 |
| Italy - CAAM 793 | F | 64 | Amb a 1 | 0 | Cry j 1 | 0,84 | Cup a 1 | 2,41 |
| Italy - CAAM 794 | M | 64 | Amb a 1 | 0 | Cry j 1 | 7,22 | Cup a 1 | 10,62 |
| Italy - CAAM 795 | M | 64 | Amb a 1 | 0 | Cry j 1 | 28,6 | Cup a 1 | 2,48 |
| Italy - CAAM 796 | F | 64 | Amb a 1 | 0 | Cry j 1 | 3,65 | Cup a 1 | 0 |
| Italy - CAAM 797 | F | 64 | Amb a 1 | 0 | Cry j 1 | 0 | Cup a 1 | 1,02 |
| Italy - CAAM 798 | F | 64 | Amb a 1 | 0 | Cry j 1 | 0 | Cup a 1 | 0,06 |
| Italy - CAAM 799 | M | 64 | Amb a 1 | 0 | Cry j 1 | 0,05 | Cup a 1 | 0,09 |
| Italy - CAAM 800 | M | 64 | Amb a 1 | 0 | Cry j 1 | 0,99 | Cup a 1 | 1,11 |
| Italy - CAAM 801 | F | 64 | Amb a 1 | 0 | Cry j 1 | 0 | Cup a 1 | 0,12 |
| Italy - CAAM 802 | F | 64 | Amb a 1 | 0 | Cry j 1 | 2,98 | Cup a 1 | 15,8 |
| Italy - CAAM 803 | M | 64 | Amb a 1 | 0 | Cry j 1 | 0 | Cup a 1 | 0,15 |
| Italy - CAAM 804 | F | 64 | Amb a 1 | 0 | Cry j 1 | 2,12 | Cup a 1 | 4,9 |
| Italy - CAAM 805 | F | 64 | Amb a 1 | 0 | Cry j 1 | 0 | Cup a 1 | 0,41 |
| Italy - CAAM 806 | M | 64 | Amb a 1 | 0 | Cry j 1 | 0 | Cup a 1 | 0,16 |
| Italy - CAAM 807 | F | 64 | Amb a 1 | 0 | Cry j 1 | 1,89 | Cup a 1 | 2,83 |
| Italy - CAAM 808 | F | 64 | Amb a 1 | 0 | Cry j 1 | 0,72 | Cup a 1 | 1,27 |
| Italy - CAAM 809 | M | 64 | Amb a 1 | 0 | Cry j 1 | 0 | Cup a 1 | 1,13 |
| Italy - CAAM 810 | F | 64 | Amb a 1 | 0 | Cry j 1 | 0 | Cup a 1 | 0,17 |
| Italy - CAAM 811 | M | 64 | Amb a 1 | 0 | Cry j 1 | 2,26 | Cup a 1 | 5,51 |
| Italy - CAAM 812 | F | 64 | Amb a 1 | 0 | Cry j 1 | 0,06 | Cup a 1 | 0,17 |
| Italy - CAAM 813 | F | 64 | Amb a 1 | 0 | Cry j 1 | 0,09 | Cup a 1 | 0,87 |
| Italy - CAAM 814 | F | 64 | Amb a 1 | 0 | Cry j 1 | 0 | Cup a 1 | 0,9 |
| Italy - CAAM 815 | M | 64 | Amb a 1 | 0 | Cry j 1 | 0 | Cup a 1 | 0,57 |
| Italy - CAAM 816 | F | 64 | Amb a 1 | 0 | Cry j 1 | 3,25 | Cup a 1 | 4,05 |
| Italy - CAAM 817 | F | 64 | Amb a 1 | 0 | Cry j 1 | 1,36 | Cup a 1 | 5,91 |
| Italy - CAAM 818 | F | 64 | Amb a 1 | 0 | Cry j 1 | 0 | Cup a 1 | 0,93 |
| Italy - CAAM 819 | F | 64 | Amb a 1 | 0 | Cry j 1 | 0 | Cup a 1 | 0,85 |
| Italy - CAAM 820 | F | 64 | Amb a 1 | 0 | Cry j 1 | 1,9 | Cup a 1 | 5,67 |
| Italy - CAAM 821 | F | 64 | Amb a 1 | 0 | Cry j 1 | 0,07 | Cup a 1 | 0 |
| Italy - CAAM 822 | F | 64 | Amb a 1 | 0 | Cry j 1 | 0 | Cup a 1 | 0,69 |
| Italy - CAAM 823 | F | 64 | Amb a 1 | 0 | Cry j 1 | 0 | Cup a 1 | 2,08 |
| Italy - CAAM 824 | M | 64 | Amb a 1 | 0 | Cry j 1 | 0,63 | Cup a 1 | 0,88 |
| Italy - CAAM 825 | F | 64 | Amb a 1 | 0 | Cry j 1 | 0 | Cup a 1 | 0,24 |
| Italy - CAAM 826 | M | 64 | Amb a 1 | 0 | Cry j 1 | 0 | Cup a 1 | 3,88 |
| Italy - CAAM 827 | M | 64 | Amb a 1 | 0 | Cry j 1 | 0 | Cup a 1 | 1,7 |
| Italy - CAAM 828 | M | 64 | Amb a 1 | 0 | Cry j 1 | 0,09 | Cup a 1 | 0,83 |
| Italy - CAAM 829 | F | 64 | Amb a 1 | 0 | Cry j 1 | 1,3 | Cup a 1 | 14 |
| Italy - CAAM 830 | F | 64 | Amb a 1 | 0 | Cry j 1 | 0,18 | Cup a 1 | 6,87 |
| Italy - CAAM 831 | M | 64 | Amb a 1 | 0 | Cry j 1 | 0 | Cup a 1 | 2,37 |
| Italy - CAAM 832 | F | 63 | Amb a 1 | 0 | Cry j 1 | 0 | Cup a 1 | 0,52 |
| Italy - CAAM 833 | M | 63 | Amb a 1 | 0 | Cry j 1 | 0,59 | Cup a 1 | 1,19 |
| Italy - CAAM 834 | F | 63 | Amb a 1 | 0 | Cry j 1 | 0,89 | Cup a 1 | 4,98 |
| Italy - CAAM 835 | F | 63 | Amb a 1 | 0 | Cry j 1 | 1,62 | Cup a 1 | 2,54 |
| Italy - CAAM 836 | F | 63 | Amb a 1 | 0 | Cry j 1 | 0,02 | Cup a 1 | 2,9 |
| Italy - CAAM 837 | F | 63 | Amb a 1 | 0 | Cry j 1 | 0,98 | Cup a 1 | 13,55 |
| Italy - CAAM 838 | F | 63 | Amb a 1 | 0 | Cry j 1 | 1,74 | Cup a 1 | 6,39 |
| Italy - CAAM 839 | F | 63 | Amb a 1 | 0 | Cry j 1 | 0,18 | Cup a 1 | 0,77 |
| Italy - CAAM 840 | F | 63 | Amb a 1 | 0 | Cry j 1 | 2,8 | Cup a 1 | 3,22 |
| Italy - CAAM 841 | F | 63 | Amb a 1 | 0 | Cry j 1 | 3,86 | Cup a 1 | 5,33 |
| Italy - CAAM 842 | M | 63 | Amb a 1 | 0 | Cry j 1 | 0,11 | Cup a 1 | 0,21 |
| Italy - CAAM 843 | M | 63 | Amb a 1 | 0 | Cry j 1 | 0 | Cup a 1 | 7,41 |
| Italy - CAAM 844 | F | 63 | Amb a 1 | 0 | Cry j 1 | 2,85 | Cup a 1 | 2,59 |
| Italy - CAAM 845 | F | 63 | Amb a 1 | 0 | Cry j 1 | 3,33 | Cup a 1 | 0 |
| Italy - CAAM 846 | M | 63 | Amb a 1 | 0 | Cry j 1 | 12,92 | Cup a 1 | 0 |
| Italy - CAAM 847 | M | 63 | Amb a 1 | 0 | Cry j 1 | 1,81 | Cup a 1 | 3,94 |
| Italy - CAAM 848 | F | 63 | Amb a 1 | 0 | Cry j 1 | 10,05 | Cup a 1 | 18,4 |
| Italy - CAAM 849 | M | 63 | Amb a 1 | 0 | Cry j 1 | 1,69 | Cup a 1 | 0,68 |
| Italy - CAAM 850 | M | 63 | Amb a 1 | 0 | Cry j 1 | 0,32 | Cup a 1 | 1,38 |
| Italy - CAAM 851 | F | 63 | Amb a 1 | 0 | Cry j 1 | 0 | Cup a 1 | 0,22 |
| Italy - CAAM 852 | F | 63 | Amb a 1 | 0 | Cry j 1 | 0,75 | Cup a 1 | 1,3 |
| Italy - CAAM 853 | F | 63 | Amb a 1 | 0 | Cry j 1 | 0,1 | Cup a 1 | 1,98 |
| Italy - CAAM 854 | F | 63 | Amb a 1 | 0 | Cry j 1 | 0,68 | Cup a 1 | 0,94 |
| Italy - CAAM 855 | M | 63 | Amb a 1 | 0 | Cry j 1 | 0,05 | Cup a 1 | 0 |
| Italy - CAAM 856 | M | 63 | Amb a 1 | 0 | Cry j 1 | 0 | Cup a 1 | 0,03 |
| Italy - CAAM 857 | F | 63 | Amb a 1 | 0 | Cry j 1 | 0 | Cup a 1 | 0,18 |
| Italy - CAAM 858 | M | 63 | Amb a 1 | 0 | Cry j 1 | 0 | Cup a 1 | 4,1 |
| Italy - CAAM 859 | M | 63 | Amb a 1 | 0 | Cry j 1 | 0 | Cup a 1 | 0,39 |
| Italy - CAAM 860 | F | 63 | Amb a 1 | 0 | Cry j 1 | 0 | Cup a 1 | 0,61 |
| Italy - CAAM 861 | M | 63 | Amb a 1 | 0 | Cry j 1 | 1,37 | Cup a 1 | 15,9 |
| Italy - CAAM 862 | F | 63 | Amb a 1 | 0 | Cry j 1 | 0 | Cup a 1 | 1,23 |
| Italy - CAAM 863 | M | 63 | Amb a 1 | 0 | Cry j 1 | 0,67 | Cup a 1 | 0,18 |
| Italy - CAAM 864 | F | 63 | Amb a 1 | 0 | Cry j 1 | 2,6 | Cup a 1 | 6,12 |
| Italy - CAAM 865 | F | 63 | Amb a 1 | 0 | Cry j 1 | 0,09 | Cup a 1 | 1,13 |
| Italy - CAAM 866 | M | 63 | Amb a 1 | 0 | Cry j 1 | 0 | Cup a 1 | 3,53 |
| Italy - CAAM 867 | M | 63 | Amb a 1 | 0 | Cry j 1 | 0 | Cup a 1 | 2,74 |
| Italy - CAAM 868 | M | 63 | Amb a 1 | 0 | Cry j 1 | 4,57 | Cup a 1 | 24,45 |
| Italy - CAAM 869 | F | 63 | Amb a 1 | 0 | Cry j 1 | 1,82 | Cup a 1 | 6,99 |
| Italy - CAAM 870 | F | 63 | Amb a 1 | 0 | Cry j 1 | 4,88 | Cup a 1 | 11,71 |
| Italy - CAAM 871 | F | 63 | Amb a 1 | 0 | Cry j 1 | 0 | Cup a 1 | 0,22 |
| Italy - CAAM 872 | F | 63 | Amb a 1 | 0 | Cry j 1 | 0,09 | Cup a 1 | 1,32 |
| Italy - CAAM 873 | F | 63 | Amb a 1 | 0 | Cry j 1 | 1,17 | Cup a 1 | 9,92 |
| Italy - CAAM 874 | M | 63 | Amb a 1 | 0 | Cry j 1 | 0 | Cup a 1 | 0,29 |
| Italy - CAAM 875 | F | 63 | Amb a 1 | 0 | Cry j 1 | 0,4 | Cup a 1 | 3,6 |
| Italy - CAAM 876 | F | 63 | Amb a 1 | 0 | Cry j 1 | 1,8 | Cup a 1 | 15 |
| Italy - CAAM 877 | F | 63 | Amb a 1 | 0 | Cry j 1 | 0,08 | Cup a 1 | 0,91 |
| Italy - CAAM 878 | M | 63 | Amb a 1 | 0 | Cry j 1 | 0 | Cup a 1 | 0,06 |
| Italy - CAAM 879 | M | 63 | Amb a 1 | 0 | Cry j 1 | 0 | Cup a 1 | 3,8 |
| Italy - CAAM 880 | F | 62 | Amb a 1 | 0 | Cry j 1 | 1,56 | Cup a 1 | 4,2 |
| Italy - CAAM 881 | F | 62 | Amb a 1 | 0 | Cry j 1 | 0 | Cup a 1 | 0,86 |
| Italy - CAAM 882 | F | 62 | Amb a 1 | 0 | Cry j 1 | 2,9 | Cup a 1 | 30,95 |
| Italy - CAAM 883 | F | 62 | Amb a 1 | 0 | Cry j 1 | 6,46 | Cup a 1 | 10,63 |
| Italy - CAAM 884 | F | 62 | Amb a 1 | 0 | Cry j 1 | 0,21 | Cup a 1 | 0,83 |
| Italy - CAAM 885 | F | 62 | Amb a 1 | 0 | Cry j 1 | 1,71 | Cup a 1 | 1,49 |
| Italy - CAAM 886 | F | 62 | Amb a 1 | 0 | Cry j 1 | 0,43 | Cup a 1 | 2,27 |
| Italy - CAAM 887 | M | 62 | Amb a 1 | 0 | Cry j 1 | 1,25 | Cup a 1 | 1,6 |
| Italy - CAAM 888 | F | 62 | Amb a 1 | 0 | Cry j 1 | 2,25 | Cup a 1 | 5,69 |
| Italy - CAAM 889 | M | 62 | Amb a 1 | 0 | Cry j 1 | 0,58 | Cup a 1 | 1,76 |
| Italy - CAAM 890 | F | 62 | Amb a 1 | 0 | Cry j 1 | 0,73 | Cup a 1 | 5,06 |
| Italy - CAAM 891 | M | 62 | Amb a 1 | 0 | Cry j 1 | 13,32 | Cup a 1 | 17,38 |
| Italy - CAAM 892 | M | 62 | Amb a 1 | 0 | Cry j 1 | 3,32 | Cup a 1 | 6,67 |
| Italy - CAAM 893 | F | 62 | Amb a 1 | 0 | Cry j 1 | 0,31 | Cup a 1 | 0,47 |
| Italy - CAAM 894 | F | 62 | Amb a 1 | 0 | Cry j 1 | 0,64 | Cup a 1 | 0 |
| Italy - CAAM 895 | F | 62 | Amb a 1 | 0 | Cry j 1 | 2,93 | Cup a 1 | 1,95 |
| Italy - CAAM 896 | M | 62 | Amb a 1 | 0 | Cry j 1 | 1,31 | Cup a 1 | 1,55 |
| Italy - CAAM 897 | F | 62 | Amb a 1 | 0 | Cry j 1 | 0,49 | Cup a 1 | 0,36 |
| Italy - CAAM 898 | F | 62 | Amb a 1 | 0 | Cry j 1 | 4,58 | Cup a 1 | 0 |
| Italy - CAAM 899 | M | 62 | Amb a 1 | 0 | Cry j 1 | 0 | Cup a 1 | 0,46 |
| Italy - CAAM 900 | M | 62 | Amb a 1 | 0 | Cry j 1 | 5,12 | Cup a 1 | 0 |
| Italy - CAAM 901 | M | 62 | Amb a 1 | 0 | Cry j 1 | 1,7 | Cup a 1 | 0 |
| Italy - CAAM 902 | F | 62 | Amb a 1 | 0 | Cry j 1 | 2,21 | Cup a 1 | 6,9 |
| Italy - CAAM 903 | M | 62 | Amb a 1 | 0 | Cry j 1 | 7,78 | Cup a 1 | 8,42 |
| Italy - CAAM 904 | M | 62 | Amb a 1 | 0 | Cry j 1 | 1,29 | Cup a 1 | 17,37 |
| Italy - CAAM 905 | M | 62 | Amb a 1 | 0 | Cry j 1 | 0,64 | Cup a 1 | 14,54 |
| Italy - CAAM 906 | F | 62 | Amb a 1 | 0 | Cry j 1 | 0 | Cup a 1 | 0,12 |
| Italy - CAAM 907 | M | 62 | Amb a 1 | 0 | Cry j 1 | 10,94 | Cup a 1 | 37,33 |
| Italy - CAAM 908 | M | 62 | Amb a 1 | 0 | Cry j 1 | 0,64 | Cup a 1 | 1,53 |
| Italy - CAAM 909 | F | 62 | Amb a 1 | 0 | Cry j 1 | 0,61 | Cup a 1 | 6,3 |
| Italy - CAAM 910 | F | 62 | Amb a 1 | 0 | Cry j 1 | 1,51 | Cup a 1 | 17,75 |
| Italy - CAAM 911 | F | 62 | Amb a 1 | 0 | Cry j 1 | 0,78 | Cup a 1 | 4,09 |
| Italy - CAAM 912 | M | 62 | Amb a 1 | 0 | Cry j 1 | 0,04 | Cup a 1 | 0,36 |
| Italy - CAAM 913 | M | 62 | Amb a 1 | 0 | Cry j 1 | 1,71 | Cup a 1 | 2,79 |
| Italy - CAAM 914 | F | 62 | Amb a 1 | 0 | Cry j 1 | 1,4 | Cup a 1 | 1,44 |
| Italy - CAAM 915 | M | 62 | Amb a 1 | 0 | Cry j 1 | 0,98 | Cup a 1 | 3,81 |
| Italy - CAAM 916 | F | 62 | Amb a 1 | 0 | Cry j 1 | 0,07 | Cup a 1 | 0,13 |
| Italy - CAAM 917 | M | 62 | Amb a 1 | 0 | Cry j 1 | 0,27 | Cup a 1 | 2,45 |
| Italy - CAAM 918 | F | 62 | Amb a 1 | 0 | Cry j 1 | 2,11 | Cup a 1 | 12,03 |
| Italy - CAAM 919 | F | 62 | Amb a 1 | 0 | Cry j 1 | 0,04 | Cup a 1 | 1,03 |
| Italy - CAAM 920 | M | 62 | Amb a 1 | 0 | Cry j 1 | 0,28 | Cup a 1 | 1,11 |
| Italy - CAAM 921 | M | 62 | Amb a 1 | 0 | Cry j 1 | 0,29 | Cup a 1 | 0,26 |
| Italy - CAAM 922 | M | 62 | Amb a 1 | 0 | Cry j 1 | 0,5 | Cup a 1 | 1,94 |
| Italy - CAAM 923 | M | 62 | Amb a 1 | 0 | Cry j 1 | 0,29 | Cup a 1 | 2,52 |
| Italy - CAAM 924 | F | 62 | Amb a 1 | 0 | Cry j 1 | 0,21 | Cup a 1 | 9,85 |
| Italy - CAAM 925 | F | 62 | Amb a 1 | 0 | Cry j 1 | 1,27 | Cup a 1 | 8,64 |
| Italy - CAAM 926 | M | 62 | Amb a 1 | 0 | Cry j 1 | 0 | Cup a 1 | 1,36 |
| Italy - CAAM 927 | F | 62 | Amb a 1 | 0 | Cry j 1 | 4 | Cup a 1 | 22,72 |
| Italy - CAAM 928 | M | 62 | Amb a 1 | 0 | Cry j 1 | 0 | Cup a 1 | 0,84 |
| Italy - CAAM 929 | F | 62 | Amb a 1 | 0 | Cry j 1 | 0 | Cup a 1 | 0,12 |
| Italy - CAAM 930 | F | 62 | Amb a 1 | 0 | Cry j 1 | 0,69 | Cup a 1 | 3,13 |
| Italy - CAAM 931 | F | 62 | Amb a 1 | 0 | Cry j 1 | 1,63 | Cup a 1 | 3,67 |
| Italy - CAAM 932 | M | 62 | Amb a 1 | 0 | Cry j 1 | 9,3 | Cup a 1 | 55,78 |
| Italy - CAAM 933 | F | 62 | Amb a 1 | 0 | Cry j 1 | 0 | Cup a 1 | 13 |
| Italy - CAAM 934 | F | 62 | Amb a 1 | 0 | Cry j 1 | 0 | Cup a 1 | 0,87 |
| Italy - CAAM 935 | F | 62 | Amb a 1 | 0 | Cry j 1 | 8,07 | Cup a 1 | 12,59 |
| Italy - CAAM 936 | F | 62 | Amb a 1 | 0 | Cry j 1 | 1,25 | Cup a 1 | 36,24 |
| Italy - CAAM 937 | M | 62 | Amb a 1 | 0 | Cry j 1 | 0 | Cup a 1 | 5,86 |
| Italy - CAAM 938 | M | 61 | Amb a 1 | 0 | Cry j 1 | 0 | Cup a 1 | 0,39 |
| Italy - CAAM 939 | M | 61 | Amb a 1 | 0 | Cry j 1 | 2,1 | Cup a 1 | 26,68 |
| Italy - CAAM 940 | F | 61 | Amb a 1 | 0 | Cry j 1 | 0,22 | Cup a 1 | 1,08 |
| Italy - CAAM 941 | F | 61 | Amb a 1 | 0 | Cry j 1 | 6,23 | Cup a 1 | 6,47 |
| Italy - CAAM 942 | F | 61 | Amb a 1 | 0 | Cry j 1 | 0,96 | Cup a 1 | 1,91 |
| Italy - CAAM 943 | F | 61 | Amb a 1 | 0 | Cry j 1 | 4,87 | Cup a 1 | 7,74 |
| Italy - CAAM 944 | F | 61 | Amb a 1 | 0 | Cry j 1 | 0,22 | Cup a 1 | 0 |
| Italy - CAAM 945 | F | 61 | Amb a 1 | 0 | Cry j 1 | 7,3 | Cup a 1 | 7,44 |
| Italy - CAAM 946 | M | 61 | Amb a 1 | 0 | Cry j 1 | 14,29 | Cup a 1 | 8,59 |
| Italy - CAAM 947 | F | 61 | Amb a 1 | 0 | Cry j 1 | 2,96 | Cup a 1 | 5,64 |
| Italy - CAAM 948 | F | 61 | Amb a 1 | 0 | Cry j 1 | 2,86 | Cup a 1 | 9,73 |
| Italy - CAAM 949 | M | 61 | Amb a 1 | 0 | Cry j 1 | 0,4 | Cup a 1 | 5,8 |
| Italy - CAAM 950 | M | 61 | Amb a 1 | 0 | Cry j 1 | 0 | Cup a 1 | 1,35 |
| Italy - CAAM 951 | M | 61 | Amb a 1 | 0 | Cry j 1 | 9,24 | Cup a 1 | 0,96 |
| Italy - CAAM 952 | F | 61 | Amb a 1 | 0 | Cry j 1 | 58,23 | Cup a 1 | 2,9 |
| Italy - CAAM 953 | F | 61 | Amb a 1 | 0 | Cry j 1 | 3,57 | Cup a 1 | 0 |
| Italy - CAAM 954 | F | 61 | Amb a 1 | 0 | Cry j 1 | 1,64 | Cup a 1 | 1,89 |
| Italy - CAAM 955 | F | 61 | Amb a 1 | 0 | Cry j 1 | 0,91 | Cup a 1 | 4,21 |
| Italy - CAAM 956 | M | 61 | Amb a 1 | 0 | Cry j 1 | 2,67 | Cup a 1 | 8,44 |
| Italy - CAAM 957 | M | 61 | Amb a 1 | 0 | Cry j 1 | 0,05 | Cup a 1 | 1,91 |
| Italy - CAAM 958 | M | 61 | Amb a 1 | 0 | Cry j 1 | 0,07 | Cup a 1 | 0,44 |
| Italy - CAAM 959 | M | 61 | Amb a 1 | 0 | Cry j 1 | 0,5 | Cup a 1 | 6,64 |
| Italy - CAAM 960 | F | 61 | Amb a 1 | 0 | Cry j 1 | 0,88 | Cup a 1 | 5,67 |
| Italy - CAAM 961 | M | 61 | Amb a 1 | 0 | Cry j 1 | 0 | Cup a 1 | 0,75 |
| Italy - CAAM 962 | F | 61 | Amb a 1 | 0 | Cry j 1 | 10,56 | Cup a 1 | 13,88 |
| Italy - CAAM 963 | F | 61 | Amb a 1 | 0 | Cry j 1 | 3,36 | Cup a 1 | 2,56 |
| Italy - CAAM 964 | M | 61 | Amb a 1 | 0 | Cry j 1 | 0 | Cup a 1 | 0,07 |
| Italy - CAAM 965 | F | 61 | Amb a 1 | 0 | Cry j 1 | 0,03 | Cup a 1 | 2,35 |
| Italy - CAAM 966 | F | 61 | Amb a 1 | 0 | Cry j 1 | 0 | Cup a 1 | 1,04 |
| Italy - CAAM 967 | F | 61 | Amb a 1 | 0 | Cry j 1 | 0 | Cup a 1 | 0,24 |
| Italy - CAAM 968 | F | 61 | Amb a 1 | 0 | Cry j 1 | 0,07 | Cup a 1 | 0,35 |
| Italy - CAAM 969 | F | 61 | Amb a 1 | 0 | Cry j 1 | 0,76 | Cup a 1 | 2,29 |
| Italy - CAAM 970 | M | 61 | Amb a 1 | 0 | Cry j 1 | 0 | Cup a 1 | 0,27 |
| Italy - CAAM 971 | M | 61 | Amb a 1 | 0 | Cry j 1 | 1,41 | Cup a 1 | 12,06 |
| Italy - CAAM 972 | F | 61 | Amb a 1 | 0 | Cry j 1 | 0,81 | Cup a 1 | 5,03 |
| Italy - CAAM 973 | F | 61 | Amb a 1 | 0 | Cry j 1 | 0,84 | Cup a 1 | 7,59 |
| Italy - CAAM 974 | M | 61 | Amb a 1 | 0 | Cry j 1 | 1,6 | Cup a 1 | 7,62 |
| Italy - CAAM 975 | M | 61 | Amb a 1 | 0 | Cry j 1 | 4,7 | Cup a 1 | 28,05 |
| Italy - CAAM 976 | F | 61 | Amb a 1 | 0 | Cry j 1 | 2,42 | Cup a 1 | 7,43 |
| Italy - CAAM 977 | F | 61 | Amb a 1 | 0 | Cry j 1 | 1,62 | Cup a 1 | 16,02 |
| Italy - CAAM 978 | F | 61 | Amb a 1 | 0 | Cry j 1 | 0 | Cup a 1 | 0,3 |
| Italy - CAAM 979 | F | 61 | Amb a 1 | 0 | Cry j 1 | 0 | Cup a 1 | 0,32 |
| Italy - CAAM 980 | F | 61 | Amb a 1 | 0 | Cry j 1 | 0 | Cup a 1 | 0,14 |
| Italy - CAAM 981 | M | 61 | Amb a 1 | 0 | Cry j 1 | 0 | Cup a 1 | 0,24 |
| Italy - CAAM 982 | F | 61 | Amb a 1 | 0 | Cry j 1 | 0 | Cup a 1 | 0,53 |
| Italy - CAAM 983 | F | 61 | Amb a 1 | 0 | Cry j 1 | 0,56 | Cup a 1 | 10,11 |
| Italy - CAAM 984 | F | 61 | Amb a 1 | 0 | Cry j 1 | 1 | Cup a 1 | 5,4 |
| Italy - CAAM 985 | F | 61 | Amb a 1 | 0 | Cry j 1 | 0 | Cup a 1 | 2,6 |
| Italy - CAAM 986 | F | 61 | Amb a 1 | 0 | Cry j 1 | 2,38 | Cup a 1 | 16,51 |
| Italy - CAAM 987 | F | 60 | Amb a 1 | 0 | Cry j 1 | 5,93 | Cup a 1 | 10,94 |
| Italy - CAAM 988 | F | 60 | Amb a 1 | 0 | Cry j 1 | 0 | Cup a 1 | 0,16 |
| Italy - CAAM 989 | F | 60 | Amb a 1 | 0 | Cry j 1 | 5,64 | Cup a 1 | 2,46 |
| Italy - CAAM 990 | F | 60 | Amb a 1 | 0 | Cry j 1 | 0,16 | Cup a 1 | 2,99 |
| Italy - CAAM 991 | F | 60 | Amb a 1 | 0 | Cry j 1 | 0 | Cup a 1 | 3,14 |
| Italy - CAAM 992 | F | 60 | Amb a 1 | 0 | Cry j 1 | 0,21 | Cup a 1 | 10,31 |
| Italy - CAAM 993 | F | 60 | Amb a 1 | 0 | Cry j 1 | 1,32 | Cup a 1 | 17 |
| Italy - CAAM 994 | M | 60 | Amb a 1 | 0 | Cry j 1 | 3 | Cup a 1 | 3,42 |
| Italy - CAAM 995 | M | 60 | Amb a 1 | 0 | Cry j 1 | 1,5 | Cup a 1 | 8,79 |
| Italy - CAAM 996 | F | 60 | Amb a 1 | 0 | Cry j 1 | 3,73 | Cup a 1 | 16,01 |
| Italy - CAAM 997 | M | 60 | Amb a 1 | 0 | Cry j 1 | 0,56 | Cup a 1 | 1,4 |
| Italy - CAAM 998 | M | 60 | Amb a 1 | 0 | Cry j 1 | 2,19 | Cup a 1 | 3,86 |
| Italy - CAAM 999 | F | 60 | Amb a 1 | 0 | Cry j 1 | 2,83 | Cup a 1 | 4,13 |
| Italy - CAAM 1000 | F | 60 | Amb a 1 | 0 | Cry j 1 | 0,68 | Cup a 1 | 0,66 |
| Italy - CAAM 1001 | F | 60 | Amb a 1 | 0 | Cry j 1 | 6,52 | Cup a 1 | 5,64 |
| Italy - CAAM 1002 | F | 60 | Amb a 1 | 0 | Cry j 1 | 1,46 | Cup a 1 | 1,1 |
| Italy - CAAM 1003 | F | 60 | Amb a 1 | 0 | Cry j 1 | 0,37 | Cup a 1 | 0,77 |
| Italy - CAAM 1004 | M | 60 | Amb a 1 | 0 | Cry j 1 | 0,42 | Cup a 1 | 1,1 |
| Italy - CAAM 1005 | F | 60 | Amb a 1 | 0 | Cry j 1 | 0,41 | Cup a 1 | 2,78 |
| Italy - CAAM 1006 | M | 60 | Amb a 1 | 0 | Cry j 1 | 0,56 | Cup a 1 | 0,48 |
| Italy - CAAM 1007 | M | 60 | Amb a 1 | 0 | Cry j 1 | 0 | Cup a 1 | 0,28 |
| Italy - CAAM 1008 | F | 60 | Amb a 1 | 0 | Cry j 1 | 0 | Cup a 1 | 2,25 |
| Italy - CAAM 1009 | M | 60 | Amb a 1 | 0 | Cry j 1 | 5,53 | Cup a 1 | 6,81 |
| Italy - CAAM 1010 | M | 60 | Amb a 1 | 0 | Cry j 1 | 0,51 | Cup a 1 | 1,84 |
| Italy - CAAM 1011 | F | 60 | Amb a 1 | 0 | Cry j 1 | 0,02 | Cup a 1 | 1,26 |
| Italy - CAAM 1012 | M | 60 | Amb a 1 | 0 | Cry j 1 | 0 | Cup a 1 | 0,18 |
| Italy - CAAM 1013 | F | 60 | Amb a 1 | 0 | Cry j 1 | 0,38 | Cup a 1 | 1,87 |
| Italy - CAAM 1014 | F | 60 | Amb a 1 | 0 | Cry j 1 | 0,12 | Cup a 1 | 0,51 |
| Italy - CAAM 1015 | F | 60 | Amb a 1 | 0 | Cry j 1 | 0,8 | Cup a 1 | 1,61 |
| Italy - CAAM 1016 | F | 60 | Amb a 1 | 0 | Cry j 1 | 1,06 | Cup a 1 | 1,61 |
| Italy - CAAM 1017 | F | 60 | Amb a 1 | 0 | Cry j 1 | 0,27 | Cup a 1 | 0,4 |
| Italy - CAAM 1018 | F | 60 | Amb a 1 | 0 | Cry j 1 | 0,32 | Cup a 1 | 0,4 |
| Italy - CAAM 1019 | M | 60 | Amb a 1 | 0 | Cry j 1 | 0 | Cup a 1 | 0,07 |
| Italy - CAAM 1020 | M | 60 | Amb a 1 | 0 | Cry j 1 | 0,8 | Cup a 1 | 15 |
| Italy - CAAM 1021 | M | 60 | Amb a 1 | 0 | Cry j 1 | 1,48 | Cup a 1 | 0,77 |
| Italy - CAAM 1022 | F | 60 | Amb a 1 | 0 | Cry j 1 | 0 | Cup a 1 | 0,05 |
| Italy - CAAM 1023 | F | 60 | Amb a 1 | 0 | Cry j 1 | 0,13 | Cup a 1 | 2,55 |
| Italy - CAAM 1024 | F | 60 | Amb a 1 | 0 | Cry j 1 | 4,58 | Cup a 1 | 10,45 |
| Italy - CAAM 1025 | F | 60 | Amb a 1 | 0 | Cry j 1 | 5,01 | Cup a 1 | 23,76 |
| Italy - CAAM 1026 | F | 60 | Amb a 1 | 0 | Cry j 1 | 0 | Cup a 1 | 0,08 |
| Italy - CAAM 1027 | F | 60 | Amb a 1 | 0 | Cry j 1 | 8,03 | Cup a 1 | 19,47 |
| Italy - CAAM 1028 | F | 60 | Amb a 1 | 0 | Cry j 1 | 1,37 | Cup a 1 | 4,15 |
| Italy - CAAM 1029 | F | 60 | Amb a 1 | 0 | Cry j 1 | 3,13 | Cup a 1 | 7,13 |
| Italy - CAAM 1030 | M | 60 | Amb a 1 | 0 | Cry j 1 | 6,25 | Cup a 1 | 12,42 |
| Italy - CAAM 1031 | M | 60 | Amb a 1 | 0 | Cry j 1 | 0 | Cup a 1 | 0,35 |
| Italy - CAAM 1032 | M | 60 | Amb a 1 | 0 | Cry j 1 | 0,33 | Cup a 1 | 2,89 |
| Italy - CAAM 1033 | M | 60 | Amb a 1 | 0 | Cry j 1 | 0 | Cup a 1 | 0,19 |
| Italy - CAAM 1034 | F | 60 | Amb a 1 | 0 | Cry j 1 | 0,23 | Cup a 1 | 5,49 |
| Italy - CAAM 1035 | F | 60 | Amb a 1 | 0 | Cry j 1 | 0 | Cup a 1 | 1,83 |
| Italy - CAAM 1036 | M | 60 | Amb a 1 | 0 | Cry j 1 | 2,65 | Cup a 1 | 6,13 |
| Italy - CAAM 1037 | M | 60 | Amb a 1 | 0 | Cry j 1 | 0 | Cup a 1 | 0,63 |
| Italy - CAAM 1038 | M | 60 | Amb a 1 | 0 | Cry j 1 | 1,68 | Cup a 1 | 12,12 |
| Italy - CAAM 1039 | M | 60 | Amb a 1 | 0 | Cry j 1 | 0 | Cup a 1 | 4 |
| Italy - CAAM 1040 | M | 60 | Amb a 1 | 0 | Cry j 1 | 0 | Cup a 1 | 1,04 |
| Italy - CAAM 1041 | M | 59 | Amb a 1 | 0 | Cry j 1 | 0 | Cup a 1 | 0,95 |
| Italy - CAAM 1042 | M | 59 | Amb a 1 | 0 | Cry j 1 | 0 | Cup a 1 | 1,32 |
| Italy - CAAM 1043 | M | 59 | Amb a 1 | 0 | Cry j 1 | 5,36 | Cup a 1 | 8,7 |
| Italy - CAAM 1044 | F | 59 | Amb a 1 | 0 | Cry j 1 | 0 | Cup a 1 | 1,14 |
| Italy - CAAM 1045 | F | 59 | Amb a 1 | 0 | Cry j 1 | 0,99 | Cup a 1 | 5,65 |
| Italy - CAAM 1046 | M | 59 | Amb a 1 | 0 | Cry j 1 | 0,12 | Cup a 1 | 0 |
| Italy - CAAM 1047 | M | 59 | Amb a 1 | 0 | Cry j 1 | 0,22 | Cup a 1 | 0,44 |
| Italy - CAAM 1048 | F | 59 | Amb a 1 | 0 | Cry j 1 | 0 | Cup a 1 | 0,13 |
| Italy - CAAM 1049 | F | 59 | Amb a 1 | 0 | Cry j 1 | 0,4 | Cup a 1 | 0,52 |
| Italy - CAAM 1050 | F | 59 | Amb a 1 | 0 | Cry j 1 | 0,67 | Cup a 1 | 3,12 |
| Italy - CAAM 1051 | M | 59 | Amb a 1 | 0 | Cry j 1 | 7,77 | Cup a 1 | 0 |
| Italy - CAAM 1052 | M | 59 | Amb a 1 | 0 | Cry j 1 | 1,36 | Cup a 1 | 0,75 |
| Italy - CAAM 1053 | M | 59 | Amb a 1 | 0 | Cry j 1 | 0,51 | Cup a 1 | 0,61 |
| Italy - CAAM 1054 | F | 59 | Amb a 1 | 0 | Cry j 1 | 0,27 | Cup a 1 | 1,1 |
| Italy - CAAM 1055 | F | 59 | Amb a 1 | 0 | Cry j 1 | 3,91 | Cup a 1 | 6,2 |
| Italy - CAAM 1056 | F | 59 | Amb a 1 | 0 | Cry j 1 | 0,68 | Cup a 1 | 0,13 |
| Italy - CAAM 1057 | F | 59 | Amb a 1 | 0 | Cry j 1 | 0,95 | Cup a 1 | 1,15 |
| Italy - CAAM 1058 | F | 59 | Amb a 1 | 0 | Cry j 1 | 0 | Cup a 1 | 0,11 |
| Italy - CAAM 1059 | M | 59 | Amb a 1 | 0 | Cry j 1 | 0,53 | Cup a 1 | 1,82 |
| Italy - CAAM 1060 | F | 59 | Amb a 1 | 0 | Cry j 1 | 0,28 | Cup a 1 | 2,14 |
| Italy - CAAM 1061 | F | 59 | Amb a 1 | 0 | Cry j 1 | 0,23 | Cup a 1 | 1,15 |
| Italy - CAAM 1062 | M | 59 | Amb a 1 | 0 | Cry j 1 | 7,46 | Cup a 1 | 18,63 |
| Italy - CAAM 1063 | M | 59 | Amb a 1 | 0 | Cry j 1 | 0,7 | Cup a 1 | 1,86 |
| Italy - CAAM 1064 | M | 59 | Amb a 1 | 0 | Cry j 1 | 0,65 | Cup a 1 | 3,52 |
| Italy - CAAM 1065 | M | 59 | Amb a 1 | 0 | Cry j 1 | 0,21 | Cup a 1 | 0,53 |
| Italy - CAAM 1066 | F | 59 | Amb a 1 | 0 | Cry j 1 | 0 | Cup a 1 | 0,63 |
| Italy - CAAM 1067 | F | 59 | Amb a 1 | 0 | Cry j 1 | 0 | Cup a 1 | 1,6 |
| Italy - CAAM 1068 | F | 59 | Amb a 1 | 0 | Cry j 1 | 4,26 | Cup a 1 | 11,04 |
| Italy - CAAM 1069 | F | 59 | Amb a 1 | 0 | Cry j 1 | 0 | Cup a 1 | 0,68 |
| Italy - CAAM 1070 | F | 59 | Amb a 1 | 0 | Cry j 1 | 0,96 | Cup a 1 | 6,07 |
| Italy - CAAM 1071 | F | 59 | Amb a 1 | 0 | Cry j 1 | 0 | Cup a 1 | 2,53 |
| Italy - CAAM 1072 | M | 59 | Amb a 1 | 0 | Cry j 1 | 0,6 | Cup a 1 | 2,16 |
| Italy - CAAM 1073 | M | 59 | Amb a 1 | 0 | Cry j 1 | 0,56 | Cup a 1 | 2,16 |
| Italy - CAAM 1074 | F | 59 | Amb a 1 | 0 | Cry j 1 | 0,08 | Cup a 1 | 0 |
| Italy - CAAM 1075 | M | 59 | Amb a 1 | 0 | Cry j 1 | 1,61 | Cup a 1 | 20,44 |
| Italy - CAAM 1076 | F | 59 | Amb a 1 | 0 | Cry j 1 | 4,27 | Cup a 1 | 10,49 |
| Italy - CAAM 1077 | F | 59 | Amb a 1 | 0 | Cry j 1 | 0 | Cup a 1 | 0,62 |
| Italy - CAAM 1078 | M | 59 | Amb a 1 | 0 | Cry j 1 | 1,22 | Cup a 1 | 3,51 |
| Italy - CAAM 1079 | F | 59 | Amb a 1 | 0 | Cry j 1 | 0,17 | Cup a 1 | 1,12 |
| Italy - CAAM 1080 | F | 59 | Amb a 1 | 0 | Cry j 1 | 6,25 | Cup a 1 | 13,33 |
| Italy - CAAM 1081 | M | 59 | Amb a 1 | 0 | Cry j 1 | 2,28 | Cup a 1 | 6,64 |
| Italy - CAAM 1082 | F | 59 | Amb a 1 | 0 | Cry j 1 | 0,19 | Cup a 1 | 10 |
| Italy - CAAM 1083 | M | 59 | Amb a 1 | 0 | Cry j 1 | 2,08 | Cup a 1 | 5,06 |
| Italy - CAAM 1084 | F | 59 | Amb a 1 | 0 | Cry j 1 | 0,12 | Cup a 1 | 0,26 |
| Italy - CAAM 1085 | M | 59 | Amb a 1 | 0 | Cry j 1 | 4,77 | Cup a 1 | 7,77 |
| Italy - CAAM 1086 | M | 59 | Amb a 1 | 0 | Cry j 1 | 0,16 | Cup a 1 | 7,63 |
| Italy - CAAM 1087 | F | 59 | Amb a 1 | 0 | Cry j 1 | 0,44 | Cup a 1 | 1,5 |
| Italy - CAAM 1088 | F | 59 | Amb a 1 | 0 | Cry j 1 | 8,62 | Cup a 1 | 59,66 |
| Italy - CAAM 1089 | M | 59 | Amb a 1 | 0 | Cry j 1 | 4,92 | Cup a 1 | 17,44 |
| Italy - CAAM 1090 | F | 59 | Amb a 1 | 0 | Cry j 1 | 1 | Cup a 1 | 16,56 |
| Italy - CAAM 1091 | M | 59 | Amb a 1 | 0 | Cry j 1 | 0,13 | Cup a 1 | 1,14 |
| Italy - CAAM 1092 | F | 59 | Amb a 1 | 0 | Cry j 1 | 0,85 | Cup a 1 | 24,27 |
| Italy - CAAM 1093 | M | 59 | Amb a 1 | 0 | Cry j 1 | 0,43 | Cup a 1 | 2,43 |
| Italy - CAAM 1094 | F | 59 | Amb a 1 | 0 | Cry j 1 | 0 | Cup a 1 | 2,65 |
| Italy - CAAM 1095 | M | 59 | Amb a 1 | 0 | Cry j 1 | 0 | Cup a 1 | 0,61 |
| Italy - CAAM 1096 | M | 59 | Amb a 1 | 0 | Cry j 1 | 0 | Cup a 1 | 0,57 |
| Italy - CAAM 1097 | M | 59 | Amb a 1 | 0 | Cry j 1 | 0 | Cup a 1 | 1,47 |
| Italy - CAAM 1098 | M | 59 | Amb a 1 | 0 | Cry j 1 | 0 | Cup a 1 | 0,32 |
| Italy - CAAM 1099 | M | 59 | Amb a 1 | 0 | Cry j 1 | 0,41 | Cup a 1 | 4,17 |
| Italy - CAAM 1100 | F | 59 | Amb a 1 | 0 | Cry j 1 | 0,1 | Cup a 1 | 1,27 |
| Italy - CAAM 1101 | F | 58 | Amb a 1 | 0 | Cry j 1 | 0,79 | Cup a 1 | 3,1 |
| Italy - CAAM 1102 | F | 58 | Amb a 1 | 0 | Cry j 1 | 1,05 | Cup a 1 | 0,83 |
| Italy - CAAM 1103 | M | 58 | Amb a 1 | 0 | Cry j 1 | 0,68 | Cup a 1 | 9,31 |
| Italy - CAAM 1104 | M | 58 | Amb a 1 | 0 | Cry j 1 | 7,53 | Cup a 1 | 5,5 |
| Italy - CAAM 1105 | F | 58 | Amb a 1 | 0 | Cry j 1 | 3,16 | Cup a 1 | 1,14 |
| Italy - CAAM 1106 | F | 58 | Amb a 1 | 0 | Cry j 1 | 0 | Cup a 1 | 3,6 |
| Italy - CAAM 1107 | F | 58 | Amb a 1 | 0 | Cry j 1 | 0,68 | Cup a 1 | 0,7 |
| Italy - CAAM 1108 | F | 58 | Amb a 1 | 0 | Cry j 1 | 1,38 | Cup a 1 | 5,46 |
| Italy - CAAM 1109 | M | 58 | Amb a 1 | 0 | Cry j 1 | 0 | Cup a 1 | 0,11 |
| Italy - CAAM 1110 | M | 58 | Amb a 1 | 0 | Cry j 1 | 0,11 | Cup a 1 | 0,45 |
| Italy - CAAM 1111 | F | 58 | Amb a 1 | 0 | Cry j 1 | 2,09 | Cup a 1 | 4,47 |
| Italy - CAAM 1112 | F | 58 | Amb a 1 | 0 | Cry j 1 | 1,8 | Cup a 1 | 18,56 |
| Italy - CAAM 1113 | M | 58 | Amb a 1 | 0 | Cry j 1 | 1,6 | Cup a 1 | 0 |
| Italy - CAAM 1114 | F | 58 | Amb a 1 | 0 | Cry j 1 | 0,98 | Cup a 1 | 6,91 |
| Italy - CAAM 1115 | F | 58 | Amb a 1 | 0 | Cry j 1 | 1,75 | Cup a 1 | 3,09 |
| Italy - CAAM 1116 | M | 58 | Amb a 1 | 0 | Cry j 1 | 29,05 | Cup a 1 | 10,7 |
| Italy - CAAM 1117 | M | 58 | Amb a 1 | 0 | Cry j 1 | 0,5 | Cup a 1 | 0,57 |
| Italy - CAAM 1118 | F | 58 | Amb a 1 | 0 | Cry j 1 | 0,96 | Cup a 1 | 1,34 |
| Italy - CAAM 1119 | F | 58 | Amb a 1 | 0 | Cry j 1 | 0 | Cup a 1 | 0,41 |
| Italy - CAAM 1120 | F | 58 | Amb a 1 | 0 | Cry j 1 | 0 | Cup a 1 | 1,2 |
| Italy - CAAM 1121 | F | 58 | Amb a 1 | 0 | Cry j 1 | 0 | Cup a 1 | 1,93 |
| Italy - CAAM 1122 | F | 58 | Amb a 1 | 0 | Cry j 1 | 0,49 | Cup a 1 | 1,79 |
| Italy - CAAM 1123 | F | 58 | Amb a 1 | 0 | Cry j 1 | 0 | Cup a 1 | 0,71 |
| Italy - CAAM 1124 | F | 58 | Amb a 1 | 0 | Cry j 1 | 3,43 | Cup a 1 | 7,15 |
| Italy - CAAM 1125 | F | 58 | Amb a 1 | 0 | Cry j 1 | 6,8 | Cup a 1 | 15,45 |
| Italy - CAAM 1126 | M | 58 | Amb a 1 | 0 | Cry j 1 | 2,06 | Cup a 1 | 8,1 |
| Italy - CAAM 1127 | F | 58 | Amb a 1 | 0 | Cry j 1 | 0,54 | Cup a 1 | 4,61 |
| Italy - CAAM 1128 | F | 58 | Amb a 1 | 0 | Cry j 1 | 0 | Cup a 1 | 8,85 |
| Italy - CAAM 1129 | M | 58 | Amb a 1 | 0 | Cry j 1 | 0,68 | Cup a 1 | 2,35 |
| Italy - CAAM 1130 | M | 58 | Amb a 1 | 0 | Cry j 1 | 0 | Cup a 1 | 2,93 |
| Italy - CAAM 1131 | F | 58 | Amb a 1 | 0 | Cry j 1 | 1,59 | Cup a 1 | 3,49 |
| Italy - CAAM 1132 | F | 58 | Amb a 1 | 0 | Cry j 1 | 0,5 | Cup a 1 | 2,61 |
| Italy - CAAM 1133 | M | 58 | Amb a 1 | 0 | Cry j 1 | 0 | Cup a 1 | 0,02 |
| Italy - CAAM 1134 | M | 58 | Amb a 1 | 0 | Cry j 1 | 0,16 | Cup a 1 | 0,53 |
| Italy - CAAM 1135 | M | 58 | Amb a 1 | 0 | Cry j 1 | 0,77 | Cup a 1 | 2,32 |
| Italy - CAAM 1136 | M | 58 | Amb a 1 | 0 | Cry j 1 | 0 | Cup a 1 | 0,25 |
| Italy - CAAM 1137 | M | 58 | Amb a 1 | 0 | Cry j 1 | 1,24 | Cup a 1 | 6,28 |
| Italy - CAAM 1138 | M | 58 | Amb a 1 | 0 | Cry j 1 | 3,22 | Cup a 1 | 8,48 |
| Italy - CAAM 1139 | M | 58 | Amb a 1 | 0 | Cry j 1 | 3,19 | Cup a 1 | 13,63 |
| Italy - CAAM 1140 | M | 58 | Amb a 1 | 0 | Cry j 1 | 0,87 | Cup a 1 | 5,48 |
| Italy - CAAM 1141 | M | 58 | Amb a 1 | 0 | Cry j 1 | 0,25 | Cup a 1 | 2,49 |
| Italy - CAAM 1142 | F | 58 | Amb a 1 | 0 | Cry j 1 | 5,54 | Cup a 1 | 16,81 |
| Italy - CAAM 1143 | M | 58 | Amb a 1 | 0 | Cry j 1 | 1,29 | Cup a 1 | 3,14 |
| Italy - CAAM 1144 | M | 58 | Amb a 1 | 0 | Cry j 1 | 5,19 | Cup a 1 | 23,36 |
| Italy - CAAM 1145 | M | 58 | Amb a 1 | 0 | Cry j 1 | 0,25 | Cup a 1 | 2,49 |
| Italy - CAAM 1146 | F | 58 | Amb a 1 | 0 | Cry j 1 | 2,1 | Cup a 1 | 7,9 |
| Italy - CAAM 1147 | M | 58 | Amb a 1 | 0 | Cry j 1 | 0,09 | Cup a 1 | 0,54 |
| Italy - CAAM 1148 | F | 58 | Amb a 1 | 0 | Cry j 1 | 1,4 | Cup a 1 | 6,52 |
| Italy - CAAM 1149 | F | 58 | Amb a 1 | 0 | Cry j 1 | 0,76 | Cup a 1 | 10,51 |
| Italy - CAAM 1150 | F | 58 | Amb a 1 | 0 | Cry j 1 | 3,82 | Cup a 1 | 31,34 |
| Italy - CAAM 1151 | F | 58 | Amb a 1 | 0 | Cry j 1 | 0,1 | Cup a 1 | 1,15 |
| Italy - CAAM 1152 | F | 58 | Amb a 1 | 0 | Cry j 1 | 0,4 | Cup a 1 | 4,49 |
| Italy - CAAM 1153 | F | 58 | Amb a 1 | 0 | Cry j 1 | 2,34 | Cup a 1 | 21,9 |
| Italy - CAAM 1154 | F | 58 | Amb a 1 | 0 | Cry j 1 | 0,99 | Cup a 1 | 10,01 |
| Italy - CAAM 1155 | F | 58 | Amb a 1 | 0 | Cry j 1 | 4,54 | Cup a 1 | 26,48 |
| Italy - CAAM 1156 | F | 58 | Amb a 1 | 0 | Cry j 1 | 1,14 | Cup a 1 | 12,06 |
| Italy - CAAM 1157 | F | 58 | Amb a 1 | 0 | Cry j 1 | 0,54 | Cup a 1 | 5,42 |
| Italy - CAAM 1158 | M | 58 | Amb a 1 | 0 | Cry j 1 | 0,87 | Cup a 1 | 8,79 |
| Italy - CAAM 1159 | F | 58 | Amb a 1 | 0 | Cry j 1 | 0 | Cup a 1 | 0,09 |
| Italy - CAAM 1160 | F | 58 | Amb a 1 | 0 | Cry j 1 | 0,18 | Cup a 1 | 2,14 |
| Italy - CAAM 1161 | F | 58 | Amb a 1 | 0 | Cry j 1 | 0 | Cup a 1 | 19 |
| Italy - CAAM 1162 | M | 58 | Amb a 1 | 0 | Cry j 1 | 2,86 | Cup a 1 | 14,7 |
| Italy - CAAM 1163 | F | 58 | Amb a 1 | 0 | Cry j 1 | 0 | Cup a 1 | 0,13 |
| Italy - CAAM 1164 | F | 58 | Amb a 1 | 0 | Cry j 1 | 0 | Cup a 1 | 0,9 |
| Italy - CAAM 1165 | F | 58 | Amb a 1 | 0 | Cry j 1 | 0,18 | Cup a 1 | 0,52 |
| Italy - CAAM 1166 | M | 58 | Amb a 1 | 0 | Cry j 1 | 0,29 | Cup a 1 | 4,03 |
| Italy - CAAM 1167 | F | 58 | Amb a 1 | 0 | Cry j 1 | 0 | Cup a 1 | 0,28 |
| Italy - CAAM 1168 | F | 57 | Amb a 1 | 0 | Cry j 1 | 8,76 | Cup a 1 | 14,7 |
| Italy - CAAM 1169 | F | 57 | Amb a 1 | 0 | Cry j 1 | 0,04 | Cup a 1 | 2,27 |
| Italy - CAAM 1170 | F | 57 | Amb a 1 | 0 | Cry j 1 | 2,78 | Cup a 1 | 2,91 |
| Italy - CAAM 1171 | M | 57 | Amb a 1 | 0 | Cry j 1 | 0 | Cup a 1 | 3,54 |
| Italy - CAAM 1172 | M | 57 | Amb a 1 | 0 | Cry j 1 | 0 | Cup a 1 | 0,11 |
| Italy - CAAM 1173 | F | 57 | Amb a 1 | 0 | Cry j 1 | 0,45 | Cup a 1 | 0,86 |
| Italy - CAAM 1174 | F | 57 | Amb a 1 | 0 | Cry j 1 | 4,29 | Cup a 1 | 0 |
| Italy - CAAM 1175 | F | 57 | Amb a 1 | 0 | Cry j 1 | 0 | Cup a 1 | 0,76 |
| Italy - CAAM 1176 | F | 57 | Amb a 1 | 0 | Cry j 1 | 12,07 | Cup a 1 | 9,67 |
| Italy - CAAM 1177 | F | 57 | Amb a 1 | 0 | Cry j 1 | 0,81 | Cup a 1 | 5,32 |
| Italy - CAAM 1178 | F | 57 | Amb a 1 | 0 | Cry j 1 | 2 | Cup a 1 | 2,78 |
| Italy - CAAM 1179 | M | 57 | Amb a 1 | 0 | Cry j 1 | 0 | Cup a 1 | 0,15 |
| Italy - CAAM 1180 | F | 57 | Amb a 1 | 0 | Cry j 1 | 4,06 | Cup a 1 | 7,4 |
| Italy - CAAM 1181 | M | 57 | Amb a 1 | 0 | Cry j 1 | 0,61 | Cup a 1 | 1,72 |
| Italy - CAAM 1182 | F | 57 | Amb a 1 | 0 | Cry j 1 | 2,89 | Cup a 1 | 9,13 |
| Italy - CAAM 1183 | F | 57 | Amb a 1 | 0 | Cry j 1 | 2,44 | Cup a 1 | 11,2 |
| Italy - CAAM 1184 | F | 57 | Amb a 1 | 0 | Cry j 1 | 0 | Cup a 1 | 2,62 |
| Italy - CAAM 1185 | F | 57 | Amb a 1 | 0 | Cry j 1 | 0,38 | Cup a 1 | 3,03 |
| Italy - CAAM 1186 | F | 57 | Amb a 1 | 0 | Cry j 1 | 0,22 | Cup a 1 | 2,29 |
| Italy - CAAM 1187 | F | 57 | Amb a 1 | 0 | Cry j 1 | 0,17 | Cup a 1 | 1,25 |
| Italy - CAAM 1188 | F | 57 | Amb a 1 | 0 | Cry j 1 | 0,05 | Cup a 1 | 0,5 |
| Italy - CAAM 1189 | F | 57 | Amb a 1 | 0 | Cry j 1 | 0,15 | Cup a 1 | 0 |
| Italy - CAAM 1190 | M | 57 | Amb a 1 | 0 | Cry j 1 | 0,4 | Cup a 1 | 2,09 |
| Italy - CAAM 1191 | M | 57 | Amb a 1 | 0 | Cry j 1 | 0 | Cup a 1 | 0,22 |
| Italy - CAAM 1192 | F | 57 | Amb a 1 | 0 | Cry j 1 | 0 | Cup a 1 | 0,61 |
| Italy - CAAM 1193 | M | 57 | Amb a 1 | 0 | Cry j 1 | 0 | Cup a 1 | 1,64 |
| Italy - CAAM 1194 | F | 57 | Amb a 1 | 0 | Cry j 1 | 0,4 | Cup a 1 | 10,59 |
| Italy - CAAM 1195 | M | 57 | Amb a 1 | 0 | Cry j 1 | 0,04 | Cup a 1 | 0,17 |
| Italy - CAAM 1196 | F | 57 | Amb a 1 | 0 | Cry j 1 | 0,06 | Cup a 1 | 1,42 |
| Italy - CAAM 1197 | F | 57 | Amb a 1 | 0 | Cry j 1 | 0 | Cup a 1 | 1,95 |
| Italy - CAAM 1198 | F | 57 | Amb a 1 | 0 | Cry j 1 | 0,81 | Cup a 1 | 0 |
| Italy - CAAM 1199 | F | 57 | Amb a 1 | 0 | Cry j 1 | 9,17 | Cup a 1 | 12,91 |
| Italy - CAAM 1200 | M | 57 | Amb a 1 | 0 | Cry j 1 | 0,28 | Cup a 1 | 2,81 |
| Italy - CAAM 1201 | M | 57 | Amb a 1 | 0 | Cry j 1 | 1,55 | Cup a 1 | 16,17 |
| Italy - CAAM 1202 | F | 57 | Amb a 1 | 0 | Cry j 1 | 0,91 | Cup a 1 | 6,15 |
| Italy - CAAM 1203 | F | 57 | Amb a 1 | 0 | Cry j 1 | 0,2 | Cup a 1 | 0,77 |
| Italy - CAAM 1204 | M | 57 | Amb a 1 | 0 | Cry j 1 | 0,97 | Cup a 1 | 6,65 |
| Italy - CAAM 1205 | F | 57 | Amb a 1 | 0 | Cry j 1 | 2,11 | Cup a 1 | 27,18 |
| Italy - CAAM 1206 | F | 57 | Amb a 1 | 0 | Cry j 1 | 0,23 | Cup a 1 | 0,74 |
| Italy - CAAM 1207 | M | 57 | Amb a 1 | 0 | Cry j 1 | 0 | Cup a 1 | 0,08 |
| Italy - CAAM 1208 | F | 57 | Amb a 1 | 0 | Cry j 1 | 1,42 | Cup a 1 | 1,5 |
| Italy - CAAM 1209 | M | 57 | Amb a 1 | 0 | Cry j 1 | 0 | Cup a 1 | 0,52 |
| Italy - CAAM 1210 | F | 57 | Amb a 1 | 0 | Cry j 1 | 22,43 | Cup a 1 | 38,26 |
| Italy - CAAM 1211 | F | 57 | Amb a 1 | 0 | Cry j 1 | 1,59 | Cup a 1 | 5,25 |
| Italy - CAAM 1212 | F | 57 | Amb a 1 | 0 | Cry j 1 | 0,32 | Cup a 1 | 2,08 |
| Italy - CAAM 1213 | F | 57 | Amb a 1 | 0 | Cry j 1 | 0,51 | Cup a 1 | 2,29 |
| Italy - CAAM 1214 | M | 57 | Amb a 1 | 0 | Cry j 1 | 0 | Cup a 1 | 8,45 |
| Italy - CAAM 1215 | M | 57 | Amb a 1 | 0 | Cry j 1 | 0,93 | Cup a 1 | 5,74 |
| Italy - CAAM 1216 | F | 57 | Amb a 1 | 0 | Cry j 1 | 0,1 | Cup a 1 | 2,46 |
| Italy - CAAM 1217 | F | 57 | Amb a 1 | 0 | Cry j 1 | 0 | Cup a 1 | 0,62 |
| Italy - CAAM 1218 | F | 57 | Amb a 1 | 0 | Cry j 1 | 0,19 | Cup a 1 | 2,52 |
| Italy - CAAM 1219 | F | 57 | Amb a 1 | 0 | Cry j 1 | 0 | Cup a 1 | 0,06 |
| Italy - CAAM 1220 | M | 57 | Amb a 1 | 0 | Cry j 1 | 16,56 | Cup a 1 | 54,47 |
| Italy - CAAM 1221 | M | 57 | Amb a 1 | 0 | Cry j 1 | 0 | Cup a 1 | 0,6 |
| Italy - CAAM 1222 | F | 56 | Amb a 1 | 0 | Cry j 1 | 0,14 | Cup a 1 | 0 |
| Italy - CAAM 1223 | F | 56 | Amb a 1 | 0 | Cry j 1 | 0 | Cup a 1 | 1,2 |
| Italy - CAAM 1224 | F | 56 | Amb a 1 | 0 | Cry j 1 | 1,44 | Cup a 1 | 0,94 |
| Italy - CAAM 1225 | F | 56 | Amb a 1 | 0 | Cry j 1 | 0,09 | Cup a 1 | 0,04 |
| Italy - CAAM 1226 | M | 56 | Amb a 1 | 0 | Cry j 1 | 2,11 | Cup a 1 | 5,56 |
| Italy - CAAM 1227 | F | 56 | Amb a 1 | 0 | Cry j 1 | 6,77 | Cup a 1 | 5,45 |
| Italy - CAAM 1228 | M | 56 | Amb a 1 | 0 | Cry j 1 | 2,07 | Cup a 1 | 4,36 |
| Italy - CAAM 1229 | F | 56 | Amb a 1 | 0 | Cry j 1 | 0,46 | Cup a 1 | 3,15 |
| Italy - CAAM 1230 | F | 56 | Amb a 1 | 0 | Cry j 1 | 0,14 | Cup a 1 | 0,27 |
| Italy - CAAM 1231 | F | 56 | Amb a 1 | 0 | Cry j 1 | 9,51 | Cup a 1 | 0,74 |
| Italy - CAAM 1232 | M | 56 | Amb a 1 | 0 | Cry j 1 | 1,64 | Cup a 1 | 1,67 |
| Italy - CAAM 1233 | F | 56 | Amb a 1 | 0 | Cry j 1 | 0,29 | Cup a 1 | 0,07 |
| Italy - CAAM 1234 | F | 56 | Amb a 1 | 0 | Cry j 1 | 3,53 | Cup a 1 | 5,83 |
| Italy - CAAM 1235 | F | 56 | Amb a 1 | 0 | Cry j 1 | 0,91 | Cup a 1 | 8,85 |
| Italy - CAAM 1236 | F | 56 | Amb a 1 | 0 | Cry j 1 | 0,65 | Cup a 1 | 9,1 |
| Italy - CAAM 1237 | F | 56 | Amb a 1 | 0 | Cry j 1 | 1,89 | Cup a 1 | 10,99 |
| Italy - CAAM 1238 | M | 56 | Amb a 1 | 0 | Cry j 1 | 0,49 | Cup a 1 | 1,1 |
| Italy - CAAM 1239 | F | 56 | Amb a 1 | 0 | Cry j 1 | 0 | Cup a 1 | 0,53 |
| Italy - CAAM 1240 | M | 56 | Amb a 1 | 0 | Cry j 1 | 0 | Cup a 1 | 1,47 |
| Italy - CAAM 1241 | M | 56 | Amb a 1 | 0 | Cry j 1 | 0,84 | Cup a 1 | 9,72 |
| Italy - CAAM 1242 | F | 56 | Amb a 1 | 0 | Cry j 1 | 0,53 | Cup a 1 | 5,07 |
| Italy - CAAM 1243 | F | 56 | Amb a 1 | 0 | Cry j 1 | 0,07 | Cup a 1 | 0,16 |
| Italy - CAAM 1244 | M | 56 | Amb a 1 | 0 | Cry j 1 | 0 | Cup a 1 | 0,61 |
| Italy - CAAM 1245 | M | 56 | Amb a 1 | 0 | Cry j 1 | 0,72 | Cup a 1 | 5,42 |
| Italy - CAAM 1246 | M | 56 | Amb a 1 | 0 | Cry j 1 | 0,49 | Cup a 1 | 1,23 |
| Italy - CAAM 1247 | F | 56 | Amb a 1 | 0 | Cry j 1 | 0,42 | Cup a 1 | 1,56 |
| Italy - CAAM 1248 | F | 56 | Amb a 1 | 0 | Cry j 1 | 3,94 | Cup a 1 | 5,44 |
| Italy - CAAM 1249 | F | 56 | Amb a 1 | 0 | Cry j 1 | 0 | Cup a 1 | 0,12 |
| Italy - CAAM 1250 | F | 56 | Amb a 1 | 0 | Cry j 1 | 0 | Cup a 1 | 1,64 |
| Italy - CAAM 1251 | M | 56 | Amb a 1 | 0 | Cry j 1 | 0 | Cup a 1 | 3,62 |
| Italy - CAAM 1252 | F | 56 | Amb a 1 | 0 | Cry j 1 | 0,37 | Cup a 1 | 4,51 |
| Italy - CAAM 1253 | M | 56 | Amb a 1 | 0 | Cry j 1 | 3,16 | Cup a 1 | 13,52 |
| Italy - CAAM 1254 | F | 56 | Amb a 1 | 0 | Cry j 1 | 9,75 | Cup a 1 | 47,3 |
| Italy - CAAM 1255 | M | 56 | Amb a 1 | 0 | Cry j 1 | 5,65 | Cup a 1 | 14,76 |
| Italy - CAAM 1256 | F | 56 | Amb a 1 | 0 | Cry j 1 | 0,31 | Cup a 1 | 3,08 |
| Italy - CAAM 1257 | M | 56 | Amb a 1 | 0 | Cry j 1 | 0 | Cup a 1 | 0,65 |
| Italy - CAAM 1258 | F | 56 | Amb a 1 | 0 | Cry j 1 | 1,11 | Cup a 1 | 1,13 |
| Italy - CAAM 1259 | F | 56 | Amb a 1 | 0 | Cry j 1 | 1,85 | Cup a 1 | 3,99 |
| Italy - CAAM 1260 | M | 56 | Amb a 1 | 0 | Cry j 1 | 1,48 | Cup a 1 | 4,94 |
| Italy - CAAM 1261 | F | 56 | Amb a 1 | 0 | Cry j 1 | 0 | Cup a 1 | 1,23 |
| Italy - CAAM 1262 | M | 56 | Amb a 1 | 0 | Cry j 1 | 0,29 | Cup a 1 | 4,75 |
| Italy - CAAM 1263 | F | 56 | Amb a 1 | 0 | Cry j 1 | 0 | Cup a 1 | 4,72 |
| Italy - CAAM 1264 | M | 56 | Amb a 1 | 0 | Cry j 1 | 1,37 | Cup a 1 | 1,02 |
| Italy - CAAM 1265 | M | 56 | Amb a 1 | 0 | Cry j 1 | 17,25 | Cup a 1 | 17,35 |
| Italy - CAAM 1266 | M | 56 | Amb a 1 | 0 | Cry j 1 | 8,65 | Cup a 1 | 22,48 |
| Italy - CAAM 1267 | F | 56 | Amb a 1 | 0 | Cry j 1 | 2,02 | Cup a 1 | 10 |
| Italy - CAAM 1268 | F | 56 | Amb a 1 | 0 | Cry j 1 | 0 | Cup a 1 | 4,21 |
| Italy - CAAM 1269 | M | 56 | Amb a 1 | 0 | Cry j 1 | 0,23 | Cup a 1 | 1,45 |
| Italy - CAAM 1270 | F | 56 | Amb a 1 | 0 | Cry j 1 | 0,94 | Cup a 1 | 3,59 |
| Italy - CAAM 1271 | F | 56 | Amb a 1 | 0 | Cry j 1 | 2,93 | Cup a 1 | 8,05 |
| Italy - CAAM 1272 | F | 56 | Amb a 1 | 0 | Cry j 1 | 0,79 | Cup a 1 | 2,53 |
| Italy - CAAM 1273 | M | 56 | Amb a 1 | 0 | Cry j 1 | 2,19 | Cup a 1 | 5,74 |
| Italy - CAAM 1274 | M | 56 | Amb a 1 | 0 | Cry j 1 | 0,31 | Cup a 1 | 3,78 |
| Italy - CAAM 1275 | M | 56 | Amb a 1 | 0 | Cry j 1 | 0,5 | Cup a 1 | 13,68 |
| Italy - CAAM 1276 | M | 56 | Amb a 1 | 0 | Cry j 1 | 0,41 | Cup a 1 | 7,19 |
| Italy - CAAM 1277 | M | 56 | Amb a 1 | 0 | Cry j 1 | 0,15 | Cup a 1 | 3,33 |
| Italy - CAAM 1278 | M | 56 | Amb a 1 | 0 | Cry j 1 | 0 | Cup a 1 | 2,93 |
| Italy - CAAM 1279 | F | 56 | Amb a 1 | 0 | Cry j 1 | 3,8 | Cup a 1 | 30 |
| Italy - CAAM 1280 | F | 56 | Amb a 1 | 0 | Cry j 1 | 0,8 | Cup a 1 | 5,52 |
| Italy - CAAM 1281 | F | 56 | Amb a 1 | 0 | Cry j 1 | 0,26 | Cup a 1 | 3,58 |
| Italy - CAAM 1282 | F | 56 | Amb a 1 | 0 | Cry j 1 | 0,25 | Cup a 1 | 2,23 |
| Italy - CAAM 1283 | M | 56 | Amb a 1 | 0 | Cry j 1 | 1,26 | Cup a 1 | 7,44 |
| Italy - CAAM 1284 | F | 56 | Amb a 1 | 0 | Cry j 1 | 0,96 | Cup a 1 | 15,68 |
| Italy - CAAM 1285 | F | 56 | Amb a 1 | 0 | Cry j 1 | 3,8 | Cup a 1 | 43 |
| Italy - CAAM 1286 | F | 56 | Amb a 1 | 0 | Cry j 1 | 1,77 | Cup a 1 | 14,13 |
| Italy - CAAM 1287 | M | 56 | Amb a 1 | 0 | Cry j 1 | 0,33 | Cup a 1 | 1,25 |
| Italy - CAAM 1288 | M | 56 | Amb a 1 | 0 | Cry j 1 | 0,25 | Cup a 1 | 9,84 |
| Italy - CAAM 1289 | M | 55 | Amb a 1 | 0 | Cry j 1 | 1,52 | Cup a 1 | 1,91 |
| Italy - CAAM 1290 | F | 55 | Amb a 1 | 0 | Cry j 1 | 1,24 | Cup a 1 | 1,62 |
| Italy - CAAM 1291 | F | 55 | Amb a 1 | 0 | Cry j 1 | 0 | Cup a 1 | 1,06 |
| Italy - CAAM 1292 | F | 55 | Amb a 1 | 0 | Cry j 1 | 0 | Cup a 1 | 5,04 |
| Italy - CAAM 1293 | F | 55 | Amb a 1 | 0 | Cry j 1 | 2,41 | Cup a 1 | 9,07 |
| Italy - CAAM 1294 | F | 55 | Amb a 1 | 0 | Cry j 1 | 0,6 | Cup a 1 | 16,84 |
| Italy - CAAM 1295 | M | 55 | Amb a 1 | 0 | Cry j 1 | 0 | Cup a 1 | 3,23 |
| Italy - CAAM 1296 | M | 55 | Amb a 1 | 0 | Cry j 1 | 0,39 | Cup a 1 | 4,99 |
| Italy - CAAM 1297 | M | 55 | Amb a 1 | 0 | Cry j 1 | 0,56 | Cup a 1 | 6,16 |
| Italy - CAAM 1298 | F | 55 | Amb a 1 | 0 | Cry j 1 | 0,54 | Cup a 1 | 1,57 |
| Italy - CAAM 1299 | F | 55 | Amb a 1 | 0 | Cry j 1 | 0 | Cup a 1 | 0,63 |
| Italy - CAAM 1300 | F | 55 | Amb a 1 | 0 | Cry j 1 | 1,18 | Cup a 1 | 1,54 |
| Italy - CAAM 1301 | M | 55 | Amb a 1 | 0 | Cry j 1 | 3,61 | Cup a 1 | 1,77 |
| Italy - CAAM 1302 | F | 55 | Amb a 1 | 0 | Cry j 1 | 1,11 | Cup a 1 | 0 |
| Italy - CAAM 1303 | M | 55 | Amb a 1 | 0 | Cry j 1 | 1,31 | Cup a 1 | 0,42 |
| Italy - CAAM 1304 | F | 55 | Amb a 1 | 0 | Cry j 1 | 0,68 | Cup a 1 | 0,87 |
| Italy - CAAM 1305 | M | 55 | Amb a 1 | 0 | Cry j 1 | 17,26 | Cup a 1 | 14,3 |
| Italy - CAAM 1306 | F | 55 | Amb a 1 | 0 | Cry j 1 | 0,29 | Cup a 1 | 0,68 |
| Italy - CAAM 1307 | F | 55 | Amb a 1 | 0 | Cry j 1 | 0 | Cup a 1 | 0,17 |
| Italy - CAAM 1308 | M | 55 | Amb a 1 | 0 | Cry j 1 | 3,15 | Cup a 1 | 6,84 |
| Italy - CAAM 1309 | F | 55 | Amb a 1 | 0 | Cry j 1 | 0,37 | Cup a 1 | 0,29 |
| Italy - CAAM 1310 | M | 55 | Amb a 1 | 0 | Cry j 1 | 0 | Cup a 1 | 0,08 |
| Italy - CAAM 1311 | F | 55 | Amb a 1 | 0 | Cry j 1 | 0,69 | Cup a 1 | 1,8 |
| Italy - CAAM 1312 | F | 55 | Amb a 1 | 0 | Cry j 1 | 1,49 | Cup a 1 | 3,46 |
| Italy - CAAM 1313 | F | 55 | Amb a 1 | 0 | Cry j 1 | 0,12 | Cup a 1 | 0,91 |
| Italy - CAAM 1314 | M | 55 | Amb a 1 | 0 | Cry j 1 | 0,17 | Cup a 1 | 1,39 |
| Italy - CAAM 1315 | M | 55 | Amb a 1 | 0 | Cry j 1 | 0,93 | Cup a 1 | 7,83 |
| Italy - CAAM 1316 | F | 55 | Amb a 1 | 0 | Cry j 1 | 0,14 | Cup a 1 | 2,08 |
| Italy - CAAM 1317 | F | 55 | Amb a 1 | 0 | Cry j 1 | 0,31 | Cup a 1 | 5,25 |
| Italy - CAAM 1318 | F | 55 | Amb a 1 | 0 | Cry j 1 | 9,06 | Cup a 1 | 5,08 |
| Italy - CAAM 1319 | M | 55 | Amb a 1 | 0 | Cry j 1 | 2,84 | Cup a 1 | 10,16 |
| Italy - CAAM 1320 | M | 55 | Amb a 1 | 0 | Cry j 1 | 12,95 | Cup a 1 | 22,02 |
| Italy - CAAM 1321 | F | 55 | Amb a 1 | 0 | Cry j 1 | 0 | Cup a 1 | 2,25 |
| Italy - CAAM 1322 | M | 55 | Amb a 1 | 0 | Cry j 1 | 0 | Cup a 1 | 4,71 |
| Italy - CAAM 1323 | F | 55 | Amb a 1 | 0 | Cry j 1 | 0,32 | Cup a 1 | 4,27 |
| Italy - CAAM 1324 | F | 55 | Amb a 1 | 0 | Cry j 1 | 0,03 | Cup a 1 | 0,17 |
| Italy - CAAM 1325 | M | 55 | Amb a 1 | 0 | Cry j 1 | 5,4 | Cup a 1 | 6,87 |
| Italy - CAAM 1326 | M | 55 | Amb a 1 | 0 | Cry j 1 | 6,39 | Cup a 1 | 4,65 |
| Italy - CAAM 1327 | F | 55 | Amb a 1 | 0 | Cry j 1 | 0 | Cup a 1 | 0,68 |
| Italy - CAAM 1328 | F | 55 | Amb a 1 | 0 | Cry j 1 | 0,39 | Cup a 1 | 2,65 |
| Italy - CAAM 1329 | F | 55 | Amb a 1 | 0 | Cry j 1 | 0 | Cup a 1 | 0,71 |
| Italy - CAAM 1330 | F | 55 | Amb a 1 | 0 | Cry j 1 | 0,11 | Cup a 1 | 2,17 |
| Italy - CAAM 1331 | M | 55 | Amb a 1 | 0 | Cry j 1 | 1,68 | Cup a 1 | 1,69 |
| Italy - CAAM 1332 | F | 55 | Amb a 1 | 0 | Cry j 1 | 4,15 | Cup a 1 | 8,71 |
| Italy - CAAM 1333 | M | 55 | Amb a 1 | 0 | Cry j 1 | 0 | Cup a 1 | 1,13 |
| Italy - CAAM 1334 | F | 55 | Amb a 1 | 0 | Cry j 1 | 1,06 | Cup a 1 | 5,47 |
| Italy - CAAM 1335 | F | 55 | Amb a 1 | 0 | Cry j 1 | 0,08 | Cup a 1 | 0,09 |
| Italy - CAAM 1336 | F | 55 | Amb a 1 | 0 | Cry j 1 | 0,35 | Cup a 1 | 1,43 |
| Italy - CAAM 1337 | M | 55 | Amb a 1 | 0 | Cry j 1 | 0 | Cup a 1 | 0,72 |
| Italy - CAAM 1338 | F | 55 | Amb a 1 | 0 | Cry j 1 | 2,77 | Cup a 1 | 11,59 |
| Italy - CAAM 1339 | F | 55 | Amb a 1 | 0 | Cry j 1 | 4,11 | Cup a 1 | 11,3 |
| Italy - CAAM 1340 | F | 55 | Amb a 1 | 0 | Cry j 1 | 0 | Cup a 1 | 0,03 |
| Italy - CAAM 1341 | M | 55 | Amb a 1 | 0 | Cry j 1 | 0,11 | Cup a 1 | 4,88 |
| Italy - CAAM 1342 | M | 55 | Amb a 1 | 0 | Cry j 1 | 3,67 | Cup a 1 | 23,73 |
| Italy - CAAM 1343 | F | 55 | Amb a 1 | 0 | Cry j 1 | 7,58 | Cup a 1 | 18,75 |
| Italy - CAAM 1344 | F | 55 | Amb a 1 | 0 | Cry j 1 | 0,51 | Cup a 1 | 8,25 |
| Italy - CAAM 1345 | M | 55 | Amb a 1 | 0 | Cry j 1 | 1,4 | Cup a 1 | 13,31 |
| Italy - CAAM 1346 | F | 55 | Amb a 1 | 0 | Cry j 1 | 0 | Cup a 1 | 2,02 |
| Italy - CAAM 1347 | F | 55 | Amb a 1 | 0 | Cry j 1 | 5,96 | Cup a 1 | 15,1 |
| Italy - CAAM 1348 | M | 55 | Amb a 1 | 0 | Cry j 1 | 0 | Cup a 1 | 1,54 |
| Italy - CAAM 1349 | M | 55 | Amb a 1 | 0 | Cry j 1 | 3 | Cup a 1 | 18 |
| Italy - CAAM 1350 | F | 55 | Amb a 1 | 0 | Cry j 1 | 0,55 | Cup a 1 | 3,16 |
| Italy - CAAM 1351 | F | 55 | Amb a 1 | 0 | Cry j 1 | 10,35 | Cup a 1 | 40,85 |
| Italy - CAAM 1352 | M | 55 | Amb a 1 | 0 | Cry j 1 | 0,09 | Cup a 1 | 21,57 |
| Italy - CAAM 1353 | F | 55 | Amb a 1 | 0 | Cry j 1 | 0,16 | Cup a 1 | 2,45 |
| Italy - CAAM 1354 | F | 55 | Amb a 1 | 0 | Cry j 1 | 0 | Cup a 1 | 0,36 |
| Italy - CAAM 1355 | F | 55 | Amb a 1 | 0 | Cry j 1 | 0 | Cup a 1 | 2,21 |
| Italy - CAAM 1356 | F | 55 | Amb a 1 | 0 | Cry j 1 | 0,64 | Cup a 1 | 6,71 |
| Italy - CAAM 1357 | F | 55 | Amb a 1 | 0 | Cry j 1 | 2,58 | Cup a 1 | 7,38 |
| Italy - CAAM 1358 | F | 55 | Amb a 1 | 0 | Cry j 1 | 0 | Cup a 1 | 0,31 |
| Italy - CAAM 1359 | M | 55 | Amb a 1 | 0 | Cry j 1 | 1,68 | Cup a 1 | 40,1 |
| Italy - CAAM 1360 | M | 55 | Amb a 1 | 0 | Cry j 1 | 0,18 | Cup a 1 | 0,18 |
| Italy - CAAM 1361 | M | 55 | Amb a 1 | 0 | Cry j 1 | 0,13 | Cup a 1 | 20,82 |
| Italy - CAAM 1362 | F | 55 | Amb a 1 | 0 | Cry j 1 | 2,44 | Cup a 1 | 23,82 |
| Italy - CAAM 1363 | F | 55 | Amb a 1 | 0 | Cry j 1 | 0 | Cup a 1 | 0,69 |
| Italy - CAAM 1364 | F | 55 | Amb a 1 | 0 | Cry j 1 | 0,05 | Cup a 1 | 3,33 |
| Italy - CAAM 1365 | F | 55 | Amb a 1 | 0 | Cry j 1 | 1,68 | Cup a 1 | 21,04 |
| Italy - CAAM 1366 | M | 55 | Amb a 1 | 0 | Cry j 1 | 0,76 | Cup a 1 | 17,15 |
| Italy - CAAM 1367 | F | 54 | Amb a 1 | 0 | Cry j 1 | 0 | Cup a 1 | 1,82 |
| Italy - CAAM 1368 | F | 54 | Amb a 1 | 0 | Cry j 1 | 0 | Cup a 1 | 1,27 |
| Italy - CAAM 1369 | M | 54 | Amb a 1 | 0 | Cry j 1 | 1,44 | Cup a 1 | 2 |
| Italy - CAAM 1370 | F | 54 | Amb a 1 | 0 | Cry j 1 | 12,59 | Cup a 1 | 29,17 |
| Italy - CAAM 1371 | M | 54 | Amb a 1 | 0 | Cry j 1 | 6,34 | Cup a 1 | 10,58 |
| Italy - CAAM 1372 | M | 54 | Amb a 1 | 0 | Cry j 1 | 0 | Cup a 1 | 4,77 |
| Italy - CAAM 1373 | F | 54 | Amb a 1 | 0 | Cry j 1 | 0 | Cup a 1 | 0,03 |
| Italy - CAAM 1374 | F | 54 | Amb a 1 | 0 | Cry j 1 | 1,28 | Cup a 1 | 2,58 |
| Italy - CAAM 1375 | F | 54 | Amb a 1 | 0 | Cry j 1 | 0,18 | Cup a 1 | 1,86 |
| Italy - CAAM 1376 | F | 54 | Amb a 1 | 0 | Cry j 1 | 14,77 | Cup a 1 | 20,61 |
| Italy - CAAM 1377 | F | 54 | Amb a 1 | 0 | Cry j 1 | 0,17 | Cup a 1 | 0 |
| Italy - CAAM 1378 | M | 54 | Amb a 1 | 0 | Cry j 1 | 2,73 | Cup a 1 | 15,75 |
| Italy - CAAM 1379 | M | 54 | Amb a 1 | 0 | Cry j 1 | 1,04 | Cup a 1 | 1,12 |
| Italy - CAAM 1380 | M | 54 | Amb a 1 | 0 | Cry j 1 | 0,37 | Cup a 1 | 2,3 |
| Italy - CAAM 1381 | M | 54 | Amb a 1 | 0 | Cry j 1 | 11,12 | Cup a 1 | 9,32 |
| Italy - CAAM 1382 | F | 54 | Amb a 1 | 0 | Cry j 1 | 3,87 | Cup a 1 | 8,97 |
| Italy - CAAM 1383 | F | 54 | Amb a 1 | 0 | Cry j 1 | 0 | Cup a 1 | 0,51 |
| Italy - CAAM 1384 | F | 54 | Amb a 1 | 0 | Cry j 1 | 0,47 | Cup a 1 | 0,28 |
| Italy - CAAM 1385 | F | 54 | Amb a 1 | 0 | Cry j 1 | 0,81 | Cup a 1 | 5,33 |
| Italy - CAAM 1386 | F | 54 | Amb a 1 | 0 | Cry j 1 | 0,62 | Cup a 1 | 1,27 |
| Italy - CAAM 1387 | M | 54 | Amb a 1 | 0 | Cry j 1 | 0,23 | Cup a 1 | 0,13 |
| Italy - CAAM 1388 | M | 54 | Amb a 1 | 0 | Cry j 1 | 4,76 | Cup a 1 | 5,77 |
| Italy - CAAM 1389 | F | 54 | Amb a 1 | 0 | Cry j 1 | 0,24 | Cup a 1 | 1,02 |
| Italy - CAAM 1390 | F | 54 | Amb a 1 | 0 | Cry j 1 | 10,04 | Cup a 1 | 7,08 |
| Italy - CAAM 1391 | M | 54 | Amb a 1 | 0 | Cry j 1 | 0 | Cup a 1 | 0,1 |
| Italy - CAAM 1392 | F | 54 | Amb a 1 | 0 | Cry j 1 | 4,93 | Cup a 1 | 11,72 |
| Italy - CAAM 1393 | F | 54 | Amb a 1 | 0 | Cry j 1 | 1,03 | Cup a 1 | 0 |
| Italy - CAAM 1394 | M | 54 | Amb a 1 | 0 | Cry j 1 | 0,69 | Cup a 1 | 1 |
| Italy - CAAM 1395 | F | 54 | Amb a 1 | 0 | Cry j 1 | 2,36 | Cup a 1 | 7,33 |
| Italy - CAAM 1396 | F | 54 | Amb a 1 | 0 | Cry j 1 | 0,53 | Cup a 1 | 1,03 |
| Italy - CAAM 1397 | M | 54 | Amb a 1 | 0 | Cry j 1 | 2,7 | Cup a 1 | 9,2 |
| Italy - CAAM 1398 | F | 54 | Amb a 1 | 0 | Cry j 1 | 0 | Cup a 1 | 0,12 |
| Italy - CAAM 1399 | F | 54 | Amb a 1 | 0 | Cry j 1 | 0,65 | Cup a 1 | 2,71 |
| Italy - CAAM 1400 | M | 54 | Amb a 1 | 0 | Cry j 1 | 0,05 | Cup a 1 | 1,28 |
| Italy - CAAM 1401 | M | 54 | Amb a 1 | 0 | Cry j 1 | 0,15 | Cup a 1 | 0,21 |
| Italy - CAAM 1402 | F | 54 | Amb a 1 | 0 | Cry j 1 | 0,47 | Cup a 1 | 2,14 |
| Italy - CAAM 1403 | F | 54 | Amb a 1 | 0 | Cry j 1 | 0,07 | Cup a 1 | 2 |
| Italy - CAAM 1404 | M | 54 | Amb a 1 | 0 | Cry j 1 | 0,18 | Cup a 1 | 1,26 |
| Italy - CAAM 1405 | M | 54 | Amb a 1 | 0 | Cry j 1 | 0 | Cup a 1 | 0,82 |
| Italy - CAAM 1406 | F | 54 | Amb a 1 | 0 | Cry j 1 | 0,41 | Cup a 1 | 2,77 |
| Italy - CAAM 1407 | F | 54 | Amb a 1 | 0 | Cry j 1 | 1,34 | Cup a 1 | 3,35 |
| Italy - CAAM 1408 | F | 54 | Amb a 1 | 0 | Cry j 1 | 2,65 | Cup a 1 | 10,61 |
| Italy - CAAM 1409 | F | 54 | Amb a 1 | 0 | Cry j 1 | 0,2 | Cup a 1 | 3,95 |
| Italy - CAAM 1410 | M | 54 | Amb a 1 | 0 | Cry j 1 | 5,54 | Cup a 1 | 5,22 |
| Italy - CAAM 1411 | F | 54 | Amb a 1 | 0 | Cry j 1 | 0,25 | Cup a 1 | 2,39 |
| Italy - CAAM 1412 | M | 54 | Amb a 1 | 0 | Cry j 1 | 0,11 | Cup a 1 | 0 |
| Italy - CAAM 1413 | F | 54 | Amb a 1 | 0 | Cry j 1 | 0 | Cup a 1 | 1,87 |
| Italy - CAAM 1414 | M | 54 | Amb a 1 | 0 | Cry j 1 | 0 | Cup a 1 | 0,08 |
| Italy - CAAM 1415 | M | 54 | Amb a 1 | 0 | Cry j 1 | 0,05 | Cup a 1 | 0,34 |
| Italy - CAAM 1416 | F | 54 | Amb a 1 | 0 | Cry j 1 | 0,2 | Cup a 1 | 6,32 |
| Italy - CAAM 1417 | F | 54 | Amb a 1 | 0 | Cry j 1 | 3,37 | Cup a 1 | 6 |
| Italy - CAAM 1418 | M | 54 | Amb a 1 | 0 | Cry j 1 | 0,52 | Cup a 1 | 13,45 |
| Italy - CAAM 1419 | F | 54 | Amb a 1 | 0 | Cry j 1 | 0,16 | Cup a 1 | 8,48 |
| Italy - CAAM 1420 | M | 54 | Amb a 1 | 0 | Cry j 1 | 0 | Cup a 1 | 2,25 |
| Italy - CAAM 1421 | M | 54 | Amb a 1 | 0 | Cry j 1 | 0 | Cup a 1 | 2,02 |
| Italy - CAAM 1422 | M | 54 | Amb a 1 | 0 | Cry j 1 | 2,02 | Cup a 1 | 9,06 |
| Italy - CAAM 1423 | F | 54 | Amb a 1 | 0 | Cry j 1 | 0,05 | Cup a 1 | 1,79 |
| Italy - CAAM 1424 | M | 54 | Amb a 1 | 0 | Cry j 1 | 0 | Cup a 1 | 0,04 |
| Italy - CAAM 1425 | M | 54 | Amb a 1 | 0 | Cry j 1 | 0,68 | Cup a 1 | 11,56 |
| Italy - CAAM 1426 | M | 54 | Amb a 1 | 0 | Cry j 1 | 2,68 | Cup a 1 | 9,97 |
| Italy - CAAM 1427 | M | 54 | Amb a 1 | 0 | Cry j 1 | 1,3 | Cup a 1 | 5,93 |
| Italy - CAAM 1428 | F | 54 | Amb a 1 | 0 | Cry j 1 | 4,66 | Cup a 1 | 24,06 |
| Italy - CAAM 1429 | F | 54 | Amb a 1 | 0 | Cry j 1 | 0 | Cup a 1 | 1,66 |
| Italy - CAAM 1430 | F | 54 | Amb a 1 | 0 | Cry j 1 | 1,91 | Cup a 1 | 6,43 |
| Italy - CAAM 1431 | M | 54 | Amb a 1 | 0 | Cry j 1 | 4,54 | Cup a 1 | 12,39 |
| Italy - CAAM 1432 | F | 54 | Amb a 1 | 0 | Cry j 1 | 7,87 | Cup a 1 | 22,62 |
| Italy - CAAM 1433 | M | 54 | Amb a 1 | 0 | Cry j 1 | 3,27 | Cup a 1 | 7,17 |
| Italy - CAAM 1434 | M | 54 | Amb a 1 | 0 | Cry j 1 | 0,5 | Cup a 1 | 2,78 |
| Italy - CAAM 1435 | M | 54 | Amb a 1 | 0 | Cry j 1 | 0,34 | Cup a 1 | 5,95 |
| Italy - CAAM 1436 | M | 54 | Amb a 1 | 0 | Cry j 1 | 2,16 | Cup a 1 | 3,21 |
| Italy - CAAM 1437 | F | 54 | Amb a 1 | 0 | Cry j 1 | 0,47 | Cup a 1 | 2,7 |
| Italy - CAAM 1438 | F | 54 | Amb a 1 | 0 | Cry j 1 | 0 | Cup a 1 | 8,66 |
| Italy - CAAM 1439 | F | 54 | Amb a 1 | 0 | Cry j 1 | 0,66 | Cup a 1 | 8,13 |
| Italy - CAAM 1440 | F | 54 | Amb a 1 | 0 | Cry j 1 | 1,2 | Cup a 1 | 3,29 |
| Italy - CAAM 1441 | M | 54 | Amb a 1 | 0 | Cry j 1 | 1,3 | Cup a 1 | 11,68 |
| Italy - CAAM 1442 | F | 54 | Amb a 1 | 0 | Cry j 1 | 0 | Cup a 1 | 3,9 |
| Italy - CAAM 1443 | F | 54 | Amb a 1 | 0 | Cry j 1 | 0,41 | Cup a 1 | 13,56 |
| Italy - CAAM 1444 | F | 54 | Amb a 1 | 0 | Cry j 1 | 0 | Cup a 1 | 1,44 |
| Italy - CAAM 1445 | M | 54 | Amb a 1 | 0 | Cry j 1 | 0,4 | Cup a 1 | 4,67 |
| Italy - CAAM 1446 | F | 54 | Amb a 1 | 0 | Cry j 1 | 0,13 | Cup a 1 | 3,46 |
| Italy - CAAM 1447 | F | 54 | Amb a 1 | 0 | Cry j 1 | 0 | Cup a 1 | 0,35 |
| Italy - CAAM 1448 | F | 54 | Amb a 1 | 0 | Cry j 1 | 4,52 | Cup a 1 | 17,31 |
| Italy - CAAM 1449 | F | 54 | Amb a 1 | 0 | Cry j 1 | 0 | Cup a 1 | 3,01 |
| Italy - CAAM 1450 | M | 54 | Amb a 1 | 0 | Cry j 1 | 0 | Cup a 1 | 4,3 |
| Italy - CAAM 1451 | F | 54 | Amb a 1 | 0 | Cry j 1 | 11,97 | Cup a 1 | 9,06 |
| Italy - CAAM 1452 | M | 54 | Amb a 1 | 0 | Cry j 1 | 1,26 | Cup a 1 | 7,59 |
| Italy - CAAM 1453 | F | 53 | Amb a 1 | 0 | Cry j 1 | 0,14 | Cup a 1 | 1,25 |
| Italy - CAAM 1454 | M | 53 | Amb a 1 | 0 | Cry j 1 | 0 | Cup a 1 | 1,91 |
| Italy - CAAM 1455 | M | 53 | Amb a 1 | 0 | Cry j 1 | 1,07 | Cup a 1 | 3,95 |
| Italy - CAAM 1456 | M | 53 | Amb a 1 | 0 | Cry j 1 | 1,25 | Cup a 1 | 0,84 |
| Italy - CAAM 1457 | M | 53 | Amb a 1 | 0 | Cry j 1 | 0 | Cup a 1 | 0,8 |
| Italy - CAAM 1458 | F | 53 | Amb a 1 | 0 | Cry j 1 | 1,74 | Cup a 1 | 2,05 |
| Italy - CAAM 1459 | F | 53 | Amb a 1 | 0 | Cry j 1 | 3,31 | Cup a 1 | 13,94 |
| Italy - CAAM 1460 | M | 53 | Amb a 1 | 0 | Cry j 1 | 0,64 | Cup a 1 | 3,33 |
| Italy - CAAM 1461 | F | 53 | Amb a 1 | 0 | Cry j 1 | 1,18 | Cup a 1 | 4,55 |
| Italy - CAAM 1462 | F | 53 | Amb a 1 | 0 | Cry j 1 | 0,1 | Cup a 1 | 0,88 |
| Italy - CAAM 1463 | F | 53 | Amb a 1 | 0 | Cry j 1 | 0 | Cup a 1 | 2,03 |
| Italy - CAAM 1464 | M | 53 | Amb a 1 | 0 | Cry j 1 | 0,25 | Cup a 1 | 3,07 |
| Italy - CAAM 1465 | F | 53 | Amb a 1 | 0 | Cry j 1 | 1,6 | Cup a 1 | 0 |
| Italy - CAAM 1466 | F | 53 | Amb a 1 | 0 | Cry j 1 | 2,85 | Cup a 1 | 5,65 |
| Italy - CAAM 1467 | F | 53 | Amb a 1 | 0 | Cry j 1 | 12,2 | Cup a 1 | 0 |
| Italy - CAAM 1468 | F | 53 | Amb a 1 | 0 | Cry j 1 | 23,24 | Cup a 1 | 0,72 |
| Italy - CAAM 1469 | F | 53 | Amb a 1 | 0 | Cry j 1 | 0,2 | Cup a 1 | 0,24 |
| Italy - CAAM 1470 | F | 53 | Amb a 1 | 0 | Cry j 1 | 1,65 | Cup a 1 | 1,79 |
| Italy - CAAM 1471 | F | 53 | Amb a 1 | 0 | Cry j 1 | 0,47 | Cup a 1 | 1,49 |
| Italy - CAAM 1472 | F | 53 | Amb a 1 | 0 | Cry j 1 | 0,63 | Cup a 1 | 1,31 |
| Italy - CAAM 1473 | M | 53 | Amb a 1 | 0 | Cry j 1 | 1,2 | Cup a 1 | 22,5 |
| Italy - CAAM 1474 | F | 53 | Amb a 1 | 0 | Cry j 1 | 0,64 | Cup a 1 | 0,54 |
| Italy - CAAM 1475 | F | 53 | Amb a 1 | 0 | Cry j 1 | 0 | Cup a 1 | 1,38 |
| Italy - CAAM 1476 | F | 53 | Amb a 1 | 0 | Cry j 1 | 11,57 | Cup a 1 | 27,47 |
| Italy - CAAM 1477 | M | 53 | Amb a 1 | 0 | Cry j 1 | 0,84 | Cup a 1 | 6,34 |
| Italy - CAAM 1478 | F | 53 | Amb a 1 | 0 | Cry j 1 | 0 | Cup a 1 | 0,32 |
| Italy - CAAM 1479 | F | 53 | Amb a 1 | 0 | Cry j 1 | 0 | Cup a 1 | 4,3 |
| Italy - CAAM 1480 | F | 53 | Amb a 1 | 0 | Cry j 1 | 0 | Cup a 1 | 1,72 |
| Italy - CAAM 1481 | F | 53 | Amb a 1 | 0 | Cry j 1 | 0 | Cup a 1 | 0,53 |
| Italy - CAAM 1482 | F | 53 | Amb a 1 | 0 | Cry j 1 | 3,73 | Cup a 1 | 4,58 |
| Italy - CAAM 1483 | M | 53 | Amb a 1 | 0 | Cry j 1 | 0,12 | Cup a 1 | 1,37 |
| Italy - CAAM 1484 | M | 53 | Amb a 1 | 0 | Cry j 1 | 0 | Cup a 1 | 0,18 |
| Italy - CAAM 1485 | M | 53 | Amb a 1 | 0 | Cry j 1 | 1,45 | Cup a 1 | 2,82 |
| Italy - CAAM 1486 | M | 53 | Amb a 1 | 0 | Cry j 1 | 0,72 | Cup a 1 | 4,48 |
| Italy - CAAM 1487 | F | 53 | Amb a 1 | 0 | Cry j 1 | 0 | Cup a 1 | 1,27 |
| Italy - CAAM 1488 | F | 53 | Amb a 1 | 0 | Cry j 1 | 0,36 | Cup a 1 | 8,51 |
| Italy - CAAM 1489 | F | 53 | Amb a 1 | 0 | Cry j 1 | 0,37 | Cup a 1 | 0,23 |
| Italy - CAAM 1490 | M | 53 | Amb a 1 | 0 | Cry j 1 | 0,19 | Cup a 1 | 2,34 |
| Italy - CAAM 1491 | M | 53 | Amb a 1 | 0 | Cry j 1 | 1,34 | Cup a 1 | 10,76 |
| Italy - CAAM 1492 | M | 53 | Amb a 1 | 0 | Cry j 1 | 0 | Cup a 1 | 2,27 |
| Italy - CAAM 1493 | F | 53 | Amb a 1 | 0 | Cry j 1 | 0,19 | Cup a 1 | 3,25 |
| Italy - CAAM 1494 | M | 53 | Amb a 1 | 0 | Cry j 1 | 1,32 | Cup a 1 | 11,67 |
| Italy - CAAM 1495 | F | 53 | Amb a 1 | 0 | Cry j 1 | 0,47 | Cup a 1 | 2,04 |
| Italy - CAAM 1496 | F | 53 | Amb a 1 | 0 | Cry j 1 | 2,9 | Cup a 1 | 23,22 |
| Italy - CAAM 1497 | F | 53 | Amb a 1 | 0 | Cry j 1 | 1,12 | Cup a 1 | 1,76 |
| Italy - CAAM 1498 | M | 53 | Amb a 1 | 0 | Cry j 1 | 0,12 | Cup a 1 | 0,06 |
| Italy - CAAM 1499 | F | 53 | Amb a 1 | 0 | Cry j 1 | 0,11 | Cup a 1 | 0,09 |
| Italy - CAAM 1500 | F | 53 | Amb a 1 | 0 | Cry j 1 | 0,22 | Cup a 1 | 1,98 |
| Italy - CAAM 1501 | M | 53 | Amb a 1 | 0 | Cry j 1 | 0,36 | Cup a 1 | 2,2 |
| Italy - CAAM 1502 | M | 53 | Amb a 1 | 0 | Cry j 1 | 0 | Cup a 1 | 0,05 |
| Italy - CAAM 1503 | F | 53 | Amb a 1 | 0 | Cry j 1 | 0 | Cup a 1 | 11,94 |
| Italy - CAAM 1504 | F | 53 | Amb a 1 | 0 | Cry j 1 | 0,06 | Cup a 1 | 2,7 |
| Italy - CAAM 1505 | F | 53 | Amb a 1 | 0 | Cry j 1 | 6,94 | Cup a 1 | 17,19 |
| Italy - CAAM 1506 | F | 53 | Amb a 1 | 0 | Cry j 1 | 0,98 | Cup a 1 | 9,91 |
| Italy - CAAM 1507 | M | 53 | Amb a 1 | 0 | Cry j 1 | 5,66 | Cup a 1 | 6,12 |
| Italy - CAAM 1508 | F | 53 | Amb a 1 | 0 | Cry j 1 | 7,37 | Cup a 1 | 23,62 |
| Italy - CAAM 1509 | M | 53 | Amb a 1 | 0 | Cry j 1 | 1,11 | Cup a 1 | 3,51 |
| Italy - CAAM 1510 | F | 53 | Amb a 1 | 0 | Cry j 1 | 0,09 | Cup a 1 | 0,76 |
| Italy - CAAM 1511 | F | 53 | Amb a 1 | 0 | Cry j 1 | 0,66 | Cup a 1 | 4,69 |
| Italy - CAAM 1512 | F | 53 | Amb a 1 | 0 | Cry j 1 | 0 | Cup a 1 | 0,57 |
| Italy - CAAM 1513 | M | 53 | Amb a 1 | 0 | Cry j 1 | 0,2 | Cup a 1 | 3,02 |
| Italy - CAAM 1514 | F | 53 | Amb a 1 | 0 | Cry j 1 | 0,61 | Cup a 1 | 5,56 |
| Italy - CAAM 1515 | F | 53 | Amb a 1 | 0 | Cry j 1 | 2,15 | Cup a 1 | 12,8 |
| Italy - CAAM 1516 | F | 53 | Amb a 1 | 0 | Cry j 1 | 0,24 | Cup a 1 | 4,87 |
| Italy - CAAM 1517 | F | 53 | Amb a 1 | 0 | Cry j 1 | 0 | Cup a 1 | 0,29 |
| Italy - CAAM 1518 | M | 53 | Amb a 1 | 0 | Cry j 1 | 0,97 | Cup a 1 | 1,67 |
| Italy - CAAM 1519 | F | 53 | Amb a 1 | 0 | Cry j 1 | 0 | Cup a 1 | 4,36 |
| Italy - CAAM 1520 | F | 53 | Amb a 1 | 0 | Cry j 1 | 0,7 | Cup a 1 | 21 |
| Italy - CAAM 1521 | F | 53 | Amb a 1 | 0 | Cry j 1 | 0 | Cup a 1 | 2,4 |
| Italy - CAAM 1522 | F | 53 | Amb a 1 | 0 | Cry j 1 | 0,13 | Cup a 1 | 2,85 |
| Italy - CAAM 1523 | F | 53 | Amb a 1 | 0 | Cry j 1 | 0,61 | Cup a 1 | 14,75 |
| Italy - CAAM 1524 | M | 53 | Amb a 1 | 0 | Cry j 1 | 0 | Cup a 1 | 0,19 |
| Italy - CAAM 1525 | M | 52 | Amb a 1 | 0 | Cry j 1 | 15,18 | Cup a 1 | 7,05 |
| Italy - CAAM 1526 | F | 52 | Amb a 1 | 0 | Cry j 1 | 3,74 | Cup a 1 | 34,49 |
| Italy - CAAM 1527 | F | 52 | Amb a 1 | 0 | Cry j 1 | 1 | Cup a 1 | 3,2 |
| Italy - CAAM 1528 | F | 52 | Amb a 1 | 0 | Cry j 1 | 2,41 | Cup a 1 | 5,3 |
| Italy - CAAM 1529 | F | 52 | Amb a 1 | 0 | Cry j 1 | 2,1 | Cup a 1 | 10,32 |
| Italy - CAAM 1530 | F | 52 | Amb a 1 | 0 | Cry j 1 | 6,15 | Cup a 1 | 6,78 |
| Italy - CAAM 1531 | F | 52 | Amb a 1 | 0 | Cry j 1 | 0 | Cup a 1 | 2,08 |
| Italy - CAAM 1532 | M | 52 | Amb a 1 | 0 | Cry j 1 | 0 | Cup a 1 | 0,17 |
| Italy - CAAM 1533 | F | 52 | Amb a 1 | 0 | Cry j 1 | 5,05 | Cup a 1 | 9,8 |
| Italy - CAAM 1534 | F | 52 | Amb a 1 | 0 | Cry j 1 | 0,16 | Cup a 1 | 2,01 |
| Italy - CAAM 1535 | F | 52 | Amb a 1 | 0 | Cry j 1 | 0 | Cup a 1 | 0,2 |
| Italy - CAAM 1536 | F | 52 | Amb a 1 | 0 | Cry j 1 | 0 | Cup a 1 | 0,57 |
| Italy - CAAM 1537 | F | 52 | Amb a 1 | 0 | Cry j 1 | 0,02 | Cup a 1 | 0,32 |
| Italy - CAAM 1538 | F | 52 | Amb a 1 | 0 | Cry j 1 | 3,7 | Cup a 1 | 9,94 |
| Italy - CAAM 1539 | F | 52 | Amb a 1 | 0 | Cry j 1 | 0,03 | Cup a 1 | 0,25 |
| Italy - CAAM 1540 | M | 52 | Amb a 1 | 0 | Cry j 1 | 0 | Cup a 1 | 2,27 |
| Italy - CAAM 1541 | M | 52 | Amb a 1 | 0 | Cry j 1 | 0,69 | Cup a 1 | 1,14 |
| Italy - CAAM 1542 | M | 52 | Amb a 1 | 0 | Cry j 1 | 0,88 | Cup a 1 | 7,36 |
| Italy - CAAM 1543 | M | 52 | Amb a 1 | 0 | Cry j 1 | 0 | Cup a 1 | 0,39 |
| Italy - CAAM 1544 | F | 52 | Amb a 1 | 0 | Cry j 1 | 0,62 | Cup a 1 | 2,99 |
| Italy - CAAM 1545 | M | 52 | Amb a 1 | 0 | Cry j 1 | 0 | Cup a 1 | 0,66 |
| Italy - CAAM 1546 | M | 52 | Amb a 1 | 0 | Cry j 1 | 0 | Cup a 1 | 0,14 |
| Italy - CAAM 1547 | F | 52 | Amb a 1 | 0 | Cry j 1 | 0,7 | Cup a 1 | 3,21 |
| Italy - CAAM 1548 | M | 52 | Amb a 1 | 0 | Cry j 1 | 3,4 | Cup a 1 | 8,86 |
| Italy - CAAM 1549 | M | 52 | Amb a 1 | 0 | Cry j 1 | 3 | Cup a 1 | 5,95 |
| Italy - CAAM 1550 | F | 52 | Amb a 1 | 0 | Cry j 1 | 17,81 | Cup a 1 | 8,02 |
| Italy - CAAM 1551 | F | 52 | Amb a 1 | 0 | Cry j 1 | 0 | Cup a 1 | 1,14 |
| Italy - CAAM 1552 | F | 52 | Amb a 1 | 0 | Cry j 1 | 4 | Cup a 1 | 6,86 |
| Italy - CAAM 1553 | M | 52 | Amb a 1 | 0 | Cry j 1 | 0 | Cup a 1 | 0,44 |
| Italy - CAAM 1554 | M | 52 | Amb a 1 | 0 | Cry j 1 | 6,06 | Cup a 1 | 19,12 |
| Italy - CAAM 1555 | F | 52 | Amb a 1 | 0 | Cry j 1 | 0 | Cup a 1 | 0,5 |
| Italy - CAAM 1556 | M | 52 | Amb a 1 | 0 | Cry j 1 | 0,31 | Cup a 1 | 0,34 |
| Italy - CAAM 1557 | F | 52 | Amb a 1 | 0 | Cry j 1 | 0 | Cup a 1 | 0,68 |
| Italy - CAAM 1558 | F | 52 | Amb a 1 | 0 | Cry j 1 | 0 | Cup a 1 | 2,59 |
| Italy - CAAM 1559 | F | 52 | Amb a 1 | 0 | Cry j 1 | 0,5 | Cup a 1 | 1,4 |
| Italy - CAAM 1560 | M | 52 | Amb a 1 | 0 | Cry j 1 | 0 | Cup a 1 | 0,99 |
| Italy - CAAM 1561 | F | 52 | Amb a 1 | 0 | Cry j 1 | 1,72 | Cup a 1 | 8,72 |
| Italy - CAAM 1562 | F | 52 | Amb a 1 | 0 | Cry j 1 | 0,32 | Cup a 1 | 0,95 |
| Italy - CAAM 1563 | F | 52 | Amb a 1 | 0 | Cry j 1 | 0,17 | Cup a 1 | 1,27 |
| Italy - CAAM 1564 | M | 52 | Amb a 1 | 0 | Cry j 1 | 0 | Cup a 1 | 0,05 |
| Italy - CAAM 1565 | M | 52 | Amb a 1 | 0 | Cry j 1 | 0 | Cup a 1 | 0,16 |
| Italy - CAAM 1566 | F | 52 | Amb a 1 | 0 | Cry j 1 | 0,35 | Cup a 1 | 0,76 |
| Italy - CAAM 1567 | M | 52 | Amb a 1 | 0 | Cry j 1 | 1,15 | Cup a 1 | 4,52 |
| Italy - CAAM 1568 | F | 52 | Amb a 1 | 0 | Cry j 1 | 1,15 | Cup a 1 | 4,78 |
| Italy - CAAM 1569 | F | 52 | Amb a 1 | 0 | Cry j 1 | 0,19 | Cup a 1 | 0,37 |
| Italy - CAAM 1570 | F | 52 | Amb a 1 | 0 | Cry j 1 | 0 | Cup a 1 | 0,55 |
| Italy - CAAM 1571 | F | 52 | Amb a 1 | 0 | Cry j 1 | 0 | Cup a 1 | 1,11 |
| Italy - CAAM 1572 | M | 52 | Amb a 1 | 0 | Cry j 1 | 0 | Cup a 1 | 0,47 |
| Italy - CAAM 1573 | M | 52 | Amb a 1 | 0 | Cry j 1 | 0 | Cup a 1 | 0,31 |
| Italy - CAAM 1574 | F | 52 | Amb a 1 | 0 | Cry j 1 | 0 | Cup a 1 | 2,13 |
| Italy - CAAM 1575 | F | 52 | Amb a 1 | 0 | Cry j 1 | 1,98 | Cup a 1 | 3,48 |
| Italy - CAAM 1576 | M | 52 | Amb a 1 | 0 | Cry j 1 | 4,48 | Cup a 1 | 17,45 |
| Italy - CAAM 1577 | F | 52 | Amb a 1 | 0 | Cry j 1 | 0,54 | Cup a 1 | 2,34 |
| Italy - CAAM 1578 | F | 52 | Amb a 1 | 0 | Cry j 1 | 0,15 | Cup a 1 | 3,51 |
| Italy - CAAM 1579 | F | 52 | Amb a 1 | 0 | Cry j 1 | 2,77 | Cup a 1 | 22,74 |
| Italy - CAAM 1580 | M | 52 | Amb a 1 | 0 | Cry j 1 | 3,26 | Cup a 1 | 5,07 |
| Italy - CAAM 1581 | F | 52 | Amb a 1 | 0 | Cry j 1 | 0,23 | Cup a 1 | 0 |
| Italy - CAAM 1582 | F | 52 | Amb a 1 | 0 | Cry j 1 | 0,96 | Cup a 1 | 5,49 |
| Italy - CAAM 1583 | F | 52 | Amb a 1 | 0 | Cry j 1 | 0,1 | Cup a 1 | 0,03 |
| Italy - CAAM 1584 | M | 52 | Amb a 1 | 0 | Cry j 1 | 0,1 | Cup a 1 | 1,88 |
| Italy - CAAM 1585 | M | 52 | Amb a 1 | 0 | Cry j 1 | 0,19 | Cup a 1 | 1,77 |
| Italy - CAAM 1586 | F | 52 | Amb a 1 | 0 | Cry j 1 | 0 | Cup a 1 | 0,28 |
| Italy - CAAM 1587 | M | 52 | Amb a 1 | 0 | Cry j 1 | 0,42 | Cup a 1 | 6,48 |
| Italy - CAAM 1588 | F | 52 | Amb a 1 | 0 | Cry j 1 | 0,46 | Cup a 1 | 1,05 |
| Italy - CAAM 1589 | F | 52 | Amb a 1 | 0 | Cry j 1 | 1,24 | Cup a 1 | 2,24 |
| Italy - CAAM 1590 | F | 52 | Amb a 1 | 0 | Cry j 1 | 0,17 | Cup a 1 | 19,72 |
| Italy - CAAM 1591 | F | 52 | Amb a 1 | 0 | Cry j 1 | 4,95 | Cup a 1 | 36,88 |
| Italy - CAAM 1592 | F | 52 | Amb a 1 | 0 | Cry j 1 | 4,66 | Cup a 1 | 49,71 |
| Italy - CAAM 1593 | F | 52 | Amb a 1 | 0 | Cry j 1 | 0 | Cup a 1 | 0,51 |
| Italy - CAAM 1594 | M | 52 | Amb a 1 | 0 | Cry j 1 | 1,03 | Cup a 1 | 2,54 |
| Italy - CAAM 1595 | F | 52 | Amb a 1 | 0 | Cry j 1 | 0 | Cup a 1 | 0,41 |
| Italy - CAAM 1596 | F | 52 | Amb a 1 | 0 | Cry j 1 | 3 | Cup a 1 | 4,49 |
| Italy - CAAM 1597 | M | 52 | Amb a 1 | 0 | Cry j 1 | 0,16 | Cup a 1 | 3,25 |
| Italy - CAAM 1598 | F | 52 | Amb a 1 | 0 | Cry j 1 | 1,02 | Cup a 1 | 34,05 |
| Italy - CAAM 1599 | F | 52 | Amb a 1 | 0 | Cry j 1 | 0,22 | Cup a 1 | 1,05 |
| Italy - CAAM 1600 | M | 52 | Amb a 1 | 0 | Cry j 1 | 0 | Cup a 1 | 9,52 |
| Italy - CAAM 1601 | F | 52 | Amb a 1 | 0 | Cry j 1 | 5,05 | Cup a 1 | 20,07 |
| Italy - CAAM 1602 | F | 52 | Amb a 1 | 0 | Cry j 1 | 0 | Cup a 1 | 1,85 |
| Italy - CAAM 1603 | F | 52 | Amb a 1 | 0 | Cry j 1 | 0 | Cup a 1 | 0,53 |
| Italy - CAAM 1604 | F | 52 | Amb a 1 | 0 | Cry j 1 | 1,46 | Cup a 1 | 9,51 |
| Italy - CAAM 1605 | F | 52 | Amb a 1 | 0 | Cry j 1 | 0,47 | Cup a 1 | 3,28 |
| Italy - CAAM 1606 | M | 52 | Amb a 1 | 0 | Cry j 1 | 2,41 | Cup a 1 | 48,21 |
| Italy - CAAM 1607 | F | 52 | Amb a 1 | 0 | Cry j 1 | 0,31 | Cup a 1 | 6,39 |
| Italy - CAAM 1608 | F | 52 | Amb a 1 | 0 | Cry j 1 | 0,14 | Cup a 1 | 5,93 |
| Italy - CAAM 1609 | M | 52 | Amb a 1 | 0 | Cry j 1 | 0,55 | Cup a 1 | 6,34 |
| Italy - CAAM 1610 | F | 52 | Amb a 1 | 0 | Cry j 1 | 0,27 | Cup a 1 | 1,55 |
| Italy - CAAM 1611 | M | 52 | Amb a 1 | 0 | Cry j 1 | 0,96 | Cup a 1 | 12,65 |
| Italy - CAAM 1612 | M | 52 | Amb a 1 | 0 | Cry j 1 | 0 | Cup a 1 | 0,12 |
| Italy - CAAM 1613 | M | 52 | Amb a 1 | 0 | Cry j 1 | 0 | Cup a 1 | 1,4 |
| Italy - CAAM 1614 | M | 52 | Amb a 1 | 0 | Cry j 1 | 0 | Cup a 1 | 5,3 |
| Italy - CAAM 1615 | F | 52 | Amb a 1 | 0 | Cry j 1 | 0 | Cup a 1 | 0,38 |
| Italy - CAAM 1616 | M | 52 | Amb a 1 | 0 | Cry j 1 | 0 | Cup a 1 | 2,68 |
| Italy - CAAM 1617 | F | 52 | Amb a 1 | 0 | Cry j 1 | 0 | Cup a 1 | 2,34 |
| Italy - CAAM 1618 | F | 51 | Amb a 1 | 0 | Cry j 1 | 13,05 | Cup a 1 | 26,64 |
| Italy - CAAM 1619 | M | 51 | Amb a 1 | 0 | Cry j 1 | 0 | Cup a 1 | 0,38 |
| Italy - CAAM 1620 | M | 51 | Amb a 1 | 0 | Cry j 1 | 1,97 | Cup a 1 | 1,48 |
| Italy - CAAM 1621 | F | 51 | Amb a 1 | 0 | Cry j 1 | 0 | Cup a 1 | 0,14 |
| Italy - CAAM 1622 | F | 51 | Amb a 1 | 0 | Cry j 1 | 5,03 | Cup a 1 | 12,01 |
| Italy - CAAM 1623 | F | 51 | Amb a 1 | 0 | Cry j 1 | 0 | Cup a 1 | 2,97 |
| Italy - CAAM 1624 | F | 51 | Amb a 1 | 0 | Cry j 1 | 0 | Cup a 1 | 0,26 |
| Italy - CAAM 1625 | F | 51 | Amb a 1 | 0 | Cry j 1 | 0 | Cup a 1 | 1,79 |
| Italy - CAAM 1626 | F | 51 | Amb a 1 | 0 | Cry j 1 | 0,12 | Cup a 1 | 0,28 |
| Italy - CAAM 1627 | F | 51 | Amb a 1 | 0 | Cry j 1 | 0,84 | Cup a 1 | 2,91 |
| Italy - CAAM 1628 | F | 51 | Amb a 1 | 0 | Cry j 1 | 0,44 | Cup a 1 | 0,62 |
| Italy - CAAM 1629 | M | 51 | Amb a 1 | 0 | Cry j 1 | 0,72 | Cup a 1 | 3,98 |
| Italy - CAAM 1630 | M | 51 | Amb a 1 | 0 | Cry j 1 | 1,79 | Cup a 1 | 7,15 |
| Italy - CAAM 1631 | F | 51 | Amb a 1 | 0 | Cry j 1 | 1,16 | Cup a 1 | 5,63 |
| Italy - CAAM 1632 | F | 51 | Amb a 1 | 0 | Cry j 1 | 0,97 | Cup a 1 | 1,89 |
| Italy - CAAM 1633 | F | 51 | Amb a 1 | 0 | Cry j 1 | 0 | Cup a 1 | 0,69 |
| Italy - CAAM 1634 | F | 51 | Amb a 1 | 0 | Cry j 1 | 0,45 | Cup a 1 | 2,04 |
| Italy - CAAM 1635 | M | 51 | Amb a 1 | 0 | Cry j 1 | 0 | Cup a 1 | 0,73 |
| Italy - CAAM 1636 | F | 51 | Amb a 1 | 0 | Cry j 1 | 2,42 | Cup a 1 | 2,05 |
| Italy - CAAM 1637 | M | 51 | Amb a 1 | 0 | Cry j 1 | 0 | Cup a 1 | 0,89 |
| Italy - CAAM 1638 | F | 51 | Amb a 1 | 0 | Cry j 1 | 0,28 | Cup a 1 | 1,97 |
| Italy - CAAM 1639 | M | 51 | Amb a 1 | 0 | Cry j 1 | 0 | Cup a 1 | 2,42 |
| Italy - CAAM 1640 | F | 51 | Amb a 1 | 0 | Cry j 1 | 9,56 | Cup a 1 | 1,09 |
| Italy - CAAM 1641 | F | 51 | Amb a 1 | 0 | Cry j 1 | 0 | Cup a 1 | 0,06 |
| Italy - CAAM 1642 | M | 51 | Amb a 1 | 0 | Cry j 1 | 6,26 | Cup a 1 | 6,13 |
| Italy - CAAM 1643 | F | 51 | Amb a 1 | 0 | Cry j 1 | 1,23 | Cup a 1 | 1,5 |
| Italy - CAAM 1644 | F | 51 | Amb a 1 | 0 | Cry j 1 | 1,1 | Cup a 1 | 1,91 |
| Italy - CAAM 1645 | F | 51 | Amb a 1 | 0 | Cry j 1 | 0,29 | Cup a 1 | 0 |
| Italy - CAAM 1646 | F | 51 | Amb a 1 | 0 | Cry j 1 | 0,37 | Cup a 1 | 1,62 |
| Italy - CAAM 1647 | F | 51 | Amb a 1 | 0 | Cry j 1 | 30,16 | Cup a 1 | 22,05 |
| Italy - CAAM 1648 | F | 51 | Amb a 1 | 0 | Cry j 1 | 1,04 | Cup a 1 | 2 |
| Italy - CAAM 1649 | F | 51 | Amb a 1 | 0 | Cry j 1 | 0 | Cup a 1 | 0,9 |
| Italy - CAAM 1650 | F | 51 | Amb a 1 | 0 | Cry j 1 | 0 | Cup a 1 | 1,4 |
| Italy - CAAM 1651 | M | 51 | Amb a 1 | 0 | Cry j 1 | 0,26 | Cup a 1 | 0,35 |
| Italy - CAAM 1652 | M | 51 | Amb a 1 | 0 | Cry j 1 | 1,76 | Cup a 1 | 8,33 |
| Italy - CAAM 1653 | M | 51 | Amb a 1 | 0 | Cry j 1 | 1,73 | Cup a 1 | 4,08 |
| Italy - CAAM 1654 | F | 51 | Amb a 1 | 0 | Cry j 1 | 0 | Cup a 1 | 2,37 |
| Italy - CAAM 1655 | M | 51 | Amb a 1 | 0 | Cry j 1 | 0,43 | Cup a 1 | 1,04 |
| Italy - CAAM 1656 | M | 51 | Amb a 1 | 0 | Cry j 1 | 0,04 | Cup a 1 | 1,83 |
| Italy - CAAM 1657 | F | 51 | Amb a 1 | 0 | Cry j 1 | 0,06 | Cup a 1 | 3,46 |
| Italy - CAAM 1658 | M | 51 | Amb a 1 | 0 | Cry j 1 | 0,18 | Cup a 1 | 3,96 |
| Italy - CAAM 1659 | M | 51 | Amb a 1 | 0 | Cry j 1 | 1 | Cup a 1 | 5 |
| Italy - CAAM 1660 | F | 51 | Amb a 1 | 0 | Cry j 1 | 0,29 | Cup a 1 | 0,18 |
| Italy - CAAM 1661 | F | 51 | Amb a 1 | 0 | Cry j 1 | 1,81 | Cup a 1 | 14,75 |
| Italy - CAAM 1662 | F | 51 | Amb a 1 | 0 | Cry j 1 | 1,89 | Cup a 1 | 14,7 |
| Italy - CAAM 1663 | F | 51 | Amb a 1 | 0 | Cry j 1 | 4,07 | Cup a 1 | 22,25 |
| Italy - CAAM 1664 | M | 51 | Amb a 1 | 0 | Cry j 1 | 0,04 | Cup a 1 | 0,06 |
| Italy - CAAM 1665 | F | 51 | Amb a 1 | 0 | Cry j 1 | 0 | Cup a 1 | 0,85 |
| Italy - CAAM 1666 | M | 51 | Amb a 1 | 0 | Cry j 1 | 0 | Cup a 1 | 0,06 |
| Italy - CAAM 1667 | F | 51 | Amb a 1 | 0 | Cry j 1 | 0,73 | Cup a 1 | 10,3 |
| Italy - CAAM 1668 | F | 51 | Amb a 1 | 0 | Cry j 1 | 0,33 | Cup a 1 | 2,25 |
| Italy - CAAM 1669 | M | 51 | Amb a 1 | 0 | Cry j 1 | 0,43 | Cup a 1 | 1,92 |
| Italy - CAAM 1670 | M | 51 | Amb a 1 | 0 | Cry j 1 | 0 | Cup a 1 | 0,97 |
| Italy - CAAM 1671 | F | 51 | Amb a 1 | 0 | Cry j 1 | 1,18 | Cup a 1 | 2,52 |
| Italy - CAAM 1672 | F | 51 | Amb a 1 | 0 | Cry j 1 | 0,54 | Cup a 1 | 5,42 |
| Italy - CAAM 1673 | F | 51 | Amb a 1 | 0 | Cry j 1 | 0 | Cup a 1 | 2,01 |
| Italy - CAAM 1674 | M | 51 | Amb a 1 | 0 | Cry j 1 | 0 | Cup a 1 | 0,9 |
| Italy - CAAM 1675 | M | 51 | Amb a 1 | 0 | Cry j 1 | 1,05 | Cup a 1 | 3,45 |
| Italy - CAAM 1676 | M | 51 | Amb a 1 | 0 | Cry j 1 | 1,33 | Cup a 1 | 3,02 |
| Italy - CAAM 1677 | M | 51 | Amb a 1 | 0 | Cry j 1 | 0,72 | Cup a 1 | 0,51 |
| Italy - CAAM 1678 | F | 51 | Amb a 1 | 0 | Cry j 1 | 0 | Cup a 1 | 2,86 |
| Italy - CAAM 1679 | M | 51 | Amb a 1 | 0 | Cry j 1 | 0,98 | Cup a 1 | 3,65 |
| Italy - CAAM 1680 | F | 51 | Amb a 1 | 0 | Cry j 1 | 0 | Cup a 1 | 0,15 |
| Italy - CAAM 1681 | M | 51 | Amb a 1 | 0 | Cry j 1 | 1,29 | Cup a 1 | 2,65 |
| Italy - CAAM 1682 | M | 51 | Amb a 1 | 0 | Cry j 1 | 1,23 | Cup a 1 | 2,59 |
| Italy - CAAM 1683 | F | 51 | Amb a 1 | 0 | Cry j 1 | 0,04 | Cup a 1 | 0,41 |
| Italy - CAAM 1684 | F | 51 | Amb a 1 | 0 | Cry j 1 | 0,21 | Cup a 1 | 3,56 |
| Italy - CAAM 1685 | F | 51 | Amb a 1 | 0 | Cry j 1 | 0,34 | Cup a 1 | 2,43 |
| Italy - CAAM 1686 | M | 51 | Amb a 1 | 0 | Cry j 1 | 0 | Cup a 1 | 0,15 |
| Italy - CAAM 1687 | F | 51 | Amb a 1 | 0 | Cry j 1 | 0 | Cup a 1 | 0,24 |
| Italy - CAAM 1688 | F | 51 | Amb a 1 | 0 | Cry j 1 | 0 | Cup a 1 | 0,39 |
| Italy - CAAM 1689 | F | 51 | Amb a 1 | 0 | Cry j 1 | 0 | Cup a 1 | 1,11 |
| Italy - CAAM 1690 | M | 51 | Amb a 1 | 0 | Cry j 1 | 4,3 | Cup a 1 | 7,68 |
| Italy - CAAM 1691 | F | 51 | Amb a 1 | 0 | Cry j 1 | 3,89 | Cup a 1 | 12,48 |
| Italy - CAAM 1692 | M | 51 | Amb a 1 | 0 | Cry j 1 | 0 | Cup a 1 | 2,87 |
| Italy - CAAM 1693 | F | 51 | Amb a 1 | 0 | Cry j 1 | 2,32 | Cup a 1 | 5,12 |
| Italy - CAAM 1694 | F | 51 | Amb a 1 | 0 | Cry j 1 | 0 | Cup a 1 | 0,23 |
| Italy - CAAM 1695 | F | 51 | Amb a 1 | 0 | Cry j 1 | 6,67 | Cup a 1 | 25,46 |
| Italy - CAAM 1696 | F | 51 | Amb a 1 | 0 | Cry j 1 | 9,55 | Cup a 1 | 32,63 |
| Italy - CAAM 1697 | F | 51 | Amb a 1 | 0 | Cry j 1 | 0,54 | Cup a 1 | 4,94 |
| Italy - CAAM 1698 | M | 51 | Amb a 1 | 0 | Cry j 1 | 4,08 | Cup a 1 | 11,77 |
| Italy - CAAM 1699 | F | 51 | Amb a 1 | 0 | Cry j 1 | 7,53 | Cup a 1 | 45,59 |
| Italy - CAAM 1700 | M | 51 | Amb a 1 | 0 | Cry j 1 | 0,61 | Cup a 1 | 2,62 |
| Italy - CAAM 1701 | M | 51 | Amb a 1 | 0 | Cry j 1 | 0,06 | Cup a 1 | 2,29 |
| Italy - CAAM 1702 | F | 51 | Amb a 1 | 0 | Cry j 1 | 0 | Cup a 1 | 0,31 |
| Italy - CAAM 1703 | M | 51 | Amb a 1 | 0 | Cry j 1 | 0 | Cup a 1 | 0,41 |
| Italy - CAAM 1704 | F | 51 | Amb a 1 | 0 | Cry j 1 | 5,98 | Cup a 1 | 35,8 |
| Italy - CAAM 1705 | F | 51 | Amb a 1 | 0 | Cry j 1 | 0,13 | Cup a 1 | 1,08 |
| Italy - CAAM 1706 | F | 51 | Amb a 1 | 0 | Cry j 1 | 2,26 | Cup a 1 | 7,53 |
| Italy - CAAM 1707 | M | 51 | Amb a 1 | 0 | Cry j 1 | 0 | Cup a 1 | 0,48 |
| Italy - CAAM 1708 | M | 51 | Amb a 1 | 0 | Cry j 1 | 0 | Cup a 1 | 0,18 |
| Italy - CAAM 1709 | M | 51 | Amb a 1 | 0 | Cry j 1 | 0,09 | Cup a 1 | 1,16 |
| Italy - CAAM 1710 | F | 51 | Amb a 1 | 0 | Cry j 1 | 2,59 | Cup a 1 | 9,37 |
| Italy - CAAM 1711 | F | 51 | Amb a 1 | 0 | Cry j 1 | 0 | Cup a 1 | 0,19 |
| Italy - CAAM 1712 | F | 51 | Amb a 1 | 0 | Cry j 1 | 0,57 | Cup a 1 | 27,02 |
| Italy - CAAM 1713 | F | 51 | Amb a 1 | 0 | Cry j 1 | 0 | Cup a 1 | 0,89 |
| Italy - CAAM 1714 | F | 51 | Amb a 1 | 0 | Cry j 1 | 0 | Cup a 1 | 5,5 |
| Italy - CAAM 1715 | F | 51 | Amb a 1 | 0 | Cry j 1 | 0 | Cup a 1 | 1,99 |
| Italy - CAAM 1716 | F | 51 | Amb a 1 | 0 | Cry j 1 | 0 | Cup a 1 | 1 |
| Italy - CAAM 1717 | F | 51 | Amb a 1 | 0 | Cry j 1 | 0 | Cup a 1 | 14 |
| Italy - CAAM 1718 | M | 51 | Amb a 1 | 0 | Cry j 1 | 0,08 | Cup a 1 | 0,79 |
| Italy - CAAM 1719 | F | 51 | Amb a 1 | 0 | Cry j 1 | 0 | Cup a 1 | 4,8 |
| Italy - CAAM 1720 | F | 51 | Amb a 1 | 0 | Cry j 1 | 0,57 | Cup a 1 | 6,82 |
| Italy - CAAM 1721 | M | 51 | Amb a 1 | 0 | Cry j 1 | 1,86 | Cup a 1 | 4,31 |
| Italy - CAAM 1722 | F | 50 | Amb a 1 | 0 | Cry j 1 | 0,61 | Cup a 1 | 1,56 |
| Italy - CAAM 1723 | M | 50 | Amb a 1 | 0 | Cry j 1 | 0,13 | Cup a 1 | 2,89 |
| Italy - CAAM 1724 | F | 50 | Amb a 1 | 0 | Cry j 1 | 0,57 | Cup a 1 | 8,21 |
| Italy - CAAM 1725 | M | 50 | Amb a 1 | 0 | Cry j 1 | 0 | Cup a 1 | 6,1 |
| Italy - CAAM 1726 | F | 50 | Amb a 1 | 0 | Cry j 1 | 0 | Cup a 1 | 1,06 |
| Italy - CAAM 1727 | M | 50 | Amb a 1 | 0 | Cry j 1 | 4,25 | Cup a 1 | 13,28 |
| Italy - CAAM 1728 | F | 50 | Amb a 1 | 0 | Cry j 1 | 0,44 | Cup a 1 | 1,57 |
| Italy - CAAM 1729 | F | 50 | Amb a 1 | 0 | Cry j 1 | 0,54 | Cup a 1 | 4,87 |
| Italy - CAAM 1730 | M | 50 | Amb a 1 | 0 | Cry j 1 | 0 | Cup a 1 | 0,54 |
| Italy - CAAM 1731 | F | 50 | Amb a 1 | 0 | Cry j 1 | 0,27 | Cup a 1 | 1,25 |
| Italy - CAAM 1732 | M | 50 | Amb a 1 | 0 | Cry j 1 | 3,03 | Cup a 1 | 6,09 |
| Italy - CAAM 1733 | F | 50 | Amb a 1 | 0 | Cry j 1 | 0,67 | Cup a 1 | 6,41 |
| Italy - CAAM 1734 | M | 50 | Amb a 1 | 0 | Cry j 1 | 0,04 | Cup a 1 | 0 |
| Italy - CAAM 1735 | F | 50 | Amb a 1 | 0 | Cry j 1 | 1,76 | Cup a 1 | 7,16 |
| Italy - CAAM 1736 | F | 50 | Amb a 1 | 0 | Cry j 1 | 0,69 | Cup a 1 | 1,13 |
| Italy - CAAM 1737 | M | 50 | Amb a 1 | 0 | Cry j 1 | 0 | Cup a 1 | 0,21 |
| Italy - CAAM 1738 | F | 50 | Amb a 1 | 0 | Cry j 1 | 0,52 | Cup a 1 | 3,82 |
| Italy - CAAM 1739 | F | 50 | Amb a 1 | 0 | Cry j 1 | 0,26 | Cup a 1 | 0,55 |
| Italy - CAAM 1740 | M | 50 | Amb a 1 | 0 | Cry j 1 | 0,53 | Cup a 1 | 2,76 |
| Italy - CAAM 1741 | F | 50 | Amb a 1 | 0 | Cry j 1 | 2,33 | Cup a 1 | 0,98 |
| Italy - CAAM 1742 | F | 50 | Amb a 1 | 0 | Cry j 1 | 0,33 | Cup a 1 | 1,91 |
| Italy - CAAM 1743 | F | 50 | Amb a 1 | 0 | Cry j 1 | 0 | Cup a 1 | 0,85 |
| Italy - CAAM 1744 | M | 50 | Amb a 1 | 0 | Cry j 1 | 0 | Cup a 1 | 0,23 |
| Italy - CAAM 1745 | F | 50 | Amb a 1 | 0 | Cry j 1 | 0,35 | Cup a 1 | 0,75 |
| Italy - CAAM 1746 | F | 50 | Amb a 1 | 0 | Cry j 1 | 0,13 | Cup a 1 | 0,93 |
| Italy - CAAM 1747 | M | 50 | Amb a 1 | 0 | Cry j 1 | 3,34 | Cup a 1 | 1,58 |
| Italy - CAAM 1748 | F | 50 | Amb a 1 | 0 | Cry j 1 | 0 | Cup a 1 | 0,91 |
| Italy - CAAM 1749 | F | 50 | Amb a 1 | 0 | Cry j 1 | 0,24 | Cup a 1 | 0,15 |
| Italy - CAAM 1750 | F | 50 | Amb a 1 | 0 | Cry j 1 | 1,42 | Cup a 1 | 6,82 |
| Italy - CAAM 1751 | F | 50 | Amb a 1 | 0 | Cry j 1 | 0 | Cup a 1 | 2,97 |
| Italy - CAAM 1752 | F | 50 | Amb a 1 | 0 | Cry j 1 | 0,7 | Cup a 1 | 1,87 |
| Italy - CAAM 1753 | F | 50 | Amb a 1 | 0 | Cry j 1 | 0 | Cup a 1 | 0,72 |
| Italy - CAAM 1754 | F | 50 | Amb a 1 | 0 | Cry j 1 | 0,85 | Cup a 1 | 7,12 |
| Italy - CAAM 1755 | F | 50 | Amb a 1 | 0 | Cry j 1 | 0 | Cup a 1 | 0,32 |
| Italy - CAAM 1756 | M | 50 | Amb a 1 | 0 | Cry j 1 | 0,46 | Cup a 1 | 1,38 |
| Italy - CAAM 1757 | F | 50 | Amb a 1 | 0 | Cry j 1 | 0,06 | Cup a 1 | 1,61 |
| Italy - CAAM 1758 | F | 50 | Amb a 1 | 0 | Cry j 1 | 0 | Cup a 1 | 0,25 |
| Italy - CAAM 1759 | F | 50 | Amb a 1 | 0 | Cry j 1 | 0,6 | Cup a 1 | 2,18 |
| Italy - CAAM 1760 | M | 50 | Amb a 1 | 0 | Cry j 1 | 0,02 | Cup a 1 | 0,03 |
| Italy - CAAM 1761 | M | 50 | Amb a 1 | 0 | Cry j 1 | 0,4 | Cup a 1 | 1,91 |
| Italy - CAAM 1762 | M | 50 | Amb a 1 | 0 | Cry j 1 | 1,85 | Cup a 1 | 3,06 |
| Italy - CAAM 1763 | F | 50 | Amb a 1 | 0 | Cry j 1 | 0 | Cup a 1 | 0,58 |
| Italy - CAAM 1764 | M | 50 | Amb a 1 | 0 | Cry j 1 | 1,44 | Cup a 1 | 3,35 |
| Italy - CAAM 1765 | F | 50 | Amb a 1 | 0 | Cry j 1 | 0,27 | Cup a 1 | 1,91 |
| Italy - CAAM 1766 | F | 50 | Amb a 1 | 0 | Cry j 1 | 0,15 | Cup a 1 | 1,15 |
| Italy - CAAM 1767 | F | 50 | Amb a 1 | 0 | Cry j 1 | 0,2 | Cup a 1 | 1,36 |
| Italy - CAAM 1768 | M | 50 | Amb a 1 | 0 | Cry j 1 | 2,44 | Cup a 1 | 31,73 |
| Italy - CAAM 1769 | F | 50 | Amb a 1 | 0 | Cry j 1 | 1,58 | Cup a 1 | 6,19 |
| Italy - CAAM 1770 | F | 50 | Amb a 1 | 0 | Cry j 1 | 0 | Cup a 1 | 6,55 |
| Italy - CAAM 1771 | M | 50 | Amb a 1 | 0 | Cry j 1 | 0 | Cup a 1 | 2,55 |
| Italy - CAAM 1772 | F | 50 | Amb a 1 | 0 | Cry j 1 | 0 | Cup a 1 | 1,05 |
| Italy - CAAM 1773 | F | 50 | Amb a 1 | 0 | Cry j 1 | 2,13 | Cup a 1 | 26,43 |
| Italy - CAAM 1774 | F | 50 | Amb a 1 | 0 | Cry j 1 | 10,58 | Cup a 1 | 20,97 |
| Italy - CAAM 1775 | F | 50 | Amb a 1 | 0 | Cry j 1 | 0 | Cup a 1 | 0,19 |
| Italy - CAAM 1776 | F | 50 | Amb a 1 | 0 | Cry j 1 | 0,05 | Cup a 1 | 0,09 |
| Italy - CAAM 1777 | F | 50 | Amb a 1 | 0 | Cry j 1 | 0,23 | Cup a 1 | 0,47 |
| Italy - CAAM 1778 | M | 50 | Amb a 1 | 0 | Cry j 1 | 0 | Cup a 1 | 0,03 |
| Italy - CAAM 1779 | F | 50 | Amb a 1 | 0 | Cry j 1 | 0,09 | Cup a 1 | 2,4 |
| Italy - CAAM 1780 | F | 50 | Amb a 1 | 0 | Cry j 1 | 0,1 | Cup a 1 | 1,6 |
| Italy - CAAM 1781 | F | 50 | Amb a 1 | 0 | Cry j 1 | 0,54 | Cup a 1 | 2,04 |
| Italy - CAAM 1782 | F | 50 | Amb a 1 | 0 | Cry j 1 | 0,49 | Cup a 1 | 1,72 |
| Italy - CAAM 1783 | F | 50 | Amb a 1 | 0 | Cry j 1 | 0,89 | Cup a 1 | 1,76 |
| Italy - CAAM 1784 | F | 50 | Amb a 1 | 0 | Cry j 1 | 0,13 | Cup a 1 | 3,67 |
| Italy - CAAM 1785 | F | 50 | Amb a 1 | 0 | Cry j 1 | 0 | Cup a 1 | 4,4 |
| Italy - CAAM 1786 | M | 50 | Amb a 1 | 0 | Cry j 1 | 0,14 | Cup a 1 | 0,43 |
| Italy - CAAM 1787 | F | 50 | Amb a 1 | 0 | Cry j 1 | 0,25 | Cup a 1 | 1,59 |
| Italy - CAAM 1788 | F | 50 | Amb a 1 | 0 | Cry j 1 | 0,32 | Cup a 1 | 3,12 |
| Italy - CAAM 1789 | F | 50 | Amb a 1 | 0 | Cry j 1 | 0,03 | Cup a 1 | 0,64 |
| Italy - CAAM 1790 | F | 50 | Amb a 1 | 0 | Cry j 1 | 0,04 | Cup a 1 | 0,93 |
| Italy - CAAM 1791 | M | 50 | Amb a 1 | 0 | Cry j 1 | 0,2 | Cup a 1 | 2,06 |
| Italy - CAAM 1792 | F | 50 | Amb a 1 | 0 | Cry j 1 | 5,07 | Cup a 1 | 16,13 |
| Italy - CAAM 1793 | F | 50 | Amb a 1 | 0 | Cry j 1 | 0 | Cup a 1 | 0,53 |
| Italy - CAAM 1794 | F | 50 | Amb a 1 | 0 | Cry j 1 | 0 | Cup a 1 | 1,1 |
| Italy - CAAM 1795 | F | 50 | Amb a 1 | 0 | Cry j 1 | 2,18 | Cup a 1 | 10,71 |
| Italy - CAAM 1796 | M | 50 | Amb a 1 | 0 | Cry j 1 | 0,02 | Cup a 1 | 0,67 |
| Italy - CAAM 1797 | M | 50 | Amb a 1 | 0 | Cry j 1 | 0 | Cup a 1 | 0,02 |
| Italy - CAAM 1798 | F | 50 | Amb a 1 | 0 | Cry j 1 | 0 | Cup a 1 | 0,05 |
| Italy - CAAM 1799 | F | 50 | Amb a 1 | 0 | Cry j 1 | 0,38 | Cup a 1 | 5,58 |
| Italy - CAAM 1800 | F | 50 | Amb a 1 | 0 | Cry j 1 | 7,21 | Cup a 1 | 14,04 |
| Italy - CAAM 1801 | M | 50 | Amb a 1 | 0 | Cry j 1 | 0,59 | Cup a 1 | 1,1 |
| Italy - CAAM 1802 | M | 50 | Amb a 1 | 0 | Cry j 1 | 0,88 | Cup a 1 | 2,4 |
| Italy - CAAM 1803 | F | 50 | Amb a 1 | 0 | Cry j 1 | 0 | Cup a 1 | 0,23 |
| Italy - CAAM 1804 | F | 50 | Amb a 1 | 0 | Cry j 1 | 0 | Cup a 1 | 0,09 |
| Italy - CAAM 1805 | F | 50 | Amb a 1 | 0 | Cry j 1 | 3,43 | Cup a 1 | 13,07 |
| Italy - CAAM 1806 | M | 50 | Amb a 1 | 0 | Cry j 1 | 3,43 | Cup a 1 | 13,52 |
| Italy - CAAM 1807 | F | 50 | Amb a 1 | 0 | Cry j 1 | 0 | Cup a 1 | 0,68 |
| Italy - CAAM 1808 | F | 50 | Amb a 1 | 0 | Cry j 1 | 0,69 | Cup a 1 | 1,47 |
| Italy - CAAM 1809 | M | 50 | Amb a 1 | 0 | Cry j 1 | 1,56 | Cup a 1 | 4,66 |
| Italy - CAAM 1810 | F | 50 | Amb a 1 | 0 | Cry j 1 | 0,64 | Cup a 1 | 2,85 |
| Italy - CAAM 1811 | F | 50 | Amb a 1 | 0 | Cry j 1 | 0 | Cup a 1 | 2,34 |
| Italy - CAAM 1812 | F | 50 | Amb a 1 | 0 | Cry j 1 | 0 | Cup a 1 | 1,13 |
| Italy - CAAM 1813 | M | 50 | Amb a 1 | 0 | Cry j 1 | 1,13 | Cup a 1 | 6,36 |
| Italy - CAAM 1814 | M | 50 | Amb a 1 | 0 | Cry j 1 | 0,25 | Cup a 1 | 10,05 |
| Italy - CAAM 1815 | M | 50 | Amb a 1 | 0 | Cry j 1 | 0,13 | Cup a 1 | 2,17 |
| Italy - CAAM 1816 | F | 50 | Amb a 1 | 0 | Cry j 1 | 1,47 | Cup a 1 | 6,91 |
| Italy - CAAM 1817 | F | 50 | Amb a 1 | 0 | Cry j 1 | 0 | Cup a 1 | 3,04 |
| Italy - CAAM 1818 | F | 50 | Amb a 1 | 0 | Cry j 1 | 11,61 | Cup a 1 | 30,77 |
| Italy - CAAM 1819 | M | 50 | Amb a 1 | 0 | Cry j 1 | 0 | Cup a 1 | 0,47 |
| Italy - CAAM 1820 | M | 50 | Amb a 1 | 0 | Cry j 1 | 0,6 | Cup a 1 | 5,48 |
| Italy - CAAM 1821 | F | 50 | Amb a 1 | 0 | Cry j 1 | 0 | Cup a 1 | 1,24 |
| Italy - CAAM 1822 | M | 50 | Amb a 1 | 0 | Cry j 1 | 0,7 | Cup a 1 | 4,95 |
| Italy - CAAM 1823 | F | 50 | Amb a 1 | 0 | Cry j 1 | 0,69 | Cup a 1 | 9,5 |
| Italy - CAAM 1824 | F | 50 | Amb a 1 | 0 | Cry j 1 | 1,2 | Cup a 1 | 44,23 |
| Italy - CAAM 1825 | F | 50 | Amb a 1 | 0 | Cry j 1 | 12,26 | Cup a 1 | 23,23 |
| Italy - CAAM 1826 | M | 50 | Amb a 1 | 0 | Cry j 1 | 0,14 | Cup a 1 | 3,2 |
| Italy - CAAM 1827 | F | 50 | Amb a 1 | 0 | Cry j 1 | 0 | Cup a 1 | 0,82 |
| Italy - CAAM 1828 | F | 50 | Amb a 1 | 0 | Cry j 1 | 1,01 | Cup a 1 | 8,93 |
| Italy - CAAM 1829 | F | 50 | Amb a 1 | 0 | Cry j 1 | 1,1 | Cup a 1 | 36 |
| Italy - CAAM 1830 | M | 50 | Amb a 1 | 0 | Cry j 1 | 0 | Cup a 1 | 2,3 |
| Italy - CAAM 1831 | F | 50 | Amb a 1 | 0 | Cry j 1 | 0 | Cup a 1 | 20 |
| Italy - CAAM 1832 | F | 50 | Amb a 1 | 0 | Cry j 1 | 0 | Cup a 1 | 6,7 |
| Italy - CAAM 1833 | F | 50 | Amb a 1 | 0 | Cry j 1 | 0,17 | Cup a 1 | 2,69 |
| Italy - CAAM 1834 | F | 50 | Amb a 1 | 0 | Cry j 1 | 0,07 | Cup a 1 | 0,97 |
| Italy - CAAM 1835 | M | 50 | Amb a 1 | 0 | Cry j 1 | 0,28 | Cup a 1 | 11,17 |
| Italy - CAAM 1836 | F | 50 | Amb a 1 | 0 | Cry j 1 | 0,1 | Cup a 1 | 1,35 |
| Italy - CAAM 1837 | F | 50 | Amb a 1 | 0 | Cry j 1 | 1,27 | Cup a 1 | 36,3 |
| Italy - CAAM 1838 | F | 49 | Amb a 1 | 0 | Cry j 1 | 0,5 | Cup a 1 | 12 |
| Italy - CAAM 1839 | F | 49 | Amb a 1 | 0 | Cry j 1 | 0,19 | Cup a 1 | 0,45 |
| Italy - CAAM 1840 | F | 49 | Amb a 1 | 0 | Cry j 1 | 2,09 | Cup a 1 | 3,48 |
| Italy - CAAM 1841 | F | 49 | Amb a 1 | 0 | Cry j 1 | 0,79 | Cup a 1 | 4,2 |
| Italy - CAAM 1842 | F | 49 | Amb a 1 | 0 | Cry j 1 | 0 | Cup a 1 | 2,1 |
| Italy - CAAM 1843 | F | 49 | Amb a 1 | 0 | Cry j 1 | 0 | Cup a 1 | 1,14 |
| Italy - CAAM 1844 | F | 49 | Amb a 1 | 0 | Cry j 1 | 2,21 | Cup a 1 | 42,96 |
| Italy - CAAM 1845 | M | 49 | Amb a 1 | 0 | Cry j 1 | 2,72 | Cup a 1 | 1,22 |
| Italy - CAAM 1846 | M | 49 | Amb a 1 | 0 | Cry j 1 | 1,6 | Cup a 1 | 7,3 |
| Italy - CAAM 1847 | M | 49 | Amb a 1 | 0 | Cry j 1 | 0,78 | Cup a 1 | 3,85 |
| Italy - CAAM 1848 | M | 49 | Amb a 1 | 0 | Cry j 1 | 0 | Cup a 1 | 2,38 |
| Italy - CAAM 1849 | M | 49 | Amb a 1 | 0 | Cry j 1 | 0 | Cup a 1 | 0,68 |
| Italy - CAAM 1850 | F | 49 | Amb a 1 | 0 | Cry j 1 | 2,28 | Cup a 1 | 5,61 |
| Italy - CAAM 1851 | F | 49 | Amb a 1 | 0 | Cry j 1 | 1,4 | Cup a 1 | 3,31 |
| Italy - CAAM 1852 | M | 49 | Amb a 1 | 0 | Cry j 1 | 0,13 | Cup a 1 | 0,42 |
| Italy - CAAM 1853 | M | 49 | Amb a 1 | 0 | Cry j 1 | 0,31 | Cup a 1 | 0 |
| Italy - CAAM 1854 | F | 49 | Amb a 1 | 0 | Cry j 1 | 3,01 | Cup a 1 | 9,85 |
| Italy - CAAM 1855 | F | 49 | Amb a 1 | 0 | Cry j 1 | 0,55 | Cup a 1 | 0,66 |
| Italy - CAAM 1856 | F | 49 | Amb a 1 | 0 | Cry j 1 | 1,66 | Cup a 1 | 3,29 |
| Italy - CAAM 1857 | M | 49 | Amb a 1 | 0 | Cry j 1 | 0,6 | Cup a 1 | 0,99 |
| Italy - CAAM 1858 | F | 49 | Amb a 1 | 0 | Cry j 1 | 1,09 | Cup a 1 | 0,98 |
| Italy - CAAM 1859 | F | 49 | Amb a 1 | 0 | Cry j 1 | 15,21 | Cup a 1 | 22,57 |
| Italy - CAAM 1860 | M | 49 | Amb a 1 | 0 | Cry j 1 | 0,64 | Cup a 1 | 1,27 |
| Italy - CAAM 1861 | M | 49 | Amb a 1 | 0 | Cry j 1 | 0,67 | Cup a 1 | 3,39 |
| Italy - CAAM 1862 | M | 49 | Amb a 1 | 0 | Cry j 1 | 0 | Cup a 1 | 0,44 |
| Italy - CAAM 1863 | F | 49 | Amb a 1 | 0 | Cry j 1 | 0 | Cup a 1 | 0,29 |
| Italy - CAAM 1864 | M | 49 | Amb a 1 | 0 | Cry j 1 | 3,47 | Cup a 1 | 3,29 |
| Italy - CAAM 1865 | F | 49 | Amb a 1 | 0 | Cry j 1 | 8,88 | Cup a 1 | 6,65 |
| Italy - CAAM 1866 | F | 49 | Amb a 1 | 0 | Cry j 1 | 1,17 | Cup a 1 | 0,3 |
| Italy - CAAM 1867 | F | 49 | Amb a 1 | 0 | Cry j 1 | 0 | Cup a 1 | 1,42 |
| Italy - CAAM 1868 | M | 49 | Amb a 1 | 0 | Cry j 1 | 1,62 | Cup a 1 | 0 |
| Italy - CAAM 1869 | M | 49 | Amb a 1 | 0 | Cry j 1 | 4,19 | Cup a 1 | 0 |
| Italy - CAAM 1870 | F | 49 | Amb a 1 | 0 | Cry j 1 | 2,2 | Cup a 1 | 3,24 |
| Italy - CAAM 1871 | M | 49 | Amb a 1 | 0 | Cry j 1 | 7,26 | Cup a 1 | 5,56 |
| Italy - CAAM 1872 | F | 49 | Amb a 1 | 0 | Cry j 1 | 5,44 | Cup a 1 | 10,67 |
| Italy - CAAM 1873 | M | 49 | Amb a 1 | 0 | Cry j 1 | 0,24 | Cup a 1 | 1,76 |
| Italy - CAAM 1874 | M | 49 | Amb a 1 | 0 | Cry j 1 | 5,67 | Cup a 1 | 42,79 |
| Italy - CAAM 1875 | M | 49 | Amb a 1 | 0 | Cry j 1 | 0,54 | Cup a 1 | 1,56 |
| Italy - CAAM 1876 | F | 49 | Amb a 1 | 0 | Cry j 1 | 0,64 | Cup a 1 | 0,91 |
| Italy - CAAM 1877 | F | 49 | Amb a 1 | 0 | Cry j 1 | 0,18 | Cup a 1 | 0,15 |
| Italy - CAAM 1878 | M | 49 | Amb a 1 | 0 | Cry j 1 | 0 | Cup a 1 | 0,1 |
| Italy - CAAM 1879 | F | 49 | Amb a 1 | 0 | Cry j 1 | 0,64 | Cup a 1 | 0,39 |
| Italy - CAAM 1880 | F | 49 | Amb a 1 | 0 | Cry j 1 | 0,03 | Cup a 1 | 1,83 |
| Italy - CAAM 1881 | M | 49 | Amb a 1 | 0 | Cry j 1 | 2,83 | Cup a 1 | 10,37 |
| Italy - CAAM 1882 | F | 49 | Amb a 1 | 0 | Cry j 1 | 12,04 | Cup a 1 | 24,58 |
| Italy - CAAM 1883 | M | 49 | Amb a 1 | 0 | Cry j 1 | 0,35 | Cup a 1 | 1,8 |
| Italy - CAAM 1884 | M | 49 | Amb a 1 | 0 | Cry j 1 | 0 | Cup a 1 | 1,5 |
| Italy - CAAM 1885 | F | 49 | Amb a 1 | 0 | Cry j 1 | 9,81 | Cup a 1 | 9,2 |
| Italy - CAAM 1886 | F | 49 | Amb a 1 | 0 | Cry j 1 | 0 | Cup a 1 | 0,13 |
| Italy - CAAM 1887 | F | 49 | Amb a 1 | 0 | Cry j 1 | 0 | Cup a 1 | 0,57 |
| Italy - CAAM 1888 | F | 49 | Amb a 1 | 0 | Cry j 1 | 4,44 | Cup a 1 | 8,64 |
| Italy - CAAM 1889 | M | 49 | Amb a 1 | 0 | Cry j 1 | 0 | Cup a 1 | 0,3 |
| Italy - CAAM 1890 | M | 49 | Amb a 1 | 0 | Cry j 1 | 0 | Cup a 1 | 3,29 |
| Italy - CAAM 1891 | M | 49 | Amb a 1 | 0 | Cry j 1 | 0,37 | Cup a 1 | 2,31 |
| Italy - CAAM 1892 | F | 49 | Amb a 1 | 0 | Cry j 1 | 1,19 | Cup a 1 | 3,91 |
| Italy - CAAM 1893 | F | 49 | Amb a 1 | 0 | Cry j 1 | 2,65 | Cup a 1 | 6,66 |
| Italy - CAAM 1894 | M | 49 | Amb a 1 | 0 | Cry j 1 | 1,89 | Cup a 1 | 3,47 |
| Italy - CAAM 1895 | F | 49 | Amb a 1 | 0 | Cry j 1 | 13,05 | Cup a 1 | 54,75 |
| Italy - CAAM 1896 | F | 49 | Amb a 1 | 0 | Cry j 1 | 0,63 | Cup a 1 | 3,93 |
| Italy - CAAM 1897 | F | 49 | Amb a 1 | 0 | Cry j 1 | 1,84 | Cup a 1 | 3,16 |
| Italy - CAAM 1898 | F | 49 | Amb a 1 | 0 | Cry j 1 | 0,06 | Cup a 1 | 5,91 |
| Italy - CAAM 1899 | M | 49 | Amb a 1 | 0 | Cry j 1 | 3,39 | Cup a 1 | 7,1 |
| Italy - CAAM 1900 | M | 49 | Amb a 1 | 0 | Cry j 1 | 0,2 | Cup a 1 | 0,47 |
| Italy - CAAM 1901 | F | 49 | Amb a 1 | 0 | Cry j 1 | 0,05 | Cup a 1 | 2,32 |
| Italy - CAAM 1902 | M | 49 | Amb a 1 | 0 | Cry j 1 | 1,2 | Cup a 1 | 9,13 |
| Italy - CAAM 1903 | F | 49 | Amb a 1 | 0 | Cry j 1 | 1,24 | Cup a 1 | 2,44 |
| Italy - CAAM 1904 | M | 49 | Amb a 1 | 0 | Cry j 1 | 3 | Cup a 1 | 11,33 |
| Italy - CAAM 1905 | F | 49 | Amb a 1 | 0 | Cry j 1 | 0,42 | Cup a 1 | 2,93 |
| Italy - CAAM 1906 | F | 49 | Amb a 1 | 0 | Cry j 1 | 0,04 | Cup a 1 | 0,24 |
| Italy - CAAM 1907 | M | 49 | Amb a 1 | 0 | Cry j 1 | 3,94 | Cup a 1 | 13,18 |
| Italy - CAAM 1908 | F | 49 | Amb a 1 | 0 | Cry j 1 | 0 | Cup a 1 | 0,34 |
| Italy - CAAM 1909 | F | 49 | Amb a 1 | 0 | Cry j 1 | 0,31 | Cup a 1 | 1,39 |
| Italy - CAAM 1910 | F | 49 | Amb a 1 | 0 | Cry j 1 | 5,65 | Cup a 1 | 11,88 |
| Italy - CAAM 1911 | F | 49 | Amb a 1 | 0 | Cry j 1 | 0,14 | Cup a 1 | 0,32 |
| Italy - CAAM 1912 | F | 49 | Amb a 1 | 0 | Cry j 1 | 4,62 | Cup a 1 | 11,42 |
| Italy - CAAM 1913 | F | 49 | Amb a 1 | 0 | Cry j 1 | 0,33 | Cup a 1 | 15,93 |
| Italy - CAAM 1914 | M | 49 | Amb a 1 | 0 | Cry j 1 | 0 | Cup a 1 | 3,35 |
| Italy - CAAM 1915 | F | 49 | Amb a 1 | 0 | Cry j 1 | 3,47 | Cup a 1 | 8,22 |
| Italy - CAAM 1916 | F | 49 | Amb a 1 | 0 | Cry j 1 | 1,11 | Cup a 1 | 4,56 |
| Italy - CAAM 1917 | F | 49 | Amb a 1 | 0 | Cry j 1 | 0 | Cup a 1 | 0,19 |
| Italy - CAAM 1918 | F | 49 | Amb a 1 | 0 | Cry j 1 | 1,17 | Cup a 1 | 5,26 |
| Italy - CAAM 1919 | F | 49 | Amb a 1 | 0 | Cry j 1 | 0,17 | Cup a 1 | 0 |
| Italy - CAAM 1920 | M | 49 | Amb a 1 | 0 | Cry j 1 | 1,54 | Cup a 1 | 8,04 |
| Italy - CAAM 1921 | F | 49 | Amb a 1 | 0 | Cry j 1 | 0,63 | Cup a 1 | 14,39 |
| Italy - CAAM 1922 | F | 49 | Amb a 1 | 0 | Cry j 1 | 0 | Cup a 1 | 0,17 |
| Italy - CAAM 1923 | M | 49 | Amb a 1 | 0 | Cry j 1 | 3,56 | Cup a 1 | 11,79 |
| Italy - CAAM 1924 | F | 49 | Amb a 1 | 0 | Cry j 1 | 2,43 | Cup a 1 | 18,35 |
| Italy - CAAM 1925 | F | 49 | Amb a 1 | 0 | Cry j 1 | 0,65 | Cup a 1 | 4,72 |
| Italy - CAAM 1926 | F | 49 | Amb a 1 | 0 | Cry j 1 | 2,02 | Cup a 1 | 9,46 |
| Italy - CAAM 1927 | M | 49 | Amb a 1 | 0 | Cry j 1 | 0,26 | Cup a 1 | 0,18 |
| Italy - CAAM 1928 | M | 49 | Amb a 1 | 0 | Cry j 1 | 0,52 | Cup a 1 | 1,05 |
| Italy - CAAM 1929 | M | 49 | Amb a 1 | 0 | Cry j 1 | 0,61 | Cup a 1 | 7,76 |
| Italy - CAAM 1930 | M | 49 | Amb a 1 | 0 | Cry j 1 | 0,38 | Cup a 1 | 3,2 |
| Italy - CAAM 1931 | F | 49 | Amb a 1 | 0 | Cry j 1 | 0 | Cup a 1 | 0,55 |
| Italy - CAAM 1932 | M | 49 | Amb a 1 | 0 | Cry j 1 | 0,04 | Cup a 1 | 2,99 |
| Italy - CAAM 1933 | F | 49 | Amb a 1 | 0 | Cry j 1 | 0 | Cup a 1 | 0,19 |
| Italy - CAAM 1934 | F | 49 | Amb a 1 | 0 | Cry j 1 | 7,14 | Cup a 1 | 21,34 |
| Italy - CAAM 1935 | M | 49 | Amb a 1 | 0 | Cry j 1 | 2,7 | Cup a 1 | 4,44 |
| Italy - CAAM 1936 | F | 49 | Amb a 1 | 0 | Cry j 1 | 1,52 | Cup a 1 | 18,86 |
| Italy - CAAM 1937 | M | 49 | Amb a 1 | 0 | Cry j 1 | 6,36 | Cup a 1 | 17,23 |
| Italy - CAAM 1938 | M | 49 | Amb a 1 | 0 | Cry j 1 | 0 | Cup a 1 | 2,72 |
| Italy - CAAM 1939 | M | 49 | Amb a 1 | 0 | Cry j 1 | 1,09 | Cup a 1 | 13,45 |
| Italy - CAAM 1940 | F | 49 | Amb a 1 | 0 | Cry j 1 | 0 | Cup a 1 | 0,09 |
| Italy - CAAM 1941 | F | 49 | Amb a 1 | 0 | Cry j 1 | 1,58 | Cup a 1 | 13,26 |
| Italy - CAAM 1942 | M | 49 | Amb a 1 | 0 | Cry j 1 | 0,18 | Cup a 1 | 0,74 |
| Italy - CAAM 1943 | F | 49 | Amb a 1 | 0 | Cry j 1 | 0,23 | Cup a 1 | 6,09 |
| Italy - CAAM 1944 | F | 49 | Amb a 1 | 0 | Cry j 1 | 2,44 | Cup a 1 | 7,42 |
| Italy - CAAM 1945 | F | 49 | Amb a 1 | 0 | Cry j 1 | 0 | Cup a 1 | 1,02 |
| Italy - CAAM 1946 | F | 49 | Amb a 1 | 0 | Cry j 1 | 0 | Cup a 1 | 0,49 |
| Italy - CAAM 1947 | M | 49 | Amb a 1 | 0 | Cry j 1 | 1,29 | Cup a 1 | 21,12 |
| Italy - CAAM 1948 | F | 49 | Amb a 1 | 0 | Cry j 1 | 14,05 | Cup a 1 | 52,25 |
| Italy - CAAM 1949 | F | 49 | Amb a 1 | 0 | Cry j 1 | 0,41 | Cup a 1 | 1,68 |
| Italy - CAAM 1950 | M | 49 | Amb a 1 | 0 | Cry j 1 | 0 | Cup a 1 | 4,55 |
| Italy - CAAM 1951 | F | 49 | Amb a 1 | 0 | Cry j 1 | 0,73 | Cup a 1 | 7,67 |
| Italy - CAAM 1952 | F | 49 | Amb a 1 | 0 | Cry j 1 | 0,28 | Cup a 1 | 4,44 |
| Italy - CAAM 1953 | F | 49 | Amb a 1 | 0 | Cry j 1 | 0,27 | Cup a 1 | 0,25 |
| Italy - CAAM 1954 | F | 49 | Amb a 1 | 0 | Cry j 1 | 0,51 | Cup a 1 | 0,97 |
| Italy - CAAM 1955 | F | 49 | Amb a 1 | 0 | Cry j 1 | 0 | Cup a 1 | 1,88 |
| Italy - CAAM 1956 | M | 49 | Amb a 1 | 0 | Cry j 1 | 1,69 | Cup a 1 | 19,49 |
| Italy - CAAM 1957 | F | 49 | Amb a 1 | 0 | Cry j 1 | 0 | Cup a 1 | 3,67 |
| Italy - CAAM 1958 | M | 49 | Amb a 1 | 0 | Cry j 1 | 0 | Cup a 1 | 0,24 |
| Italy - CAAM 1959 | M | 49 | Amb a 1 | 0 | Cry j 1 | 0 | Cup a 1 | 0,89 |
| Italy - CAAM 1960 | M | 49 | Amb a 1 | 0 | Cry j 1 | 0 | Cup a 1 | 1,8 |
| Italy - CAAM 1961 | F | 49 | Amb a 1 | 0 | Cry j 1 | 2,43 | Cup a 1 | 23,3 |
| Italy - CAAM 1962 | F | 49 | Amb a 1 | 0 | Cry j 1 | 0,68 | Cup a 1 | 11,97 |
| Italy - CAAM 1963 | F | 49 | Amb a 1 | 0 | Cry j 1 | 0,55 | Cup a 1 | 3,64 |
| Italy - CAAM 1964 | F | 49 | Amb a 1 | 0 | Cry j 1 | 9,22 | Cup a 1 | 47,74 |
| Italy - CAAM 1965 | F | 49 | Amb a 1 | 0 | Cry j 1 | 0,65 | Cup a 1 | 13,73 |
| Italy - CAAM 1966 | F | 49 | Amb a 1 | 0 | Cry j 1 | 0,23 | Cup a 1 | 2,46 |
| Italy - CAAM 1967 | M | 49 | Amb a 1 | 0 | Cry j 1 | 1,81 | Cup a 1 | 15,13 |
| Italy - CAAM 1968 | F | 49 | Amb a 1 | 0 | Cry j 1 | 0 | Cup a 1 | 0,3 |
| Italy - CAAM 1969 | F | 49 | Amb a 1 | 0 | Cry j 1 | 0,72 | Cup a 1 | 7,62 |
| Italy - CAAM 1970 | F | 49 | Amb a 1 | 0 | Cry j 1 | 0 | Cup a 1 | 0,73 |
| Italy - CAAM 1971 | M | 49 | Amb a 1 | 0 | Cry j 1 | 3,9 | Cup a 1 | 8,51 |
| Italy - CAAM 1972 | F | 49 | Amb a 1 | 0 | Cry j 1 | 0 | Cup a 1 | 1,35 |
| Italy - CAAM 1973 | F | 49 | Amb a 1 | 0 | Cry j 1 | 1,8 | Cup a 1 | 45 |
| Italy - CAAM 1974 | F | 49 | Amb a 1 | 0 | Cry j 1 | 0 | Cup a 1 | 1,2 |
| Italy - CAAM 1975 | M | 49 | Amb a 1 | 0 | Cry j 1 | 0,27 | Cup a 1 | 1,01 |
| Italy - CAAM 1976 | F | 49 | Amb a 1 | 0 | Cry j 1 | 1,6 | Cup a 1 | 16 |
| Italy - CAAM 1977 | F | 49 | Amb a 1 | 0 | Cry j 1 | 0 | Cup a 1 | 0,7 |
| Italy - CAAM 1978 | M | 49 | Amb a 1 | 0 | Cry j 1 | 0 | Cup a 1 | 1,2 |
| Italy - CAAM 1979 | F | 49 | Amb a 1 | 0 | Cry j 1 | 0 | Cup a 1 | 2,2 |
| Italy - CAAM 1980 | F | 49 | Amb a 1 | 0 | Cry j 1 | 0,13 | Cup a 1 | 1,25 |
| Italy - CAAM 1981 | F | 49 | Amb a 1 | 0 | Cry j 1 | 0 | Cup a 1 | 0,31 |
| Italy - CAAM 1982 | F | 49 | Amb a 1 | 0 | Cry j 1 | 0,06 | Cup a 1 | 0,23 |
| Italy - CAAM 1983 | M | 49 | Amb a 1 | 0 | Cry j 1 | 0,46 | Cup a 1 | 2,11 |
| Italy - CAAM 1984 | F | 49 | Amb a 1 | 0 | Cry j 1 | 0,24 | Cup a 1 | 4,53 |
| Italy - CAAM 1985 | F | 49 | Amb a 1 | 0 | Cry j 1 | 4,51 | Cup a 1 | 62,05 |
| Italy - CAAM 1986 | F | 48 | Amb a 1 | 0 | Cry j 1 | 0,51 | Cup a 1 | 4,09 |
| Italy - CAAM 1987 | F | 48 | Amb a 1 | 0 | Cry j 1 | 9,98 | Cup a 1 | 4,53 |
| Italy - CAAM 1988 | M | 48 | Amb a 1 | 0 | Cry j 1 | 0 | Cup a 1 | 1,05 |
| Italy - CAAM 1989 | M | 48 | Amb a 1 | 0 | Cry j 1 | 0 | Cup a 1 | 1,72 |
| Italy - CAAM 1990 | M | 48 | Amb a 1 | 0 | Cry j 1 | 2,82 | Cup a 1 | 4,42 |
| Italy - CAAM 1991 | F | 48 | Amb a 1 | 0 | Cry j 1 | 0 | Cup a 1 | 0,75 |
| Italy - CAAM 1992 | F | 48 | Amb a 1 | 0 | Cry j 1 | 1,7 | Cup a 1 | 1,09 |
| Italy - CAAM 1993 | F | 48 | Amb a 1 | 0 | Cry j 1 | 0 | Cup a 1 | 0,07 |
| Italy - CAAM 1994 | F | 48 | Amb a 1 | 0 | Cry j 1 | 1,73 | Cup a 1 | 2,82 |
| Italy - CAAM 1995 | M | 48 | Amb a 1 | 0 | Cry j 1 | 1,46 | Cup a 1 | 1,91 |
| Italy - CAAM 1996 | F | 48 | Amb a 1 | 0 | Cry j 1 | 0 | Cup a 1 | 1 |
| Italy - CAAM 1997 | F | 48 | Amb a 1 | 0 | Cry j 1 | 0 | Cup a 1 | 0,35 |
| Italy - CAAM 1998 | M | 48 | Amb a 1 | 0 | Cry j 1 | 0 | Cup a 1 | 1,55 |
| Italy - CAAM 1999 | M | 48 | Amb a 1 | 0 | Cry j 1 | 1,61 | Cup a 1 | 3,53 |
| Italy - CAAM 2000 | F | 48 | Amb a 1 | 0 | Cry j 1 | 0,57 | Cup a 1 | 1,11 |
| Italy - CAAM 2001 | F | 48 | Amb a 1 | 0 | Cry j 1 | 0,6 | Cup a 1 | 15 |
| Italy - CAAM 2002 | F | 48 | Amb a 1 | 0 | Cry j 1 | 1,2 | Cup a 1 | 4,48 |
| Italy - CAAM 2003 | F | 48 | Amb a 1 | 0 | Cry j 1 | 0 | Cup a 1 | 0,04 |
| Italy - CAAM 2004 | F | 48 | Amb a 1 | 0 | Cry j 1 | 0,64 | Cup a 1 | 6,26 |
| Italy - CAAM 2005 | F | 48 | Amb a 1 | 0 | Cry j 1 | 5,34 | Cup a 1 | 17,68 |
| Italy - CAAM 2006 | M | 48 | Amb a 1 | 0 | Cry j 1 | 0,29 | Cup a 1 | 0 |
| Italy - CAAM 2007 | M | 48 | Amb a 1 | 0 | Cry j 1 | 1,13 | Cup a 1 | 1,46 |
| Italy - CAAM 2008 | F | 48 | Amb a 1 | 0 | Cry j 1 | 0,94 | Cup a 1 | 1,02 |
| Italy - CAAM 2009 | F | 48 | Amb a 1 | 0 | Cry j 1 | 1,13 | Cup a 1 | 8,03 |
| Italy - CAAM 2010 | M | 48 | Amb a 1 | 0 | Cry j 1 | 1,47 | Cup a 1 | 2,29 |
| Italy - CAAM 2011 | F | 48 | Amb a 1 | 0 | Cry j 1 | 0 | Cup a 1 | 1,47 |
| Italy - CAAM 2012 | F | 48 | Amb a 1 | 0 | Cry j 1 | 1,64 | Cup a 1 | 3,08 |
| Italy - CAAM 2013 | F | 48 | Amb a 1 | 0 | Cry j 1 | 0,39 | Cup a 1 | 1,76 |
| Italy - CAAM 2014 | F | 48 | Amb a 1 | 0 | Cry j 1 | 0 | Cup a 1 | 6,41 |
| Italy - CAAM 2015 | F | 48 | Amb a 1 | 0 | Cry j 1 | 2,58 | Cup a 1 | 7,08 |
| Italy - CAAM 2016 | F | 48 | Amb a 1 | 0 | Cry j 1 | 6,69 | Cup a 1 | 3,52 |
| Italy - CAAM 2017 | F | 48 | Amb a 1 | 0 | Cry j 1 | 0,73 | Cup a 1 | 0,66 |
| Italy - CAAM 2018 | M | 48 | Amb a 1 | 0 | Cry j 1 | 0,98 | Cup a 1 | 2,02 |
| Italy - CAAM 2019 | M | 48 | Amb a 1 | 0 | Cry j 1 | 0,12 | Cup a 1 | 1,98 |
| Italy - CAAM 2020 | M | 48 | Amb a 1 | 0 | Cry j 1 | 0,61 | Cup a 1 | 1,05 |
| Italy - CAAM 2021 | F | 48 | Amb a 1 | 0 | Cry j 1 | 0,63 | Cup a 1 | 0,84 |
| Italy - CAAM 2022 | F | 48 | Amb a 1 | 0 | Cry j 1 | 0,5 | Cup a 1 | 2,22 |
| Italy - CAAM 2023 | F | 48 | Amb a 1 | 0 | Cry j 1 | 0,33 | Cup a 1 | 0,75 |
| Italy - CAAM 2024 | M | 48 | Amb a 1 | 0 | Cry j 1 | 0,09 | Cup a 1 | 0,15 |
| Italy - CAAM 2025 | F | 48 | Amb a 1 | 0 | Cry j 1 | 0,11 | Cup a 1 | 8,9 |
| Italy - CAAM 2026 | M | 48 | Amb a 1 | 0 | Cry j 1 | 0,18 | Cup a 1 | 3,92 |
| Italy - CAAM 2027 | F | 48 | Amb a 1 | 0 | Cry j 1 | 0,05 | Cup a 1 | 0 |
| Italy - CAAM 2028 | M | 48 | Amb a 1 | 0 | Cry j 1 | 0,03 | Cup a 1 | 1,42 |
| Italy - CAAM 2029 | F | 48 | Amb a 1 | 0 | Cry j 1 | 0,37 | Cup a 1 | 3,64 |
| Italy - CAAM 2030 | F | 48 | Amb a 1 | 0 | Cry j 1 | 0 | Cup a 1 | 0,49 |
| Italy - CAAM 2031 | F | 48 | Amb a 1 | 0 | Cry j 1 | 2,17 | Cup a 1 | 2,52 |
| Italy - CAAM 2032 | F | 48 | Amb a 1 | 0 | Cry j 1 | 0 | Cup a 1 | 1,05 |
| Italy - CAAM 2033 | F | 48 | Amb a 1 | 0 | Cry j 1 | 1,19 | Cup a 1 | 4,05 |
| Italy - CAAM 2034 | M | 48 | Amb a 1 | 0 | Cry j 1 | 0 | Cup a 1 | 0,66 |
| Italy - CAAM 2035 | F | 48 | Amb a 1 | 0 | Cry j 1 | 3,38 | Cup a 1 | 3,78 |
| Italy - CAAM 2036 | F | 48 | Amb a 1 | 0 | Cry j 1 | 7,38 | Cup a 1 | 17,12 |
| Italy - CAAM 2037 | M | 48 | Amb a 1 | 0 | Cry j 1 | 1,66 | Cup a 1 | 5,51 |
| Italy - CAAM 2038 | F | 48 | Amb a 1 | 0 | Cry j 1 | 0 | Cup a 1 | 0,29 |
| Italy - CAAM 2039 | M | 48 | Amb a 1 | 0 | Cry j 1 | 0,25 | Cup a 1 | 1,39 |
| Italy - CAAM 2040 | M | 48 | Amb a 1 | 0 | Cry j 1 | 0 | Cup a 1 | 0,04 |
| Italy - CAAM 2041 | F | 48 | Amb a 1 | 0 | Cry j 1 | 0 | Cup a 1 | 0,73 |
| Italy - CAAM 2042 | F | 48 | Amb a 1 | 0 | Cry j 1 | 3,64 | Cup a 1 | 5,06 |
| Italy - CAAM 2043 | M | 48 | Amb a 1 | 0 | Cry j 1 | 1,07 | Cup a 1 | 1,64 |
| Italy - CAAM 2044 | M | 48 | Amb a 1 | 0 | Cry j 1 | 0,13 | Cup a 1 | 0,39 |
| Italy - CAAM 2045 | M | 48 | Amb a 1 | 0 | Cry j 1 | 0 | Cup a 1 | 0,64 |
| Italy - CAAM 2046 | F | 48 | Amb a 1 | 0 | Cry j 1 | 0 | Cup a 1 | 1,11 |
| Italy - CAAM 2047 | F | 48 | Amb a 1 | 0 | Cry j 1 | 0 | Cup a 1 | 2,02 |
| Italy - CAAM 2048 | F | 48 | Amb a 1 | 0 | Cry j 1 | 0,16 | Cup a 1 | 0,36 |
| Italy - CAAM 2049 | F | 48 | Amb a 1 | 0 | Cry j 1 | 0 | Cup a 1 | 1,36 |
| Italy - CAAM 2050 | F | 48 | Amb a 1 | 0 | Cry j 1 | 0,07 | Cup a 1 | 0,43 |
| Italy - CAAM 2051 | F | 48 | Amb a 1 | 0 | Cry j 1 | 0,1 | Cup a 1 | 2,73 |
| Italy - CAAM 2052 | M | 48 | Amb a 1 | 0 | Cry j 1 | 0,35 | Cup a 1 | 2,05 |
| Italy - CAAM 2053 | M | 48 | Amb a 1 | 0 | Cry j 1 | 0,19 | Cup a 1 | 6,03 |
| Italy - CAAM 2054 | M | 48 | Amb a 1 | 0 | Cry j 1 | 0 | Cup a 1 | 0,2 |
| Italy - CAAM 2055 | M | 48 | Amb a 1 | 0 | Cry j 1 | 0,07 | Cup a 1 | 0,29 |
| Italy - CAAM 2056 | M | 48 | Amb a 1 | 0 | Cry j 1 | 0 | Cup a 1 | 0,2 |
| Italy - CAAM 2057 | F | 48 | Amb a 1 | 0 | Cry j 1 | 0,04 | Cup a 1 | 0,07 |
| Italy - CAAM 2058 | M | 48 | Amb a 1 | 0 | Cry j 1 | 0 | Cup a 1 | 11,26 |
| Italy - CAAM 2059 | M | 48 | Amb a 1 | 0 | Cry j 1 | 1,62 | Cup a 1 | 19,68 |
| Italy - CAAM 2060 | F | 48 | Amb a 1 | 0 | Cry j 1 | 0,71 | Cup a 1 | 2,44 |
| Italy - CAAM 2061 | F | 48 | Amb a 1 | 0 | Cry j 1 | 0,97 | Cup a 1 | 4,15 |
| Italy - CAAM 2062 | M | 48 | Amb a 1 | 0 | Cry j 1 | 1 | Cup a 1 | 12 |
| Italy - CAAM 2063 | F | 48 | Amb a 1 | 0 | Cry j 1 | 0,67 | Cup a 1 | 2,83 |
| Italy - CAAM 2064 | M | 48 | Amb a 1 | 0 | Cry j 1 | 0,27 | Cup a 1 | 5,04 |
| Italy - CAAM 2065 | F | 48 | Amb a 1 | 0 | Cry j 1 | 9,29 | Cup a 1 | 27,44 |
| Italy - CAAM 2066 | M | 48 | Amb a 1 | 0 | Cry j 1 | 0,79 | Cup a 1 | 2,56 |
| Italy - CAAM 2067 | F | 48 | Amb a 1 | 0 | Cry j 1 | 0,61 | Cup a 1 | 3,14 |
| Italy - CAAM 2068 | M | 48 | Amb a 1 | 0 | Cry j 1 | 1,16 | Cup a 1 | 3,84 |
| Italy - CAAM 2069 | F | 48 | Amb a 1 | 0 | Cry j 1 | 8,87 | Cup a 1 | 16,86 |
| Italy - CAAM 2070 | M | 48 | Amb a 1 | 0 | Cry j 1 | 1 | Cup a 1 | 1,47 |
| Italy - CAAM 2071 | M | 48 | Amb a 1 | 0 | Cry j 1 | 0,12 | Cup a 1 | 3,59 |
| Italy - CAAM 2072 | F | 48 | Amb a 1 | 0 | Cry j 1 | 0 | Cup a 1 | 0,88 |
| Italy - CAAM 2073 | M | 48 | Amb a 1 | 0 | Cry j 1 | 0,74 | Cup a 1 | 3,97 |
| Italy - CAAM 2074 | F | 48 | Amb a 1 | 0 | Cry j 1 | 0 | Cup a 1 | 0,25 |
| Italy - CAAM 2075 | F | 48 | Amb a 1 | 0 | Cry j 1 | 0,99 | Cup a 1 | 5,46 |
| Italy - CAAM 2076 | M | 48 | Amb a 1 | 0 | Cry j 1 | 0,15 | Cup a 1 | 4,97 |
| Italy - CAAM 2077 | F | 48 | Amb a 1 | 0 | Cry j 1 | 5,09 | Cup a 1 | 29,78 |
| Italy - CAAM 2078 | F | 48 | Amb a 1 | 0 | Cry j 1 | 12,09 | Cup a 1 | 44,87 |
| Italy - CAAM 2079 | M | 48 | Amb a 1 | 0 | Cry j 1 | 1,32 | Cup a 1 | 11,27 |
| Italy - CAAM 2080 | F | 48 | Amb a 1 | 0 | Cry j 1 | 0,31 | Cup a 1 | 3,13 |
| Italy - CAAM 2081 | F | 48 | Amb a 1 | 0 | Cry j 1 | 0,32 | Cup a 1 | 1,04 |
| Italy - CAAM 2082 | F | 48 | Amb a 1 | 0 | Cry j 1 | 0,31 | Cup a 1 | 10,91 |
| Italy - CAAM 2083 | M | 48 | Amb a 1 | 0 | Cry j 1 | 0,13 | Cup a 1 | 1,54 |
| Italy - CAAM 2084 | F | 48 | Amb a 1 | 0 | Cry j 1 | 1,3 | Cup a 1 | 22,18 |
| Italy - CAAM 2085 | F | 48 | Amb a 1 | 0 | Cry j 1 | 0 | Cup a 1 | 0,24 |
| Italy - CAAM 2086 | M | 48 | Amb a 1 | 0 | Cry j 1 | 0,36 | Cup a 1 | 3,02 |
| Italy - CAAM 2087 | F | 48 | Amb a 1 | 0 | Cry j 1 | 0,16 | Cup a 1 | 8,31 |
| Italy - CAAM 2088 | F | 48 | Amb a 1 | 0 | Cry j 1 | 0 | Cup a 1 | 0,7 |
| Italy - CAAM 2089 | M | 48 | Amb a 1 | 0 | Cry j 1 | 0,09 | Cup a 1 | 4,56 |
| Italy - CAAM 2090 | F | 48 | Amb a 1 | 0 | Cry j 1 | 8 | Cup a 1 | 36 |
| Italy - CAAM 2091 | F | 48 | Amb a 1 | 0 | Cry j 1 | 0 | Cup a 1 | 3,3 |
| Italy - CAAM 2092 | F | 48 | Amb a 1 | 0 | Cry j 1 | 1,1 | Cup a 1 | 21 |
| Italy - CAAM 2093 | F | 48 | Amb a 1 | 0 | Cry j 1 | 0 | Cup a 1 | 0,9 |
| Italy - CAAM 2094 | F | 48 | Amb a 1 | 0 | Cry j 1 | 0 | Cup a 1 | 6,2 |
| Italy - CAAM 2095 | F | 48 | Amb a 1 | 0 | Cry j 1 | 0,1 | Cup a 1 | 2,24 |
| Italy - CAAM 2096 | F | 48 | Amb a 1 | 0 | Cry j 1 | 0 | Cup a 1 | 2,8 |
| Italy - CAAM 2097 | F | 48 | Amb a 1 | 0 | Cry j 1 | 0,08 | Cup a 1 | 1,52 |
| Italy - CAAM 2098 | M | 48 | Amb a 1 | 0 | Cry j 1 | 0,11 | Cup a 1 | 1,2 |
| Italy - CAAM 2099 | M | 48 | Amb a 1 | 0 | Cry j 1 | 0,09 | Cup a 1 | 0,43 |
| Italy - CAAM 2100 | F | 48 | Amb a 1 | 0 | Cry j 1 | 0 | Cup a 1 | 0,1 |
| Italy - CAAM 2101 | F | 47 | Amb a 1 | 0 | Cry j 1 | 1,77 | Cup a 1 | 4,15 |
| Italy - CAAM 2102 | F | 47 | Amb a 1 | 0 | Cry j 1 | 3,19 | Cup a 1 | 4,51 |
| Italy - CAAM 2103 | M | 47 | Amb a 1 | 0 | Cry j 1 | 6,53 | Cup a 1 | 8,58 |
| Italy - CAAM 2104 | F | 47 | Amb a 1 | 0 | Cry j 1 | 0 | Cup a 1 | 0,19 |
| Italy - CAAM 2105 | F | 47 | Amb a 1 | 0 | Cry j 1 | 0,68 | Cup a 1 | 2,61 |
| Italy - CAAM 2106 | F | 47 | Amb a 1 | 0 | Cry j 1 | 0,1 | Cup a 1 | 1,57 |
| Italy - CAAM 2107 | M | 47 | Amb a 1 | 0 | Cry j 1 | 2,39 | Cup a 1 | 0 |
| Italy - CAAM 2108 | F | 47 | Amb a 1 | 0 | Cry j 1 | 7,91 | Cup a 1 | 30,13 |
| Italy - CAAM 2109 | M | 47 | Amb a 1 | 0 | Cry j 1 | 5,2 | Cup a 1 | 67 |
| Italy - CAAM 2110 | M | 47 | Amb a 1 | 0 | Cry j 1 | 16,26 | Cup a 1 | 49,14 |
| Italy - CAAM 2111 | M | 47 | Amb a 1 | 0 | Cry j 1 | 13,39 | Cup a 1 | 8,38 |
| Italy - CAAM 2112 | M | 47 | Amb a 1 | 0 | Cry j 1 | 0 | Cup a 1 | 1,67 |
| Italy - CAAM 2113 | M | 47 | Amb a 1 | 0 | Cry j 1 | 0,64 | Cup a 1 | 1,82 |
| Italy - CAAM 2114 | M | 47 | Amb a 1 | 0 | Cry j 1 | 1,38 | Cup a 1 | 5,29 |
| Italy - CAAM 2115 | F | 47 | Amb a 1 | 0 | Cry j 1 | 1,98 | Cup a 1 | 13,22 |
| Italy - CAAM 2116 | M | 47 | Amb a 1 | 0 | Cry j 1 | 0 | Cup a 1 | 1,28 |
| Italy - CAAM 2117 | M | 47 | Amb a 1 | 0 | Cry j 1 | 0 | Cup a 1 | 1 |
| Italy - CAAM 2118 | M | 47 | Amb a 1 | 0 | Cry j 1 | 0,03 | Cup a 1 | 2,58 |
| Italy - CAAM 2119 | M | 47 | Amb a 1 | 0 | Cry j 1 | 1,5 | Cup a 1 | 6,38 |
| Italy - CAAM 2120 | F | 47 | Amb a 1 | 0 | Cry j 1 | 0,32 | Cup a 1 | 0,96 |
| Italy - CAAM 2121 | F | 47 | Amb a 1 | 0 | Cry j 1 | 0,17 | Cup a 1 | 2,81 |
| Italy - CAAM 2122 | M | 47 | Amb a 1 | 0 | Cry j 1 | 2,48 | Cup a 1 | 3,37 |
| Italy - CAAM 2123 | F | 47 | Amb a 1 | 0 | Cry j 1 | 0 | Cup a 1 | 3,91 |
| Italy - CAAM 2124 | M | 47 | Amb a 1 | 0 | Cry j 1 | 0,64 | Cup a 1 | 0,57 |
| Italy - CAAM 2125 | M | 47 | Amb a 1 | 0 | Cry j 1 | 0,59 | Cup a 1 | 1,76 |
| Italy - CAAM 2126 | F | 47 | Amb a 1 | 0 | Cry j 1 | 0,61 | Cup a 1 | 2,76 |
| Italy - CAAM 2127 | M | 47 | Amb a 1 | 0 | Cry j 1 | 0,18 | Cup a 1 | 2,79 |
| Italy - CAAM 2128 | F | 47 | Amb a 1 | 0 | Cry j 1 | 2,16 | Cup a 1 | 10,09 |
| Italy - CAAM 2129 | F | 47 | Amb a 1 | 0 | Cry j 1 | 51,77 | Cup a 1 | 64,45 |
| Italy - CAAM 2130 | F | 47 | Amb a 1 | 0 | Cry j 1 | 3,71 | Cup a 1 | 10,1 |
| Italy - CAAM 2131 | M | 47 | Amb a 1 | 0 | Cry j 1 | 5,87 | Cup a 1 | 4,81 |
| Italy - CAAM 2132 | M | 47 | Amb a 1 | 0 | Cry j 1 | 0,53 | Cup a 1 | 0,47 |
| Italy - CAAM 2133 | F | 47 | Amb a 1 | 0 | Cry j 1 | 7,16 | Cup a 1 | 4,92 |
| Italy - CAAM 2134 | M | 47 | Amb a 1 | 0 | Cry j 1 | 1,31 | Cup a 1 | 0 |
| Italy - CAAM 2135 | M | 47 | Amb a 1 | 0 | Cry j 1 | 5,19 | Cup a 1 | 4,59 |
| Italy - CAAM 2136 | F | 47 | Amb a 1 | 0 | Cry j 1 | 0,38 | Cup a 1 | 0,31 |
| Italy - CAAM 2137 | F | 47 | Amb a 1 | 0 | Cry j 1 | 0,78 | Cup a 1 | 4,88 |
| Italy - CAAM 2138 | M | 47 | Amb a 1 | 0 | Cry j 1 | 0,29 | Cup a 1 | 0,21 |
| Italy - CAAM 2139 | F | 47 | Amb a 1 | 0 | Cry j 1 | 0,12 | Cup a 1 | 1,28 |
| Italy - CAAM 2140 | F | 47 | Amb a 1 | 0 | Cry j 1 | 0,38 | Cup a 1 | 2,6 |
| Italy - CAAM 2141 | M | 47 | Amb a 1 | 0 | Cry j 1 | 1,23 | Cup a 1 | 5,27 |
| Italy - CAAM 2142 | F | 47 | Amb a 1 | 0 | Cry j 1 | 2,52 | Cup a 1 | 2,57 |
| Italy - CAAM 2143 | M | 47 | Amb a 1 | 0 | Cry j 1 | 0 | Cup a 1 | 1,62 |
| Italy - CAAM 2144 | F | 47 | Amb a 1 | 0 | Cry j 1 | 0 | Cup a 1 | 0,68 |
| Italy - CAAM 2145 | F | 47 | Amb a 1 | 0 | Cry j 1 | 4,73 | Cup a 1 | 20,67 |
| Italy - CAAM 2146 | M | 47 | Amb a 1 | 0 | Cry j 1 | 16,23 | Cup a 1 | 19,57 |
| Italy - CAAM 2147 | M | 47 | Amb a 1 | 0 | Cry j 1 | 7,29 | Cup a 1 | 17,98 |
| Italy - CAAM 2148 | F | 47 | Amb a 1 | 0 | Cry j 1 | 0 | Cup a 1 | 0,44 |
| Italy - CAAM 2149 | F | 47 | Amb a 1 | 0 | Cry j 1 | 0,39 | Cup a 1 | 3,62 |
| Italy - CAAM 2150 | F | 47 | Amb a 1 | 0 | Cry j 1 | 0,04 | Cup a 1 | 0 |
| Italy - CAAM 2151 | F | 47 | Amb a 1 | 0 | Cry j 1 | 0 | Cup a 1 | 0,07 |
| Italy - CAAM 2152 | F | 47 | Amb a 1 | 0 | Cry j 1 | 0 | Cup a 1 | 0,05 |
| Italy - CAAM 2153 | F | 47 | Amb a 1 | 0 | Cry j 1 | 0 | Cup a 1 | 7,5 |
| Italy - CAAM 2154 | F | 47 | Amb a 1 | 0 | Cry j 1 | 0,81 | Cup a 1 | 4,04 |
| Italy - CAAM 2155 | F | 47 | Amb a 1 | 0 | Cry j 1 | 7,75 | Cup a 1 | 25,69 |
| Italy - CAAM 2156 | M | 47 | Amb a 1 | 0 | Cry j 1 | 0,97 | Cup a 1 | 3,4 |
| Italy - CAAM 2157 | M | 47 | Amb a 1 | 0 | Cry j 1 | 0,25 | Cup a 1 | 0 |
| Italy - CAAM 2158 | F | 47 | Amb a 1 | 0 | Cry j 1 | 4,35 | Cup a 1 | 12,27 |
| Italy - CAAM 2159 | F | 47 | Amb a 1 | 0 | Cry j 1 | 0,23 | Cup a 1 | 0,44 |
| Italy - CAAM 2160 | F | 47 | Amb a 1 | 0 | Cry j 1 | 0,63 | Cup a 1 | 7,74 |
| Italy - CAAM 2161 | M | 47 | Amb a 1 | 0 | Cry j 1 | 0,06 | Cup a 1 | 0 |
| Italy - CAAM 2162 | F | 47 | Amb a 1 | 0 | Cry j 1 | 0,51 | Cup a 1 | 1,44 |
| Italy - CAAM 2163 | M | 47 | Amb a 1 | 0 | Cry j 1 | 0 | Cup a 1 | 0,36 |
| Italy - CAAM 2164 | M | 47 | Amb a 1 | 0 | Cry j 1 | 0,14 | Cup a 1 | 0,41 |
| Italy - CAAM 2165 | F | 47 | Amb a 1 | 0 | Cry j 1 | 0,28 | Cup a 1 | 0,87 |
| Italy - CAAM 2166 | M | 47 | Amb a 1 | 0 | Cry j 1 | 0,08 | Cup a 1 | 0 |
| Italy - CAAM 2167 | M | 47 | Amb a 1 | 0 | Cry j 1 | 0,17 | Cup a 1 | 0,63 |
| Italy - CAAM 2168 | F | 47 | Amb a 1 | 0 | Cry j 1 | 0,94 | Cup a 1 | 2,65 |
| Italy - CAAM 2169 | F | 47 | Amb a 1 | 0 | Cry j 1 | 0 | Cup a 1 | 2,33 |
| Italy - CAAM 2170 | F | 47 | Amb a 1 | 0 | Cry j 1 | 0,64 | Cup a 1 | 0,22 |
| Italy - CAAM 2171 | F | 47 | Amb a 1 | 0 | Cry j 1 | 1,26 | Cup a 1 | 2,72 |
| Italy - CAAM 2172 | F | 47 | Amb a 1 | 0 | Cry j 1 | 0,03 | Cup a 1 | 0,09 |
| Italy - CAAM 2173 | F | 47 | Amb a 1 | 0 | Cry j 1 | 0 | Cup a 1 | 0,27 |
| Italy - CAAM 2174 | F | 47 | Amb a 1 | 0 | Cry j 1 | 0,11 | Cup a 1 | 0,08 |
| Italy - CAAM 2175 | F | 47 | Amb a 1 | 0 | Cry j 1 | 0 | Cup a 1 | 0,55 |
| Italy - CAAM 2176 | F | 47 | Amb a 1 | 0 | Cry j 1 | 0,81 | Cup a 1 | 6,58 |
| Italy - CAAM 2177 | F | 47 | Amb a 1 | 0 | Cry j 1 | 1,44 | Cup a 1 | 11,16 |
| Italy - CAAM 2178 | F | 47 | Amb a 1 | 0 | Cry j 1 | 5,77 | Cup a 1 | 17,63 |
| Italy - CAAM 2179 | M | 47 | Amb a 1 | 0 | Cry j 1 | 5,05 | Cup a 1 | 14,1 |
| Italy - CAAM 2180 | M | 47 | Amb a 1 | 0 | Cry j 1 | 0 | Cup a 1 | 0,32 |
| Italy - CAAM 2181 | M | 47 | Amb a 1 | 0 | Cry j 1 | 0,41 | Cup a 1 | 1,99 |
| Italy - CAAM 2182 | F | 47 | Amb a 1 | 0 | Cry j 1 | 0,09 | Cup a 1 | 0,05 |
| Italy - CAAM 2183 | F | 47 | Amb a 1 | 0 | Cry j 1 | 1,07 | Cup a 1 | 6,42 |
| Italy - CAAM 2184 | F | 47 | Amb a 1 | 0 | Cry j 1 | 0 | Cup a 1 | 0,39 |
| Italy - CAAM 2185 | F | 47 | Amb a 1 | 0 | Cry j 1 | 0 | Cup a 1 | 0,35 |
| Italy - CAAM 2186 | F | 47 | Amb a 1 | 0 | Cry j 1 | 0,67 | Cup a 1 | 12,46 |
| Italy - CAAM 2187 | M | 47 | Amb a 1 | 0 | Cry j 1 | 0,03 | Cup a 1 | 0,02 |
| Italy - CAAM 2188 | M | 47 | Amb a 1 | 0 | Cry j 1 | 0,02 | Cup a 1 | 0,04 |
| Italy - CAAM 2189 | M | 47 | Amb a 1 | 0 | Cry j 1 | 0,04 | Cup a 1 | 0,71 |
| Italy - CAAM 2190 | M | 47 | Amb a 1 | 0 | Cry j 1 | 0 | Cup a 1 | 1,3 |
| Italy - CAAM 2191 | F | 47 | Amb a 1 | 0 | Cry j 1 | 1,33 | Cup a 1 | 12,97 |
| Italy - CAAM 2192 | M | 47 | Amb a 1 | 0 | Cry j 1 | 0 | Cup a 1 | 7,42 |
| Italy - CAAM 2193 | F | 47 | Amb a 1 | 0 | Cry j 1 | 0,3 | Cup a 1 | 8,18 |
| Italy - CAAM 2194 | F | 47 | Amb a 1 | 0 | Cry j 1 | 1,03 | Cup a 1 | 35,87 |
| Italy - CAAM 2195 | M | 47 | Amb a 1 | 0 | Cry j 1 | 1,38 | Cup a 1 | 9,3 |
| Italy - CAAM 2196 | F | 47 | Amb a 1 | 0 | Cry j 1 | 0,49 | Cup a 1 | 8,86 |
| Italy - CAAM 2197 | M | 47 | Amb a 1 | 0 | Cry j 1 | 3,4 | Cup a 1 | 2,25 |
| Italy - CAAM 2198 | F | 47 | Amb a 1 | 0 | Cry j 1 | 0,63 | Cup a 1 | 10,6 |
| Italy - CAAM 2199 | F | 47 | Amb a 1 | 0 | Cry j 1 | 0 | Cup a 1 | 0,14 |
| Italy - CAAM 2200 | F | 47 | Amb a 1 | 0 | Cry j 1 | 1,17 | Cup a 1 | 6,72 |
| Italy - CAAM 2201 | F | 47 | Amb a 1 | 0 | Cry j 1 | 0,94 | Cup a 1 | 13,24 |
| Italy - CAAM 2202 | F | 47 | Amb a 1 | 0 | Cry j 1 | 1,05 | Cup a 1 | 5,8 |
| Italy - CAAM 2203 | F | 47 | Amb a 1 | 0 | Cry j 1 | 2,03 | Cup a 1 | 7,43 |
| Italy - CAAM 2204 | F | 47 | Amb a 1 | 0 | Cry j 1 | 3,96 | Cup a 1 | 17,3 |
| Italy - CAAM 2205 | F | 47 | Amb a 1 | 0 | Cry j 1 | 0 | Cup a 1 | 0,47 |
| Italy - CAAM 2206 | M | 47 | Amb a 1 | 0 | Cry j 1 | 1,63 | Cup a 1 | 5,8 |
| Italy - CAAM 2207 | F | 47 | Amb a 1 | 0 | Cry j 1 | 0 | Cup a 1 | 1,1 |
| Italy - CAAM 2208 | F | 47 | Amb a 1 | 0 | Cry j 1 | 9,66 | Cup a 1 | 53,37 |
| Italy - CAAM 2209 | F | 47 | Amb a 1 | 0 | Cry j 1 | 0 | Cup a 1 | 0,52 |
| Italy - CAAM 2210 | F | 47 | Amb a 1 | 0 | Cry j 1 | 1,03 | Cup a 1 | 2,59 |
| Italy - CAAM 2211 | M | 47 | Amb a 1 | 0 | Cry j 1 | 0 | Cup a 1 | 4,22 |
| Italy - CAAM 2212 | F | 47 | Amb a 1 | 0 | Cry j 1 | 0 | Cup a 1 | 0,42 |
| Italy - CAAM 2213 | F | 47 | Amb a 1 | 0 | Cry j 1 | 2,52 | Cup a 1 | 18,93 |
| Italy - CAAM 2214 | M | 47 | Amb a 1 | 0 | Cry j 1 | 0 | Cup a 1 | 0,96 |
| Italy - CAAM 2215 | M | 47 | Amb a 1 | 0 | Cry j 1 | 4,46 | Cup a 1 | 40,45 |
| Italy - CAAM 2216 | F | 47 | Amb a 1 | 0 | Cry j 1 | 6,2 | Cup a 1 | 101 |
| Italy - CAAM 2217 | F | 47 | Amb a 1 | 0 | Cry j 1 | 0,36 | Cup a 1 | 5,54 |
| Italy - CAAM 2218 | F | 47 | Amb a 1 | 0 | Cry j 1 | 2,91 | Cup a 1 | 19,98 |
| Italy - CAAM 2219 | F | 47 | Amb a 1 | 0 | Cry j 1 | 1,39 | Cup a 1 | 17,95 |
| Italy - CAAM 2220 | F | 47 | Amb a 1 | 0 | Cry j 1 | 1,73 | Cup a 1 | 10,73 |
| Italy - CAAM 2221 | F | 47 | Amb a 1 | 0 | Cry j 1 | 0 | Cup a 1 | 0,34 |
| Italy - CAAM 2222 | M | 47 | Amb a 1 | 0 | Cry j 1 | 0 | Cup a 1 | 0,65 |
| Italy - CAAM 2223 | F | 47 | Amb a 1 | 0 | Cry j 1 | 0 | Cup a 1 | 0,12 |
| Italy - CAAM 2224 | F | 47 | Amb a 1 | 0 | Cry j 1 | 0 | Cup a 1 | 0,23 |
| Italy - CAAM 2225 | F | 47 | Amb a 1 | 0 | Cry j 1 | 0 | Cup a 1 | 0,78 |
| Italy - CAAM 2226 | M | 47 | Amb a 1 | 0 | Cry j 1 | 0 | Cup a 1 | 1,57 |
| Italy - CAAM 2227 | F | 47 | Amb a 1 | 0 | Cry j 1 | 0,06 | Cup a 1 | 6,22 |
| Italy - CAAM 2228 | F | 47 | Amb a 1 | 0 | Cry j 1 | 0 | Cup a 1 | 0,11 |
| Italy - CAAM 2229 | M | 47 | Amb a 1 | 0 | Cry j 1 | 0,39 | Cup a 1 | 1,06 |
| Italy - CAAM 2230 | M | 47 | Amb a 1 | 0 | Cry j 1 | 0 | Cup a 1 | 15,35 |
| Italy - CAAM 2231 | F | 47 | Amb a 1 | 0 | Cry j 1 | 0,8 | Cup a 1 | 18 |
| Italy - CAAM 2232 | M | 47 | Amb a 1 | 0 | Cry j 1 | 4 | Cup a 1 | 18 |
| Italy - CAAM 2233 | F | 47 | Amb a 1 | 0 | Cry j 1 | 0 | Cup a 1 | 2,2 |
| Italy - CAAM 2234 | F | 47 | Amb a 1 | 0 | Cry j 1 | 0 | Cup a 1 | 2,5 |
| Italy - CAAM 2235 | M | 47 | Amb a 1 | 0 | Cry j 1 | 2,9 | Cup a 1 | 7,4 |
| Italy - CAAM 2236 | M | 47 | Amb a 1 | 0 | Cry j 1 | 0,66 | Cup a 1 | 2,78 |
| Italy - CAAM 2237 | F | 46 | Amb a 1 | 0 | Cry j 1 | 0 | Cup a 1 | 1,06 |
| Italy - CAAM 2238 | F | 46 | Amb a 1 | 0 | Cry j 1 | 0 | Cup a 1 | 6,24 |
| Italy - CAAM 2239 | F | 46 | Amb a 1 | 0 | Cry j 1 | 0,2 | Cup a 1 | 5,41 |
| Italy - CAAM 2240 | M | 46 | Amb a 1 | 0 | Cry j 1 | 0 | Cup a 1 | 2,88 |
| Italy - CAAM 2241 | F | 46 | Amb a 1 | 0 | Cry j 1 | 0,98 | Cup a 1 | 3,77 |
| Italy - CAAM 2242 | M | 46 | Amb a 1 | 0 | Cry j 1 | 0,39 | Cup a 1 | 5,77 |
| Italy - CAAM 2243 | F | 46 | Amb a 1 | 0 | Cry j 1 | 4,77 | Cup a 1 | 6,06 |
| Italy - CAAM 2244 | F | 46 | Amb a 1 | 0 | Cry j 1 | 1,9 | Cup a 1 | 14 |
| Italy - CAAM 2245 | M | 46 | Amb a 1 | 0 | Cry j 1 | 3,02 | Cup a 1 | 5,68 |
| Italy - CAAM 2246 | M | 46 | Amb a 1 | 0 | Cry j 1 | 0,76 | Cup a 1 | 2,99 |
| Italy - CAAM 2247 | M | 46 | Amb a 1 | 0 | Cry j 1 | 0,05 | Cup a 1 | 0,81 |
| Italy - CAAM 2248 | F | 46 | Amb a 1 | 0 | Cry j 1 | 0,8 | Cup a 1 | 13 |
| Italy - CAAM 2249 | F | 46 | Amb a 1 | 0 | Cry j 1 | 0,9 | Cup a 1 | 7,01 |
| Italy - CAAM 2250 | M | 46 | Amb a 1 | 0 | Cry j 1 | 0,11 | Cup a 1 | 0,74 |
| Italy - CAAM 2251 | M | 46 | Amb a 1 | 0 | Cry j 1 | 0,36 | Cup a 1 | 0,67 |
| Italy - CAAM 2252 | F | 46 | Amb a 1 | 0 | Cry j 1 | 0,14 | Cup a 1 | 1,67 |
| Italy - CAAM 2253 | F | 46 | Amb a 1 | 0 | Cry j 1 | 5,41 | Cup a 1 | 5,74 |
| Italy - CAAM 2254 | F | 46 | Amb a 1 | 0 | Cry j 1 | 0 | Cup a 1 | 0,32 |
| Italy - CAAM 2255 | F | 46 | Amb a 1 | 0 | Cry j 1 | 0,27 | Cup a 1 | 13,48 |
| Italy - CAAM 2256 | F | 46 | Amb a 1 | 0 | Cry j 1 | 1,21 | Cup a 1 | 1,94 |
| Italy - CAAM 2257 | M | 46 | Amb a 1 | 0 | Cry j 1 | 1,62 | Cup a 1 | 2,57 |
| Italy - CAAM 2258 | F | 46 | Amb a 1 | 0 | Cry j 1 | 5,51 | Cup a 1 | 5,59 |
| Italy - CAAM 2259 | F | 46 | Amb a 1 | 0 | Cry j 1 | 0 | Cup a 1 | 0,23 |
| Italy - CAAM 2260 | F | 46 | Amb a 1 | 0 | Cry j 1 | 5,06 | Cup a 1 | 12,6 |
| Italy - CAAM 2261 | F | 46 | Amb a 1 | 0 | Cry j 1 | 1,9 | Cup a 1 | 24 |
| Italy - CAAM 2262 | F | 46 | Amb a 1 | 0 | Cry j 1 | 0,08 | Cup a 1 | 5,24 |
| Italy - CAAM 2263 | M | 46 | Amb a 1 | 0 | Cry j 1 | 2,01 | Cup a 1 | 6,68 |
| Italy - CAAM 2264 | F | 46 | Amb a 1 | 0 | Cry j 1 | 2,91 | Cup a 1 | 6,88 |
| Italy - CAAM 2265 | F | 46 | Amb a 1 | 0 | Cry j 1 | 0,68 | Cup a 1 | 0,64 |
| Italy - CAAM 2266 | F | 46 | Amb a 1 | 0 | Cry j 1 | 4,12 | Cup a 1 | 4,58 |
| Italy - CAAM 2267 | F | 46 | Amb a 1 | 0 | Cry j 1 | 0 | Cup a 1 | 0,67 |
| Italy - CAAM 2268 | M | 46 | Amb a 1 | 0 | Cry j 1 | 0,34 | Cup a 1 | 0,52 |
| Italy - CAAM 2269 | F | 46 | Amb a 1 | 0 | Cry j 1 | 2,82 | Cup a 1 | 12,92 |
| Italy - CAAM 2270 | M | 46 | Amb a 1 | 0 | Cry j 1 | 1,95 | Cup a 1 | 6,54 |
| Italy - CAAM 2271 | M | 46 | Amb a 1 | 0 | Cry j 1 | 8,88 | Cup a 1 | 7,07 |
| Italy - CAAM 2272 | F | 46 | Amb a 1 | 0 | Cry j 1 | 1,69 | Cup a 1 | 3,19 |
| Italy - CAAM 2273 | M | 46 | Amb a 1 | 0 | Cry j 1 | 0 | Cup a 1 | 0,61 |
| Italy - CAAM 2274 | F | 46 | Amb a 1 | 0 | Cry j 1 | 2,07 | Cup a 1 | 5,19 |
| Italy - CAAM 2275 | F | 46 | Amb a 1 | 0 | Cry j 1 | 0 | Cup a 1 | 0,22 |
| Italy - CAAM 2276 | M | 46 | Amb a 1 | 0 | Cry j 1 | 12,22 | Cup a 1 | 8,77 |
| Italy - CAAM 2277 | F | 46 | Amb a 1 | 0 | Cry j 1 | 0,31 | Cup a 1 | 0,51 |
| Italy - CAAM 2278 | M | 46 | Amb a 1 | 0 | Cry j 1 | 0,23 | Cup a 1 | 0,45 |
| Italy - CAAM 2279 | M | 46 | Amb a 1 | 0 | Cry j 1 | 0 | Cup a 1 | 0,15 |
| Italy - CAAM 2280 | M | 46 | Amb a 1 | 0 | Cry j 1 | 0,49 | Cup a 1 | 0,15 |
| Italy - CAAM 2281 | F | 46 | Amb a 1 | 0 | Cry j 1 | 1,1 | Cup a 1 | 2,12 |
| Italy - CAAM 2282 | M | 46 | Amb a 1 | 0 | Cry j 1 | 0,66 | Cup a 1 | 3,45 |
| Italy - CAAM 2283 | F | 46 | Amb a 1 | 0 | Cry j 1 | 0,32 | Cup a 1 | 9,33 |
| Italy - CAAM 2284 | F | 46 | Amb a 1 | 0 | Cry j 1 | 2,7 | Cup a 1 | 9,95 |
| Italy - CAAM 2285 | F | 46 | Amb a 1 | 0 | Cry j 1 | 2,28 | Cup a 1 | 8,91 |
| Italy - CAAM 2286 | F | 46 | Amb a 1 | 0 | Cry j 1 | 0 | Cup a 1 | 0,31 |
| Italy - CAAM 2287 | F | 46 | Amb a 1 | 0 | Cry j 1 | 1,08 | Cup a 1 | 7,82 |
| Italy - CAAM 2288 | F | 46 | Amb a 1 | 0 | Cry j 1 | 0,21 | Cup a 1 | 0,93 |
| Italy - CAAM 2289 | M | 46 | Amb a 1 | 0 | Cry j 1 | 0,42 | Cup a 1 | 2,91 |
| Italy - CAAM 2290 | M | 46 | Amb a 1 | 0 | Cry j 1 | 0,1 | Cup a 1 | 0,16 |
| Italy - CAAM 2291 | F | 46 | Amb a 1 | 0 | Cry j 1 | 0,1 | Cup a 1 | 0,35 |
| Italy - CAAM 2292 | M | 46 | Amb a 1 | 0 | Cry j 1 | 0,29 | Cup a 1 | 1,19 |
| Italy - CAAM 2293 | F | 46 | Amb a 1 | 0 | Cry j 1 | 0,08 | Cup a 1 | 0 |
| Italy - CAAM 2294 | F | 46 | Amb a 1 | 0 | Cry j 1 | 1,31 | Cup a 1 | 6,43 |
| Italy - CAAM 2295 | M | 46 | Amb a 1 | 0 | Cry j 1 | 0,27 | Cup a 1 | 1,61 |
| Italy - CAAM 2296 | F | 46 | Amb a 1 | 0 | Cry j 1 | 7,45 | Cup a 1 | 7,72 |
| Italy - CAAM 2297 | F | 46 | Amb a 1 | 0 | Cry j 1 | 12,1 | Cup a 1 | 32,32 |
| Italy - CAAM 2298 | M | 46 | Amb a 1 | 0 | Cry j 1 | 0,58 | Cup a 1 | 9,62 |
| Italy - CAAM 2299 | F | 46 | Amb a 1 | 0 | Cry j 1 | 0 | Cup a 1 | 1,16 |
| Italy - CAAM 2300 | M | 46 | Amb a 1 | 0 | Cry j 1 | 2,97 | Cup a 1 | 8,48 |
| Italy - CAAM 2301 | F | 46 | Amb a 1 | 0 | Cry j 1 | 0 | Cup a 1 | 0,05 |
| Italy - CAAM 2302 | F | 46 | Amb a 1 | 0 | Cry j 1 | 0 | Cup a 1 | 0,46 |
| Italy - CAAM 2303 | F | 46 | Amb a 1 | 0 | Cry j 1 | 0 | Cup a 1 | 0,03 |
| Italy - CAAM 2304 | F | 46 | Amb a 1 | 0 | Cry j 1 | 0,02 | Cup a 1 | 0,77 |
| Italy - CAAM 2305 | F | 46 | Amb a 1 | 0 | Cry j 1 | 0,45 | Cup a 1 | 1,84 |
| Italy - CAAM 2306 | M | 46 | Amb a 1 | 0 | Cry j 1 | 0 | Cup a 1 | 2,74 |
| Italy - CAAM 2307 | M | 46 | Amb a 1 | 0 | Cry j 1 | 2,75 | Cup a 1 | 11,77 |
| Italy - CAAM 2308 | M | 46 | Amb a 1 | 0 | Cry j 1 | 0 | Cup a 1 | 0,11 |
| Italy - CAAM 2309 | F | 46 | Amb a 1 | 0 | Cry j 1 | 0,09 | Cup a 1 | 1,84 |
| Italy - CAAM 2310 | M | 46 | Amb a 1 | 0 | Cry j 1 | 1,71 | Cup a 1 | 4,98 |
| Italy - CAAM 2311 | M | 46 | Amb a 1 | 0 | Cry j 1 | 18,13 | Cup a 1 | 26,22 |
| Italy - CAAM 2312 | F | 46 | Amb a 1 | 0 | Cry j 1 | 0,89 | Cup a 1 | 5,7 |
| Italy - CAAM 2313 | F | 46 | Amb a 1 | 0 | Cry j 1 | 0 | Cup a 1 | 1,48 |
| Italy - CAAM 2314 | M | 46 | Amb a 1 | 0 | Cry j 1 | 0,09 | Cup a 1 | 0,47 |
| Italy - CAAM 2315 | M | 46 | Amb a 1 | 0 | Cry j 1 | 2,41 | Cup a 1 | 5,37 |
| Italy - CAAM 2316 | F | 46 | Amb a 1 | 0 | Cry j 1 | 2,65 | Cup a 1 | 6,33 |
| Italy - CAAM 2317 | F | 46 | Amb a 1 | 0 | Cry j 1 | 0,03 | Cup a 1 | 0,21 |
| Italy - CAAM 2318 | F | 46 | Amb a 1 | 0 | Cry j 1 | 0,11 | Cup a 1 | 0,54 |
| Italy - CAAM 2319 | M | 46 | Amb a 1 | 0 | Cry j 1 | 0 | Cup a 1 | 0,05 |
| Italy - CAAM 2320 | M | 46 | Amb a 1 | 0 | Cry j 1 | 2,44 | Cup a 1 | 3,94 |
| Italy - CAAM 2321 | F | 46 | Amb a 1 | 0 | Cry j 1 | 2,44 | Cup a 1 | 6,2 |
| Italy - CAAM 2322 | F | 46 | Amb a 1 | 0 | Cry j 1 | 0 | Cup a 1 | 2,98 |
| Italy - CAAM 2323 | F | 46 | Amb a 1 | 0 | Cry j 1 | 0 | Cup a 1 | 1,73 |
| Italy - CAAM 2324 | F | 46 | Amb a 1 | 0 | Cry j 1 | 0,12 | Cup a 1 | 1,54 |
| Italy - CAAM 2325 | M | 46 | Amb a 1 | 0 | Cry j 1 | 0 | Cup a 1 | 0,05 |
| Italy - CAAM 2326 | F | 46 | Amb a 1 | 0 | Cry j 1 | 0 | Cup a 1 | 2,52 |
| Italy - CAAM 2327 | M | 46 | Amb a 1 | 0 | Cry j 1 | 8,39 | Cup a 1 | 18,32 |
| Italy - CAAM 2328 | F | 46 | Amb a 1 | 0 | Cry j 1 | 0 | Cup a 1 | 0,59 |
| Italy - CAAM 2329 | F | 46 | Amb a 1 | 0 | Cry j 1 | 0 | Cup a 1 | 0,47 |
| Italy - CAAM 2330 | F | 46 | Amb a 1 | 0 | Cry j 1 | 0,3 | Cup a 1 | 1,65 |
| Italy - CAAM 2331 | F | 46 | Amb a 1 | 0 | Cry j 1 | 0,83 | Cup a 1 | 6,45 |
| Italy - CAAM 2332 | M | 46 | Amb a 1 | 0 | Cry j 1 | 1,72 | Cup a 1 | 9,41 |
| Italy - CAAM 2333 | F | 46 | Amb a 1 | 0 | Cry j 1 | 0 | Cup a 1 | 0,55 |
| Italy - CAAM 2334 | F | 46 | Amb a 1 | 0 | Cry j 1 | 0,02 | Cup a 1 | 0,13 |
| Italy - CAAM 2335 | M | 46 | Amb a 1 | 0 | Cry j 1 | 0 | Cup a 1 | 2,54 |
| Italy - CAAM 2336 | F | 46 | Amb a 1 | 0 | Cry j 1 | 0,48 | Cup a 1 | 1,44 |
| Italy - CAAM 2337 | F | 46 | Amb a 1 | 0 | Cry j 1 | 0,14 | Cup a 1 | 2,85 |
| Italy - CAAM 2338 | M | 46 | Amb a 1 | 0 | Cry j 1 | 0,06 | Cup a 1 | 2,21 |
| Italy - CAAM 2339 | F | 46 | Amb a 1 | 0 | Cry j 1 | 0,62 | Cup a 1 | 1,24 |
| Italy - CAAM 2340 | F | 46 | Amb a 1 | 0 | Cry j 1 | 0 | Cup a 1 | 5,87 |
| Italy - CAAM 2341 | M | 46 | Amb a 1 | 0 | Cry j 1 | 2,54 | Cup a 1 | 8,05 |
| Italy - CAAM 2342 | F | 46 | Amb a 1 | 0 | Cry j 1 | 0 | Cup a 1 | 0,34 |
| Italy - CAAM 2343 | F | 46 | Amb a 1 | 0 | Cry j 1 | 0,04 | Cup a 1 | 3,19 |
| Italy - CAAM 2344 | M | 46 | Amb a 1 | 0 | Cry j 1 | 0 | Cup a 1 | 0,8 |
| Italy - CAAM 2345 | M | 46 | Amb a 1 | 0 | Cry j 1 | 0,11 | Cup a 1 | 1,23 |
| Italy - CAAM 2346 | F | 46 | Amb a 1 | 0 | Cry j 1 | 0,13 | Cup a 1 | 2,11 |
| Italy - CAAM 2347 | M | 46 | Amb a 1 | 0 | Cry j 1 | 0 | Cup a 1 | 0,21 |
| Italy - CAAM 2348 | F | 46 | Amb a 1 | 0 | Cry j 1 | 0,74 | Cup a 1 | 17,98 |
| Italy - CAAM 2349 | F | 46 | Amb a 1 | 0 | Cry j 1 | 0 | Cup a 1 | 1,83 |
| Italy - CAAM 2350 | F | 46 | Amb a 1 | 0 | Cry j 1 | 0 | Cup a 1 | 0,26 |
| Italy - CAAM 2351 | F | 46 | Amb a 1 | 0 | Cry j 1 | 0,6 | Cup a 1 | 2,73 |
| Italy - CAAM 2352 | F | 46 | Amb a 1 | 0 | Cry j 1 | 1,54 | Cup a 1 | 6,83 |
| Italy - CAAM 2353 | M | 46 | Amb a 1 | 0 | Cry j 1 | 0 | Cup a 1 | 0,53 |
| Italy - CAAM 2354 | M | 46 | Amb a 1 | 0 | Cry j 1 | 2,33 | Cup a 1 | 16,58 |
| Italy - CAAM 2355 | F | 46 | Amb a 1 | 0 | Cry j 1 | 0,3 | Cup a 1 | 17 |
| Italy - CAAM 2356 | M | 46 | Amb a 1 | 0 | Cry j 1 | 1,31 | Cup a 1 | 11,29 |
| Italy - CAAM 2357 | F | 46 | Amb a 1 | 0 | Cry j 1 | 0,2 | Cup a 1 | 0,62 |
| Italy - CAAM 2358 | F | 46 | Amb a 1 | 0 | Cry j 1 | 0 | Cup a 1 | 0,64 |
| Italy - CAAM 2359 | F | 46 | Amb a 1 | 0 | Cry j 1 | 2,33 | Cup a 1 | 17,67 |
| Italy - CAAM 2360 | M | 46 | Amb a 1 | 0 | Cry j 1 | 5,29 | Cup a 1 | 15,05 |
| Italy - CAAM 2361 | F | 46 | Amb a 1 | 0 | Cry j 1 | 0,51 | Cup a 1 | 4,72 |
| Italy - CAAM 2362 | F | 46 | Amb a 1 | 0 | Cry j 1 | 0,72 | Cup a 1 | 12,83 |
| Italy - CAAM 2363 | F | 46 | Amb a 1 | 0 | Cry j 1 | 0,73 | Cup a 1 | 3,88 |
| Italy - CAAM 2364 | F | 46 | Amb a 1 | 0 | Cry j 1 | 0 | Cup a 1 | 0,18 |
| Italy - CAAM 2365 | F | 46 | Amb a 1 | 0 | Cry j 1 | 0 | Cup a 1 | 0,05 |
| Italy - CAAM 2366 | F | 46 | Amb a 1 | 0 | Cry j 1 | 0,43 | Cup a 1 | 6,99 |
| Italy - CAAM 2367 | F | 46 | Amb a 1 | 0 | Cry j 1 | 0 | Cup a 1 | 0,34 |
| Italy - CAAM 2368 | M | 46 | Amb a 1 | 0 | Cry j 1 | 0,09 | Cup a 1 | 0,08 |
| Italy - CAAM 2369 | F | 46 | Amb a 1 | 0 | Cry j 1 | 0,19 | Cup a 1 | 3,06 |
| Italy - CAAM 2370 | F | 46 | Amb a 1 | 0 | Cry j 1 | 7,09 | Cup a 1 | 10,14 |
| Italy - CAAM 2371 | F | 46 | Amb a 1 | 0 | Cry j 1 | 4,3 | Cup a 1 | 19,51 |
| Italy - CAAM 2372 | F | 46 | Amb a 1 | 0 | Cry j 1 | 0 | Cup a 1 | 5,58 |
| Italy - CAAM 2373 | F | 46 | Amb a 1 | 0 | Cry j 1 | 0,2 | Cup a 1 | 1,7 |
| Italy - CAAM 2374 | F | 46 | Amb a 1 | 0 | Cry j 1 | 1,48 | Cup a 1 | 4,16 |
| Italy - CAAM 2375 | F | 46 | Amb a 1 | 0 | Cry j 1 | 0 | Cup a 1 | 0,81 |
| Italy - CAAM 2376 | F | 46 | Amb a 1 | 0 | Cry j 1 | 0 | Cup a 1 | 0,27 |
| Italy - CAAM 2377 | F | 46 | Amb a 1 | 0 | Cry j 1 | 4,59 | Cup a 1 | 17,81 |
| Italy - CAAM 2378 | M | 46 | Amb a 1 | 0 | Cry j 1 | 2,57 | Cup a 1 | 14,36 |
| Italy - CAAM 2379 | M | 46 | Amb a 1 | 0 | Cry j 1 | 0 | Cup a 1 | 0,5 |
| Italy - CAAM 2380 | F | 46 | Amb a 1 | 0 | Cry j 1 | 0 | Cup a 1 | 1,71 |
| Italy - CAAM 2381 | F | 46 | Amb a 1 | 0 | Cry j 1 | 0,16 | Cup a 1 | 5,16 |
| Italy - CAAM 2382 | F | 46 | Amb a 1 | 0 | Cry j 1 | 2,02 | Cup a 1 | 8,92 |
| Italy - CAAM 2383 | F | 46 | Amb a 1 | 0 | Cry j 1 | 2,84 | Cup a 1 | 32,32 |
| Italy - CAAM 2384 | F | 46 | Amb a 1 | 0 | Cry j 1 | 4,77 | Cup a 1 | 12,56 |
| Italy - CAAM 2385 | M | 46 | Amb a 1 | 0 | Cry j 1 | 1,88 | Cup a 1 | 16,81 |
| Italy - CAAM 2386 | M | 46 | Amb a 1 | 0 | Cry j 1 | 0,99 | Cup a 1 | 5,25 |
| Italy - CAAM 2387 | F | 46 | Amb a 1 | 0 | Cry j 1 | 2,92 | Cup a 1 | 40,55 |
| Italy - CAAM 2388 | M | 46 | Amb a 1 | 0 | Cry j 1 | 0,22 | Cup a 1 | 0,39 |
| Italy - CAAM 2389 | F | 46 | Amb a 1 | 0 | Cry j 1 | 0,07 | Cup a 1 | 3,86 |
| Italy - CAAM 2390 | F | 46 | Amb a 1 | 0 | Cry j 1 | 0,89 | Cup a 1 | 2,98 |
| Italy - CAAM 2391 | F | 46 | Amb a 1 | 0 | Cry j 1 | 0 | Cup a 1 | 0,62 |
| Italy - CAAM 2392 | F | 46 | Amb a 1 | 0 | Cry j 1 | 0 | Cup a 1 | 0,66 |
| Italy - CAAM 2393 | F | 46 | Amb a 1 | 0 | Cry j 1 | 0,55 | Cup a 1 | 6,47 |
| Italy - CAAM 2394 | M | 46 | Amb a 1 | 0 | Cry j 1 | 0,31 | Cup a 1 | 0,84 |
| Italy - CAAM 2395 | F | 46 | Amb a 1 | 0 | Cry j 1 | 0 | Cup a 1 | 0,26 |
| Italy - CAAM 2396 | M | 46 | Amb a 1 | 0 | Cry j 1 | 0 | Cup a 1 | 1,4 |
| Italy - CAAM 2397 | F | 46 | Amb a 1 | 0 | Cry j 1 | 3,67 | Cup a 1 | 8,72 |
| Italy - CAAM 2398 | F | 46 | Amb a 1 | 0 | Cry j 1 | 0,06 | Cup a 1 | 0,25 |
| Italy - CAAM 2399 | M | 46 | Amb a 1 | 0 | Cry j 1 | 0 | Cup a 1 | 0,07 |
| Italy - CAAM 2400 | M | 46 | Amb a 1 | 0 | Cry j 1 | 2,07 | Cup a 1 | 14,65 |
| Italy - CAAM 2401 | M | 46 | Amb a 1 | 0 | Cry j 1 | 2,73 | Cup a 1 | 27,6 |
| Italy - CAAM 2402 | F | 46 | Amb a 1 | 0 | Cry j 1 | 0,7 | Cup a 1 | 1,63 |
| Italy - CAAM 2403 | M | 46 | Amb a 1 | 0 | Cry j 1 | 0 | Cup a 1 | 0,94 |
| Italy - CAAM 2404 | F | 46 | Amb a 1 | 0 | Cry j 1 | 0,5 | Cup a 1 | 5,4 |
| Italy - CAAM 2405 | F | 45 | Amb a 1 | 0 | Cry j 1 | 2,24 | Cup a 1 | 10,22 |
| Italy - CAAM 2406 | F | 45 | Amb a 1 | 0 | Cry j 1 | 0 | Cup a 1 | 0,07 |
| Italy - CAAM 2407 | F | 45 | Amb a 1 | 0 | Cry j 1 | 0,04 | Cup a 1 | 0,32 |
| Italy - CAAM 2408 | M | 45 | Amb a 1 | 0 | Cry j 1 | 2,48 | Cup a 1 | 10,25 |
| Italy - CAAM 2409 | F | 45 | Amb a 1 | 0 | Cry j 1 | 0 | Cup a 1 | 1,37 |
| Italy - CAAM 2410 | F | 45 | Amb a 1 | 0 | Cry j 1 | 0 | Cup a 1 | 2,25 |
| Italy - CAAM 2411 | M | 45 | Amb a 1 | 0 | Cry j 1 | 0,4 | Cup a 1 | 2,64 |
| Italy - CAAM 2412 | F | 45 | Amb a 1 | 0 | Cry j 1 | 0,74 | Cup a 1 | 1,34 |
| Italy - CAAM 2413 | F | 45 | Amb a 1 | 0 | Cry j 1 | 0,18 | Cup a 1 | 1,06 |
| Italy - CAAM 2414 | F | 45 | Amb a 1 | 0 | Cry j 1 | 0 | Cup a 1 | 1,03 |
| Italy - CAAM 2415 | M | 45 | Amb a 1 | 0 | Cry j 1 | 2 | Cup a 1 | 5,14 |
| Italy - CAAM 2416 | M | 45 | Amb a 1 | 0 | Cry j 1 | 1,16 | Cup a 1 | 4,91 |
| Italy - CAAM 2417 | M | 45 | Amb a 1 | 0 | Cry j 1 | 2,76 | Cup a 1 | 3,95 |
| Italy - CAAM 2418 | F | 45 | Amb a 1 | 0 | Cry j 1 | 8 | Cup a 1 | 2,84 |
| Italy - CAAM 2419 | F | 45 | Amb a 1 | 0 | Cry j 1 | 0 | Cup a 1 | 1,16 |
| Italy - CAAM 2420 | F | 45 | Amb a 1 | 0 | Cry j 1 | 1,44 | Cup a 1 | 0,39 |
| Italy - CAAM 2421 | M | 45 | Amb a 1 | 0 | Cry j 1 | 0 | Cup a 1 | 4 |
| Italy - CAAM 2422 | M | 45 | Amb a 1 | 0 | Cry j 1 | 0 | Cup a 1 | 1,1 |
| Italy - CAAM 2423 | F | 45 | Amb a 1 | 0 | Cry j 1 | 0 | Cup a 1 | 2,2 |
| Italy - CAAM 2424 | F | 45 | Amb a 1 | 0 | Cry j 1 | 0 | Cup a 1 | 0,81 |
| Italy - CAAM 2425 | F | 45 | Amb a 1 | 0 | Cry j 1 | 0 | Cup a 1 | 2,99 |
| Italy - CAAM 2426 | F | 45 | Amb a 1 | 0 | Cry j 1 | 0 | Cup a 1 | 3,82 |
| Italy - CAAM 2427 | F | 45 | Amb a 1 | 0 | Cry j 1 | 0 | Cup a 1 | 0,21 |
| Italy - CAAM 2428 | F | 45 | Amb a 1 | 0 | Cry j 1 | 1,78 | Cup a 1 | 0,78 |
| Italy - CAAM 2429 | F | 45 | Amb a 1 | 0 | Cry j 1 | 0,11 | Cup a 1 | 0,67 |
| Italy - CAAM 2430 | F | 45 | Amb a 1 | 0 | Cry j 1 | 3,39 | Cup a 1 | 29,63 |
| Italy - CAAM 2431 | M | 45 | Amb a 1 | 0 | Cry j 1 | 13,93 | Cup a 1 | 22,99 |
| Italy - CAAM 2432 | F | 45 | Amb a 1 | 0 | Cry j 1 | 0,14 | Cup a 1 | 0,37 |
| Italy - CAAM 2433 | M | 45 | Amb a 1 | 0 | Cry j 1 | 2,02 | Cup a 1 | 4,09 |
| Italy - CAAM 2434 | F | 45 | Amb a 1 | 0 | Cry j 1 | 0,09 | Cup a 1 | 0,18 |
| Italy - CAAM 2435 | F | 45 | Amb a 1 | 0 | Cry j 1 | 0,32 | Cup a 1 | 0,47 |
| Italy - CAAM 2436 | F | 45 | Amb a 1 | 0 | Cry j 1 | 0 | Cup a 1 | 0,11 |
| Italy - CAAM 2437 | F | 45 | Amb a 1 | 0 | Cry j 1 | 6,83 | Cup a 1 | 21,2 |
| Italy - CAAM 2438 | F | 45 | Amb a 1 | 0 | Cry j 1 | 2,15 | Cup a 1 | 4,05 |
| Italy - CAAM 2439 | F | 45 | Amb a 1 | 0 | Cry j 1 | 1,32 | Cup a 1 | 0 |
| Italy - CAAM 2440 | F | 45 | Amb a 1 | 0 | Cry j 1 | 0,26 | Cup a 1 | 0,58 |
| Italy - CAAM 2441 | F | 45 | Amb a 1 | 0 | Cry j 1 | 0,2 | Cup a 1 | 0,7 |
| Italy - CAAM 2442 | F | 45 | Amb a 1 | 0 | Cry j 1 | 6,89 | Cup a 1 | 3,02 |
| Italy - CAAM 2443 | F | 45 | Amb a 1 | 0 | Cry j 1 | 0,19 | Cup a 1 | 0,27 |
| Italy - CAAM 2444 | M | 45 | Amb a 1 | 0 | Cry j 1 | 3,99 | Cup a 1 | 3,03 |
| Italy - CAAM 2445 | F | 45 | Amb a 1 | 0 | Cry j 1 | 0,21 | Cup a 1 | 0,24 |
| Italy - CAAM 2446 | F | 45 | Amb a 1 | 0 | Cry j 1 | 11,53 | Cup a 1 | 8,76 |
| Italy - CAAM 2447 | M | 45 | Amb a 1 | 0 | Cry j 1 | 0 | Cup a 1 | 0,61 |
| Italy - CAAM 2448 | M | 45 | Amb a 1 | 0 | Cry j 1 | 0 | Cup a 1 | 0,27 |
| Italy - CAAM 2449 | F | 45 | Amb a 1 | 0 | Cry j 1 | 0,76 | Cup a 1 | 1,28 |
| Italy - CAAM 2450 | F | 45 | Amb a 1 | 0 | Cry j 1 | 0,22 | Cup a 1 | 0,47 |
| Italy - CAAM 2451 | M | 45 | Amb a 1 | 0 | Cry j 1 | 0 | Cup a 1 | 0,66 |
| Italy - CAAM 2452 | F | 45 | Amb a 1 | 0 | Cry j 1 | 0 | Cup a 1 | 4,07 |
| Italy - CAAM 2453 | F | 45 | Amb a 1 | 0 | Cry j 1 | 1,04 | Cup a 1 | 14,05 |
| Italy - CAAM 2454 | F | 45 | Amb a 1 | 0 | Cry j 1 | 5,03 | Cup a 1 | 12,29 |
| Italy - CAAM 2455 | F | 45 | Amb a 1 | 0 | Cry j 1 | 0 | Cup a 1 | 0,61 |
| Italy - CAAM 2456 | F | 45 | Amb a 1 | 0 | Cry j 1 | 1,19 | Cup a 1 | 1,83 |
| Italy - CAAM 2457 | M | 45 | Amb a 1 | 0 | Cry j 1 | 0,18 | Cup a 1 | 0,62 |
| Italy - CAAM 2458 | F | 45 | Amb a 1 | 0 | Cry j 1 | 0,23 | Cup a 1 | 1,87 |
| Italy - CAAM 2459 | M | 45 | Amb a 1 | 0 | Cry j 1 | 3,58 | Cup a 1 | 17,34 |
| Italy - CAAM 2460 | F | 45 | Amb a 1 | 0 | Cry j 1 | 13,39 | Cup a 1 | 24,22 |
| Italy - CAAM 2461 | M | 45 | Amb a 1 | 0 | Cry j 1 | 0 | Cup a 1 | 0,05 |
| Italy - CAAM 2462 | F | 45 | Amb a 1 | 0 | Cry j 1 | 0 | Cup a 1 | 0,41 |
| Italy - CAAM 2463 | F | 45 | Amb a 1 | 0 | Cry j 1 | 0,95 | Cup a 1 | 3,72 |
| Italy - CAAM 2464 | M | 45 | Amb a 1 | 0 | Cry j 1 | 19,73 | Cup a 1 | 84,95 |
| Italy - CAAM 2465 | M | 45 | Amb a 1 | 0 | Cry j 1 | 0,37 | Cup a 1 | 6,16 |
| Italy - CAAM 2466 | F | 45 | Amb a 1 | 0 | Cry j 1 | 0,15 | Cup a 1 | 4,34 |
| Italy - CAAM 2467 | F | 45 | Amb a 1 | 0 | Cry j 1 | 0,06 | Cup a 1 | 0,64 |
| Italy - CAAM 2468 | M | 45 | Amb a 1 | 0 | Cry j 1 | 4,99 | Cup a 1 | 6,24 |
| Italy - CAAM 2469 | M | 45 | Amb a 1 | 0 | Cry j 1 | 15,9 | Cup a 1 | 13,5 |
| Italy - CAAM 2470 | M | 45 | Amb a 1 | 0 | Cry j 1 | 0,26 | Cup a 1 | 4,64 |
| Italy - CAAM 2471 | M | 45 | Amb a 1 | 0 | Cry j 1 | 0,61 | Cup a 1 | 3,31 |
| Italy - CAAM 2472 | F | 45 | Amb a 1 | 0 | Cry j 1 | 0,87 | Cup a 1 | 2 |
| Italy - CAAM 2473 | M | 45 | Amb a 1 | 0 | Cry j 1 | 6,91 | Cup a 1 | 8,39 |
| Italy - CAAM 2474 | F | 45 | Amb a 1 | 0 | Cry j 1 | 0,91 | Cup a 1 | 2,77 |
| Italy - CAAM 2475 | F | 45 | Amb a 1 | 0 | Cry j 1 | 0 | Cup a 1 | 1,03 |
| Italy - CAAM 2476 | M | 45 | Amb a 1 | 0 | Cry j 1 | 2,42 | Cup a 1 | 9,32 |
| Italy - CAAM 2477 | M | 45 | Amb a 1 | 0 | Cry j 1 | 0 | Cup a 1 | 0,7 |
| Italy - CAAM 2478 | M | 45 | Amb a 1 | 0 | Cry j 1 | 0,06 | Cup a 1 | 0,75 |
| Italy - CAAM 2479 | F | 45 | Amb a 1 | 0 | Cry j 1 | 0 | Cup a 1 | 0,13 |
| Italy - CAAM 2480 | M | 45 | Amb a 1 | 0 | Cry j 1 | 0 | Cup a 1 | 1,41 |
| Italy - CAAM 2481 | M | 45 | Amb a 1 | 0 | Cry j 1 | 0,26 | Cup a 1 | 0,21 |
| Italy - CAAM 2482 | M | 45 | Amb a 1 | 0 | Cry j 1 | 0 | Cup a 1 | 0,5 |
| Italy - CAAM 2483 | F | 45 | Amb a 1 | 0 | Cry j 1 | 2,99 | Cup a 1 | 3,61 |
| Italy - CAAM 2484 | M | 45 | Amb a 1 | 0 | Cry j 1 | 0,75 | Cup a 1 | 1,35 |
| Italy - CAAM 2485 | M | 45 | Amb a 1 | 0 | Cry j 1 | 0 | Cup a 1 | 2,53 |
| Italy - CAAM 2486 | M | 45 | Amb a 1 | 0 | Cry j 1 | 0 | Cup a 1 | 1,41 |
| Italy - CAAM 2487 | F | 45 | Amb a 1 | 0 | Cry j 1 | 0,41 | Cup a 1 | 2,72 |
| Italy - CAAM 2488 | F | 45 | Amb a 1 | 0 | Cry j 1 | 0,1 | Cup a 1 | 1,68 |
| Italy - CAAM 2489 | M | 45 | Amb a 1 | 0 | Cry j 1 | 1,24 | Cup a 1 | 4,35 |
| Italy - CAAM 2490 | F | 45 | Amb a 1 | 0 | Cry j 1 | 0 | Cup a 1 | 1,42 |
| Italy - CAAM 2491 | F | 45 | Amb a 1 | 0 | Cry j 1 | 1,54 | Cup a 1 | 5,16 |
| Italy - CAAM 2492 | F | 45 | Amb a 1 | 0 | Cry j 1 | 0,58 | Cup a 1 | 0,99 |
| Italy - CAAM 2493 | F | 45 | Amb a 1 | 0 | Cry j 1 | 0,37 | Cup a 1 | 0,78 |
| Italy - CAAM 2494 | M | 45 | Amb a 1 | 0 | Cry j 1 | 0 | Cup a 1 | 0,86 |
| Italy - CAAM 2495 | F | 45 | Amb a 1 | 0 | Cry j 1 | 2,8 | Cup a 1 | 11,56 |
| Italy - CAAM 2496 | F | 45 | Amb a 1 | 0 | Cry j 1 | 3,11 | Cup a 1 | 11,86 |
| Italy - CAAM 2497 | M | 45 | Amb a 1 | 0 | Cry j 1 | 2,9 | Cup a 1 | 7,79 |
| Italy - CAAM 2498 | F | 45 | Amb a 1 | 0 | Cry j 1 | 0,04 | Cup a 1 | 0,42 |
| Italy - CAAM 2499 | F | 45 | Amb a 1 | 0 | Cry j 1 | 1,26 | Cup a 1 | 2,72 |
| Italy - CAAM 2500 | F | 45 | Amb a 1 | 0 | Cry j 1 | 3,84 | Cup a 1 | 17,5 |
| Italy - CAAM 2501 | F | 45 | Amb a 1 | 0 | Cry j 1 | 2,01 | Cup a 1 | 10,65 |
| Italy - CAAM 2502 | F | 45 | Amb a 1 | 0 | Cry j 1 | 0,6 | Cup a 1 | 6,56 |
| Italy - CAAM 2503 | F | 45 | Amb a 1 | 0 | Cry j 1 | 0,14 | Cup a 1 | 0,21 |
| Italy - CAAM 2504 | F | 45 | Amb a 1 | 0 | Cry j 1 | 0,75 | Cup a 1 | 0,96 |
| Italy - CAAM 2505 | F | 45 | Amb a 1 | 0 | Cry j 1 | 0,07 | Cup a 1 | 1,36 |
| Italy - CAAM 2506 | F | 45 | Amb a 1 | 0 | Cry j 1 | 0,23 | Cup a 1 | 2,92 |
| Italy - CAAM 2507 | M | 45 | Amb a 1 | 0 | Cry j 1 | 0 | Cup a 1 | 0,73 |
| Italy - CAAM 2508 | F | 45 | Amb a 1 | 0 | Cry j 1 | 12,71 | Cup a 1 | 62,61 |
| Italy - CAAM 2509 | M | 45 | Amb a 1 | 0 | Cry j 1 | 0 | Cup a 1 | 0,1 |
| Italy - CAAM 2510 | F | 45 | Amb a 1 | 0 | Cry j 1 | 0,47 | Cup a 1 | 4,42 |
| Italy - CAAM 2511 | F | 45 | Amb a 1 | 0 | Cry j 1 | 2,61 | Cup a 1 | 22,56 |
| Italy - CAAM 2512 | M | 45 | Amb a 1 | 0 | Cry j 1 | 0,4 | Cup a 1 | 0 |
| Italy - CAAM 2513 | M | 45 | Amb a 1 | 0 | Cry j 1 | 1,54 | Cup a 1 | 7,84 |
| Italy - CAAM 2514 | F | 45 | Amb a 1 | 0 | Cry j 1 | 7,95 | Cup a 1 | 27,04 |
| Italy - CAAM 2515 | F | 45 | Amb a 1 | 0 | Cry j 1 | 0,14 | Cup a 1 | 1,48 |
| Italy - CAAM 2516 | M | 45 | Amb a 1 | 0 | Cry j 1 | 0 | Cup a 1 | 2,87 |
| Italy - CAAM 2517 | F | 45 | Amb a 1 | 0 | Cry j 1 | 0,4 | Cup a 1 | 6,47 |
| Italy - CAAM 2518 | F | 45 | Amb a 1 | 0 | Cry j 1 | 0,34 | Cup a 1 | 15,14 |
| Italy - CAAM 2519 | F | 45 | Amb a 1 | 0 | Cry j 1 | 0,64 | Cup a 1 | 18,4 |
| Italy - CAAM 2520 | F | 45 | Amb a 1 | 0 | Cry j 1 | 0,05 | Cup a 1 | 0,77 |
| Italy - CAAM 2521 | F | 45 | Amb a 1 | 0 | Cry j 1 | 0 | Cup a 1 | 7 |
| Italy - CAAM 2522 | F | 45 | Amb a 1 | 0 | Cry j 1 | 0,56 | Cup a 1 | 5,79 |
| Italy - CAAM 2523 | M | 45 | Amb a 1 | 0 | Cry j 1 | 0,45 | Cup a 1 | 4,74 |
| Italy - CAAM 2524 | M | 45 | Amb a 1 | 0 | Cry j 1 | 0 | Cup a 1 | 1,58 |
| Italy - CAAM 2525 | M | 45 | Amb a 1 | 0 | Cry j 1 | 1,91 | Cup a 1 | 7 |
| Italy - CAAM 2526 | F | 45 | Amb a 1 | 0 | Cry j 1 | 0,61 | Cup a 1 | 1,61 |
| Italy - CAAM 2527 | F | 45 | Amb a 1 | 0 | Cry j 1 | 0 | Cup a 1 | 2,66 |
| Italy - CAAM 2528 | F | 45 | Amb a 1 | 0 | Cry j 1 | 2,15 | Cup a 1 | 31,27 |
| Italy - CAAM 2529 | F | 45 | Amb a 1 | 0 | Cry j 1 | 4,78 | Cup a 1 | 19,78 |
| Italy - CAAM 2530 | M | 45 | Amb a 1 | 0 | Cry j 1 | 0,39 | Cup a 1 | 5,04 |
| Italy - CAAM 2531 | F | 45 | Amb a 1 | 0 | Cry j 1 | 0,21 | Cup a 1 | 1,71 |
| Italy - CAAM 2532 | F | 45 | Amb a 1 | 0 | Cry j 1 | 0 | Cup a 1 | 0,07 |
| Italy - CAAM 2533 | F | 45 | Amb a 1 | 0 | Cry j 1 | 0 | Cup a 1 | 1,19 |
| Italy - CAAM 2534 | M | 45 | Amb a 1 | 0 | Cry j 1 | 0,45 | Cup a 1 | 3,31 |
| Italy - CAAM 2535 | F | 45 | Amb a 1 | 0 | Cry j 1 | 0 | Cup a 1 | 3,5 |
| Italy - CAAM 2536 | M | 45 | Amb a 1 | 0 | Cry j 1 | 2,59 | Cup a 1 | 15,48 |
| Italy - CAAM 2537 | F | 45 | Amb a 1 | 0 | Cry j 1 | 1,2 | Cup a 1 | 11 |
| Italy - CAAM 2538 | F | 45 | Amb a 1 | 0 | Cry j 1 | 3,84 | Cup a 1 | 3,52 |
| Italy - CAAM 2539 | M | 45 | Amb a 1 | 0 | Cry j 1 | 3,5 | Cup a 1 | 22 |
| Italy - CAAM 2540 | M | 45 | Amb a 1 | 0 | Cry j 1 | 0 | Cup a 1 | 3,4 |
| Italy - CAAM 2541 | M | 45 | Amb a 1 | 0 | Cry j 1 | 0,08 | Cup a 1 | 0,87 |
| Italy - CAAM 2542 | F | 45 | Amb a 1 | 0 | Cry j 1 | 0 | Cup a 1 | 0,15 |
| Italy - CAAM 2543 | F | 45 | Amb a 1 | 0 | Cry j 1 | 0,19 | Cup a 1 | 13,28 |
| Italy - CAAM 2544 | M | 45 | Amb a 1 | 0 | Cry j 1 | 0,41 | Cup a 1 | 15,05 |
| Italy - CAAM 2545 | M | 45 | Amb a 1 | 0 | Cry j 1 | 0,1 | Cup a 1 | 5,1 |
| Italy - CAAM 2546 | F | 45 | Amb a 1 | 0 | Cry j 1 | 0,9 | Cup a 1 | 39,57 |
| Italy - CAAM 2547 | F | 45 | Amb a 1 | 0 | Cry j 1 | 21,55 | Cup a 1 | 13,91 |
| Italy - CAAM 2548 | F | 44 | Amb a 1 | 0 | Cry j 1 | 0,75 | Cup a 1 | 0,95 |
| Italy - CAAM 2549 | F | 44 | Amb a 1 | 0 | Cry j 1 | 0,14 | Cup a 1 | 4,68 |
| Italy - CAAM 2550 | F | 44 | Amb a 1 | 0 | Cry j 1 | 3,39 | Cup a 1 | 4,7 |
| Italy - CAAM 2551 | F | 44 | Amb a 1 | 0 | Cry j 1 | 1,43 | Cup a 1 | 6,33 |
| Italy - CAAM 2552 | F | 44 | Amb a 1 | 0 | Cry j 1 | 1,17 | Cup a 1 | 4,3 |
| Italy - CAAM 2553 | M | 44 | Amb a 1 | 0 | Cry j 1 | 0,08 | Cup a 1 | 0,78 |
| Italy - CAAM 2554 | F | 44 | Amb a 1 | 0 | Cry j 1 | 0 | Cup a 1 | 0,16 |
| Italy - CAAM 2555 | F | 44 | Amb a 1 | 0 | Cry j 1 | 0,55 | Cup a 1 | 5,52 |
| Italy - CAAM 2556 | F | 44 | Amb a 1 | 0 | Cry j 1 | 0 | Cup a 1 | 4,33 |
| Italy - CAAM 2557 | F | 44 | Amb a 1 | 0 | Cry j 1 | 0 | Cup a 1 | 0,95 |
| Italy - CAAM 2558 | M | 44 | Amb a 1 | 0 | Cry j 1 | 0,28 | Cup a 1 | 12,06 |
| Italy - CAAM 2559 | M | 44 | Amb a 1 | 0 | Cry j 1 | 0 | Cup a 1 | 1,69 |
| Italy - CAAM 2560 | M | 44 | Amb a 1 | 0 | Cry j 1 | 0,28 | Cup a 1 | 6,22 |
| Italy - CAAM 2561 | M | 44 | Amb a 1 | 0 | Cry j 1 | 3,69 | Cup a 1 | 2,21 |
| Italy - CAAM 2562 | F | 44 | Amb a 1 | 0 | Cry j 1 | 1,09 | Cup a 1 | 0 |
| Italy - CAAM 2563 | M | 44 | Amb a 1 | 0 | Cry j 1 | 2,43 | Cup a 1 | 4,31 |
| Italy - CAAM 2564 | M | 44 | Amb a 1 | 0 | Cry j 1 | 4,9 | Cup a 1 | 13,31 |
| Italy - CAAM 2565 | M | 44 | Amb a 1 | 0 | Cry j 1 | 0,24 | Cup a 1 | 2,6 |
| Italy - CAAM 2566 | F | 44 | Amb a 1 | 0 | Cry j 1 | 2,2 | Cup a 1 | 2,3 |
| Italy - CAAM 2567 | M | 44 | Amb a 1 | 0 | Cry j 1 | 0 | Cup a 1 | 0,43 |
| Italy - CAAM 2568 | M | 44 | Amb a 1 | 0 | Cry j 1 | 1,59 | Cup a 1 | 3,42 |
| Italy - CAAM 2569 | F | 44 | Amb a 1 | 0 | Cry j 1 | 0 | Cup a 1 | 1,1 |
| Italy - CAAM 2570 | M | 44 | Amb a 1 | 0 | Cry j 1 | 0 | Cup a 1 | 1,32 |
| Italy - CAAM 2571 | F | 44 | Amb a 1 | 0 | Cry j 1 | 7,69 | Cup a 1 | 3,52 |
| Italy - CAAM 2572 | F | 44 | Amb a 1 | 0 | Cry j 1 | 3,84 | Cup a 1 | 4,78 |
| Italy - CAAM 2573 | M | 44 | Amb a 1 | 0 | Cry j 1 | 1,47 | Cup a 1 | 6,32 |
| Italy - CAAM 2574 | F | 44 | Amb a 1 | 0 | Cry j 1 | 0,06 | Cup a 1 | 0 |
| Italy - CAAM 2575 | M | 44 | Amb a 1 | 0 | Cry j 1 | 0 | Cup a 1 | 0,92 |
| Italy - CAAM 2576 | F | 44 | Amb a 1 | 0 | Cry j 1 | 0 | Cup a 1 | 0,44 |
| Italy - CAAM 2577 | F | 44 | Amb a 1 | 0 | Cry j 1 | 0 | Cup a 1 | 0,66 |
| Italy - CAAM 2578 | F | 44 | Amb a 1 | 0 | Cry j 1 | 2,54 | Cup a 1 | 2,07 |
| Italy - CAAM 2579 | F | 44 | Amb a 1 | 0 | Cry j 1 | 2,6 | Cup a 1 | 4,54 |
| Italy - CAAM 2580 | F | 44 | Amb a 1 | 0 | Cry j 1 | 0,64 | Cup a 1 | 0 |
| Italy - CAAM 2581 | M | 44 | Amb a 1 | 0 | Cry j 1 | 0,17 | Cup a 1 | 0,35 |
| Italy - CAAM 2582 | F | 44 | Amb a 1 | 0 | Cry j 1 | 0,79 | Cup a 1 | 1,46 |
| Italy - CAAM 2583 | M | 44 | Amb a 1 | 0 | Cry j 1 | 0,62 | Cup a 1 | 8,78 |
| Italy - CAAM 2584 | F | 44 | Amb a 1 | 0 | Cry j 1 | 0,6 | Cup a 1 | 0,61 |
| Italy - CAAM 2585 | F | 44 | Amb a 1 | 0 | Cry j 1 | 0 | Cup a 1 | 0,13 |
| Italy - CAAM 2586 | M | 44 | Amb a 1 | 0 | Cry j 1 | 0,5 | Cup a 1 | 1,45 |
| Italy - CAAM 2587 | M | 44 | Amb a 1 | 0 | Cry j 1 | 0,93 | Cup a 1 | 2,92 |
| Italy - CAAM 2588 | F | 44 | Amb a 1 | 0 | Cry j 1 | 6,14 | Cup a 1 | 1,83 |
| Italy - CAAM 2589 | F | 44 | Amb a 1 | 0 | Cry j 1 | 0,79 | Cup a 1 | 0,86 |
| Italy - CAAM 2590 | F | 44 | Amb a 1 | 0 | Cry j 1 | 0,12 | Cup a 1 | 0,5 |
| Italy - CAAM 2591 | F | 44 | Amb a 1 | 0 | Cry j 1 | 9,71 | Cup a 1 | 0 |
| Italy - CAAM 2592 | M | 44 | Amb a 1 | 0 | Cry j 1 | 0,17 | Cup a 1 | 0,33 |
| Italy - CAAM 2593 | F | 44 | Amb a 1 | 0 | Cry j 1 | 9,15 | Cup a 1 | 2,5 |
| Italy - CAAM 2594 | F | 44 | Amb a 1 | 0 | Cry j 1 | 5,03 | Cup a 1 | 5,19 |
| Italy - CAAM 2595 | F | 44 | Amb a 1 | 0 | Cry j 1 | 0,57 | Cup a 1 | 0,56 |
| Italy - CAAM 2596 | M | 44 | Amb a 1 | 0 | Cry j 1 | 0,8 | Cup a 1 | 1,28 |
| Italy - CAAM 2597 | F | 44 | Amb a 1 | 0 | Cry j 1 | 0 | Cup a 1 | 0,34 |
| Italy - CAAM 2598 | F | 44 | Amb a 1 | 0 | Cry j 1 | 1,67 | Cup a 1 | 0,8 |
| Italy - CAAM 2599 | M | 44 | Amb a 1 | 0 | Cry j 1 | 1,02 | Cup a 1 | 7,63 |
| Italy - CAAM 2600 | M | 44 | Amb a 1 | 0 | Cry j 1 | 5,8 | Cup a 1 | 4,51 |
| Italy - CAAM 2601 | F | 44 | Amb a 1 | 0 | Cry j 1 | 3,59 | Cup a 1 | 3,6 |
| Italy - CAAM 2602 | M | 44 | Amb a 1 | 0 | Cry j 1 | 1,79 | Cup a 1 | 0,93 |
| Italy - CAAM 2603 | F | 44 | Amb a 1 | 0 | Cry j 1 | 0 | Cup a 1 | 0,47 |
| Italy - CAAM 2604 | M | 44 | Amb a 1 | 0 | Cry j 1 | 1,74 | Cup a 1 | 2,71 |
| Italy - CAAM 2605 | M | 44 | Amb a 1 | 0 | Cry j 1 | 0 | Cup a 1 | 0,58 |
| Italy - CAAM 2606 | F | 44 | Amb a 1 | 0 | Cry j 1 | 1,28 | Cup a 1 | 12,29 |
| Italy - CAAM 2607 | M | 44 | Amb a 1 | 0 | Cry j 1 | 1,55 | Cup a 1 | 17,13 |
| Italy - CAAM 2608 | F | 44 | Amb a 1 | 0 | Cry j 1 | 1,02 | Cup a 1 | 4,74 |
| Italy - CAAM 2609 | F | 44 | Amb a 1 | 0 | Cry j 1 | 0,21 | Cup a 1 | 0,12 |
| Italy - CAAM 2610 | M | 44 | Amb a 1 | 0 | Cry j 1 | 0,08 | Cup a 1 | 0,06 |
| Italy - CAAM 2611 | F | 44 | Amb a 1 | 0 | Cry j 1 | 0,04 | Cup a 1 | 1,13 |
| Italy - CAAM 2612 | F | 44 | Amb a 1 | 0 | Cry j 1 | 0,41 | Cup a 1 | 1,17 |
| Italy - CAAM 2613 | F | 44 | Amb a 1 | 0 | Cry j 1 | 0,13 | Cup a 1 | 0,3 |
| Italy - CAAM 2614 | F | 44 | Amb a 1 | 0 | Cry j 1 | 0 | Cup a 1 | 0,67 |
| Italy - CAAM 2615 | M | 44 | Amb a 1 | 0 | Cry j 1 | 0,3 | Cup a 1 | 3,95 |
| Italy - CAAM 2616 | F | 44 | Amb a 1 | 0 | Cry j 1 | 1,56 | Cup a 1 | 5,97 |
| Italy - CAAM 2617 | F | 44 | Amb a 1 | 0 | Cry j 1 | 0,11 | Cup a 1 | 1,5 |
| Italy - CAAM 2618 | M | 44 | Amb a 1 | 0 | Cry j 1 | 0 | Cup a 1 | 0,09 |
| Italy - CAAM 2619 | M | 44 | Amb a 1 | 0 | Cry j 1 | 0 | Cup a 1 | 0,49 |
| Italy - CAAM 2620 | M | 44 | Amb a 1 | 0 | Cry j 1 | 2,75 | Cup a 1 | 3,33 |
| Italy - CAAM 2621 | F | 44 | Amb a 1 | 0 | Cry j 1 | 0,02 | Cup a 1 | 2 |
| Italy - CAAM 2622 | M | 44 | Amb a 1 | 0 | Cry j 1 | 0,08 | Cup a 1 | 0,21 |
| Italy - CAAM 2623 | F | 44 | Amb a 1 | 0 | Cry j 1 | 1,57 | Cup a 1 | 6,68 |
| Italy - CAAM 2624 | M | 44 | Amb a 1 | 0 | Cry j 1 | 1,04 | Cup a 1 | 9,75 |
| Italy - CAAM 2625 | F | 44 | Amb a 1 | 0 | Cry j 1 | 0,85 | Cup a 1 | 1,96 |
| Italy - CAAM 2626 | F | 44 | Amb a 1 | 0 | Cry j 1 | 0,18 | Cup a 1 | 5,43 |
| Italy - CAAM 2627 | F | 44 | Amb a 1 | 0 | Cry j 1 | 0,13 | Cup a 1 | 1,7 |
| Italy - CAAM 2628 | M | 44 | Amb a 1 | 0 | Cry j 1 | 0 | Cup a 1 | 1,48 |
| Italy - CAAM 2629 | F | 44 | Amb a 1 | 0 | Cry j 1 | 1,07 | Cup a 1 | 3,39 |
| Italy - CAAM 2630 | F | 44 | Amb a 1 | 0 | Cry j 1 | 4,9 | Cup a 1 | 5,76 |
| Italy - CAAM 2631 | F | 44 | Amb a 1 | 0 | Cry j 1 | 0,21 | Cup a 1 | 0,66 |
| Italy - CAAM 2632 | F | 44 | Amb a 1 | 0 | Cry j 1 | 2,86 | Cup a 1 | 3,62 |
| Italy - CAAM 2633 | F | 44 | Amb a 1 | 0 | Cry j 1 | 1,91 | Cup a 1 | 11,86 |
| Italy - CAAM 2634 | F | 44 | Amb a 1 | 0 | Cry j 1 | 0,56 | Cup a 1 | 2,2 |
| Italy - CAAM 2635 | M | 44 | Amb a 1 | 0 | Cry j 1 | 1,67 | Cup a 1 | 2,55 |
| Italy - CAAM 2636 | M | 44 | Amb a 1 | 0 | Cry j 1 | 3,07 | Cup a 1 | 14,86 |
| Italy - CAAM 2637 | M | 44 | Amb a 1 | 0 | Cry j 1 | 0,35 | Cup a 1 | 3,58 |
| Italy - CAAM 2638 | F | 44 | Amb a 1 | 0 | Cry j 1 | 10,69 | Cup a 1 | 10,9 |
| Italy - CAAM 2639 | M | 44 | Amb a 1 | 0 | Cry j 1 | 9,39 | Cup a 1 | 19,29 |
| Italy - CAAM 2640 | F | 44 | Amb a 1 | 0 | Cry j 1 | 0,22 | Cup a 1 | 6,47 |
| Italy - CAAM 2641 | M | 44 | Amb a 1 | 0 | Cry j 1 | 0,03 | Cup a 1 | 1,26 |
| Italy - CAAM 2642 | M | 44 | Amb a 1 | 0 | Cry j 1 | 1,74 | Cup a 1 | 2,51 |
| Italy - CAAM 2643 | F | 44 | Amb a 1 | 0 | Cry j 1 | 0 | Cup a 1 | 3,4 |
| Italy - CAAM 2644 | F | 44 | Amb a 1 | 0 | Cry j 1 | 0,83 | Cup a 1 | 3,53 |
| Italy - CAAM 2645 | F | 44 | Amb a 1 | 0 | Cry j 1 | 4,04 | Cup a 1 | 5,14 |
| Italy - CAAM 2646 | M | 44 | Amb a 1 | 0 | Cry j 1 | 0,62 | Cup a 1 | 2,44 |
| Italy - CAAM 2647 | M | 44 | Amb a 1 | 0 | Cry j 1 | 5,92 | Cup a 1 | 27,45 |
| Italy - CAAM 2648 | M | 44 | Amb a 1 | 0 | Cry j 1 | 0 | Cup a 1 | 2,66 |
| Italy - CAAM 2649 | M | 44 | Amb a 1 | 0 | Cry j 1 | 5,6 | Cup a 1 | 9,34 |
| Italy - CAAM 2650 | M | 44 | Amb a 1 | 0 | Cry j 1 | 0 | Cup a 1 | 3,16 |
| Italy - CAAM 2651 | M | 44 | Amb a 1 | 0 | Cry j 1 | 2,07 | Cup a 1 | 18,35 |
| Italy - CAAM 2652 | F | 44 | Amb a 1 | 0 | Cry j 1 | 6,64 | Cup a 1 | 18,3 |
| Italy - CAAM 2653 | M | 44 | Amb a 1 | 0 | Cry j 1 | 0 | Cup a 1 | 0,17 |
| Italy - CAAM 2654 | F | 44 | Amb a 1 | 0 | Cry j 1 | 0 | Cup a 1 | 1,67 |
| Italy - CAAM 2655 | F | 44 | Amb a 1 | 0 | Cry j 1 | 0 | Cup a 1 | 1,56 |
| Italy - CAAM 2656 | M | 44 | Amb a 1 | 0 | Cry j 1 | 0,52 | Cup a 1 | 22,43 |
| Italy - CAAM 2657 | M | 44 | Amb a 1 | 0 | Cry j 1 | 0,59 | Cup a 1 | 4,31 |
| Italy - CAAM 2658 | F | 44 | Amb a 1 | 0 | Cry j 1 | 0,02 | Cup a 1 | 0,04 |
| Italy - CAAM 2659 | M | 44 | Amb a 1 | 0 | Cry j 1 | 0,06 | Cup a 1 | 6,42 |
| Italy - CAAM 2660 | F | 44 | Amb a 1 | 0 | Cry j 1 | 0 | Cup a 1 | 8,95 |
| Italy - CAAM 2661 | M | 44 | Amb a 1 | 0 | Cry j 1 | 3,05 | Cup a 1 | 3,63 |
| Italy - CAAM 2662 | M | 44 | Amb a 1 | 0 | Cry j 1 | 0,36 | Cup a 1 | 1,22 |
| Italy - CAAM 2663 | M | 44 | Amb a 1 | 0 | Cry j 1 | 0 | Cup a 1 | 0,53 |
| Italy - CAAM 2664 | M | 44 | Amb a 1 | 0 | Cry j 1 | 0,62 | Cup a 1 | 0,76 |
| Italy - CAAM 2665 | F | 44 | Amb a 1 | 0 | Cry j 1 | 1,99 | Cup a 1 | 2,15 |
| Italy - CAAM 2666 | F | 44 | Amb a 1 | 0 | Cry j 1 | 0 | Cup a 1 | 14,5 |
| Italy - CAAM 2667 | M | 44 | Amb a 1 | 0 | Cry j 1 | 15,01 | Cup a 1 | 37,71 |
| Italy - CAAM 2668 | F | 44 | Amb a 1 | 0 | Cry j 1 | 0,6 | Cup a 1 | 1,57 |
| Italy - CAAM 2669 | F | 44 | Amb a 1 | 0 | Cry j 1 | 0,03 | Cup a 1 | 0,04 |
| Italy - CAAM 2670 | M | 44 | Amb a 1 | 0 | Cry j 1 | 6,17 | Cup a 1 | 27,67 |
| Italy - CAAM 2671 | M | 44 | Amb a 1 | 0 | Cry j 1 | 0,11 | Cup a 1 | 0,9 |
| Italy - CAAM 2672 | M | 44 | Amb a 1 | 0 | Cry j 1 | 0,07 | Cup a 1 | 0,05 |
| Italy - CAAM 2673 | F | 44 | Amb a 1 | 0 | Cry j 1 | 0 | Cup a 1 | 0,52 |
| Italy - CAAM 2674 | F | 44 | Amb a 1 | 0 | Cry j 1 | 8,3 | Cup a 1 | 27,82 |
| Italy - CAAM 2675 | M | 44 | Amb a 1 | 0 | Cry j 1 | 0 | Cup a 1 | 0,81 |
| Italy - CAAM 2676 | F | 44 | Amb a 1 | 0 | Cry j 1 | 0,05 | Cup a 1 | 1,03 |
| Italy - CAAM 2677 | F | 44 | Amb a 1 | 0 | Cry j 1 | 2,41 | Cup a 1 | 4,04 |
| Italy - CAAM 2678 | F | 44 | Amb a 1 | 0 | Cry j 1 | 0 | Cup a 1 | 0,39 |
| Italy - CAAM 2679 | F | 44 | Amb a 1 | 0 | Cry j 1 | 0,55 | Cup a 1 | 0,67 |
| Italy - CAAM 2680 | M | 44 | Amb a 1 | 0 | Cry j 1 | 0,2 | Cup a 1 | 8,99 |
| Italy - CAAM 2681 | F | 44 | Amb a 1 | 0 | Cry j 1 | 0 | Cup a 1 | 0,15 |
| Italy - CAAM 2682 | F | 44 | Amb a 1 | 0 | Cry j 1 | 20,08 | Cup a 1 | 43,31 |
| Italy - CAAM 2683 | M | 44 | Amb a 1 | 0 | Cry j 1 | 0,36 | Cup a 1 | 10,76 |
| Italy - CAAM 2684 | M | 44 | Amb a 1 | 0 | Cry j 1 | 0,25 | Cup a 1 | 17,12 |
| Italy - CAAM 2685 | M | 44 | Amb a 1 | 0 | Cry j 1 | 3,04 | Cup a 1 | 6,19 |
| Italy - CAAM 2686 | F | 44 | Amb a 1 | 0 | Cry j 1 | 0 | Cup a 1 | 0,17 |
| Italy - CAAM 2687 | M | 44 | Amb a 1 | 0 | Cry j 1 | 0,2 | Cup a 1 | 3,56 |
| Italy - CAAM 2688 | F | 44 | Amb a 1 | 0 | Cry j 1 | 4,14 | Cup a 1 | 8,54 |
| Italy - CAAM 2689 | M | 44 | Amb a 1 | 0 | Cry j 1 | 0,19 | Cup a 1 | 0,98 |
| Italy - CAAM 2690 | F | 44 | Amb a 1 | 0 | Cry j 1 | 0,95 | Cup a 1 | 19,85 |
| Italy - CAAM 2691 | M | 44 | Amb a 1 | 0 | Cry j 1 | 0 | Cup a 1 | 0,66 |
| Italy - CAAM 2692 | M | 44 | Amb a 1 | 0 | Cry j 1 | 0,14 | Cup a 1 | 2,79 |
| Italy - CAAM 2693 | M | 44 | Amb a 1 | 0 | Cry j 1 | 0,35 | Cup a 1 | 4,12 |
| Italy - CAAM 2694 | F | 44 | Amb a 1 | 0 | Cry j 1 | 5,23 | Cup a 1 | 17,53 |
| Italy - CAAM 2695 | F | 44 | Amb a 1 | 0 | Cry j 1 | 2,93 | Cup a 1 | 14,7 |
| Italy - CAAM 2696 | F | 44 | Amb a 1 | 0 | Cry j 1 | 0,26 | Cup a 1 | 1,52 |
| Italy - CAAM 2697 | M | 44 | Amb a 1 | 0 | Cry j 1 | 0,97 | Cup a 1 | 13,83 |
| Italy - CAAM 2698 | F | 44 | Amb a 1 | 0 | Cry j 1 | 0,31 | Cup a 1 | 12,03 |
| Italy - CAAM 2699 | M | 44 | Amb a 1 | 0 | Cry j 1 | 0,59 | Cup a 1 | 7,04 |
| Italy - CAAM 2700 | M | 44 | Amb a 1 | 0 | Cry j 1 | 0,21 | Cup a 1 | 0,82 |
| Italy - CAAM 2701 | F | 44 | Amb a 1 | 0 | Cry j 1 | 0,26 | Cup a 1 | 10,86 |
| Italy - CAAM 2702 | F | 44 | Amb a 1 | 0 | Cry j 1 | 1,83 | Cup a 1 | 10,24 |
| Italy - CAAM 2703 | M | 44 | Amb a 1 | 0 | Cry j 1 | 0,98 | Cup a 1 | 7,44 |
| Italy - CAAM 2704 | M | 44 | Amb a 1 | 0 | Cry j 1 | 7,66 | Cup a 1 | 26,02 |
| Italy - CAAM 2705 | F | 44 | Amb a 1 | 0 | Cry j 1 | 0,25 | Cup a 1 | 0,29 |
| Italy - CAAM 2706 | F | 44 | Amb a 1 | 0 | Cry j 1 | 1,22 | Cup a 1 | 7,72 |
| Italy - CAAM 2707 | M | 44 | Amb a 1 | 0 | Cry j 1 | 0 | Cup a 1 | 0,48 |
| Italy - CAAM 2708 | M | 44 | Amb a 1 | 0 | Cry j 1 | 0 | Cup a 1 | 0,47 |
| Italy - CAAM 2709 | M | 44 | Amb a 1 | 0 | Cry j 1 | 0 | Cup a 1 | 0,43 |
| Italy - CAAM 2710 | M | 44 | Amb a 1 | 0 | Cry j 1 | 0,16 | Cup a 1 | 2,14 |
| Italy - CAAM 2711 | M | 44 | Amb a 1 | 0 | Cry j 1 | 1,04 | Cup a 1 | 8,53 |
| Italy - CAAM 2712 | F | 44 | Amb a 1 | 0 | Cry j 1 | 4,24 | Cup a 1 | 8,05 |
| Italy - CAAM 2713 | F | 44 | Amb a 1 | 0 | Cry j 1 | 0,29 | Cup a 1 | 2,59 |
| Italy - CAAM 2714 | M | 44 | Amb a 1 | 0 | Cry j 1 | 0 | Cup a 1 | 8,19 |
| Italy - CAAM 2715 | F | 44 | Amb a 1 | 0 | Cry j 1 | 0 | Cup a 1 | 0,11 |
| Italy - CAAM 2716 | F | 44 | Amb a 1 | 0 | Cry j 1 | 0,59 | Cup a 1 | 8,89 |
| Italy - CAAM 2717 | M | 44 | Amb a 1 | 0 | Cry j 1 | 0,4 | Cup a 1 | 5,51 |
| Italy - CAAM 2718 | F | 44 | Amb a 1 | 0 | Cry j 1 | 0,12 | Cup a 1 | 36,64 |
| Italy - CAAM 2719 | M | 44 | Amb a 1 | 0 | Cry j 1 | 0 | Cup a 1 | 1,12 |
| Italy - CAAM 2720 | F | 44 | Amb a 1 | 0 | Cry j 1 | 0 | Cup a 1 | 9,2 |
| Italy - CAAM 2721 | F | 44 | Amb a 1 | 0 | Cry j 1 | 1,22 | Cup a 1 | 10,85 |
| Italy - CAAM 2722 | M | 44 | Amb a 1 | 0 | Cry j 1 | 0 | Cup a 1 | 2,55 |
| Italy - CAAM 2723 | F | 44 | Amb a 1 | 0 | Cry j 1 | 0 | Cup a 1 | 0,71 |
| Italy - CAAM 2724 | M | 44 | Amb a 1 | 0 | Cry j 1 | 0,54 | Cup a 1 | 9,77 |
| Italy - CAAM 2725 | F | 44 | Amb a 1 | 0 | Cry j 1 | 0 | Cup a 1 | 0,28 |
| Italy - CAAM 2726 | F | 44 | Amb a 1 | 0 | Cry j 1 | 0 | Cup a 1 | 2,74 |
| Italy - CAAM 2727 | F | 44 | Amb a 1 | 0 | Cry j 1 | 0 | Cup a 1 | 1,8 |
| Italy - CAAM 2728 | F | 44 | Amb a 1 | 0 | Cry j 1 | 4,37 | Cup a 1 | 5,06 |
| Italy - CAAM 2729 | F | 44 | Amb a 1 | 0 | Cry j 1 | 0 | Cup a 1 | 5,2 |
| Italy - CAAM 2730 | M | 44 | Amb a 1 | 0 | Cry j 1 | 2 | Cup a 1 | 57 |
| Italy - CAAM 2731 | F | 44 | Amb a 1 | 0 | Cry j 1 | 0 | Cup a 1 | 21 |
| Italy - CAAM 2732 | F | 44 | Amb a 1 | 0 | Cry j 1 | 0,1 | Cup a 1 | 0,22 |
| Italy - CAAM 2733 | M | 43 | Amb a 1 | 0 | Cry j 1 | 10,76 | Cup a 1 | 18,21 |
| Italy - CAAM 2734 | F | 43 | Amb a 1 | 0 | Cry j 1 | 0 | Cup a 1 | 3,24 |
| Italy - CAAM 2735 | F | 43 | Amb a 1 | 0 | Cry j 1 | 0,53 | Cup a 1 | 4,58 |
| Italy - CAAM 2736 | M | 43 | Amb a 1 | 0 | Cry j 1 | 0 | Cup a 1 | 2,73 |
| Italy - CAAM 2737 | F | 43 | Amb a 1 | 0 | Cry j 1 | 11 | Cup a 1 | 14,58 |
| Italy - CAAM 2738 | M | 43 | Amb a 1 | 0 | Cry j 1 | 0 | Cup a 1 | 2,48 |
| Italy - CAAM 2739 | F | 43 | Amb a 1 | 0 | Cry j 1 | 0,57 | Cup a 1 | 5,62 |
| Italy - CAAM 2740 | M | 43 | Amb a 1 | 0 | Cry j 1 | 1,09 | Cup a 1 | 1,16 |
| Italy - CAAM 2741 | M | 43 | Amb a 1 | 0 | Cry j 1 | 8,9 | Cup a 1 | 12,93 |
| Italy - CAAM 2742 | M | 43 | Amb a 1 | 0 | Cry j 1 | 5,88 | Cup a 1 | 3,22 |
| Italy - CAAM 2743 | F | 43 | Amb a 1 | 0 | Cry j 1 | 0 | Cup a 1 | 4,84 |
| Italy - CAAM 2744 | F | 43 | Amb a 1 | 0 | Cry j 1 | 0 | Cup a 1 | 4,77 |
| Italy - CAAM 2745 | F | 43 | Amb a 1 | 0 | Cry j 1 | 0,14 | Cup a 1 | 0,23 |
| Italy - CAAM 2746 | M | 43 | Amb a 1 | 0 | Cry j 1 | 0 | Cup a 1 | 0,64 |
| Italy - CAAM 2747 | M | 43 | Amb a 1 | 0 | Cry j 1 | 1,89 | Cup a 1 | 2,98 |
| Italy - CAAM 2748 | M | 43 | Amb a 1 | 0 | Cry j 1 | 0 | Cup a 1 | 1,38 |
| Italy - CAAM 2749 | F | 43 | Amb a 1 | 0 | Cry j 1 | 3,52 | Cup a 1 | 5,85 |
| Italy - CAAM 2750 | M | 43 | Amb a 1 | 0 | Cry j 1 | 0,9 | Cup a 1 | 4,34 |
| Italy - CAAM 2751 | F | 43 | Amb a 1 | 0 | Cry j 1 | 0 | Cup a 1 | 2,45 |
| Italy - CAAM 2752 | F | 43 | Amb a 1 | 0 | Cry j 1 | 0,49 | Cup a 1 | 0,2 |
| Italy - CAAM 2753 | F | 43 | Amb a 1 | 0 | Cry j 1 | 1,71 | Cup a 1 | 2,06 |
| Italy - CAAM 2754 | F | 43 | Amb a 1 | 0 | Cry j 1 | 0 | Cup a 1 | 1,58 |
| Italy - CAAM 2755 | F | 43 | Amb a 1 | 0 | Cry j 1 | 0 | Cup a 1 | 3,44 |
| Italy - CAAM 2756 | F | 43 | Amb a 1 | 0 | Cry j 1 | 3,74 | Cup a 1 | 9,21 |
| Italy - CAAM 2757 | M | 43 | Amb a 1 | 0 | Cry j 1 | 0,3 | Cup a 1 | 0,3 |
| Italy - CAAM 2758 | F | 43 | Amb a 1 | 0 | Cry j 1 | 1,76 | Cup a 1 | 3,42 |
| Italy - CAAM 2759 | F | 43 | Amb a 1 | 0 | Cry j 1 | 0 | Cup a 1 | 1,48 |
| Italy - CAAM 2760 | M | 43 | Amb a 1 | 0 | Cry j 1 | 7,31 | Cup a 1 | 12,56 |
| Italy - CAAM 2761 | F | 43 | Amb a 1 | 0 | Cry j 1 | 0,79 | Cup a 1 | 3,54 |
| Italy - CAAM 2762 | F | 43 | Amb a 1 | 0 | Cry j 1 | 0,36 | Cup a 1 | 1,71 |
| Italy - CAAM 2763 | F | 43 | Amb a 1 | 0 | Cry j 1 | 0,13 | Cup a 1 | 0 |
| Italy - CAAM 2764 | M | 43 | Amb a 1 | 0 | Cry j 1 | 2,33 | Cup a 1 | 9,89 |
| Italy - CAAM 2765 | F | 43 | Amb a 1 | 0 | Cry j 1 | 0 | Cup a 1 | 0,44 |
| Italy - CAAM 2766 | F | 43 | Amb a 1 | 0 | Cry j 1 | 1,09 | Cup a 1 | 8,98 |
| Italy - CAAM 2767 | F | 43 | Amb a 1 | 0 | Cry j 1 | 1,08 | Cup a 1 | 3,71 |
| Italy - CAAM 2768 | F | 43 | Amb a 1 | 0 | Cry j 1 | 0,85 | Cup a 1 | 3,92 |
| Italy - CAAM 2769 | F | 43 | Amb a 1 | 0 | Cry j 1 | 0,03 | Cup a 1 | 1,01 |
| Italy - CAAM 2770 | F | 43 | Amb a 1 | 0 | Cry j 1 | 0 | Cup a 1 | 3,12 |
| Italy - CAAM 2771 | F | 43 | Amb a 1 | 0 | Cry j 1 | 1,38 | Cup a 1 | 1,49 |
| Italy - CAAM 2772 | M | 43 | Amb a 1 | 0 | Cry j 1 | 0,63 | Cup a 1 | 1,34 |
| Italy - CAAM 2773 | F | 43 | Amb a 1 | 0 | Cry j 1 | 3,1 | Cup a 1 | 2,67 |
| Italy - CAAM 2774 | F | 43 | Amb a 1 | 0 | Cry j 1 | 0 | Cup a 1 | 0,08 |
| Italy - CAAM 2775 | M | 43 | Amb a 1 | 0 | Cry j 1 | 3,42 | Cup a 1 | 1,94 |
| Italy - CAAM 2776 | F | 43 | Amb a 1 | 0 | Cry j 1 | 0,09 | Cup a 1 | 0,24 |
| Italy - CAAM 2777 | M | 43 | Amb a 1 | 0 | Cry j 1 | 0,79 | Cup a 1 | 0,85 |
| Italy - CAAM 2778 | F | 43 | Amb a 1 | 0 | Cry j 1 | 9,97 | Cup a 1 | 8,44 |
| Italy - CAAM 2779 | M | 43 | Amb a 1 | 0 | Cry j 1 | 1,59 | Cup a 1 | 3,6 |
| Italy - CAAM 2780 | F | 43 | Amb a 1 | 0 | Cry j 1 | 0,97 | Cup a 1 | 0,86 |
| Italy - CAAM 2781 | F | 43 | Amb a 1 | 0 | Cry j 1 | 0,15 | Cup a 1 | 0 |
| Italy - CAAM 2782 | F | 43 | Amb a 1 | 0 | Cry j 1 | 0,46 | Cup a 1 | 0,61 |
| Italy - CAAM 2783 | F | 43 | Amb a 1 | 0 | Cry j 1 | 0 | Cup a 1 | 0,4 |
| Italy - CAAM 2784 | F | 43 | Amb a 1 | 0 | Cry j 1 | 0,81 | Cup a 1 | 1,15 |
| Italy - CAAM 2785 | F | 43 | Amb a 1 | 0 | Cry j 1 | 0,38 | Cup a 1 | 0,94 |
| Italy - CAAM 2786 | M | 43 | Amb a 1 | 0 | Cry j 1 | 0,16 | Cup a 1 | 0,45 |
| Italy - CAAM 2787 | F | 43 | Amb a 1 | 0 | Cry j 1 | 0,34 | Cup a 1 | 0,31 |
| Italy - CAAM 2788 | F | 43 | Amb a 1 | 0 | Cry j 1 | 0 | Cup a 1 | 0,15 |
| Italy - CAAM 2789 | F | 43 | Amb a 1 | 0 | Cry j 1 | 19,84 | Cup a 1 | 41,84 |
| Italy - CAAM 2790 | F | 43 | Amb a 1 | 0 | Cry j 1 | 0,65 | Cup a 1 | 2,17 |
| Italy - CAAM 2791 | F | 43 | Amb a 1 | 0 | Cry j 1 | 0,83 | Cup a 1 | 1,43 |
| Italy - CAAM 2792 | F | 43 | Amb a 1 | 0 | Cry j 1 | 1,58 | Cup a 1 | 2,71 |
| Italy - CAAM 2793 | F | 43 | Amb a 1 | 0 | Cry j 1 | 0 | Cup a 1 | 15,16 |
| Italy - CAAM 2794 | M | 43 | Amb a 1 | 0 | Cry j 1 | 0,35 | Cup a 1 | 0 |
| Italy - CAAM 2795 | M | 43 | Amb a 1 | 0 | Cry j 1 | 0 | Cup a 1 | 0,11 |
| Italy - CAAM 2796 | F | 43 | Amb a 1 | 0 | Cry j 1 | 6,56 | Cup a 1 | 24,75 |
| Italy - CAAM 2797 | F | 43 | Amb a 1 | 0 | Cry j 1 | 0 | Cup a 1 | 3,3 |
| Italy - CAAM 2798 | M | 43 | Amb a 1 | 0 | Cry j 1 | 0,99 | Cup a 1 | 5,39 |
| Italy - CAAM 2799 | M | 43 | Amb a 1 | 0 | Cry j 1 | 0,58 | Cup a 1 | 4,2 |
| Italy - CAAM 2800 | M | 43 | Amb a 1 | 0 | Cry j 1 | 0,99 | Cup a 1 | 3,38 |
| Italy - CAAM 2801 | M | 43 | Amb a 1 | 0 | Cry j 1 | 0,2 | Cup a 1 | 1,58 |
| Italy - CAAM 2802 | F | 43 | Amb a 1 | 0 | Cry j 1 | 0,28 | Cup a 1 | 5,47 |
| Italy - CAAM 2803 | M | 43 | Amb a 1 | 0 | Cry j 1 | 4,2 | Cup a 1 | 8,68 |
| Italy - CAAM 2804 | F | 43 | Amb a 1 | 0 | Cry j 1 | 0,41 | Cup a 1 | 1,77 |
| Italy - CAAM 2805 | M | 43 | Amb a 1 | 0 | Cry j 1 | 0,21 | Cup a 1 | 2,95 |
| Italy - CAAM 2806 | M | 43 | Amb a 1 | 0 | Cry j 1 | 2,16 | Cup a 1 | 11,94 |
| Italy - CAAM 2807 | F | 43 | Amb a 1 | 0 | Cry j 1 | 0,77 | Cup a 1 | 1,53 |
| Italy - CAAM 2808 | M | 43 | Amb a 1 | 0 | Cry j 1 | 3,17 | Cup a 1 | 3,54 |
| Italy - CAAM 2809 | F | 43 | Amb a 1 | 0 | Cry j 1 | 0,24 | Cup a 1 | 2,58 |
| Italy - CAAM 2810 | M | 43 | Amb a 1 | 0 | Cry j 1 | 0,03 | Cup a 1 | 1,69 |
| Italy - CAAM 2811 | M | 43 | Amb a 1 | 0 | Cry j 1 | 0,61 | Cup a 1 | 0,71 |
| Italy - CAAM 2812 | F | 43 | Amb a 1 | 0 | Cry j 1 | 0 | Cup a 1 | 4,82 |
| Italy - CAAM 2813 | M | 43 | Amb a 1 | 0 | Cry j 1 | 0 | Cup a 1 | 0,08 |
| Italy - CAAM 2814 | F | 43 | Amb a 1 | 0 | Cry j 1 | 0 | Cup a 1 | 0,17 |
| Italy - CAAM 2815 | F | 43 | Amb a 1 | 0 | Cry j 1 | 0,89 | Cup a 1 | 3,76 |
| Italy - CAAM 2816 | M | 43 | Amb a 1 | 0 | Cry j 1 | 0 | Cup a 1 | 3,29 |
| Italy - CAAM 2817 | F | 43 | Amb a 1 | 0 | Cry j 1 | 0 | Cup a 1 | 6,35 |
| Italy - CAAM 2818 | F | 43 | Amb a 1 | 0 | Cry j 1 | 0,19 | Cup a 1 | 0,71 |
| Italy - CAAM 2819 | F | 43 | Amb a 1 | 0 | Cry j 1 | 0,37 | Cup a 1 | 1,27 |
| Italy - CAAM 2820 | F | 43 | Amb a 1 | 0 | Cry j 1 | 0,14 | Cup a 1 | 1,02 |
| Italy - CAAM 2821 | M | 43 | Amb a 1 | 0 | Cry j 1 | 2,21 | Cup a 1 | 17,93 |
| Italy - CAAM 2822 | F | 43 | Amb a 1 | 0 | Cry j 1 | 0,85 | Cup a 1 | 1,38 |
| Italy - CAAM 2823 | F | 43 | Amb a 1 | 0 | Cry j 1 | 5,42 | Cup a 1 | 8,11 |
| Italy - CAAM 2824 | F | 43 | Amb a 1 | 0 | Cry j 1 | 0,03 | Cup a 1 | 2,27 |
| Italy - CAAM 2825 | M | 43 | Amb a 1 | 0 | Cry j 1 | 0 | Cup a 1 | 0,31 |
| Italy - CAAM 2826 | M | 43 | Amb a 1 | 0 | Cry j 1 | 0,66 | Cup a 1 | 2,15 |
| Italy - CAAM 2827 | M | 43 | Amb a 1 | 0 | Cry j 1 | 0 | Cup a 1 | 0,54 |
| Italy - CAAM 2828 | F | 43 | Amb a 1 | 0 | Cry j 1 | 18,24 | Cup a 1 | 18,89 |
| Italy - CAAM 2829 | F | 43 | Amb a 1 | 0 | Cry j 1 | 0 | Cup a 1 | 6,16 |
| Italy - CAAM 2830 | M | 43 | Amb a 1 | 0 | Cry j 1 | 4,02 | Cup a 1 | 36,13 |
| Italy - CAAM 2831 | F | 43 | Amb a 1 | 0 | Cry j 1 | 0 | Cup a 1 | 1,65 |
| Italy - CAAM 2832 | F | 43 | Amb a 1 | 0 | Cry j 1 | 0 | Cup a 1 | 0,53 |
| Italy - CAAM 2833 | F | 43 | Amb a 1 | 0 | Cry j 1 | 2,6 | Cup a 1 | 14,5 |
| Italy - CAAM 2834 | F | 43 | Amb a 1 | 0 | Cry j 1 | 2,93 | Cup a 1 | 8,85 |
| Italy - CAAM 2835 | F | 43 | Amb a 1 | 0 | Cry j 1 | 0,13 | Cup a 1 | 4,42 |
| Italy - CAAM 2836 | F | 43 | Amb a 1 | 0 | Cry j 1 | 2,58 | Cup a 1 | 19,04 |
| Italy - CAAM 2837 | F | 43 | Amb a 1 | 0 | Cry j 1 | 1,15 | Cup a 1 | 6,12 |
| Italy - CAAM 2838 | M | 43 | Amb a 1 | 0 | Cry j 1 | 2,47 | Cup a 1 | 7,37 |
| Italy - CAAM 2839 | M | 43 | Amb a 1 | 0 | Cry j 1 | 14,49 | Cup a 1 | 9,97 |
| Italy - CAAM 2840 | F | 43 | Amb a 1 | 0 | Cry j 1 | 0 | Cup a 1 | 0,09 |
| Italy - CAAM 2841 | F | 43 | Amb a 1 | 0 | Cry j 1 | 0 | Cup a 1 | 0,25 |
| Italy - CAAM 2842 | F | 43 | Amb a 1 | 0 | Cry j 1 | 0,32 | Cup a 1 | 1,33 |
| Italy - CAAM 2843 | F | 43 | Amb a 1 | 0 | Cry j 1 | 0 | Cup a 1 | 2,13 |
| Italy - CAAM 2844 | M | 43 | Amb a 1 | 0 | Cry j 1 | 0,11 | Cup a 1 | 0,38 |
| Italy - CAAM 2845 | F | 43 | Amb a 1 | 0 | Cry j 1 | 0 | Cup a 1 | 0,85 |
| Italy - CAAM 2846 | M | 43 | Amb a 1 | 0 | Cry j 1 | 0,99 | Cup a 1 | 3,08 |
| Italy - CAAM 2847 | M | 43 | Amb a 1 | 0 | Cry j 1 | 0 | Cup a 1 | 5,48 |
| Italy - CAAM 2848 | F | 43 | Amb a 1 | 0 | Cry j 1 | 0 | Cup a 1 | 1,12 |
| Italy - CAAM 2849 | M | 43 | Amb a 1 | 0 | Cry j 1 | 0,11 | Cup a 1 | 1,4 |
| Italy - CAAM 2850 | F | 43 | Amb a 1 | 0 | Cry j 1 | 0,06 | Cup a 1 | 0,05 |
| Italy - CAAM 2851 | F | 43 | Amb a 1 | 0 | Cry j 1 | 0,3 | Cup a 1 | 0,44 |
| Italy - CAAM 2852 | M | 43 | Amb a 1 | 0 | Cry j 1 | 0 | Cup a 1 | 0,17 |
| Italy - CAAM 2853 | M | 43 | Amb a 1 | 0 | Cry j 1 | 1,54 | Cup a 1 | 4,69 |
| Italy - CAAM 2854 | M | 43 | Amb a 1 | 0 | Cry j 1 | 0 | Cup a 1 | 0,03 |
| Italy - CAAM 2855 | F | 43 | Amb a 1 | 0 | Cry j 1 | 1,32 | Cup a 1 | 2,42 |
| Italy - CAAM 2856 | M | 43 | Amb a 1 | 0 | Cry j 1 | 1,03 | Cup a 1 | 5,55 |
| Italy - CAAM 2857 | F | 43 | Amb a 1 | 0 | Cry j 1 | 0,09 | Cup a 1 | 0,68 |
| Italy - CAAM 2858 | F | 43 | Amb a 1 | 0 | Cry j 1 | 0 | Cup a 1 | 0,36 |
| Italy - CAAM 2859 | F | 43 | Amb a 1 | 0 | Cry j 1 | 0 | Cup a 1 | 0,81 |
| Italy - CAAM 2860 | F | 43 | Amb a 1 | 0 | Cry j 1 | 0 | Cup a 1 | 2,37 |
| Italy - CAAM 2861 | F | 43 | Amb a 1 | 0 | Cry j 1 | 1,24 | Cup a 1 | 2,26 |
| Italy - CAAM 2862 | M | 43 | Amb a 1 | 0 | Cry j 1 | 1,19 | Cup a 1 | 4,53 |
| Italy - CAAM 2863 | F | 43 | Amb a 1 | 0 | Cry j 1 | 15,72 | Cup a 1 | 51,21 |
| Italy - CAAM 2864 | F | 43 | Amb a 1 | 0 | Cry j 1 | 1,88 | Cup a 1 | 7,9 |
| Italy - CAAM 2865 | F | 43 | Amb a 1 | 0 | Cry j 1 | 2,88 | Cup a 1 | 7,89 |
| Italy - CAAM 2866 | F | 43 | Amb a 1 | 0 | Cry j 1 | 0 | Cup a 1 | 0,04 |
| Italy - CAAM 2867 | F | 43 | Amb a 1 | 0 | Cry j 1 | 0,07 | Cup a 1 | 2,29 |
| Italy - CAAM 2868 | F | 43 | Amb a 1 | 0 | Cry j 1 | 0 | Cup a 1 | 0,07 |
| Italy - CAAM 2869 | F | 43 | Amb a 1 | 0 | Cry j 1 | 1,15 | Cup a 1 | 8,01 |
| Italy - CAAM 2870 | M | 43 | Amb a 1 | 0 | Cry j 1 | 2,98 | Cup a 1 | 64,48 |
| Italy - CAAM 2871 | F | 43 | Amb a 1 | 0 | Cry j 1 | 0 | Cup a 1 | 1,02 |
| Italy - CAAM 2872 | M | 43 | Amb a 1 | 0 | Cry j 1 | 2,85 | Cup a 1 | 14,35 |
| Italy - CAAM 2873 | M | 43 | Amb a 1 | 0 | Cry j 1 | 0 | Cup a 1 | 0,5 |
| Italy - CAAM 2874 | M | 43 | Amb a 1 | 0 | Cry j 1 | 0 | Cup a 1 | 0,11 |
| Italy - CAAM 2875 | F | 43 | Amb a 1 | 0 | Cry j 1 | 3,47 | Cup a 1 | 6,88 |
| Italy - CAAM 2876 | M | 43 | Amb a 1 | 0 | Cry j 1 | 3,2 | Cup a 1 | 7,22 |
| Italy - CAAM 2877 | M | 43 | Amb a 1 | 0 | Cry j 1 | 1,02 | Cup a 1 | 4,46 |
| Italy - CAAM 2878 | M | 43 | Amb a 1 | 0 | Cry j 1 | 0,27 | Cup a 1 | 2,14 |
| Italy - CAAM 2879 | F | 43 | Amb a 1 | 0 | Cry j 1 | 1,34 | Cup a 1 | 6,11 |
| Italy - CAAM 2880 | F | 43 | Amb a 1 | 0 | Cry j 1 | 0,15 | Cup a 1 | 2,87 |
| Italy - CAAM 2881 | F | 43 | Amb a 1 | 0 | Cry j 1 | 0,06 | Cup a 1 | 1,63 |
| Italy - CAAM 2882 | F | 43 | Amb a 1 | 0 | Cry j 1 | 0,81 | Cup a 1 | 2,83 |
| Italy - CAAM 2883 | M | 43 | Amb a 1 | 0 | Cry j 1 | 12,25 | Cup a 1 | 43,94 |
| Italy - CAAM 2884 | F | 43 | Amb a 1 | 0 | Cry j 1 | 0,14 | Cup a 1 | 0,14 |
| Italy - CAAM 2885 | M | 43 | Amb a 1 | 0 | Cry j 1 | 0 | Cup a 1 | 1,32 |
| Italy - CAAM 2886 | M | 43 | Amb a 1 | 0 | Cry j 1 | 1,95 | Cup a 1 | 8,27 |
| Italy - CAAM 2887 | F | 43 | Amb a 1 | 0 | Cry j 1 | 0,47 | Cup a 1 | 8,12 |
| Italy - CAAM 2888 | F | 43 | Amb a 1 | 0 | Cry j 1 | 0,81 | Cup a 1 | 5,47 |
| Italy - CAAM 2889 | F | 43 | Amb a 1 | 0 | Cry j 1 | 0 | Cup a 1 | 4,41 |
| Italy - CAAM 2890 | F | 43 | Amb a 1 | 0 | Cry j 1 | 0,34 | Cup a 1 | 2,89 |
| Italy - CAAM 2891 | F | 43 | Amb a 1 | 0 | Cry j 1 | 2,81 | Cup a 1 | 9,36 |
| Italy - CAAM 2892 | M | 43 | Amb a 1 | 0 | Cry j 1 | 0,2 | Cup a 1 | 7,7 |
| Italy - CAAM 2893 | M | 43 | Amb a 1 | 0 | Cry j 1 | 0,86 | Cup a 1 | 3,36 |
| Italy - CAAM 2894 | F | 43 | Amb a 1 | 0 | Cry j 1 | 0,85 | Cup a 1 | 2,21 |
| Italy - CAAM 2895 | F | 43 | Amb a 1 | 0 | Cry j 1 | 0,97 | Cup a 1 | 3,73 |
| Italy - CAAM 2896 | F | 43 | Amb a 1 | 0 | Cry j 1 | 0,49 | Cup a 1 | 2,47 |
| Italy - CAAM 2897 | F | 43 | Amb a 1 | 0 | Cry j 1 | 0 | Cup a 1 | 0,58 |
| Italy - CAAM 2898 | F | 43 | Amb a 1 | 0 | Cry j 1 | 0 | Cup a 1 | 13,21 |
| Italy - CAAM 2899 | F | 43 | Amb a 1 | 0 | Cry j 1 | 0 | Cup a 1 | 0,93 |
| Italy - CAAM 2900 | F | 43 | Amb a 1 | 0 | Cry j 1 | 0 | Cup a 1 | 1,44 |
| Italy - CAAM 2901 | F | 43 | Amb a 1 | 0 | Cry j 1 | 0,46 | Cup a 1 | 15,82 |
| Italy - CAAM 2902 | M | 43 | Amb a 1 | 0 | Cry j 1 | 0 | Cup a 1 | 1,06 |
| Italy - CAAM 2903 | F | 43 | Amb a 1 | 0 | Cry j 1 | 0 | Cup a 1 | 0,53 |
| Italy - CAAM 2904 | F | 43 | Amb a 1 | 0 | Cry j 1 | 0 | Cup a 1 | 0,32 |
| Italy - CAAM 2905 | F | 43 | Amb a 1 | 0 | Cry j 1 | 0 | Cup a 1 | 2,09 |
| Italy - CAAM 2906 | F | 43 | Amb a 1 | 0 | Cry j 1 | 0 | Cup a 1 | 2 |
| Italy - CAAM 2907 | M | 43 | Amb a 1 | 0 | Cry j 1 | 0 | Cup a 1 | 0,38 |
| Italy - CAAM 2908 | M | 43 | Amb a 1 | 0 | Cry j 1 | 0,17 | Cup a 1 | 1,26 |
| Italy - CAAM 2909 | F | 43 | Amb a 1 | 0 | Cry j 1 | 0,2 | Cup a 1 | 1,84 |
| Italy - CAAM 2910 | M | 43 | Amb a 1 | 0 | Cry j 1 | 0,77 | Cup a 1 | 5,72 |
| Italy - CAAM 2911 | M | 43 | Amb a 1 | 0 | Cry j 1 | 0 | Cup a 1 | 0,69 |
| Italy - CAAM 2912 | F | 43 | Amb a 1 | 0 | Cry j 1 | 0,68 | Cup a 1 | 5,25 |
| Italy - CAAM 2913 | F | 43 | Amb a 1 | 0 | Cry j 1 | 0 | Cup a 1 | 0,55 |
| Italy - CAAM 2914 | F | 43 | Amb a 1 | 0 | Cry j 1 | 1,8 | Cup a 1 | 15,89 |
| Italy - CAAM 2915 | F | 43 | Amb a 1 | 0 | Cry j 1 | 1,74 | Cup a 1 | 43,87 |
| Italy - CAAM 2916 | M | 43 | Amb a 1 | 0 | Cry j 1 | 0 | Cup a 1 | 0,28 |
| Italy - CAAM 2917 | F | 43 | Amb a 1 | 0 | Cry j 1 | 6,37 | Cup a 1 | 24,81 |
| Italy - CAAM 2918 | M | 43 | Amb a 1 | 0 | Cry j 1 | 3,37 | Cup a 1 | 28,99 |
| Italy - CAAM 2919 | M | 43 | Amb a 1 | 0 | Cry j 1 | 2,91 | Cup a 1 | 34,74 |
| Italy - CAAM 2920 | F | 43 | Amb a 1 | 0 | Cry j 1 | 3,93 | Cup a 1 | 30,05 |
| Italy - CAAM 2921 | F | 43 | Amb a 1 | 0 | Cry j 1 | 0,21 | Cup a 1 | 8,53 |
| Italy - CAAM 2922 | M | 43 | Amb a 1 | 0 | Cry j 1 | 1,33 | Cup a 1 | 7,63 |
| Italy - CAAM 2923 | F | 43 | Amb a 1 | 0 | Cry j 1 | 3,82 | Cup a 1 | 37,56 |
| Italy - CAAM 2924 | F | 43 | Amb a 1 | 0 | Cry j 1 | 0 | Cup a 1 | 4,26 |
| Italy - CAAM 2925 | M | 43 | Amb a 1 | 0 | Cry j 1 | 0,22 | Cup a 1 | 8,87 |
| Italy - CAAM 2926 | M | 43 | Amb a 1 | 0 | Cry j 1 | 0 | Cup a 1 | 0,71 |
| Italy - CAAM 2927 | F | 43 | Amb a 1 | 0 | Cry j 1 | 0 | Cup a 1 | 7,7 |
| Italy - CAAM 2928 | F | 43 | Amb a 1 | 0 | Cry j 1 | 0,13 | Cup a 1 | 4,05 |
| Italy - CAAM 2929 | M | 43 | Amb a 1 | 0 | Cry j 1 | 0 | Cup a 1 | 5,9 |
| Italy - CAAM 2930 | F | 43 | Amb a 1 | 0 | Cry j 1 | 0 | Cup a 1 | 2 |
| Italy - CAAM 2931 | F | 43 | Amb a 1 | 0 | Cry j 1 | 0,5 | Cup a 1 | 12 |
| Italy - CAAM 2932 | F | 43 | Amb a 1 | 0 | Cry j 1 | 0 | Cup a 1 | 0,08 |
| Italy - CAAM 2933 | F | 43 | Amb a 1 | 0 | Cry j 1 | 0 | Cup a 1 | 0,1 |
| Italy - CAAM 2934 | M | 43 | Amb a 1 | 0 | Cry j 1 | 0,51 | Cup a 1 | 9,55 |
| Italy - CAAM 2935 | F | 43 | Amb a 1 | 0 | Cry j 1 | 1,3 | Cup a 1 | 15,35 |
| Italy - CAAM 2936 | F | 43 | Amb a 1 | 0 | Cry j 1 | 0,5 | Cup a 1 | 4,72 |
| Italy - CAAM 2937 | F | 43 | Amb a 1 | 0 | Cry j 1 | 1,88 | Cup a 1 | 0,95 |
| Italy - CAAM 2938 | M | 43 | Amb a 1 | 0 | Cry j 1 | 0,07 | Cup a 1 | 0,98 |
| Italy - CAAM 2939 | F | 42 | Amb a 1 | 0 | Cry j 1 | 0 | Cup a 1 | 0,41 |
| Italy - CAAM 2940 | F | 42 | Amb a 1 | 0 | Cry j 1 | 0,05 | Cup a 1 | 0,41 |
| Italy - CAAM 2941 | M | 42 | Amb a 1 | 0 | Cry j 1 | 2,46 | Cup a 1 | 2,96 |
| Italy - CAAM 2942 | F | 42 | Amb a 1 | 0 | Cry j 1 | 0 | Cup a 1 | 1,74 |
| Italy - CAAM 2943 | M | 42 | Amb a 1 | 0 | Cry j 1 | 0 | Cup a 1 | 6,32 |
| Italy - CAAM 2944 | F | 42 | Amb a 1 | 0 | Cry j 1 | 0,47 | Cup a 1 | 4,86 |
| Italy - CAAM 2945 | F | 42 | Amb a 1 | 0 | Cry j 1 | 0,87 | Cup a 1 | 12,85 |
| Italy - CAAM 2946 | M | 42 | Amb a 1 | 0 | Cry j 1 | 1,04 | Cup a 1 | 2,22 |
| Italy - CAAM 2947 | M | 42 | Amb a 1 | 0 | Cry j 1 | 3,68 | Cup a 1 | 3,91 |
| Italy - CAAM 2948 | M | 42 | Amb a 1 | 0 | Cry j 1 | 0 | Cup a 1 | 0,17 |
| Italy - CAAM 2949 | F | 42 | Amb a 1 | 0 | Cry j 1 | 0 | Cup a 1 | 7,3 |
| Italy - CAAM 2950 | F | 42 | Amb a 1 | 0 | Cry j 1 | 0,21 | Cup a 1 | 4,21 |
| Italy - CAAM 2951 | F | 42 | Amb a 1 | 0 | Cry j 1 | 0 | Cup a 1 | 1,06 |
| Italy - CAAM 2952 | F | 42 | Amb a 1 | 0 | Cry j 1 | 3,84 | Cup a 1 | 41,15 |
| Italy - CAAM 2953 | M | 42 | Amb a 1 | 0 | Cry j 1 | 2,04 | Cup a 1 | 2,98 |
| Italy - CAAM 2954 | F | 42 | Amb a 1 | 0 | Cry j 1 | 2,33 | Cup a 1 | 3,07 |
| Italy - CAAM 2955 | M | 42 | Amb a 1 | 0 | Cry j 1 | 0 | Cup a 1 | 1,47 |
| Italy - CAAM 2956 | F | 42 | Amb a 1 | 0 | Cry j 1 | 1,62 | Cup a 1 | 1,68 |
| Italy - CAAM 2957 | M | 42 | Amb a 1 | 0 | Cry j 1 | 0,96 | Cup a 1 | 2,66 |
| Italy - CAAM 2958 | F | 42 | Amb a 1 | 0 | Cry j 1 | 0 | Cup a 1 | 0,49 |
| Italy - CAAM 2959 | M | 42 | Amb a 1 | 0 | Cry j 1 | 0 | Cup a 1 | 0,16 |
| Italy - CAAM 2960 | F | 42 | Amb a 1 | 0 | Cry j 1 | 5,47 | Cup a 1 | 19,83 |
| Italy - CAAM 2961 | F | 42 | Amb a 1 | 0 | Cry j 1 | 4,63 | Cup a 1 | 26,19 |
| Italy - CAAM 2962 | F | 42 | Amb a 1 | 0 | Cry j 1 | 3,33 | Cup a 1 | 11,66 |
| Italy - CAAM 2963 | F | 42 | Amb a 1 | 0 | Cry j 1 | 2,49 | Cup a 1 | 4,63 |
| Italy - CAAM 2964 | F | 42 | Amb a 1 | 0 | Cry j 1 | 3 | Cup a 1 | 5,5 |
| Italy - CAAM 2965 | F | 42 | Amb a 1 | 0 | Cry j 1 | 3,12 | Cup a 1 | 7,22 |
| Italy - CAAM 2966 | F | 42 | Amb a 1 | 0 | Cry j 1 | 0,53 | Cup a 1 | 8,55 |
| Italy - CAAM 2967 | M | 42 | Amb a 1 | 0 | Cry j 1 | 0,59 | Cup a 1 | 0,85 |
| Italy - CAAM 2968 | M | 42 | Amb a 1 | 0 | Cry j 1 | 2,11 | Cup a 1 | 1,41 |
| Italy - CAAM 2969 | M | 42 | Amb a 1 | 0 | Cry j 1 | 8,47 | Cup a 1 | 11,23 |
| Italy - CAAM 2970 | F | 42 | Amb a 1 | 0 | Cry j 1 | 0,3 | Cup a 1 | 0,23 |
| Italy - CAAM 2971 | F | 42 | Amb a 1 | 0 | Cry j 1 | 0,36 | Cup a 1 | 5,42 |
| Italy - CAAM 2972 | M | 42 | Amb a 1 | 0 | Cry j 1 | 16,9 | Cup a 1 | 5,42 |
| Italy - CAAM 2973 | M | 42 | Amb a 1 | 0 | Cry j 1 | 0 | Cup a 1 | 0,33 |
| Italy - CAAM 2974 | F | 42 | Amb a 1 | 0 | Cry j 1 | 6,73 | Cup a 1 | 0,6 |
| Italy - CAAM 2975 | M | 42 | Amb a 1 | 0 | Cry j 1 | 7,72 | Cup a 1 | 0 |
| Italy - CAAM 2976 | F | 42 | Amb a 1 | 0 | Cry j 1 | 3,91 | Cup a 1 | 8,18 |
| Italy - CAAM 2977 | F | 42 | Amb a 1 | 0 | Cry j 1 | 0,67 | Cup a 1 | 0 |
| Italy - CAAM 2978 | F | 42 | Amb a 1 | 0 | Cry j 1 | 3,72 | Cup a 1 | 18,45 |
| Italy - CAAM 2979 | F | 42 | Amb a 1 | 0 | Cry j 1 | 0 | Cup a 1 | 0,43 |
| Italy - CAAM 2980 | M | 42 | Amb a 1 | 0 | Cry j 1 | 0 | Cup a 1 | 0,32 |
| Italy - CAAM 2981 | M | 42 | Amb a 1 | 0 | Cry j 1 | 0 | Cup a 1 | 0,17 |
| Italy - CAAM 2982 | F | 42 | Amb a 1 | 0 | Cry j 1 | 2,77 | Cup a 1 | 30,88 |
| Italy - CAAM 2983 | F | 42 | Amb a 1 | 0 | Cry j 1 | 27,99 | Cup a 1 | 23,02 |
| Italy - CAAM 2984 | F | 42 | Amb a 1 | 0 | Cry j 1 | 0,42 | Cup a 1 | 0,2 |
| Italy - CAAM 2985 | F | 42 | Amb a 1 | 0 | Cry j 1 | 4,7 | Cup a 1 | 3,7 |
| Italy - CAAM 2986 | M | 42 | Amb a 1 | 0 | Cry j 1 | 1,23 | Cup a 1 | 1,21 |
| Italy - CAAM 2987 | M | 42 | Amb a 1 | 0 | Cry j 1 | 0,31 | Cup a 1 | 0,54 |
| Italy - CAAM 2988 | F | 42 | Amb a 1 | 0 | Cry j 1 | 1 | Cup a 1 | 2,92 |
| Italy - CAAM 2989 | F | 42 | Amb a 1 | 0 | Cry j 1 | 0,23 | Cup a 1 | 0,6 |
| Italy - CAAM 2990 | F | 42 | Amb a 1 | 0 | Cry j 1 | 1,38 | Cup a 1 | 1,18 |
| Italy - CAAM 2991 | M | 42 | Amb a 1 | 0 | Cry j 1 | 0,04 | Cup a 1 | 0,35 |
| Italy - CAAM 2992 | F | 42 | Amb a 1 | 0 | Cry j 1 | 0,51 | Cup a 1 | 1,42 |
| Italy - CAAM 2993 | F | 42 | Amb a 1 | 0 | Cry j 1 | 6,57 | Cup a 1 | 2,19 |
| Italy - CAAM 2994 | M | 42 | Amb a 1 | 0 | Cry j 1 | 7,73 | Cup a 1 | 7,7 |
| Italy - CAAM 2995 | M | 42 | Amb a 1 | 0 | Cry j 1 | 1,23 | Cup a 1 | 7,11 |
| Italy - CAAM 2996 | F | 42 | Amb a 1 | 0 | Cry j 1 | 0 | Cup a 1 | 3,75 |
| Italy - CAAM 2997 | F | 42 | Amb a 1 | 0 | Cry j 1 | 0 | Cup a 1 | 3,09 |
| Italy - CAAM 2998 | F | 42 | Amb a 1 | 0 | Cry j 1 | 0 | Cup a 1 | 0,74 |
| Italy - CAAM 2999 | M | 42 | Amb a 1 | 0 | Cry j 1 | 0 | Cup a 1 | 0,12 |
| Italy - CAAM 3000 | M | 42 | Amb a 1 | 0 | Cry j 1 | 2,71 | Cup a 1 | 9,24 |
| Italy - CAAM 3001 | F | 42 | Amb a 1 | 0 | Cry j 1 | 1,23 | Cup a 1 | 10,46 |
| Italy - CAAM 3002 | F | 42 | Amb a 1 | 0 | Cry j 1 | 1,45 | Cup a 1 | 2,64 |
| Italy - CAAM 3003 | F | 42 | Amb a 1 | 0 | Cry j 1 | 0,15 | Cup a 1 | 2,37 |
| Italy - CAAM 3004 | M | 42 | Amb a 1 | 0 | Cry j 1 | 0,07 | Cup a 1 | 0,44 |
| Italy - CAAM 3005 | M | 42 | Amb a 1 | 0 | Cry j 1 | 0 | Cup a 1 | 1,39 |
| Italy - CAAM 3006 | F | 42 | Amb a 1 | 0 | Cry j 1 | 0,08 | Cup a 1 | 0,68 |
| Italy - CAAM 3007 | M | 42 | Amb a 1 | 0 | Cry j 1 | 0,11 | Cup a 1 | 0,57 |
| Italy - CAAM 3008 | F | 42 | Amb a 1 | 0 | Cry j 1 | 0,25 | Cup a 1 | 3,64 |
| Italy - CAAM 3009 | M | 42 | Amb a 1 | 0 | Cry j 1 | 0,88 | Cup a 1 | 8,27 |
| Italy - CAAM 3010 | M | 42 | Amb a 1 | 0 | Cry j 1 | 0,12 | Cup a 1 | 1,17 |
| Italy - CAAM 3011 | M | 42 | Amb a 1 | 0 | Cry j 1 | 8,58 | Cup a 1 | 14,65 |
| Italy - CAAM 3012 | F | 42 | Amb a 1 | 0 | Cry j 1 | 3,98 | Cup a 1 | 8,52 |
| Italy - CAAM 3013 | F | 42 | Amb a 1 | 0 | Cry j 1 | 0,85 | Cup a 1 | 1,48 |
| Italy - CAAM 3014 | M | 42 | Amb a 1 | 0 | Cry j 1 | 0,17 | Cup a 1 | 3,39 |
| Italy - CAAM 3015 | M | 42 | Amb a 1 | 0 | Cry j 1 | 0,21 | Cup a 1 | 2,04 |
| Italy - CAAM 3016 | F | 42 | Amb a 1 | 0 | Cry j 1 | 0 | Cup a 1 | 0,33 |
| Italy - CAAM 3017 | F | 42 | Amb a 1 | 0 | Cry j 1 | 1,22 | Cup a 1 | 8,6 |
| Italy - CAAM 3018 | M | 42 | Amb a 1 | 0 | Cry j 1 | 0 | Cup a 1 | 0,29 |
| Italy - CAAM 3019 | F | 42 | Amb a 1 | 0 | Cry j 1 | 0,04 | Cup a 1 | 1,53 |
| Italy - CAAM 3020 | F | 42 | Amb a 1 | 0 | Cry j 1 | 0 | Cup a 1 | 0,29 |
| Italy - CAAM 3021 | F | 42 | Amb a 1 | 0 | Cry j 1 | 0 | Cup a 1 | 1,34 |
| Italy - CAAM 3022 | M | 42 | Amb a 1 | 0 | Cry j 1 | 0,5 | Cup a 1 | 8,99 |
| Italy - CAAM 3023 | F | 42 | Amb a 1 | 0 | Cry j 1 | 5,63 | Cup a 1 | 18,44 |
| Italy - CAAM 3024 | F | 42 | Amb a 1 | 0 | Cry j 1 | 0,37 | Cup a 1 | 2,21 |
| Italy - CAAM 3025 | M | 42 | Amb a 1 | 0 | Cry j 1 | 1,42 | Cup a 1 | 2,97 |
| Italy - CAAM 3026 | F | 42 | Amb a 1 | 0 | Cry j 1 | 1,14 | Cup a 1 | 6,72 |
| Italy - CAAM 3027 | F | 42 | Amb a 1 | 0 | Cry j 1 | 0 | Cup a 1 | 1,56 |
| Italy - CAAM 3028 | F | 42 | Amb a 1 | 0 | Cry j 1 | 0,3 | Cup a 1 | 5,98 |
| Italy - CAAM 3029 | F | 42 | Amb a 1 | 0 | Cry j 1 | 2,32 | Cup a 1 | 2,62 |
| Italy - CAAM 3030 | F | 42 | Amb a 1 | 0 | Cry j 1 | 0 | Cup a 1 | 0,24 |
| Italy - CAAM 3031 | F | 42 | Amb a 1 | 0 | Cry j 1 | 0,08 | Cup a 1 | 1,6 |
| Italy - CAAM 3032 | F | 42 | Amb a 1 | 0 | Cry j 1 | 2,02 | Cup a 1 | 7,11 |
| Italy - CAAM 3033 | F | 42 | Amb a 1 | 0 | Cry j 1 | 0 | Cup a 1 | 0,41 |
| Italy - CAAM 3034 | M | 42 | Amb a 1 | 0 | Cry j 1 | 0,93 | Cup a 1 | 1,42 |
| Italy - CAAM 3035 | F | 42 | Amb a 1 | 0 | Cry j 1 | 5,44 | Cup a 1 | 21,02 |
| Italy - CAAM 3036 | F | 42 | Amb a 1 | 0 | Cry j 1 | 0 | Cup a 1 | 2,7 |
| Italy - CAAM 3037 | M | 42 | Amb a 1 | 0 | Cry j 1 | 0 | Cup a 1 | 0,67 |
| Italy - CAAM 3038 | F | 42 | Amb a 1 | 0 | Cry j 1 | 0,69 | Cup a 1 | 2,58 |
| Italy - CAAM 3039 | M | 42 | Amb a 1 | 0 | Cry j 1 | 0,47 | Cup a 1 | 6,69 |
| Italy - CAAM 3040 | F | 42 | Amb a 1 | 0 | Cry j 1 | 0,36 | Cup a 1 | 0,71 |
| Italy - CAAM 3041 | F | 42 | Amb a 1 | 0 | Cry j 1 | 0 | Cup a 1 | 0,06 |
| Italy - CAAM 3042 | M | 42 | Amb a 1 | 0 | Cry j 1 | 0,05 | Cup a 1 | 2,18 |
| Italy - CAAM 3043 | F | 42 | Amb a 1 | 0 | Cry j 1 | 1,5 | Cup a 1 | 4,89 |
| Italy - CAAM 3044 | F | 42 | Amb a 1 | 0 | Cry j 1 | 5,83 | Cup a 1 | 12,18 |
| Italy - CAAM 3045 | M | 42 | Amb a 1 | 0 | Cry j 1 | 0 | Cup a 1 | 2,35 |
| Italy - CAAM 3046 | M | 42 | Amb a 1 | 0 | Cry j 1 | 0 | Cup a 1 | 1,66 |
| Italy - CAAM 3047 | F | 42 | Amb a 1 | 0 | Cry j 1 | 0,29 | Cup a 1 | 0,86 |
| Italy - CAAM 3048 | M | 42 | Amb a 1 | 0 | Cry j 1 | 0 | Cup a 1 | 2,68 |
| Italy - CAAM 3049 | F | 42 | Amb a 1 | 0 | Cry j 1 | 0 | Cup a 1 | 4,41 |
| Italy - CAAM 3050 | M | 42 | Amb a 1 | 0 | Cry j 1 | 0,55 | Cup a 1 | 1,97 |
| Italy - CAAM 3051 | F | 42 | Amb a 1 | 0 | Cry j 1 | 0,56 | Cup a 1 | 0,75 |
| Italy - CAAM 3052 | M | 42 | Amb a 1 | 0 | Cry j 1 | 0 | Cup a 1 | 0,28 |
| Italy - CAAM 3053 | M | 42 | Amb a 1 | 0 | Cry j 1 | 2,74 | Cup a 1 | 2,02 |
| Italy - CAAM 3054 | F | 42 | Amb a 1 | 0 | Cry j 1 | 0 | Cup a 1 | 1,28 |
| Italy - CAAM 3055 | F | 42 | Amb a 1 | 0 | Cry j 1 | 0 | Cup a 1 | 4,02 |
| Italy - CAAM 3056 | M | 42 | Amb a 1 | 0 | Cry j 1 | 0,07 | Cup a 1 | 0,85 |
| Italy - CAAM 3057 | F | 42 | Amb a 1 | 0 | Cry j 1 | 4,58 | Cup a 1 | 18,05 |
| Italy - CAAM 3058 | F | 42 | Amb a 1 | 0 | Cry j 1 | 0,16 | Cup a 1 | 0,26 |
| Italy - CAAM 3059 | M | 42 | Amb a 1 | 0 | Cry j 1 | 1 | Cup a 1 | 1,5 |
| Italy - CAAM 3060 | F | 42 | Amb a 1 | 0 | Cry j 1 | 0,57 | Cup a 1 | 5,11 |
| Italy - CAAM 3061 | F | 42 | Amb a 1 | 0 | Cry j 1 | 0,48 | Cup a 1 | 1,8 |
| Italy - CAAM 3062 | F | 42 | Amb a 1 | 0 | Cry j 1 | 0,04 | Cup a 1 | 6,66 |
| Italy - CAAM 3063 | M | 42 | Amb a 1 | 0 | Cry j 1 | 5,24 | Cup a 1 | 11,76 |
| Italy - CAAM 3064 | M | 42 | Amb a 1 | 0 | Cry j 1 | 0,32 | Cup a 1 | 0,21 |
| Italy - CAAM 3065 | M | 42 | Amb a 1 | 0 | Cry j 1 | 1,41 | Cup a 1 | 6,7 |
| Italy - CAAM 3066 | F | 42 | Amb a 1 | 0 | Cry j 1 | 3,38 | Cup a 1 | 5,85 |
| Italy - CAAM 3067 | M | 42 | Amb a 1 | 0 | Cry j 1 | 0,13 | Cup a 1 | 10,77 |
| Italy - CAAM 3068 | F | 42 | Amb a 1 | 0 | Cry j 1 | 0 | Cup a 1 | 0,07 |
| Italy - CAAM 3069 | F | 42 | Amb a 1 | 0 | Cry j 1 | 7,24 | Cup a 1 | 26,84 |
| Italy - CAAM 3070 | F | 42 | Amb a 1 | 0 | Cry j 1 | 0 | Cup a 1 | 0,69 |
| Italy - CAAM 3071 | M | 42 | Amb a 1 | 0 | Cry j 1 | 0,28 | Cup a 1 | 9,02 |
| Italy - CAAM 3072 | M | 42 | Amb a 1 | 0 | Cry j 1 | 0,1 | Cup a 1 | 3,5 |
| Italy - CAAM 3073 | F | 42 | Amb a 1 | 0 | Cry j 1 | 11,86 | Cup a 1 | 20,52 |
| Italy - CAAM 3074 | F | 42 | Amb a 1 | 0 | Cry j 1 | 1,67 | Cup a 1 | 4,26 |
| Italy - CAAM 3075 | M | 42 | Amb a 1 | 0 | Cry j 1 | 1,2 | Cup a 1 | 5,36 |
| Italy - CAAM 3076 | M | 42 | Amb a 1 | 0 | Cry j 1 | 0,47 | Cup a 1 | 4,7 |
| Italy - CAAM 3077 | F | 42 | Amb a 1 | 0 | Cry j 1 | 0,57 | Cup a 1 | 4 |
| Italy - CAAM 3078 | F | 42 | Amb a 1 | 0 | Cry j 1 | 4,91 | Cup a 1 | 10,4 |
| Italy - CAAM 3079 | M | 42 | Amb a 1 | 0 | Cry j 1 | 5,1 | Cup a 1 | 13,89 |
| Italy - CAAM 3080 | M | 42 | Amb a 1 | 0 | Cry j 1 | 4,86 | Cup a 1 | 41,78 |
| Italy - CAAM 3081 | F | 42 | Amb a 1 | 0 | Cry j 1 | 0 | Cup a 1 | 2,98 |
| Italy - CAAM 3082 | M | 42 | Amb a 1 | 0 | Cry j 1 | 2,18 | Cup a 1 | 4,31 |
| Italy - CAAM 3083 | M | 42 | Amb a 1 | 0 | Cry j 1 | 0 | Cup a 1 | 2,73 |
| Italy - CAAM 3084 | M | 42 | Amb a 1 | 0 | Cry j 1 | 0,43 | Cup a 1 | 5,24 |
| Italy - CAAM 3085 | F | 42 | Amb a 1 | 0 | Cry j 1 | 0,18 | Cup a 1 | 1,27 |
| Italy - CAAM 3086 | M | 42 | Amb a 1 | 0 | Cry j 1 | 11,63 | Cup a 1 | 34,1 |
| Italy - CAAM 3087 | M | 42 | Amb a 1 | 0 | Cry j 1 | 7,22 | Cup a 1 | 26,35 |
| Italy - CAAM 3088 | F | 42 | Amb a 1 | 0 | Cry j 1 | 5,05 | Cup a 1 | 40,22 |
| Italy - CAAM 3089 | M | 42 | Amb a 1 | 0 | Cry j 1 | 0,16 | Cup a 1 | 6,81 |
| Italy - CAAM 3090 | M | 42 | Amb a 1 | 0 | Cry j 1 | 0 | Cup a 1 | 3,03 |
| Italy - CAAM 3091 | M | 42 | Amb a 1 | 0 | Cry j 1 | 0,83 | Cup a 1 | 5,58 |
| Italy - CAAM 3092 | F | 42 | Amb a 1 | 0 | Cry j 1 | 0 | Cup a 1 | 2,09 |
| Italy - CAAM 3093 | M | 42 | Amb a 1 | 0 | Cry j 1 | 0 | Cup a 1 | 1,51 |
| Italy - CAAM 3094 | M | 42 | Amb a 1 | 0 | Cry j 1 | 0,08 | Cup a 1 | 3,93 |
| Italy - CAAM 3095 | M | 42 | Amb a 1 | 0 | Cry j 1 | 2,3 | Cup a 1 | 13,67 |
| Italy - CAAM 3096 | F | 42 | Amb a 1 | 0 | Cry j 1 | 0,26 | Cup a 1 | 9,57 |
| Italy - CAAM 3097 | M | 42 | Amb a 1 | 0 | Cry j 1 | 22,02 | Cup a 1 | 55,33 |
| Italy - CAAM 3098 | F | 42 | Amb a 1 | 0 | Cry j 1 | 3,98 | Cup a 1 | 24,56 |
| Italy - CAAM 3099 | F | 42 | Amb a 1 | 0 | Cry j 1 | 0 | Cup a 1 | 2,12 |
| Italy - CAAM 3100 | F | 42 | Amb a 1 | 0 | Cry j 1 | 0,82 | Cup a 1 | 12,72 |
| Italy - CAAM 3101 | F | 42 | Amb a 1 | 0 | Cry j 1 | 0,09 | Cup a 1 | 3,41 |
| Italy - CAAM 3102 | M | 42 | Amb a 1 | 0 | Cry j 1 | 0 | Cup a 1 | 1,26 |
| Italy - CAAM 3103 | F | 42 | Amb a 1 | 0 | Cry j 1 | 21,92 | Cup a 1 | 51,64 |
| Italy - CAAM 3104 | M | 42 | Amb a 1 | 0 | Cry j 1 | 16,89 | Cup a 1 | 50,54 |
| Italy - CAAM 3105 | F | 42 | Amb a 1 | 0 | Cry j 1 | 0 | Cup a 1 | 4,99 |
| Italy - CAAM 3106 | M | 42 | Amb a 1 | 0 | Cry j 1 | 0,62 | Cup a 1 | 31,64 |
| Italy - CAAM 3107 | F | 42 | Amb a 1 | 0 | Cry j 1 | 1,26 | Cup a 1 | 20,46 |
| Italy - CAAM 3108 | M | 42 | Amb a 1 | 0 | Cry j 1 | 0,52 | Cup a 1 | 9,83 |
| Italy - CAAM 3109 | F | 42 | Amb a 1 | 0 | Cry j 1 | 4,48 | Cup a 1 | 12,96 |
| Italy - CAAM 3110 | F | 42 | Amb a 1 | 0 | Cry j 1 | 0 | Cup a 1 | 0,12 |
| Italy - CAAM 3111 | M | 42 | Amb a 1 | 0 | Cry j 1 | 0 | Cup a 1 | 3,48 |
| Italy - CAAM 3112 | F | 42 | Amb a 1 | 0 | Cry j 1 | 0,88 | Cup a 1 | 8,11 |
| Italy - CAAM 3113 | M | 42 | Amb a 1 | 0 | Cry j 1 | 0 | Cup a 1 | 2,7 |
| Italy - CAAM 3114 | F | 42 | Amb a 1 | 0 | Cry j 1 | 0 | Cup a 1 | 0,27 |
| Italy - CAAM 3115 | F | 42 | Amb a 1 | 0 | Cry j 1 | 0,3 | Cup a 1 | 3,62 |
| Italy - CAAM 3116 | F | 42 | Amb a 1 | 0 | Cry j 1 | 0,4 | Cup a 1 | 10 |
| Italy - CAAM 3117 | M | 42 | Amb a 1 | 0 | Cry j 1 | 0 | Cup a 1 | 1,1 |
| Italy - CAAM 3118 | F | 42 | Amb a 1 | 0 | Cry j 1 | 0 | Cup a 1 | 13 |
| Italy - CAAM 3119 | M | 42 | Amb a 1 | 0 | Cry j 1 | 0 | Cup a 1 | 0,3 |
| Italy - CAAM 3120 | F | 42 | Amb a 1 | 0 | Cry j 1 | 3,8 | Cup a 1 | 7,8 |
| Italy - CAAM 3121 | F | 42 | Amb a 1 | 0 | Cry j 1 | 2,4 | Cup a 1 | 68 |
| Italy - CAAM 3122 | F | 42 | Amb a 1 | 0 | Cry j 1 | 11 | Cup a 1 | 91 |
| Italy - CAAM 3123 | F | 42 | Amb a 1 | 0 | Cry j 1 | 2,23 | Cup a 1 | 5,34 |
| Italy - CAAM 3124 | F | 42 | Amb a 1 | 0 | Cry j 1 | 3,74 | Cup a 1 | 13,81 |
| Italy - CAAM 3125 | F | 42 | Amb a 1 | 0 | Cry j 1 | 2,55 | Cup a 1 | 58,59 |
| Italy - CAAM 3126 | F | 42 | Amb a 1 | 0 | Cry j 1 | 0,55 | Cup a 1 | 27,18 |
| Italy - CAAM 3127 | M | 42 | Amb a 1 | 0 | Cry j 1 | 0,11 | Cup a 1 | 11,84 |
| Italy - CAAM 3128 | M | 42 | Amb a 1 | 0 | Cry j 1 | 0 | Cup a 1 | 0,32 |
| Italy - CAAM 3129 | F | 41 | Amb a 1 | 0 | Cry j 1 | 3,74 | Cup a 1 | 2,05 |
| Italy - CAAM 3130 | M | 41 | Amb a 1 | 0 | Cry j 1 | 0,26 | Cup a 1 | 2,01 |
| Italy - CAAM 3131 | F | 41 | Amb a 1 | 0 | Cry j 1 | 1,57 | Cup a 1 | 2,1 |
| Italy - CAAM 3132 | M | 41 | Amb a 1 | 0 | Cry j 1 | 0 | Cup a 1 | 1,07 |
| Italy - CAAM 3133 | M | 41 | Amb a 1 | 0 | Cry j 1 | 0,19 | Cup a 1 | 1,57 |
| Italy - CAAM 3134 | F | 41 | Amb a 1 | 0 | Cry j 1 | 0,69 | Cup a 1 | 7,89 |
| Italy - CAAM 3135 | F | 41 | Amb a 1 | 0 | Cry j 1 | 0,63 | Cup a 1 | 1,85 |
| Italy - CAAM 3136 | F | 41 | Amb a 1 | 0 | Cry j 1 | 0,75 | Cup a 1 | 4,83 |
| Italy - CAAM 3137 | M | 41 | Amb a 1 | 0 | Cry j 1 | 0 | Cup a 1 | 4,32 |
| Italy - CAAM 3138 | M | 41 | Amb a 1 | 0 | Cry j 1 | 1,2 | Cup a 1 | 1,45 |
| Italy - CAAM 3139 | F | 41 | Amb a 1 | 0 | Cry j 1 | 0 | Cup a 1 | 4,45 |
| Italy - CAAM 3140 | M | 41 | Amb a 1 | 0 | Cry j 1 | 1,2 | Cup a 1 | 5,01 |
| Italy - CAAM 3141 | F | 41 | Amb a 1 | 0 | Cry j 1 | 1,02 | Cup a 1 | 2,1 |
| Italy - CAAM 3142 | F | 41 | Amb a 1 | 0 | Cry j 1 | 13,05 | Cup a 1 | 58,53 |
| Italy - CAAM 3143 | M | 41 | Amb a 1 | 0 | Cry j 1 | 1,89 | Cup a 1 | 2,81 |
| Italy - CAAM 3144 | F | 41 | Amb a 1 | 0 | Cry j 1 | 0,92 | Cup a 1 | 1,59 |
| Italy - CAAM 3145 | F | 41 | Amb a 1 | 0 | Cry j 1 | 0 | Cup a 1 | 2,04 |
| Italy - CAAM 3146 | F | 41 | Amb a 1 | 0 | Cry j 1 | 3,46 | Cup a 1 | 1,69 |
| Italy - CAAM 3147 | F | 41 | Amb a 1 | 0 | Cry j 1 | 3,85 | Cup a 1 | 6,01 |
| Italy - CAAM 3148 | F | 41 | Amb a 1 | 0 | Cry j 1 | 0 | Cup a 1 | 1,15 |
| Italy - CAAM 3149 | M | 41 | Amb a 1 | 0 | Cry j 1 | 1,78 | Cup a 1 | 2,97 |
| Italy - CAAM 3150 | M | 41 | Amb a 1 | 0 | Cry j 1 | 0,92 | Cup a 1 | 1 |
| Italy - CAAM 3151 | F | 41 | Amb a 1 | 0 | Cry j 1 | 1,5 | Cup a 1 | 1,65 |
| Italy - CAAM 3152 | M | 41 | Amb a 1 | 0 | Cry j 1 | 0,35 | Cup a 1 | 0,84 |
| Italy - CAAM 3153 | F | 41 | Amb a 1 | 0 | Cry j 1 | 0 | Cup a 1 | 0,33 |
| Italy - CAAM 3154 | M | 41 | Amb a 1 | 0 | Cry j 1 | 0 | Cup a 1 | 4,73 |
| Italy - CAAM 3155 | F | 41 | Amb a 1 | 0 | Cry j 1 | 0 | Cup a 1 | 0,32 |
| Italy - CAAM 3156 | F | 41 | Amb a 1 | 0 | Cry j 1 | 1,65 | Cup a 1 | 12,29 |
| Italy - CAAM 3157 | F | 41 | Amb a 1 | 0 | Cry j 1 | 0 | Cup a 1 | 0,18 |
| Italy - CAAM 3158 | M | 41 | Amb a 1 | 0 | Cry j 1 | 1,09 | Cup a 1 | 2,98 |
| Italy - CAAM 3159 | F | 41 | Amb a 1 | 0 | Cry j 1 | 49,95 | Cup a 1 | 48,13 |
| Italy - CAAM 3160 | F | 41 | Amb a 1 | 0 | Cry j 1 | 0,51 | Cup a 1 | 0,25 |
| Italy - CAAM 3161 | F | 41 | Amb a 1 | 0 | Cry j 1 | 5,99 | Cup a 1 | 6,49 |
| Italy - CAAM 3162 | F | 41 | Amb a 1 | 0 | Cry j 1 | 1,94 | Cup a 1 | 0,66 |
| Italy - CAAM 3163 | F | 41 | Amb a 1 | 0 | Cry j 1 | 0,99 | Cup a 1 | 1,81 |
| Italy - CAAM 3164 | F | 41 | Amb a 1 | 0 | Cry j 1 | 0 | Cup a 1 | 4,33 |
| Italy - CAAM 3165 | F | 41 | Amb a 1 | 0 | Cry j 1 | 2,37 | Cup a 1 | 1,64 |
| Italy - CAAM 3166 | F | 41 | Amb a 1 | 0 | Cry j 1 | 0,12 | Cup a 1 | 0,12 |
| Italy - CAAM 3167 | F | 41 | Amb a 1 | 0 | Cry j 1 | 5,25 | Cup a 1 | 4 |
| Italy - CAAM 3168 | F | 41 | Amb a 1 | 0 | Cry j 1 | 5,54 | Cup a 1 | 4,89 |
| Italy - CAAM 3169 | F | 41 | Amb a 1 | 0 | Cry j 1 | 2,49 | Cup a 1 | 6,85 |
| Italy - CAAM 3170 | M | 41 | Amb a 1 | 0 | Cry j 1 | 0,39 | Cup a 1 | 0,38 |
| Italy - CAAM 3171 | M | 41 | Amb a 1 | 0 | Cry j 1 | 0,76 | Cup a 1 | 5,47 |
| Italy - CAAM 3172 | M | 41 | Amb a 1 | 0 | Cry j 1 | 1,02 | Cup a 1 | 3,37 |
| Italy - CAAM 3173 | M | 41 | Amb a 1 | 0 | Cry j 1 | 0 | Cup a 1 | 0,31 |
| Italy - CAAM 3174 | F | 41 | Amb a 1 | 0 | Cry j 1 | 2,16 | Cup a 1 | 1,87 |
| Italy - CAAM 3175 | M | 41 | Amb a 1 | 0 | Cry j 1 | 5,78 | Cup a 1 | 4,71 |
| Italy - CAAM 3176 | F | 41 | Amb a 1 | 0 | Cry j 1 | 3,09 | Cup a 1 | 2,72 |
| Italy - CAAM 3177 | F | 41 | Amb a 1 | 0 | Cry j 1 | 0 | Cup a 1 | 0,98 |
| Italy - CAAM 3178 | M | 41 | Amb a 1 | 0 | Cry j 1 | 1,34 | Cup a 1 | 2,55 |
| Italy - CAAM 3179 | F | 41 | Amb a 1 | 0 | Cry j 1 | 0 | Cup a 1 | 0,18 |
| Italy - CAAM 3180 | M | 41 | Amb a 1 | 0 | Cry j 1 | 1,72 | Cup a 1 | 1,18 |
| Italy - CAAM 3181 | F | 41 | Amb a 1 | 0 | Cry j 1 | 2,87 | Cup a 1 | 3,19 |
| Italy - CAAM 3182 | F | 41 | Amb a 1 | 0 | Cry j 1 | 0 | Cup a 1 | 0,83 |
| Italy - CAAM 3183 | F | 41 | Amb a 1 | 0 | Cry j 1 | 5,49 | Cup a 1 | 17,01 |
| Italy - CAAM 3184 | F | 41 | Amb a 1 | 0 | Cry j 1 | 2,06 | Cup a 1 | 6,44 |
| Italy - CAAM 3185 | F | 41 | Amb a 1 | 0 | Cry j 1 | 2,67 | Cup a 1 | 6,32 |
| Italy - CAAM 3186 | M | 41 | Amb a 1 | 0 | Cry j 1 | 0,46 | Cup a 1 | 1,64 |
| Italy - CAAM 3187 | M | 41 | Amb a 1 | 0 | Cry j 1 | 1,23 | Cup a 1 | 2,52 |
| Italy - CAAM 3188 | F | 41 | Amb a 1 | 0 | Cry j 1 | 1,54 | Cup a 1 | 6,76 |
| Italy - CAAM 3189 | F | 41 | Amb a 1 | 0 | Cry j 1 | 0,24 | Cup a 1 | 0,23 |
| Italy - CAAM 3190 | F | 41 | Amb a 1 | 0 | Cry j 1 | 2,04 | Cup a 1 | 3,86 |
| Italy - CAAM 3191 | F | 41 | Amb a 1 | 0 | Cry j 1 | 0,51 | Cup a 1 | 0,95 |
| Italy - CAAM 3192 | F | 41 | Amb a 1 | 0 | Cry j 1 | 0,41 | Cup a 1 | 1,65 |
| Italy - CAAM 3193 | F | 41 | Amb a 1 | 0 | Cry j 1 | 0 | Cup a 1 | 1,02 |
| Italy - CAAM 3194 | M | 41 | Amb a 1 | 0 | Cry j 1 | 4,05 | Cup a 1 | 6,71 |
| Italy - CAAM 3195 | F | 41 | Amb a 1 | 0 | Cry j 1 | 2,87 | Cup a 1 | 13,57 |
| Italy - CAAM 3196 | M | 41 | Amb a 1 | 0 | Cry j 1 | 0 | Cup a 1 | 1,2 |
| Italy - CAAM 3197 | F | 41 | Amb a 1 | 0 | Cry j 1 | 0 | Cup a 1 | 0,09 |
| Italy - CAAM 3198 | M | 41 | Amb a 1 | 0 | Cry j 1 | 0,36 | Cup a 1 | 1,47 |
| Italy - CAAM 3199 | F | 41 | Amb a 1 | 0 | Cry j 1 | 5,09 | Cup a 1 | 18,94 |
| Italy - CAAM 3200 | F | 41 | Amb a 1 | 0 | Cry j 1 | 4,36 | Cup a 1 | 19,46 |
| Italy - CAAM 3201 | F | 41 | Amb a 1 | 0 | Cry j 1 | 0,6 | Cup a 1 | 2,34 |
| Italy - CAAM 3202 | M | 41 | Amb a 1 | 0 | Cry j 1 | 0,81 | Cup a 1 | 3,36 |
| Italy - CAAM 3203 | F | 41 | Amb a 1 | 0 | Cry j 1 | 0,05 | Cup a 1 | 0,18 |
| Italy - CAAM 3204 | F | 41 | Amb a 1 | 0 | Cry j 1 | 0 | Cup a 1 | 0,04 |
| Italy - CAAM 3205 | M | 41 | Amb a 1 | 0 | Cry j 1 | 0 | Cup a 1 | 0,12 |
| Italy - CAAM 3206 | F | 41 | Amb a 1 | 0 | Cry j 1 | 1,14 | Cup a 1 | 11,23 |
| Italy - CAAM 3207 | M | 41 | Amb a 1 | 0 | Cry j 1 | 0 | Cup a 1 | 0,9 |
| Italy - CAAM 3208 | M | 41 | Amb a 1 | 0 | Cry j 1 | 0,74 | Cup a 1 | 2,1 |
| Italy - CAAM 3209 | M | 41 | Amb a 1 | 0 | Cry j 1 | 0,2 | Cup a 1 | 1,42 |
| Italy - CAAM 3210 | M | 41 | Amb a 1 | 0 | Cry j 1 | 1,09 | Cup a 1 | 9,28 |
| Italy - CAAM 3211 | M | 41 | Amb a 1 | 0 | Cry j 1 | 0 | Cup a 1 | 1,43 |
| Italy - CAAM 3212 | F | 41 | Amb a 1 | 0 | Cry j 1 | 0,38 | Cup a 1 | 1,68 |
| Italy - CAAM 3213 | F | 41 | Amb a 1 | 0 | Cry j 1 | 2,41 | Cup a 1 | 1,28 |
| Italy - CAAM 3214 | M | 41 | Amb a 1 | 0 | Cry j 1 | 1,09 | Cup a 1 | 3,98 |
| Italy - CAAM 3215 | M | 41 | Amb a 1 | 0 | Cry j 1 | 0 | Cup a 1 | 0,04 |
| Italy - CAAM 3216 | M | 41 | Amb a 1 | 0 | Cry j 1 | 0,27 | Cup a 1 | 0,94 |
| Italy - CAAM 3217 | F | 41 | Amb a 1 | 0 | Cry j 1 | 9,6 | Cup a 1 | 13,93 |
| Italy - CAAM 3218 | F | 41 | Amb a 1 | 0 | Cry j 1 | 0 | Cup a 1 | 2 |
| Italy - CAAM 3219 | F | 41 | Amb a 1 | 0 | Cry j 1 | 0,55 | Cup a 1 | 3 |
| Italy - CAAM 3220 | M | 41 | Amb a 1 | 0 | Cry j 1 | 0 | Cup a 1 | 0,29 |
| Italy - CAAM 3221 | F | 41 | Amb a 1 | 0 | Cry j 1 | 1,1 | Cup a 1 | 2,94 |
| Italy - CAAM 3222 | M | 41 | Amb a 1 | 0 | Cry j 1 | 1,45 | Cup a 1 | 4,55 |
| Italy - CAAM 3223 | F | 41 | Amb a 1 | 0 | Cry j 1 | 0 | Cup a 1 | 3,63 |
| Italy - CAAM 3224 | F | 41 | Amb a 1 | 0 | Cry j 1 | 2,14 | Cup a 1 | 6,14 |
| Italy - CAAM 3225 | F | 41 | Amb a 1 | 0 | Cry j 1 | 0 | Cup a 1 | 0,52 |
| Italy - CAAM 3226 | F | 41 | Amb a 1 | 0 | Cry j 1 | 0,43 | Cup a 1 | 0,88 |
| Italy - CAAM 3227 | F | 41 | Amb a 1 | 0 | Cry j 1 | 0,91 | Cup a 1 | 3,78 |
| Italy - CAAM 3228 | F | 41 | Amb a 1 | 0 | Cry j 1 | 0,46 | Cup a 1 | 1,66 |
| Italy - CAAM 3229 | F | 41 | Amb a 1 | 0 | Cry j 1 | 0 | Cup a 1 | 0,14 |
| Italy - CAAM 3230 | F | 41 | Amb a 1 | 0 | Cry j 1 | 0 | Cup a 1 | 3,24 |
| Italy - CAAM 3231 | F | 41 | Amb a 1 | 0 | Cry j 1 | 0 | Cup a 1 | 56 |
| Italy - CAAM 3232 | M | 41 | Amb a 1 | 0 | Cry j 1 | 0,25 | Cup a 1 | 0,91 |
| Italy - CAAM 3233 | M | 41 | Amb a 1 | 0 | Cry j 1 | 0,12 | Cup a 1 | 2,72 |
| Italy - CAAM 3234 | M | 41 | Amb a 1 | 0 | Cry j 1 | 2,35 | Cup a 1 | 6,2 |
| Italy - CAAM 3235 | F | 41 | Amb a 1 | 0 | Cry j 1 | 0,72 | Cup a 1 | 2,54 |
| Italy - CAAM 3236 | F | 41 | Amb a 1 | 0 | Cry j 1 | 0 | Cup a 1 | 0,32 |
| Italy - CAAM 3237 | F | 41 | Amb a 1 | 0 | Cry j 1 | 0,13 | Cup a 1 | 0,29 |
| Italy - CAAM 3238 | F | 41 | Amb a 1 | 0 | Cry j 1 | 0 | Cup a 1 | 0,19 |
| Italy - CAAM 3239 | F | 41 | Amb a 1 | 0 | Cry j 1 | 0,37 | Cup a 1 | 1,44 |
| Italy - CAAM 3240 | M | 41 | Amb a 1 | 0 | Cry j 1 | 0,21 | Cup a 1 | 0,31 |
| Italy - CAAM 3241 | M | 41 | Amb a 1 | 0 | Cry j 1 | 0,17 | Cup a 1 | 2,22 |
| Italy - CAAM 3242 | F | 41 | Amb a 1 | 0 | Cry j 1 | 0 | Cup a 1 | 0,09 |
| Italy - CAAM 3243 | M | 41 | Amb a 1 | 0 | Cry j 1 | 1,34 | Cup a 1 | 10,63 |
| Italy - CAAM 3244 | M | 41 | Amb a 1 | 0 | Cry j 1 | 0,46 | Cup a 1 | 0,8 |
| Italy - CAAM 3245 | F | 41 | Amb a 1 | 0 | Cry j 1 | 0,24 | Cup a 1 | 6,23 |
| Italy - CAAM 3246 | F | 41 | Amb a 1 | 0 | Cry j 1 | 0,27 | Cup a 1 | 1,12 |
| Italy - CAAM 3247 | F | 41 | Amb a 1 | 0 | Cry j 1 | 0,41 | Cup a 1 | 2,81 |
| Italy - CAAM 3248 | M | 41 | Amb a 1 | 0 | Cry j 1 | 3,44 | Cup a 1 | 10,07 |
| Italy - CAAM 3249 | M | 41 | Amb a 1 | 0 | Cry j 1 | 3,05 | Cup a 1 | 5,2 |
| Italy - CAAM 3250 | M | 41 | Amb a 1 | 0 | Cry j 1 | 0 | Cup a 1 | 6,77 |
| Italy - CAAM 3251 | M | 41 | Amb a 1 | 0 | Cry j 1 | 0 | Cup a 1 | 0,21 |
| Italy - CAAM 3252 | F | 41 | Amb a 1 | 0 | Cry j 1 | 0,22 | Cup a 1 | 2,08 |
| Italy - CAAM 3253 | M | 41 | Amb a 1 | 0 | Cry j 1 | 0,18 | Cup a 1 | 1,68 |
| Italy - CAAM 3254 | M | 41 | Amb a 1 | 0 | Cry j 1 | 0,37 | Cup a 1 | 2,6 |
| Italy - CAAM 3255 | F | 41 | Amb a 1 | 0 | Cry j 1 | 3,22 | Cup a 1 | 8,2 |
| Italy - CAAM 3256 | M | 41 | Amb a 1 | 0 | Cry j 1 | 0,11 | Cup a 1 | 1,06 |
| Italy - CAAM 3257 | F | 41 | Amb a 1 | 0 | Cry j 1 | 0,22 | Cup a 1 | 1,67 |
| Italy - CAAM 3258 | M | 41 | Amb a 1 | 0 | Cry j 1 | 0 | Cup a 1 | 0,54 |
| Italy - CAAM 3259 | F | 41 | Amb a 1 | 0 | Cry j 1 | 0,21 | Cup a 1 | 1,73 |
| Italy - CAAM 3260 | F | 41 | Amb a 1 | 0 | Cry j 1 | 18,68 | Cup a 1 | 41,1 |
| Italy - CAAM 3261 | F | 41 | Amb a 1 | 0 | Cry j 1 | 4,28 | Cup a 1 | 22,06 |
| Italy - CAAM 3262 | M | 41 | Amb a 1 | 0 | Cry j 1 | 30,07 | Cup a 1 | 69,63 |
| Italy - CAAM 3263 | F | 41 | Amb a 1 | 0 | Cry j 1 | 5,42 | Cup a 1 | 43,71 |
| Italy - CAAM 3264 | F | 41 | Amb a 1 | 0 | Cry j 1 | 2,32 | Cup a 1 | 7,16 |
| Italy - CAAM 3265 | F | 41 | Amb a 1 | 0 | Cry j 1 | 38,82 | Cup a 1 | 69,3 |
| Italy - CAAM 3266 | F | 41 | Amb a 1 | 0 | Cry j 1 | 14,88 | Cup a 1 | 49,09 |
| Italy - CAAM 3267 | F | 41 | Amb a 1 | 0 | Cry j 1 | 0,46 | Cup a 1 | 13,77 |
| Italy - CAAM 3268 | F | 41 | Amb a 1 | 0 | Cry j 1 | 0 | Cup a 1 | 0,39 |
| Italy - CAAM 3269 | F | 41 | Amb a 1 | 0 | Cry j 1 | 0 | Cup a 1 | 6,42 |
| Italy - CAAM 3270 | F | 41 | Amb a 1 | 0 | Cry j 1 | 0 | Cup a 1 | 0,06 |
| Italy - CAAM 3271 | F | 41 | Amb a 1 | 0 | Cry j 1 | 3,3 | Cup a 1 | 39,44 |
| Italy - CAAM 3272 | M | 41 | Amb a 1 | 0 | Cry j 1 | 0,08 | Cup a 1 | 2,09 |
| Italy - CAAM 3273 | F | 41 | Amb a 1 | 0 | Cry j 1 | 0,44 | Cup a 1 | 5,88 |
| Italy - CAAM 3274 | M | 41 | Amb a 1 | 0 | Cry j 1 | 3 | Cup a 1 | 22,75 |
| Italy - CAAM 3275 | F | 41 | Amb a 1 | 0 | Cry j 1 | 0 | Cup a 1 | 2,65 |
| Italy - CAAM 3276 | M | 41 | Amb a 1 | 0 | Cry j 1 | 0,55 | Cup a 1 | 9,19 |
| Italy - CAAM 3277 | F | 41 | Amb a 1 | 0 | Cry j 1 | 0,06 | Cup a 1 | 1,4 |
| Italy - CAAM 3278 | F | 41 | Amb a 1 | 0 | Cry j 1 | 0 | Cup a 1 | 0,58 |
| Italy - CAAM 3279 | M | 41 | Amb a 1 | 0 | Cry j 1 | 6,16 | Cup a 1 | 29,55 |
| Italy - CAAM 3280 | F | 41 | Amb a 1 | 0 | Cry j 1 | 0,99 | Cup a 1 | 14,99 |
| Italy - CAAM 3281 | M | 41 | Amb a 1 | 0 | Cry j 1 | 1,05 | Cup a 1 | 1,67 |
| Italy - CAAM 3282 | M | 41 | Amb a 1 | 0 | Cry j 1 | 7,9 | Cup a 1 | 37,89 |
| Italy - CAAM 3283 | F | 41 | Amb a 1 | 0 | Cry j 1 | 0,09 | Cup a 1 | 0,95 |
| Italy - CAAM 3284 | M | 41 | Amb a 1 | 0 | Cry j 1 | 0 | Cup a 1 | 1,47 |
| Italy - CAAM 3285 | F | 41 | Amb a 1 | 0 | Cry j 1 | 1,52 | Cup a 1 | 15,2 |
| Italy - CAAM 3286 | F | 41 | Amb a 1 | 0 | Cry j 1 | 0,57 | Cup a 1 | 7,48 |
| Italy - CAAM 3287 | F | 41 | Amb a 1 | 0 | Cry j 1 | 0 | Cup a 1 | 1,5 |
| Italy - CAAM 3288 | F | 41 | Amb a 1 | 0 | Cry j 1 | 0,37 | Cup a 1 | 2,07 |
| Italy - CAAM 3289 | F | 41 | Amb a 1 | 0 | Cry j 1 | 0 | Cup a 1 | 0,7 |
| Italy - CAAM 3290 | F | 41 | Amb a 1 | 0 | Cry j 1 | 1 | Cup a 1 | 23 |
| Italy - CAAM 3291 | F | 41 | Amb a 1 | 0 | Cry j 1 | 1 | Cup a 1 | 32 |
| Italy - CAAM 3292 | F | 41 | Amb a 1 | 0 | Cry j 1 | 0 | Cup a 1 | 1,7 |
| Italy - CAAM 3293 | F | 41 | Amb a 1 | 0 | Cry j 1 | 0 | Cup a 1 | 0,8 |
| Italy - CAAM 3294 | M | 41 | Amb a 1 | 0 | Cry j 1 | 0,6 | Cup a 1 | 10 |
| Italy - CAAM 3295 | F | 41 | Amb a 1 | 0 | Cry j 1 | 0,09 | Cup a 1 | 3,68 |
| Italy - CAAM 3296 | F | 41 | Amb a 1 | 0 | Cry j 1 | 0 | Cup a 1 | 0,3 |
| Italy - CAAM 3297 | F | 41 | Amb a 1 | 0 | Cry j 1 | 0 | Cup a 1 | 0,06 |
| Italy - CAAM 3298 | F | 41 | Amb a 1 | 0 | Cry j 1 | 0 | Cup a 1 | 0,3 |
| Italy - CAAM 3299 | M | 41 | Amb a 1 | 0 | Cry j 1 | 0,47 | Cup a 1 | 7,4 |
| Italy - CAAM 3300 | F | 41 | Amb a 1 | 0 | Cry j 1 | 0,1 | Cup a 1 | 5,61 |
| Italy - CAAM 3301 | F | 41 | Amb a 1 | 0 | Cry j 1 | 0,2 | Cup a 1 | 5,43 |
| Italy - CAAM 3302 | F | 41 | Amb a 1 | 0 | Cry j 1 | 0 | Cup a 1 | 2,61 |
| Italy - CAAM 3303 | F | 41 | Amb a 1 | 0 | Cry j 1 | 0,11 | Cup a 1 | 1,11 |
| Italy - CAAM 3304 | F | 41 | Amb a 1 | 0 | Cry j 1 | 3,81 | Cup a 1 | 37,96 |
| Italy - CAAM 3305 | F | 41 | Amb a 1 | 0 | Cry j 1 | 3,04 | Cup a 1 | 19,39 |
| Italy - CAAM 3306 | M | 41 | Amb a 1 | 0 | Cry j 1 | 0,68 | Cup a 1 | 3,76 |
| Italy - CAAM 3307 | M | 41 | Amb a 1 | 0 | Cry j 1 | 2,07 | Cup a 1 | 15,43 |
| Italy - CAAM 3308 | F | 41 | Amb a 1 | 0 | Cry j 1 | 0,52 | Cup a 1 | 11,02 |
| Italy - CAAM 3309 | M | 41 | Amb a 1 | 0 | Cry j 1 | 0,53 | Cup a 1 | 3,91 |
| Italy - CAAM 3310 | M | 40 | Amb a 1 | 0 | Cry j 1 | 0,63 | Cup a 1 | 5,01 |
| Italy - CAAM 3311 | F | 40 | Amb a 1 | 0 | Cry j 1 | 5,07 | Cup a 1 | 26,93 |
| Italy - CAAM 3312 | F | 40 | Amb a 1 | 0 | Cry j 1 | 6,69 | Cup a 1 | 27,7 |
| Italy - CAAM 3313 | M | 40 | Amb a 1 | 0 | Cry j 1 | 1,83 | Cup a 1 | 1,33 |
| Italy - CAAM 3314 | M | 40 | Amb a 1 | 0 | Cry j 1 | 0,8 | Cup a 1 | 7,72 |
| Italy - CAAM 3315 | M | 40 | Amb a 1 | 0 | Cry j 1 | 0 | Cup a 1 | 1,09 |
| Italy - CAAM 3316 | F | 40 | Amb a 1 | 0 | Cry j 1 | 1,7 | Cup a 1 | 7,77 |
| Italy - CAAM 3317 | F | 40 | Amb a 1 | 0 | Cry j 1 | 1,08 | Cup a 1 | 1,51 |
| Italy - CAAM 3318 | M | 40 | Amb a 1 | 0 | Cry j 1 | 0 | Cup a 1 | 0,56 |
| Italy - CAAM 3319 | M | 40 | Amb a 1 | 0 | Cry j 1 | 1,94 | Cup a 1 | 7,51 |
| Italy - CAAM 3320 | F | 40 | Amb a 1 | 0 | Cry j 1 | 9,92 | Cup a 1 | 4,96 |
| Italy - CAAM 3321 | F | 40 | Amb a 1 | 0 | Cry j 1 | 0,52 | Cup a 1 | 4,98 |
| Italy - CAAM 3322 | F | 40 | Amb a 1 | 0 | Cry j 1 | 0 | Cup a 1 | 0,87 |
| Italy - CAAM 3323 | F | 40 | Amb a 1 | 0 | Cry j 1 | 0,93 | Cup a 1 | 6,8 |
| Italy - CAAM 3324 | F | 40 | Amb a 1 | 0 | Cry j 1 | 1,09 | Cup a 1 | 22,26 |
| Italy - CAAM 3325 | F | 40 | Amb a 1 | 0 | Cry j 1 | 1,09 | Cup a 1 | 2,09 |
| Italy - CAAM 3326 | F | 40 | Amb a 1 | 0 | Cry j 1 | 0 | Cup a 1 | 2,94 |
| Italy - CAAM 3327 | F | 40 | Amb a 1 | 0 | Cry j 1 | 9,94 | Cup a 1 | 44,57 |
| Italy - CAAM 3328 | F | 40 | Amb a 1 | 0 | Cry j 1 | 0 | Cup a 1 | 0,96 |
| Italy - CAAM 3329 | F | 40 | Amb a 1 | 0 | Cry j 1 | 0 | Cup a 1 | 2,52 |
| Italy - CAAM 3330 | F | 40 | Amb a 1 | 0 | Cry j 1 | 1,48 | Cup a 1 | 3,08 |
| Italy - CAAM 3331 | M | 40 | Amb a 1 | 0 | Cry j 1 | 0 | Cup a 1 | 2,94 |
| Italy - CAAM 3332 | M | 40 | Amb a 1 | 0 | Cry j 1 | 0,03 | Cup a 1 | 0,38 |
| Italy - CAAM 3333 | F | 40 | Amb a 1 | 0 | Cry j 1 | 0 | Cup a 1 | 0,66 |
| Italy - CAAM 3334 | M | 40 | Amb a 1 | 0 | Cry j 1 | 0,37 | Cup a 1 | 1,01 |
| Italy - CAAM 3335 | M | 40 | Amb a 1 | 0 | Cry j 1 | 2,52 | Cup a 1 | 6,11 |
| Italy - CAAM 3336 | F | 40 | Amb a 1 | 0 | Cry j 1 | 0,57 | Cup a 1 | 1,13 |
| Italy - CAAM 3337 | M | 40 | Amb a 1 | 0 | Cry j 1 | 0,88 | Cup a 1 | 0,96 |
| Italy - CAAM 3338 | F | 40 | Amb a 1 | 0 | Cry j 1 | 2,4 | Cup a 1 | 12,67 |
| Italy - CAAM 3339 | F | 40 | Amb a 1 | 0 | Cry j 1 | 0,31 | Cup a 1 | 4,11 |
| Italy - CAAM 3340 | F | 40 | Amb a 1 | 0 | Cry j 1 | 0 | Cup a 1 | 0,48 |
| Italy - CAAM 3341 | M | 40 | Amb a 1 | 0 | Cry j 1 | 0 | Cup a 1 | 0,58 |
| Italy - CAAM 3342 | F | 40 | Amb a 1 | 0 | Cry j 1 | 3,54 | Cup a 1 | 4,51 |
| Italy - CAAM 3343 | M | 40 | Amb a 1 | 0 | Cry j 1 | 0 | Cup a 1 | 0,84 |
| Italy - CAAM 3344 | F | 40 | Amb a 1 | 0 | Cry j 1 | 0,54 | Cup a 1 | 1,68 |
| Italy - CAAM 3345 | M | 40 | Amb a 1 | 0 | Cry j 1 | 1,08 | Cup a 1 | 0 |
| Italy - CAAM 3346 | M | 40 | Amb a 1 | 0 | Cry j 1 | 0,06 | Cup a 1 | 0,18 |
| Italy - CAAM 3347 | M | 40 | Amb a 1 | 0 | Cry j 1 | 0,17 | Cup a 1 | 0,28 |
| Italy - CAAM 3348 | M | 40 | Amb a 1 | 0 | Cry j 1 | 2,96 | Cup a 1 | 9,09 |
| Italy - CAAM 3349 | F | 40 | Amb a 1 | 0 | Cry j 1 | 0,34 | Cup a 1 | 0,12 |
| Italy - CAAM 3350 | F | 40 | Amb a 1 | 0 | Cry j 1 | 0,21 | Cup a 1 | 0,29 |
| Italy - CAAM 3351 | M | 40 | Amb a 1 | 0 | Cry j 1 | 0,63 | Cup a 1 | 0,6 |
| Italy - CAAM 3352 | F | 40 | Amb a 1 | 0 | Cry j 1 | 1,75 | Cup a 1 | 8,06 |
| Italy - CAAM 3353 | F | 40 | Amb a 1 | 0 | Cry j 1 | 2,7 | Cup a 1 | 3,11 |
| Italy - CAAM 3354 | M | 40 | Amb a 1 | 0 | Cry j 1 | 1,42 | Cup a 1 | 1,79 |
| Italy - CAAM 3355 | F | 40 | Amb a 1 | 0 | Cry j 1 | 22,11 | Cup a 1 | 9,64 |
| Italy - CAAM 3356 | F | 40 | Amb a 1 | 0 | Cry j 1 | 1,95 | Cup a 1 | 4,11 |
| Italy - CAAM 3357 | M | 40 | Amb a 1 | 0 | Cry j 1 | 0,15 | Cup a 1 | 0,34 |
| Italy - CAAM 3358 | F | 40 | Amb a 1 | 0 | Cry j 1 | 0,23 | Cup a 1 | 0,18 |
| Italy - CAAM 3359 | F | 40 | Amb a 1 | 0 | Cry j 1 | 13,37 | Cup a 1 | 3,9 |
| Italy - CAAM 3360 | M | 40 | Amb a 1 | 0 | Cry j 1 | 0,58 | Cup a 1 | 1,57 |
| Italy - CAAM 3361 | M | 40 | Amb a 1 | 0 | Cry j 1 | 6,49 | Cup a 1 | 18,5 |
| Italy - CAAM 3362 | M | 40 | Amb a 1 | 0 | Cry j 1 | 0,11 | Cup a 1 | 2,75 |
| Italy - CAAM 3363 | F | 40 | Amb a 1 | 0 | Cry j 1 | 0 | Cup a 1 | 0,74 |
| Italy - CAAM 3364 | M | 40 | Amb a 1 | 0 | Cry j 1 | 0,1 | Cup a 1 | 0,37 |
| Italy - CAAM 3365 | M | 40 | Amb a 1 | 0 | Cry j 1 | 13,67 | Cup a 1 | 18,33 |
| Italy - CAAM 3366 | M | 40 | Amb a 1 | 0 | Cry j 1 | 0 | Cup a 1 | 1,54 |
| Italy - CAAM 3367 | M | 40 | Amb a 1 | 0 | Cry j 1 | 5,35 | Cup a 1 | 16,09 |
| Italy - CAAM 3368 | M | 40 | Amb a 1 | 0 | Cry j 1 | 3,05 | Cup a 1 | 7,19 |
| Italy - CAAM 3369 | M | 40 | Amb a 1 | 0 | Cry j 1 | 0,99 | Cup a 1 | 2,49 |
| Italy - CAAM 3370 | F | 40 | Amb a 1 | 0 | Cry j 1 | 7,27 | Cup a 1 | 16,01 |
| Italy - CAAM 3371 | M | 40 | Amb a 1 | 0 | Cry j 1 | 0,03 | Cup a 1 | 0,23 |
| Italy - CAAM 3372 | F | 40 | Amb a 1 | 0 | Cry j 1 | 0 | Cup a 1 | 0,06 |
| Italy - CAAM 3373 | F | 40 | Amb a 1 | 0 | Cry j 1 | 0,12 | Cup a 1 | 0,13 |
| Italy - CAAM 3374 | M | 40 | Amb a 1 | 0 | Cry j 1 | 1,68 | Cup a 1 | 11,8 |
| Italy - CAAM 3375 | F | 40 | Amb a 1 | 0 | Cry j 1 | 3,44 | Cup a 1 | 3,71 |
| Italy - CAAM 3376 | M | 40 | Amb a 1 | 0 | Cry j 1 | 2,06 | Cup a 1 | 5,12 |
| Italy - CAAM 3377 | F | 40 | Amb a 1 | 0 | Cry j 1 | 0 | Cup a 1 | 0,71 |
| Italy - CAAM 3378 | M | 40 | Amb a 1 | 0 | Cry j 1 | 0,93 | Cup a 1 | 2,83 |
| Italy - CAAM 3379 | F | 40 | Amb a 1 | 0 | Cry j 1 | 0,67 | Cup a 1 | 0,42 |
| Italy - CAAM 3380 | F | 40 | Amb a 1 | 0 | Cry j 1 | 4,6 | Cup a 1 | 4,25 |
| Italy - CAAM 3381 | F | 40 | Amb a 1 | 0 | Cry j 1 | 3,72 | Cup a 1 | 19,75 |
| Italy - CAAM 3382 | F | 40 | Amb a 1 | 0 | Cry j 1 | 0 | Cup a 1 | 2,09 |
| Italy - CAAM 3383 | F | 40 | Amb a 1 | 0 | Cry j 1 | 5,93 | Cup a 1 | 16,83 |
| Italy - CAAM 3384 | F | 40 | Amb a 1 | 0 | Cry j 1 | 0,33 | Cup a 1 | 0,91 |
| Italy - CAAM 3385 | F | 40 | Amb a 1 | 0 | Cry j 1 | 9,84 | Cup a 1 | 38,63 |
| Italy - CAAM 3386 | M | 40 | Amb a 1 | 0 | Cry j 1 | 0 | Cup a 1 | 0,41 |
| Italy - CAAM 3387 | F | 40 | Amb a 1 | 0 | Cry j 1 | 0,13 | Cup a 1 | 4,65 |
| Italy - CAAM 3388 | M | 40 | Amb a 1 | 0 | Cry j 1 | 2,02 | Cup a 1 | 3,3 |
| Italy - CAAM 3389 | F | 40 | Amb a 1 | 0 | Cry j 1 | 0,36 | Cup a 1 | 1,68 |
| Italy - CAAM 3390 | F | 40 | Amb a 1 | 0 | Cry j 1 | 0,53 | Cup a 1 | 2,97 |
| Italy - CAAM 3391 | F | 40 | Amb a 1 | 0 | Cry j 1 | 0,54 | Cup a 1 | 3,57 |
| Italy - CAAM 3392 | F | 40 | Amb a 1 | 0 | Cry j 1 | 0 | Cup a 1 | 2,85 |
| Italy - CAAM 3393 | F | 40 | Amb a 1 | 0 | Cry j 1 | 3,84 | Cup a 1 | 67,94 |
| Italy - CAAM 3394 | F | 40 | Amb a 1 | 0 | Cry j 1 | 0 | Cup a 1 | 5,52 |
| Italy - CAAM 3395 | F | 40 | Amb a 1 | 0 | Cry j 1 | 0,83 | Cup a 1 | 5,13 |
| Italy - CAAM 3396 | F | 40 | Amb a 1 | 0 | Cry j 1 | 1,61 | Cup a 1 | 10,72 |
| Italy - CAAM 3397 | F | 40 | Amb a 1 | 0 | Cry j 1 | 15,39 | Cup a 1 | 27,19 |
| Italy - CAAM 3398 | M | 40 | Amb a 1 | 0 | Cry j 1 | 0 | Cup a 1 | 2,27 |
| Italy - CAAM 3399 | F | 40 | Amb a 1 | 0 | Cry j 1 | 0,08 | Cup a 1 | 0,82 |
| Italy - CAAM 3400 | F | 40 | Amb a 1 | 0 | Cry j 1 | 0,59 | Cup a 1 | 2,45 |
| Italy - CAAM 3401 | M | 40 | Amb a 1 | 0 | Cry j 1 | 1,05 | Cup a 1 | 3,27 |
| Italy - CAAM 3402 | F | 40 | Amb a 1 | 0 | Cry j 1 | 6,87 | Cup a 1 | 10,02 |
| Italy - CAAM 3403 | M | 40 | Amb a 1 | 0 | Cry j 1 | 0,49 | Cup a 1 | 5,73 |
| Italy - CAAM 3404 | M | 40 | Amb a 1 | 0 | Cry j 1 | 0,87 | Cup a 1 | 1,13 |
| Italy - CAAM 3405 | F | 40 | Amb a 1 | 0 | Cry j 1 | 5,47 | Cup a 1 | 9,09 |
| Italy - CAAM 3406 | F | 40 | Amb a 1 | 0 | Cry j 1 | 0 | Cup a 1 | 0,28 |
| Italy - CAAM 3407 | F | 40 | Amb a 1 | 0 | Cry j 1 | 0 | Cup a 1 | 0,61 |
| Italy - CAAM 3408 | M | 40 | Amb a 1 | 0 | Cry j 1 | 0,42 | Cup a 1 | 2,52 |
| Italy - CAAM 3409 | F | 40 | Amb a 1 | 0 | Cry j 1 | 0,04 | Cup a 1 | 0,82 |
| Italy - CAAM 3410 | F | 40 | Amb a 1 | 0 | Cry j 1 | 0,12 | Cup a 1 | 0,42 |
| Italy - CAAM 3411 | M | 40 | Amb a 1 | 0 | Cry j 1 | 1,3 | Cup a 1 | 5,54 |
| Italy - CAAM 3412 | F | 40 | Amb a 1 | 0 | Cry j 1 | 1,31 | Cup a 1 | 2,33 |
| Italy - CAAM 3413 | F | 40 | Amb a 1 | 0 | Cry j 1 | 1,57 | Cup a 1 | 8,53 |
| Italy - CAAM 3414 | M | 40 | Amb a 1 | 0 | Cry j 1 | 0,51 | Cup a 1 | 3,43 |
| Italy - CAAM 3415 | M | 40 | Amb a 1 | 0 | Cry j 1 | 1,67 | Cup a 1 | 3,71 |
| Italy - CAAM 3416 | M | 40 | Amb a 1 | 0 | Cry j 1 | 0,17 | Cup a 1 | 0 |
| Italy - CAAM 3417 | M | 40 | Amb a 1 | 0 | Cry j 1 | 0 | Cup a 1 | 0,04 |
| Italy - CAAM 3418 | F | 40 | Amb a 1 | 0 | Cry j 1 | 0,41 | Cup a 1 | 0,87 |
| Italy - CAAM 3419 | M | 40 | Amb a 1 | 0 | Cry j 1 | 1,01 | Cup a 1 | 2,03 |
| Italy - CAAM 3420 | F | 40 | Amb a 1 | 0 | Cry j 1 | 10,56 | Cup a 1 | 54,64 |
| Italy - CAAM 3421 | F | 40 | Amb a 1 | 0 | Cry j 1 | 5,51 | Cup a 1 | 9,46 |
| Italy - CAAM 3422 | M | 40 | Amb a 1 | 0 | Cry j 1 | 2,02 | Cup a 1 | 16,63 |
| Italy - CAAM 3423 | M | 40 | Amb a 1 | 0 | Cry j 1 | 0,52 | Cup a 1 | 2,71 |
| Italy - CAAM 3424 | M | 40 | Amb a 1 | 0 | Cry j 1 | 0,78 | Cup a 1 | 2,33 |
| Italy - CAAM 3425 | F | 40 | Amb a 1 | 0 | Cry j 1 | 0,16 | Cup a 1 | 2,99 |
| Italy - CAAM 3426 | M | 40 | Amb a 1 | 0 | Cry j 1 | 0 | Cup a 1 | 0,82 |
| Italy - CAAM 3427 | F | 40 | Amb a 1 | 0 | Cry j 1 | 0,87 | Cup a 1 | 3,88 |
| Italy - CAAM 3428 | M | 40 | Amb a 1 | 0 | Cry j 1 | 0,34 | Cup a 1 | 1,7 |
| Italy - CAAM 3429 | F | 40 | Amb a 1 | 0 | Cry j 1 | 7,09 | Cup a 1 | 13,39 |
| Italy - CAAM 3430 | F | 40 | Amb a 1 | 0 | Cry j 1 | 2,76 | Cup a 1 | 7,51 |
| Italy - CAAM 3431 | M | 40 | Amb a 1 | 0 | Cry j 1 | 1,15 | Cup a 1 | 2,01 |
| Italy - CAAM 3432 | M | 40 | Amb a 1 | 0 | Cry j 1 | 0 | Cup a 1 | 0,18 |
| Italy - CAAM 3433 | M | 40 | Amb a 1 | 0 | Cry j 1 | 33,59 | Cup a 1 | 48,46 |
| Italy - CAAM 3434 | F | 40 | Amb a 1 | 0 | Cry j 1 | 0,09 | Cup a 1 | 4,83 |
| Italy - CAAM 3435 | F | 40 | Amb a 1 | 0 | Cry j 1 | 4,54 | Cup a 1 | 29,77 |
| Italy - CAAM 3436 | F | 40 | Amb a 1 | 0 | Cry j 1 | 1,16 | Cup a 1 | 2,75 |
| Italy - CAAM 3437 | F | 40 | Amb a 1 | 0 | Cry j 1 | 3,07 | Cup a 1 | 9,14 |
| Italy - CAAM 3438 | M | 40 | Amb a 1 | 0 | Cry j 1 | 0,07 | Cup a 1 | 0,66 |
| Italy - CAAM 3439 | M | 40 | Amb a 1 | 0 | Cry j 1 | 4,98 | Cup a 1 | 18,47 |
| Italy - CAAM 3440 | F | 40 | Amb a 1 | 0 | Cry j 1 | 1,21 | Cup a 1 | 2,91 |
| Italy - CAAM 3441 | M | 40 | Amb a 1 | 0 | Cry j 1 | 0 | Cup a 1 | 0,12 |
| Italy - CAAM 3442 | M | 40 | Amb a 1 | 0 | Cry j 1 | 4,74 | Cup a 1 | 14,78 |
| Italy - CAAM 3443 | M | 40 | Amb a 1 | 0 | Cry j 1 | 1,37 | Cup a 1 | 8,77 |
| Italy - CAAM 3444 | M | 40 | Amb a 1 | 0 | Cry j 1 | 9,34 | Cup a 1 | 21,48 |
| Italy - CAAM 3445 | M | 40 | Amb a 1 | 0 | Cry j 1 | 2,28 | Cup a 1 | 16,05 |
| Italy - CAAM 3446 | F | 40 | Amb a 1 | 0 | Cry j 1 | 0,18 | Cup a 1 | 4,85 |
| Italy - CAAM 3447 | F | 40 | Amb a 1 | 0 | Cry j 1 | 2,97 | Cup a 1 | 23,98 |
| Italy - CAAM 3448 | F | 40 | Amb a 1 | 0 | Cry j 1 | 6,21 | Cup a 1 | 18,52 |
| Italy - CAAM 3449 | M | 40 | Amb a 1 | 0 | Cry j 1 | 2,5 | Cup a 1 | 4,98 |
| Italy - CAAM 3450 | F | 40 | Amb a 1 | 0 | Cry j 1 | 13,34 | Cup a 1 | 45,6 |
| Italy - CAAM 3451 | M | 40 | Amb a 1 | 0 | Cry j 1 | 0,12 | Cup a 1 | 4,89 |
| Italy - CAAM 3452 | M | 40 | Amb a 1 | 0 | Cry j 1 | 0 | Cup a 1 | 0,77 |
| Italy - CAAM 3453 | F | 40 | Amb a 1 | 0 | Cry j 1 | 3,97 | Cup a 1 | 9,3 |
| Italy - CAAM 3454 | F | 40 | Amb a 1 | 0 | Cry j 1 | 0 | Cup a 1 | 1,61 |
| Italy - CAAM 3455 | F | 40 | Amb a 1 | 0 | Cry j 1 | 1,61 | Cup a 1 | 12,19 |
| Italy - CAAM 3456 | F | 40 | Amb a 1 | 0 | Cry j 1 | 0,07 | Cup a 1 | 5,52 |
| Italy - CAAM 3457 | F | 40 | Amb a 1 | 0 | Cry j 1 | 0,37 | Cup a 1 | 11,26 |
| Italy - CAAM 3458 | M | 40 | Amb a 1 | 0 | Cry j 1 | 0,47 | Cup a 1 | 1,3 |
| Italy - CAAM 3459 | F | 40 | Amb a 1 | 0 | Cry j 1 | 0,3 | Cup a 1 | 4,26 |
| Italy - CAAM 3460 | M | 40 | Amb a 1 | 0 | Cry j 1 | 0 | Cup a 1 | 0,25 |
| Italy - CAAM 3461 | F | 40 | Amb a 1 | 0 | Cry j 1 | 1,92 | Cup a 1 | 18,92 |
| Italy - CAAM 3462 | F | 40 | Amb a 1 | 0 | Cry j 1 | 0,28 | Cup a 1 | 1,89 |
| Italy - CAAM 3463 | M | 40 | Amb a 1 | 0 | Cry j 1 | 0 | Cup a 1 | 2,98 |
| Italy - CAAM 3464 | F | 40 | Amb a 1 | 0 | Cry j 1 | 0 | Cup a 1 | 0,27 |
| Italy - CAAM 3465 | F | 40 | Amb a 1 | 0 | Cry j 1 | 0,62 | Cup a 1 | 13,1 |
| Italy - CAAM 3466 | M | 40 | Amb a 1 | 0 | Cry j 1 | 0,1 | Cup a 1 | 3,39 |
| Italy - CAAM 3467 | F | 40 | Amb a 1 | 0 | Cry j 1 | 0,07 | Cup a 1 | 1,69 |
| Italy - CAAM 3468 | M | 40 | Amb a 1 | 0 | Cry j 1 | 1,3 | Cup a 1 | 7,9 |
| Italy - CAAM 3469 | F | 40 | Amb a 1 | 0 | Cry j 1 | 3,19 | Cup a 1 | 13,49 |
| Italy - CAAM 3470 | F | 40 | Amb a 1 | 0 | Cry j 1 | 0,46 | Cup a 1 | 5,63 |
| Italy - CAAM 3471 | F | 40 | Amb a 1 | 0 | Cry j 1 | 0 | Cup a 1 | 2,06 |
| Italy - CAAM 3472 | F | 40 | Amb a 1 | 0 | Cry j 1 | 0 | Cup a 1 | 0,61 |
| Italy - CAAM 3473 | F | 40 | Amb a 1 | 0 | Cry j 1 | 0 | Cup a 1 | 0,3 |
| Italy - CAAM 3474 | F | 40 | Amb a 1 | 0 | Cry j 1 | 3,84 | Cup a 1 | 35,59 |
| Italy - CAAM 3475 | F | 40 | Amb a 1 | 0 | Cry j 1 | 0 | Cup a 1 | 15,49 |
| Italy - CAAM 3476 | F | 40 | Amb a 1 | 0 | Cry j 1 | 1,73 | Cup a 1 | 8,58 |
| Italy - CAAM 3477 | F | 40 | Amb a 1 | 0 | Cry j 1 | 1,21 | Cup a 1 | 9,39 |
| Italy - CAAM 3478 | F | 40 | Amb a 1 | 0 | Cry j 1 | 0 | Cup a 1 | 1,07 |
| Italy - CAAM 3479 | M | 40 | Amb a 1 | 0 | Cry j 1 | 2,42 | Cup a 1 | 14,01 |
| Italy - CAAM 3480 | F | 40 | Amb a 1 | 0 | Cry j 1 | 0,46 | Cup a 1 | 8,67 |
| Italy - CAAM 3481 | M | 40 | Amb a 1 | 0 | Cry j 1 | 0,3 | Cup a 1 | 4,82 |
| Italy - CAAM 3482 | F | 40 | Amb a 1 | 0 | Cry j 1 | 0,44 | Cup a 1 | 8,63 |
| Italy - CAAM 3483 | F | 40 | Amb a 1 | 0 | Cry j 1 | 0,25 | Cup a 1 | 2,23 |
| Italy - CAAM 3484 | F | 40 | Amb a 1 | 0 | Cry j 1 | 1,22 | Cup a 1 | 5,13 |
| Italy - CAAM 3485 | M | 40 | Amb a 1 | 0 | Cry j 1 | 0,51 | Cup a 1 | 9,06 |
| Italy - CAAM 3486 | F | 40 | Amb a 1 | 0 | Cry j 1 | 0,7 | Cup a 1 | 17 |
| Italy - CAAM 3487 | F | 40 | Amb a 1 | 0 | Cry j 1 | 1,3 | Cup a 1 | 13 |
| Italy - CAAM 3488 | M | 40 | Amb a 1 | 0 | Cry j 1 | 0 | Cup a 1 | 7,5 |
| Italy - CAAM 3489 | M | 40 | Amb a 1 | 0 | Cry j 1 | 0,6 | Cup a 1 | 10 |
| Italy - CAAM 3490 | F | 40 | Amb a 1 | 0 | Cry j 1 | 0,08 | Cup a 1 | 3,95 |
| Italy - CAAM 3491 | F | 40 | Amb a 1 | 0 | Cry j 1 | 0,09 | Cup a 1 | 9,8 |
| Italy - CAAM 3492 | F | 40 | Amb a 1 | 0 | Cry j 1 | 0,28 | Cup a 1 | 8,03 |
| Italy - CAAM 3493 | F | 40 | Amb a 1 | 0 | Cry j 1 | 0,09 | Cup a 1 | 14,83 |
| Italy - CAAM 3494 | F | 40 | Amb a 1 | 0 | Cry j 1 | 0 | Cup a 1 | 2,32 |
| Italy - CAAM 3495 | F | 40 | Amb a 1 | 0 | Cry j 1 | 0,18 | Cup a 1 | 4,54 |
| Italy - CAAM 3496 | F | 40 | Amb a 1 | 0 | Cry j 1 | 0,46 | Cup a 1 | 7,84 |
| Italy - CAAM 3497 | F | 39 | Amb a 1 | 0 | Cry j 1 | 0,11 | Cup a 1 | 1,89 |
| Italy - CAAM 3498 | F | 39 | Amb a 1 | 0 | Cry j 1 | 0 | Cup a 1 | 3,2 |
| Italy - CAAM 3499 | M | 39 | Amb a 1 | 0 | Cry j 1 | 6,37 | Cup a 1 | 5,85 |
| Italy - CAAM 3500 | F | 39 | Amb a 1 | 0 | Cry j 1 | 0 | Cup a 1 | 2,79 |
| Italy - CAAM 3501 | F | 39 | Amb a 1 | 0 | Cry j 1 | 0 | Cup a 1 | 1,43 |
| Italy - CAAM 3502 | F | 39 | Amb a 1 | 0 | Cry j 1 | 0,86 | Cup a 1 | 0 |
| Italy - CAAM 3503 | F | 39 | Amb a 1 | 0 | Cry j 1 | 0 | Cup a 1 | 2,43 |
| Italy - CAAM 3504 | F | 39 | Amb a 1 | 0 | Cry j 1 | 0 | Cup a 1 | 0,91 |
| Italy - CAAM 3505 | F | 39 | Amb a 1 | 0 | Cry j 1 | 2,79 | Cup a 1 | 8,59 |
| Italy - CAAM 3506 | M | 39 | Amb a 1 | 0 | Cry j 1 | 0 | Cup a 1 | 3,12 |
| Italy - CAAM 3507 | M | 39 | Amb a 1 | 0 | Cry j 1 | 5,51 | Cup a 1 | 6,3 |
| Italy - CAAM 3508 | F | 39 | Amb a 1 | 0 | Cry j 1 | 9,29 | Cup a 1 | 31,67 |
| Italy - CAAM 3509 | F | 39 | Amb a 1 | 0 | Cry j 1 | 7,81 | Cup a 1 | 26,67 |
| Italy - CAAM 3510 | F | 39 | Amb a 1 | 0 | Cry j 1 | 1,78 | Cup a 1 | 2,88 |
| Italy - CAAM 3511 | F | 39 | Amb a 1 | 0 | Cry j 1 | 1,21 | Cup a 1 | 3,46 |
| Italy - CAAM 3512 | F | 39 | Amb a 1 | 0 | Cry j 1 | 2,88 | Cup a 1 | 6,74 |
| Italy - CAAM 3513 | M | 39 | Amb a 1 | 0 | Cry j 1 | 1,4 | Cup a 1 | 0 |
| Italy - CAAM 3514 | F | 39 | Amb a 1 | 0 | Cry j 1 | 0 | Cup a 1 | 1,12 |
| Italy - CAAM 3515 | F | 39 | Amb a 1 | 0 | Cry j 1 | 0 | Cup a 1 | 3,42 |
| Italy - CAAM 3516 | F | 39 | Amb a 1 | 0 | Cry j 1 | 0 | Cup a 1 | 4,5 |
| Italy - CAAM 3517 | M | 39 | Amb a 1 | 0 | Cry j 1 | 4,78 | Cup a 1 | 9,15 |
| Italy - CAAM 3518 | M | 39 | Amb a 1 | 0 | Cry j 1 | 0,44 | Cup a 1 | 0,66 |
| Italy - CAAM 3519 | F | 39 | Amb a 1 | 0 | Cry j 1 | 0,23 | Cup a 1 | 1,05 |
| Italy - CAAM 3520 | F | 39 | Amb a 1 | 0 | Cry j 1 | 5,94 | Cup a 1 | 11,36 |
| Italy - CAAM 3521 | F | 39 | Amb a 1 | 0 | Cry j 1 | 2,16 | Cup a 1 | 2,49 |
| Italy - CAAM 3522 | F | 39 | Amb a 1 | 0 | Cry j 1 | 0,44 | Cup a 1 | 2,6 |
| Italy - CAAM 3523 | M | 39 | Amb a 1 | 0 | Cry j 1 | 2,42 | Cup a 1 | 6,22 |
| Italy - CAAM 3524 | F | 39 | Amb a 1 | 0 | Cry j 1 | 2,04 | Cup a 1 | 2,1 |
| Italy - CAAM 3525 | M | 39 | Amb a 1 | 0 | Cry j 1 | 1,21 | Cup a 1 | 6,42 |
| Italy - CAAM 3526 | M | 39 | Amb a 1 | 0 | Cry j 1 | 0,39 | Cup a 1 | 0,35 |
| Italy - CAAM 3527 | M | 39 | Amb a 1 | 0 | Cry j 1 | 0,51 | Cup a 1 | 4,85 |
| Italy - CAAM 3528 | M | 39 | Amb a 1 | 0 | Cry j 1 | 0 | Cup a 1 | 6,89 |
| Italy - CAAM 3529 | F | 39 | Amb a 1 | 0 | Cry j 1 | 3,23 | Cup a 1 | 7,64 |
| Italy - CAAM 3530 | F | 39 | Amb a 1 | 0 | Cry j 1 | 0,27 | Cup a 1 | 2,55 |
| Italy - CAAM 3531 | M | 39 | Amb a 1 | 0 | Cry j 1 | 1,3 | Cup a 1 | 2,21 |
| Italy - CAAM 3532 | F | 39 | Amb a 1 | 0 | Cry j 1 | 0 | Cup a 1 | 0,41 |
| Italy - CAAM 3533 | F | 39 | Amb a 1 | 0 | Cry j 1 | 1,75 | Cup a 1 | 4,35 |
| Italy - CAAM 3534 | M | 39 | Amb a 1 | 0 | Cry j 1 | 4,79 | Cup a 1 | 9,87 |
| Italy - CAAM 3535 | M | 39 | Amb a 1 | 0 | Cry j 1 | 8,4 | Cup a 1 | 8,27 |
| Italy - CAAM 3536 | M | 39 | Amb a 1 | 0 | Cry j 1 | 0,28 | Cup a 1 | 0,57 |
| Italy - CAAM 3537 | M | 39 | Amb a 1 | 0 | Cry j 1 | 1,42 | Cup a 1 | 1,65 |
| Italy - CAAM 3538 | F | 39 | Amb a 1 | 0 | Cry j 1 | 1,35 | Cup a 1 | 11,15 |
| Italy - CAAM 3539 | M | 39 | Amb a 1 | 0 | Cry j 1 | 0,46 | Cup a 1 | 2,22 |
| Italy - CAAM 3540 | M | 39 | Amb a 1 | 0 | Cry j 1 | 26,25 | Cup a 1 | 15,89 |
| Italy - CAAM 3541 | M | 39 | Amb a 1 | 0 | Cry j 1 | 0 | Cup a 1 | 1,02 |
| Italy - CAAM 3542 | F | 39 | Amb a 1 | 0 | Cry j 1 | 1,1 | Cup a 1 | 0,88 |
| Italy - CAAM 3543 | F | 39 | Amb a 1 | 0 | Cry j 1 | 0,9 | Cup a 1 | 7,8 |
| Italy - CAAM 3544 | F | 39 | Amb a 1 | 0 | Cry j 1 | 0,96 | Cup a 1 | 3,68 |
| Italy - CAAM 3545 | F | 39 | Amb a 1 | 0 | Cry j 1 | 0 | Cup a 1 | 0,87 |
| Italy - CAAM 3546 | F | 39 | Amb a 1 | 0 | Cry j 1 | 2,48 | Cup a 1 | 2,8 |
| Italy - CAAM 3547 | F | 39 | Amb a 1 | 0 | Cry j 1 | 0,68 | Cup a 1 | 5,7 |
| Italy - CAAM 3548 | M | 39 | Amb a 1 | 0 | Cry j 1 | 0,66 | Cup a 1 | 2,55 |
| Italy - CAAM 3549 | F | 39 | Amb a 1 | 0 | Cry j 1 | 7,81 | Cup a 1 | 18,05 |
| Italy - CAAM 3550 | M | 39 | Amb a 1 | 0 | Cry j 1 | 0,17 | Cup a 1 | 0,21 |
| Italy - CAAM 3551 | F | 39 | Amb a 1 | 0 | Cry j 1 | 0 | Cup a 1 | 0,3 |
| Italy - CAAM 3552 | F | 39 | Amb a 1 | 0 | Cry j 1 | 0,07 | Cup a 1 | 1,39 |
| Italy - CAAM 3553 | M | 39 | Amb a 1 | 0 | Cry j 1 | 0,57 | Cup a 1 | 2,35 |
| Italy - CAAM 3554 | F | 39 | Amb a 1 | 0 | Cry j 1 | 0,34 | Cup a 1 | 4,91 |
| Italy - CAAM 3555 | F | 39 | Amb a 1 | 0 | Cry j 1 | 6,43 | Cup a 1 | 1,31 |
| Italy - CAAM 3556 | F | 39 | Amb a 1 | 0 | Cry j 1 | 1,02 | Cup a 1 | 2,74 |
| Italy - CAAM 3557 | F | 39 | Amb a 1 | 0 | Cry j 1 | 2,22 | Cup a 1 | 9,77 |
| Italy - CAAM 3558 | F | 39 | Amb a 1 | 0 | Cry j 1 | 2,5 | Cup a 1 | 9,24 |
| Italy - CAAM 3559 | F | 39 | Amb a 1 | 0 | Cry j 1 | 3,1 | Cup a 1 | 7,19 |
| Italy - CAAM 3560 | F | 39 | Amb a 1 | 0 | Cry j 1 | 1,85 | Cup a 1 | 4,02 |
| Italy - CAAM 3561 | F | 39 | Amb a 1 | 0 | Cry j 1 | 0,03 | Cup a 1 | 0,25 |
| Italy - CAAM 3562 | F | 39 | Amb a 1 | 0 | Cry j 1 | 0,03 | Cup a 1 | 0,4 |
| Italy - CAAM 3563 | F | 39 | Amb a 1 | 0 | Cry j 1 | 0 | Cup a 1 | 0,27 |
| Italy - CAAM 3564 | F | 39 | Amb a 1 | 0 | Cry j 1 | 1,61 | Cup a 1 | 6,26 |
| Italy - CAAM 3565 | M | 39 | Amb a 1 | 0 | Cry j 1 | 0 | Cup a 1 | 0,07 |
| Italy - CAAM 3566 | M | 39 | Amb a 1 | 0 | Cry j 1 | 0,31 | Cup a 1 | 1,3 |
| Italy - CAAM 3567 | M | 39 | Amb a 1 | 0 | Cry j 1 | 9,48 | Cup a 1 | 27,78 |
| Italy - CAAM 3568 | M | 39 | Amb a 1 | 0 | Cry j 1 | 11,47 | Cup a 1 | 30,55 |
| Italy - CAAM 3569 | F | 39 | Amb a 1 | 0 | Cry j 1 | 0,2 | Cup a 1 | 4,45 |
| Italy - CAAM 3570 | M | 39 | Amb a 1 | 0 | Cry j 1 | 0,05 | Cup a 1 | 0,31 |
| Italy - CAAM 3571 | F | 39 | Amb a 1 | 0 | Cry j 1 | 0,13 | Cup a 1 | 1,09 |
| Italy - CAAM 3572 | M | 39 | Amb a 1 | 0 | Cry j 1 | 0,46 | Cup a 1 | 1,06 |
| Italy - CAAM 3573 | F | 39 | Amb a 1 | 0 | Cry j 1 | 1,68 | Cup a 1 | 3,3 |
| Italy - CAAM 3574 | F | 39 | Amb a 1 | 0 | Cry j 1 | 0 | Cup a 1 | 0,84 |
| Italy - CAAM 3575 | F | 39 | Amb a 1 | 0 | Cry j 1 | 5,39 | Cup a 1 | 14,11 |
| Italy - CAAM 3576 | F | 39 | Amb a 1 | 0 | Cry j 1 | 0 | Cup a 1 | 1,19 |
| Italy - CAAM 3577 | M | 39 | Amb a 1 | 0 | Cry j 1 | 1 | Cup a 1 | 13,58 |
| Italy - CAAM 3578 | F | 39 | Amb a 1 | 0 | Cry j 1 | 12,32 | Cup a 1 | 10,94 |
| Italy - CAAM 3579 | M | 39 | Amb a 1 | 0 | Cry j 1 | 0,67 | Cup a 1 | 6,01 |
| Italy - CAAM 3580 | M | 39 | Amb a 1 | 0 | Cry j 1 | 0,75 | Cup a 1 | 8,54 |
| Italy - CAAM 3581 | F | 39 | Amb a 1 | 0 | Cry j 1 | 0 | Cup a 1 | 1,05 |
| Italy - CAAM 3582 | M | 39 | Amb a 1 | 0 | Cry j 1 | 0 | Cup a 1 | 0,14 |
| Italy - CAAM 3583 | M | 39 | Amb a 1 | 0 | Cry j 1 | 0 | Cup a 1 | 0,47 |
| Italy - CAAM 3584 | F | 39 | Amb a 1 | 0 | Cry j 1 | 3,85 | Cup a 1 | 5,14 |
| Italy - CAAM 3585 | M | 39 | Amb a 1 | 0 | Cry j 1 | 1,63 | Cup a 1 | 5,33 |
| Italy - CAAM 3586 | F | 39 | Amb a 1 | 0 | Cry j 1 | 1,28 | Cup a 1 | 19,36 |
| Italy - CAAM 3587 | F | 39 | Amb a 1 | 0 | Cry j 1 | 0,03 | Cup a 1 | 3,17 |
| Italy - CAAM 3588 | M | 39 | Amb a 1 | 0 | Cry j 1 | 8,14 | Cup a 1 | 17,61 |
| Italy - CAAM 3589 | M | 39 | Amb a 1 | 0 | Cry j 1 | 0 | Cup a 1 | 1,52 |
| Italy - CAAM 3590 | F | 39 | Amb a 1 | 0 | Cry j 1 | 0,09 | Cup a 1 | 0 |
| Italy - CAAM 3591 | M | 39 | Amb a 1 | 0 | Cry j 1 | 0 | Cup a 1 | 0,3 |
| Italy - CAAM 3592 | F | 39 | Amb a 1 | 0 | Cry j 1 | 0,17 | Cup a 1 | 4,08 |
| Italy - CAAM 3593 | F | 39 | Amb a 1 | 0 | Cry j 1 | 0 | Cup a 1 | 1,42 |
| Italy - CAAM 3594 | F | 39 | Amb a 1 | 0 | Cry j 1 | 0,63 | Cup a 1 | 10,12 |
| Italy - CAAM 3595 | M | 39 | Amb a 1 | 0 | Cry j 1 | 0 | Cup a 1 | 0,07 |
| Italy - CAAM 3596 | M | 39 | Amb a 1 | 0 | Cry j 1 | 0,12 | Cup a 1 | 0 |
| Italy - CAAM 3597 | F | 39 | Amb a 1 | 0 | Cry j 1 | 2,57 | Cup a 1 | 5,62 |
| Italy - CAAM 3598 | F | 39 | Amb a 1 | 0 | Cry j 1 | 0 | Cup a 1 | 3,04 |
| Italy - CAAM 3599 | F | 39 | Amb a 1 | 0 | Cry j 1 | 6,92 | Cup a 1 | 20,35 |
| Italy - CAAM 3600 | M | 39 | Amb a 1 | 0 | Cry j 1 | 0,97 | Cup a 1 | 2,09 |
| Italy - CAAM 3601 | F | 39 | Amb a 1 | 0 | Cry j 1 | 6,78 | Cup a 1 | 12,53 |
| Italy - CAAM 3602 | M | 39 | Amb a 1 | 0 | Cry j 1 | 0,15 | Cup a 1 | 0,6 |
| Italy - CAAM 3603 | F | 39 | Amb a 1 | 0 | Cry j 1 | 0 | Cup a 1 | 0,23 |
| Italy - CAAM 3604 | F | 39 | Amb a 1 | 0 | Cry j 1 | 0,09 | Cup a 1 | 1,15 |
| Italy - CAAM 3605 | F | 39 | Amb a 1 | 0 | Cry j 1 | 4,75 | Cup a 1 | 19,45 |
| Italy - CAAM 3606 | M | 39 | Amb a 1 | 0 | Cry j 1 | 0 | Cup a 1 | 0,79 |
| Italy - CAAM 3607 | F | 39 | Amb a 1 | 0 | Cry j 1 | 2,06 | Cup a 1 | 11,27 |
| Italy - CAAM 3608 | F | 39 | Amb a 1 | 0 | Cry j 1 | 6,62 | Cup a 1 | 26,35 |
| Italy - CAAM 3609 | M | 39 | Amb a 1 | 0 | Cry j 1 | 0 | Cup a 1 | 4,3 |
| Italy - CAAM 3610 | M | 39 | Amb a 1 | 0 | Cry j 1 | 1,63 | Cup a 1 | 3,62 |
| Italy - CAAM 3611 | F | 39 | Amb a 1 | 0 | Cry j 1 | 0 | Cup a 1 | 0,03 |
| Italy - CAAM 3612 | M | 39 | Amb a 1 | 0 | Cry j 1 | 0,18 | Cup a 1 | 1,21 |
| Italy - CAAM 3613 | F | 39 | Amb a 1 | 0 | Cry j 1 | 2,73 | Cup a 1 | 6,25 |
| Italy - CAAM 3614 | M | 39 | Amb a 1 | 0 | Cry j 1 | 1,48 | Cup a 1 | 13,43 |
| Italy - CAAM 3615 | F | 39 | Amb a 1 | 0 | Cry j 1 | 0,03 | Cup a 1 | 1,03 |
| Italy - CAAM 3616 | M | 39 | Amb a 1 | 0 | Cry j 1 | 0,86 | Cup a 1 | 3,24 |
| Italy - CAAM 3617 | F | 39 | Amb a 1 | 0 | Cry j 1 | 0,9 | Cup a 1 | 12,94 |
| Italy - CAAM 3618 | M | 39 | Amb a 1 | 0 | Cry j 1 | 0 | Cup a 1 | 0,5 |
| Italy - CAAM 3619 | M | 39 | Amb a 1 | 0 | Cry j 1 | 0,93 | Cup a 1 | 12,81 |
| Italy - CAAM 3620 | F | 39 | Amb a 1 | 0 | Cry j 1 | 0,44 | Cup a 1 | 8,82 |
| Italy - CAAM 3621 | M | 39 | Amb a 1 | 0 | Cry j 1 | 0 | Cup a 1 | 0,1 |
| Italy - CAAM 3622 | M | 39 | Amb a 1 | 0 | Cry j 1 | 1,61 | Cup a 1 | 10,4 |
| Italy - CAAM 3623 | M | 39 | Amb a 1 | 0 | Cry j 1 | 4,25 | Cup a 1 | 13,67 |
| Italy - CAAM 3624 | M | 39 | Amb a 1 | 0 | Cry j 1 | 1,27 | Cup a 1 | 3,31 |
| Italy - CAAM 3625 | F | 39 | Amb a 1 | 0 | Cry j 1 | 1,04 | Cup a 1 | 8,25 |
| Italy - CAAM 3626 | M | 39 | Amb a 1 | 0 | Cry j 1 | 18,98 | Cup a 1 | 38 |
| Italy - CAAM 3627 | F | 39 | Amb a 1 | 0 | Cry j 1 | 0,24 | Cup a 1 | 8,1 |
| Italy - CAAM 3628 | F | 39 | Amb a 1 | 0 | Cry j 1 | 13,11 | Cup a 1 | 22,89 |
| Italy - CAAM 3629 | F | 39 | Amb a 1 | 0 | Cry j 1 | 0,47 | Cup a 1 | 5,5 |
| Italy - CAAM 3630 | M | 39 | Amb a 1 | 0 | Cry j 1 | 0,23 | Cup a 1 | 1,45 |
| Italy - CAAM 3631 | F | 39 | Amb a 1 | 0 | Cry j 1 | 0,05 | Cup a 1 | 1,77 |
| Italy - CAAM 3632 | F | 39 | Amb a 1 | 0 | Cry j 1 | 2,54 | Cup a 1 | 20,8 |
| Italy - CAAM 3633 | F | 39 | Amb a 1 | 0 | Cry j 1 | 1,82 | Cup a 1 | 5,16 |
| Italy - CAAM 3634 | F | 39 | Amb a 1 | 0 | Cry j 1 | 18,96 | Cup a 1 | 38,42 |
| Italy - CAAM 3635 | M | 39 | Amb a 1 | 0 | Cry j 1 | 3,6 | Cup a 1 | 12,26 |
| Italy - CAAM 3636 | F | 39 | Amb a 1 | 0 | Cry j 1 | 8,71 | Cup a 1 | 26,98 |
| Italy - CAAM 3637 | F | 39 | Amb a 1 | 0 | Cry j 1 | 0,96 | Cup a 1 | 6,64 |
| Italy - CAAM 3638 | M | 39 | Amb a 1 | 0 | Cry j 1 | 0 | Cup a 1 | 0,04 |
| Italy - CAAM 3639 | M | 39 | Amb a 1 | 0 | Cry j 1 | 0,66 | Cup a 1 | 17,42 |
| Italy - CAAM 3640 | F | 39 | Amb a 1 | 0 | Cry j 1 | 0 | Cup a 1 | 2,75 |
| Italy - CAAM 3641 | M | 39 | Amb a 1 | 0 | Cry j 1 | 0 | Cup a 1 | 1,09 |
| Italy - CAAM 3642 | M | 39 | Amb a 1 | 0 | Cry j 1 | 0,4 | Cup a 1 | 8,14 |
| Italy - CAAM 3643 | M | 39 | Amb a 1 | 0 | Cry j 1 | 0,55 | Cup a 1 | 2,5 |
| Italy - CAAM 3644 | F | 39 | Amb a 1 | 0 | Cry j 1 | 4,14 | Cup a 1 | 24,75 |
| Italy - CAAM 3645 | F | 39 | Amb a 1 | 0 | Cry j 1 | 0,22 | Cup a 1 | 5,42 |
| Italy - CAAM 3646 | M | 39 | Amb a 1 | 0 | Cry j 1 | 0 | Cup a 1 | 0,32 |
| Italy - CAAM 3647 | F | 39 | Amb a 1 | 0 | Cry j 1 | 0 | Cup a 1 | 6,87 |
| Italy - CAAM 3648 | F | 39 | Amb a 1 | 0 | Cry j 1 | 0 | Cup a 1 | 0,74 |
| Italy - CAAM 3649 | F | 39 | Amb a 1 | 0 | Cry j 1 | 0,37 | Cup a 1 | 1,48 |
| Italy - CAAM 3650 | F | 39 | Amb a 1 | 0 | Cry j 1 | 0 | Cup a 1 | 0,14 |
| Italy - CAAM 3651 | F | 39 | Amb a 1 | 0 | Cry j 1 | 0 | Cup a 1 | 0,42 |
| Italy - CAAM 3652 | F | 39 | Amb a 1 | 0 | Cry j 1 | 0,31 | Cup a 1 | 2,36 |
| Italy - CAAM 3653 | M | 39 | Amb a 1 | 0 | Cry j 1 | 1,96 | Cup a 1 | 9,43 |
| Italy - CAAM 3654 | M | 39 | Amb a 1 | 0 | Cry j 1 | 0 | Cup a 1 | 2,2 |
| Italy - CAAM 3655 | F | 39 | Amb a 1 | 0 | Cry j 1 | 50,8 | Cup a 1 | 44,99 |
| Italy - CAAM 3656 | F | 39 | Amb a 1 | 0 | Cry j 1 | 0 | Cup a 1 | 2,1 |
| Italy - CAAM 3657 | M | 39 | Amb a 1 | 0 | Cry j 1 | 1,95 | Cup a 1 | 10,95 |
| Italy - CAAM 3658 | F | 39 | Amb a 1 | 0 | Cry j 1 | 0 | Cup a 1 | 2,86 |
| Italy - CAAM 3659 | M | 39 | Amb a 1 | 0 | Cry j 1 | 4,42 | Cup a 1 | 13,14 |
| Italy - CAAM 3660 | M | 39 | Amb a 1 | 0 | Cry j 1 | 0,57 | Cup a 1 | 4,46 |
| Italy - CAAM 3661 | F | 39 | Amb a 1 | 0 | Cry j 1 | 5,33 | Cup a 1 | 27,23 |
| Italy - CAAM 3662 | F | 39 | Amb a 1 | 0 | Cry j 1 | 1,97 | Cup a 1 | 7,16 |
| Italy - CAAM 3663 | M | 39 | Amb a 1 | 0 | Cry j 1 | 0 | Cup a 1 | 0,17 |
| Italy - CAAM 3664 | F | 39 | Amb a 1 | 0 | Cry j 1 | 0,2 | Cup a 1 | 2,68 |
| Italy - CAAM 3665 | M | 39 | Amb a 1 | 0 | Cry j 1 | 0 | Cup a 1 | 0,12 |
| Italy - CAAM 3666 | F | 39 | Amb a 1 | 0 | Cry j 1 | 0,26 | Cup a 1 | 3,35 |
| Italy - CAAM 3667 | F | 39 | Amb a 1 | 0 | Cry j 1 | 0 | Cup a 1 | 1,4 |
| Italy - CAAM 3668 | M | 39 | Amb a 1 | 0 | Cry j 1 | 1 | Cup a 1 | 15 |
| Italy - CAAM 3669 | M | 39 | Amb a 1 | 0 | Cry j 1 | 0,6 | Cup a 1 | 4,5 |
| Italy - CAAM 3670 | F | 39 | Amb a 1 | 0 | Cry j 1 | 0 | Cup a 1 | 0,9 |
| Italy - CAAM 3671 | M | 39 | Amb a 1 | 0 | Cry j 1 | 8 | Cup a 1 | 7,2 |
| Italy - CAAM 3672 | F | 38 | Amb a 1 | 0 | Cry j 1 | 0 | Cup a 1 | 0,29 |
| Italy - CAAM 3673 | M | 38 | Amb a 1 | 0 | Cry j 1 | 0,29 | Cup a 1 | 1,48 |
| Italy - CAAM 3674 | M | 38 | Amb a 1 | 0 | Cry j 1 | 0 | Cup a 1 | 8,4 |
| Italy - CAAM 3675 | F | 38 | Amb a 1 | 0 | Cry j 1 | 4,93 | Cup a 1 | 8,16 |
| Italy - CAAM 3676 | M | 38 | Amb a 1 | 0 | Cry j 1 | 1,5 | Cup a 1 | 4,01 |
| Italy - CAAM 3677 | M | 38 | Amb a 1 | 0 | Cry j 1 | 0,19 | Cup a 1 | 2,72 |
| Italy - CAAM 3678 | F | 38 | Amb a 1 | 0 | Cry j 1 | 2,48 | Cup a 1 | 1,05 |
| Italy - CAAM 3679 | F | 38 | Amb a 1 | 0 | Cry j 1 | 16,92 | Cup a 1 | 42,04 |
| Italy - CAAM 3680 | F | 38 | Amb a 1 | 0 | Cry j 1 | 0,39 | Cup a 1 | 1,16 |
| Italy - CAAM 3681 | F | 38 | Amb a 1 | 0 | Cry j 1 | 0 | Cup a 1 | 0,3 |
| Italy - CAAM 3682 | F | 38 | Amb a 1 | 0 | Cry j 1 | 0,66 | Cup a 1 | 2,58 |
| Italy - CAAM 3683 | F | 38 | Amb a 1 | 0 | Cry j 1 | 1,65 | Cup a 1 | 5,01 |
| Italy - CAAM 3684 | M | 38 | Amb a 1 | 0 | Cry j 1 | 0 | Cup a 1 | 2,14 |
| Italy - CAAM 3685 | M | 38 | Amb a 1 | 0 | Cry j 1 | 0 | Cup a 1 | 2,06 |
| Italy - CAAM 3686 | F | 38 | Amb a 1 | 0 | Cry j 1 | 0 | Cup a 1 | 4,9 |
| Italy - CAAM 3687 | F | 38 | Amb a 1 | 0 | Cry j 1 | 6,21 | Cup a 1 | 11,83 |
| Italy - CAAM 3688 | M | 38 | Amb a 1 | 0 | Cry j 1 | 0 | Cup a 1 | 1,64 |
| Italy - CAAM 3689 | F | 38 | Amb a 1 | 0 | Cry j 1 | 7,91 | Cup a 1 | 11,31 |
| Italy - CAAM 3690 | M | 38 | Amb a 1 | 0 | Cry j 1 | 0,11 | Cup a 1 | 0,52 |
| Italy - CAAM 3691 | F | 38 | Amb a 1 | 0 | Cry j 1 | 2,76 | Cup a 1 | 14,92 |
| Italy - CAAM 3692 | M | 38 | Amb a 1 | 0 | Cry j 1 | 1,35 | Cup a 1 | 11,08 |
| Italy - CAAM 3693 | F | 38 | Amb a 1 | 0 | Cry j 1 | 0 | Cup a 1 | 0,88 |
| Italy - CAAM 3694 | F | 38 | Amb a 1 | 0 | Cry j 1 | 0 | Cup a 1 | 1,47 |
| Italy - CAAM 3695 | M | 38 | Amb a 1 | 0 | Cry j 1 | 0 | Cup a 1 | 0,75 |
| Italy - CAAM 3696 | F | 38 | Amb a 1 | 0 | Cry j 1 | 0,91 | Cup a 1 | 1,27 |
| Italy - CAAM 3697 | M | 38 | Amb a 1 | 0 | Cry j 1 | 0 | Cup a 1 | 1,36 |
| Italy - CAAM 3698 | M | 38 | Amb a 1 | 0 | Cry j 1 | 1,21 | Cup a 1 | 0 |
| Italy - CAAM 3699 | M | 38 | Amb a 1 | 0 | Cry j 1 | 1,9 | Cup a 1 | 7,84 |
| Italy - CAAM 3700 | M | 38 | Amb a 1 | 0 | Cry j 1 | 0 | Cup a 1 | 0,42 |
| Italy - CAAM 3701 | F | 38 | Amb a 1 | 0 | Cry j 1 | 8,74 | Cup a 1 | 29,86 |
| Italy - CAAM 3702 | F | 38 | Amb a 1 | 0 | Cry j 1 | 0 | Cup a 1 | 0,37 |
| Italy - CAAM 3703 | M | 38 | Amb a 1 | 0 | Cry j 1 | 0 | Cup a 1 | 0,1 |
| Italy - CAAM 3704 | F | 38 | Amb a 1 | 0 | Cry j 1 | 12,36 | Cup a 1 | 13,47 |
| Italy - CAAM 3705 | F | 38 | Amb a 1 | 0 | Cry j 1 | 3,05 | Cup a 1 | 5,15 |
| Italy - CAAM 3706 | M | 38 | Amb a 1 | 0 | Cry j 1 | 0,77 | Cup a 1 | 3,14 |
| Italy - CAAM 3707 | M | 38 | Amb a 1 | 0 | Cry j 1 | 0,31 | Cup a 1 | 0,66 |
| Italy - CAAM 3708 | F | 38 | Amb a 1 | 0 | Cry j 1 | 2,53 | Cup a 1 | 4,56 |
| Italy - CAAM 3709 | F | 38 | Amb a 1 | 0 | Cry j 1 | 0 | Cup a 1 | 0,22 |
| Italy - CAAM 3710 | M | 38 | Amb a 1 | 0 | Cry j 1 | 0,18 | Cup a 1 | 0,61 |
| Italy - CAAM 3711 | F | 38 | Amb a 1 | 0 | Cry j 1 | 0,11 | Cup a 1 | 0,13 |
| Italy - CAAM 3712 | M | 38 | Amb a 1 | 0 | Cry j 1 | 0,61 | Cup a 1 | 0,31 |
| Italy - CAAM 3713 | F | 38 | Amb a 1 | 0 | Cry j 1 | 0,21 | Cup a 1 | 0,98 |
| Italy - CAAM 3714 | M | 38 | Amb a 1 | 0 | Cry j 1 | 18 | Cup a 1 | 16,73 |
| Italy - CAAM 3715 | M | 38 | Amb a 1 | 0 | Cry j 1 | 26,52 | Cup a 1 | 16,97 |
| Italy - CAAM 3716 | M | 38 | Amb a 1 | 0 | Cry j 1 | 19,63 | Cup a 1 | 1,32 |
| Italy - CAAM 3717 | M | 38 | Amb a 1 | 0 | Cry j 1 | 5,12 | Cup a 1 | 0 |
| Italy - CAAM 3718 | F | 38 | Amb a 1 | 0 | Cry j 1 | 1,03 | Cup a 1 | 0,97 |
| Italy - CAAM 3719 | M | 38 | Amb a 1 | 0 | Cry j 1 | 0,26 | Cup a 1 | 0,5 |
| Italy - CAAM 3720 | M | 38 | Amb a 1 | 0 | Cry j 1 | 0 | Cup a 1 | 0,57 |
| Italy - CAAM 3721 | M | 38 | Amb a 1 | 0 | Cry j 1 | 0,84 | Cup a 1 | 1,81 |
| Italy - CAAM 3722 | F | 38 | Amb a 1 | 0 | Cry j 1 | 1,99 | Cup a 1 | 1,02 |
| Italy - CAAM 3723 | F | 38 | Amb a 1 | 0 | Cry j 1 | 0,16 | Cup a 1 | 1,65 |
| Italy - CAAM 3724 | M | 38 | Amb a 1 | 0 | Cry j 1 | 0,11 | Cup a 1 | 0,18 |
| Italy - CAAM 3725 | M | 38 | Amb a 1 | 0 | Cry j 1 | 0,19 | Cup a 1 | 2,06 |
| Italy - CAAM 3726 | M | 38 | Amb a 1 | 0 | Cry j 1 | 0,9 | Cup a 1 | 0,38 |
| Italy - CAAM 3727 | M | 38 | Amb a 1 | 0 | Cry j 1 | 0,14 | Cup a 1 | 2,27 |
| Italy - CAAM 3728 | F | 38 | Amb a 1 | 0 | Cry j 1 | 1,65 | Cup a 1 | 5,11 |
| Italy - CAAM 3729 | F | 38 | Amb a 1 | 0 | Cry j 1 | 3,93 | Cup a 1 | 7,09 |
| Italy - CAAM 3730 | F | 38 | Amb a 1 | 0 | Cry j 1 | 1,3 | Cup a 1 | 1,13 |
| Italy - CAAM 3731 | M | 38 | Amb a 1 | 0 | Cry j 1 | 0 | Cup a 1 | 0,04 |
| Italy - CAAM 3732 | F | 38 | Amb a 1 | 0 | Cry j 1 | 0 | Cup a 1 | 0,03 |
| Italy - CAAM 3733 | F | 38 | Amb a 1 | 0 | Cry j 1 | 8,35 | Cup a 1 | 10,12 |
| Italy - CAAM 3734 | F | 38 | Amb a 1 | 0 | Cry j 1 | 0,36 | Cup a 1 | 1,24 |
| Italy - CAAM 3735 | F | 38 | Amb a 1 | 0 | Cry j 1 | 0,52 | Cup a 1 | 0,71 |
| Italy - CAAM 3736 | M | 38 | Amb a 1 | 0 | Cry j 1 | 0,76 | Cup a 1 | 28,18 |
| Italy - CAAM 3737 | F | 38 | Amb a 1 | 0 | Cry j 1 | 1,01 | Cup a 1 | 4,94 |
| Italy - CAAM 3738 | M | 38 | Amb a 1 | 0 | Cry j 1 | 0 | Cup a 1 | 0,16 |
| Italy - CAAM 3739 | F | 38 | Amb a 1 | 0 | Cry j 1 | 1,54 | Cup a 1 | 4,18 |
| Italy - CAAM 3740 | M | 38 | Amb a 1 | 0 | Cry j 1 | 0 | Cup a 1 | 0,75 |
| Italy - CAAM 3741 | M | 38 | Amb a 1 | 0 | Cry j 1 | 0,94 | Cup a 1 | 6,25 |
| Italy - CAAM 3742 | M | 38 | Amb a 1 | 0 | Cry j 1 | 0,84 | Cup a 1 | 4,4 |
| Italy - CAAM 3743 | F | 38 | Amb a 1 | 0 | Cry j 1 | 0,15 | Cup a 1 | 0,18 |
| Italy - CAAM 3744 | F | 38 | Amb a 1 | 0 | Cry j 1 | 0,83 | Cup a 1 | 3,87 |
| Italy - CAAM 3745 | F | 38 | Amb a 1 | 0 | Cry j 1 | 2,66 | Cup a 1 | 7,74 |
| Italy - CAAM 3746 | F | 38 | Amb a 1 | 0 | Cry j 1 | 9,32 | Cup a 1 | 17,98 |
| Italy - CAAM 3747 | F | 38 | Amb a 1 | 0 | Cry j 1 | 1,08 | Cup a 1 | 19,12 |
| Italy - CAAM 3748 | M | 38 | Amb a 1 | 0 | Cry j 1 | 0,77 | Cup a 1 | 11,44 |
| Italy - CAAM 3749 | F | 38 | Amb a 1 | 0 | Cry j 1 | 0 | Cup a 1 | 0,45 |
| Italy - CAAM 3750 | M | 38 | Amb a 1 | 0 | Cry j 1 | 1,22 | Cup a 1 | 6,4 |
| Italy - CAAM 3751 | M | 38 | Amb a 1 | 0 | Cry j 1 | 1,7 | Cup a 1 | 6,96 |
| Italy - CAAM 3752 | F | 38 | Amb a 1 | 0 | Cry j 1 | 0,39 | Cup a 1 | 2,92 |
| Italy - CAAM 3753 | F | 38 | Amb a 1 | 0 | Cry j 1 | 0,02 | Cup a 1 | 0,16 |
| Italy - CAAM 3754 | F | 38 | Amb a 1 | 0 | Cry j 1 | 1,22 | Cup a 1 | 5,75 |
| Italy - CAAM 3755 | M | 38 | Amb a 1 | 0 | Cry j 1 | 0,08 | Cup a 1 | 0 |
| Italy - CAAM 3756 | F | 38 | Amb a 1 | 0 | Cry j 1 | 0,13 | Cup a 1 | 2,51 |
| Italy - CAAM 3757 | M | 38 | Amb a 1 | 0 | Cry j 1 | 0,11 | Cup a 1 | 2,59 |
| Italy - CAAM 3758 | M | 38 | Amb a 1 | 0 | Cry j 1 | 0 | Cup a 1 | 0,24 |
| Italy - CAAM 3759 | M | 38 | Amb a 1 | 0 | Cry j 1 | 0,67 | Cup a 1 | 3,49 |
| Italy - CAAM 3760 | M | 38 | Amb a 1 | 0 | Cry j 1 | 1,08 | Cup a 1 | 10,29 |
| Italy - CAAM 3761 | M | 38 | Amb a 1 | 0 | Cry j 1 | 4,35 | Cup a 1 | 7,83 |
| Italy - CAAM 3762 | F | 38 | Amb a 1 | 0 | Cry j 1 | 0,15 | Cup a 1 | 1,13 |
| Italy - CAAM 3763 | F | 38 | Amb a 1 | 0 | Cry j 1 | 0,36 | Cup a 1 | 2,18 |
| Italy - CAAM 3764 | M | 38 | Amb a 1 | 0 | Cry j 1 | 0,14 | Cup a 1 | 3,9 |
| Italy - CAAM 3765 | F | 38 | Amb a 1 | 0 | Cry j 1 | 3,51 | Cup a 1 | 7,27 |
| Italy - CAAM 3766 | F | 38 | Amb a 1 | 0 | Cry j 1 | 0 | Cup a 1 | 0,1 |
| Italy - CAAM 3767 | F | 38 | Amb a 1 | 0 | Cry j 1 | 4,56 | Cup a 1 | 15,49 |
| Italy - CAAM 3768 | F | 38 | Amb a 1 | 0 | Cry j 1 | 30,9 | Cup a 1 | 46,86 |
| Italy - CAAM 3769 | M | 38 | Amb a 1 | 0 | Cry j 1 | 1,2 | Cup a 1 | 1,67 |
| Italy - CAAM 3770 | F | 38 | Amb a 1 | 0 | Cry j 1 | 0,34 | Cup a 1 | 3,76 |
| Italy - CAAM 3771 | M | 38 | Amb a 1 | 0 | Cry j 1 | 8,72 | Cup a 1 | 15,57 |
| Italy - CAAM 3772 | F | 38 | Amb a 1 | 0 | Cry j 1 | 0 | Cup a 1 | 2,61 |
| Italy - CAAM 3773 | F | 38 | Amb a 1 | 0 | Cry j 1 | 2,47 | Cup a 1 | 7,82 |
| Italy - CAAM 3774 | F | 38 | Amb a 1 | 0 | Cry j 1 | 4,2 | Cup a 1 | 11,87 |
| Italy - CAAM 3775 | M | 38 | Amb a 1 | 0 | Cry j 1 | 0 | Cup a 1 | 0,66 |
| Italy - CAAM 3776 | M | 38 | Amb a 1 | 0 | Cry j 1 | 0 | Cup a 1 | 0,58 |
| Italy - CAAM 3777 | F | 38 | Amb a 1 | 0 | Cry j 1 | 4,18 | Cup a 1 | 4,92 |
| Italy - CAAM 3778 | M | 38 | Amb a 1 | 0 | Cry j 1 | 0,71 | Cup a 1 | 2,58 |
| Italy - CAAM 3779 | F | 38 | Amb a 1 | 0 | Cry j 1 | 0 | Cup a 1 | 1,97 |
| Italy - CAAM 3780 | F | 38 | Amb a 1 | 0 | Cry j 1 | 1,51 | Cup a 1 | 2,44 |
| Italy - CAAM 3781 | M | 38 | Amb a 1 | 0 | Cry j 1 | 1,9 | Cup a 1 | 2,4 |
| Italy - CAAM 3782 | F | 38 | Amb a 1 | 0 | Cry j 1 | 27,63 | Cup a 1 | 36,27 |
| Italy - CAAM 3783 | F | 38 | Amb a 1 | 0 | Cry j 1 | 1,73 | Cup a 1 | 0,74 |
| Italy - CAAM 3784 | M | 38 | Amb a 1 | 0 | Cry j 1 | 9,4 | Cup a 1 | 8,99 |
| Italy - CAAM 3785 | F | 38 | Amb a 1 | 0 | Cry j 1 | 3,54 | Cup a 1 | 8,46 |
| Italy - CAAM 3786 | M | 38 | Amb a 1 | 0 | Cry j 1 | 0,99 | Cup a 1 | 1,52 |
| Italy - CAAM 3787 | M | 38 | Amb a 1 | 0 | Cry j 1 | 0,97 | Cup a 1 | 1,96 |
| Italy - CAAM 3788 | F | 38 | Amb a 1 | 0 | Cry j 1 | 0 | Cup a 1 | 0,11 |
| Italy - CAAM 3789 | F | 38 | Amb a 1 | 0 | Cry j 1 | 0 | Cup a 1 | 0,78 |
| Italy - CAAM 3790 | M | 38 | Amb a 1 | 0 | Cry j 1 | 0,7 | Cup a 1 | 2,07 |
| Italy - CAAM 3791 | F | 38 | Amb a 1 | 0 | Cry j 1 | 3,84 | Cup a 1 | 11,61 |
| Italy - CAAM 3792 | F | 38 | Amb a 1 | 0 | Cry j 1 | 0 | Cup a 1 | 0,71 |
| Italy - CAAM 3793 | F | 38 | Amb a 1 | 0 | Cry j 1 | 4,04 | Cup a 1 | 23,61 |
| Italy - CAAM 3794 | F | 38 | Amb a 1 | 0 | Cry j 1 | 2,51 | Cup a 1 | 2,48 |
| Italy - CAAM 3795 | F | 38 | Amb a 1 | 0 | Cry j 1 | 0,27 | Cup a 1 | 2,08 |
| Italy - CAAM 3796 | F | 38 | Amb a 1 | 0 | Cry j 1 | 0,14 | Cup a 1 | 1,02 |
| Italy - CAAM 3797 | F | 38 | Amb a 1 | 0 | Cry j 1 | 0,88 | Cup a 1 | 2,56 |
| Italy - CAAM 3798 | F | 38 | Amb a 1 | 0 | Cry j 1 | 0,05 | Cup a 1 | 0 |
| Italy - CAAM 3799 | F | 38 | Amb a 1 | 0 | Cry j 1 | 0,08 | Cup a 1 | 0,67 |
| Italy - CAAM 3800 | F | 38 | Amb a 1 | 0 | Cry j 1 | 0,56 | Cup a 1 | 6,37 |
| Italy - CAAM 3801 | F | 38 | Amb a 1 | 0 | Cry j 1 | 0 | Cup a 1 | 0,15 |
| Italy - CAAM 3802 | F | 38 | Amb a 1 | 0 | Cry j 1 | 0,03 | Cup a 1 | 0,23 |
| Italy - CAAM 3803 | F | 38 | Amb a 1 | 0 | Cry j 1 | 0,15 | Cup a 1 | 0,58 |
| Italy - CAAM 3804 | F | 38 | Amb a 1 | 0 | Cry j 1 | 0,54 | Cup a 1 | 6,14 |
| Italy - CAAM 3805 | M | 38 | Amb a 1 | 0 | Cry j 1 | 0,85 | Cup a 1 | 2,56 |
| Italy - CAAM 3806 | F | 38 | Amb a 1 | 0 | Cry j 1 | 0 | Cup a 1 | 1,62 |
| Italy - CAAM 3807 | F | 38 | Amb a 1 | 0 | Cry j 1 | 0 | Cup a 1 | 2,91 |
| Italy - CAAM 3808 | F | 38 | Amb a 1 | 0 | Cry j 1 | 0 | Cup a 1 | 0,41 |
| Italy - CAAM 3809 | M | 38 | Amb a 1 | 0 | Cry j 1 | 0 | Cup a 1 | 0,46 |
| Italy - CAAM 3810 | F | 38 | Amb a 1 | 0 | Cry j 1 | 18,73 | Cup a 1 | 68,78 |
| Italy - CAAM 3811 | F | 38 | Amb a 1 | 0 | Cry j 1 | 0,4 | Cup a 1 | 1,82 |
| Italy - CAAM 3812 | F | 38 | Amb a 1 | 0 | Cry j 1 | 4,87 | Cup a 1 | 15,46 |
| Italy - CAAM 3813 | F | 38 | Amb a 1 | 0 | Cry j 1 | 9,62 | Cup a 1 | 15,46 |
| Italy - CAAM 3814 | F | 38 | Amb a 1 | 0 | Cry j 1 | 1,76 | Cup a 1 | 8,93 |
| Italy - CAAM 3815 | M | 38 | Amb a 1 | 0 | Cry j 1 | 0 | Cup a 1 | 1,25 |
| Italy - CAAM 3816 | M | 38 | Amb a 1 | 0 | Cry j 1 | 0,8 | Cup a 1 | 3,41 |
| Italy - CAAM 3817 | M | 38 | Amb a 1 | 0 | Cry j 1 | 0,45 | Cup a 1 | 11,57 |
| Italy - CAAM 3818 | M | 38 | Amb a 1 | 0 | Cry j 1 | 0,14 | Cup a 1 | 2,21 |
| Italy - CAAM 3819 | F | 38 | Amb a 1 | 0 | Cry j 1 | 2,37 | Cup a 1 | 6,59 |
| Italy - CAAM 3820 | F | 38 | Amb a 1 | 0 | Cry j 1 | 0 | Cup a 1 | 0,18 |
| Italy - CAAM 3821 | M | 38 | Amb a 1 | 0 | Cry j 1 | 8,99 | Cup a 1 | 33,81 |
| Italy - CAAM 3822 | F | 38 | Amb a 1 | 0 | Cry j 1 | 0,15 | Cup a 1 | 0,41 |
| Italy - CAAM 3823 | M | 38 | Amb a 1 | 0 | Cry j 1 | 2 | Cup a 1 | 13,59 |
| Italy - CAAM 3824 | F | 38 | Amb a 1 | 0 | Cry j 1 | 3,8 | Cup a 1 | 9 |
| Italy - CAAM 3825 | F | 38 | Amb a 1 | 0 | Cry j 1 | 30,08 | Cup a 1 | 65,93 |
| Italy - CAAM 3826 | M | 38 | Amb a 1 | 0 | Cry j 1 | 0,07 | Cup a 1 | 4,76 |
| Italy - CAAM 3827 | F | 38 | Amb a 1 | 0 | Cry j 1 | 0,29 | Cup a 1 | 13,58 |
| Italy - CAAM 3828 | F | 38 | Amb a 1 | 0 | Cry j 1 | 0,78 | Cup a 1 | 4,68 |
| Italy - CAAM 3829 | M | 38 | Amb a 1 | 0 | Cry j 1 | 0 | Cup a 1 | 1,19 |
| Italy - CAAM 3830 | M | 38 | Amb a 1 | 0 | Cry j 1 | 0 | Cup a 1 | 0,32 |
| Italy - CAAM 3831 | F | 38 | Amb a 1 | 0 | Cry j 1 | 0,37 | Cup a 1 | 2,14 |
| Italy - CAAM 3832 | M | 38 | Amb a 1 | 0 | Cry j 1 | 5,77 | Cup a 1 | 14,92 |
| Italy - CAAM 3833 | F | 38 | Amb a 1 | 0 | Cry j 1 | 0 | Cup a 1 | 2,93 |
| Italy - CAAM 3834 | F | 38 | Amb a 1 | 0 | Cry j 1 | 2,25 | Cup a 1 | 23,03 |
| Italy - CAAM 3835 | F | 38 | Amb a 1 | 0 | Cry j 1 | 0 | Cup a 1 | 3,78 |
| Italy - CAAM 3836 | F | 38 | Amb a 1 | 0 | Cry j 1 | 0 | Cup a 1 | 0,14 |
| Italy - CAAM 3837 | F | 38 | Amb a 1 | 0 | Cry j 1 | 0 | Cup a 1 | 4,55 |
| Italy - CAAM 3838 | F | 38 | Amb a 1 | 0 | Cry j 1 | 2,07 | Cup a 1 | 21,7 |
| Italy - CAAM 3839 | F | 38 | Amb a 1 | 0 | Cry j 1 | 0,49 | Cup a 1 | 7,68 |
| Italy - CAAM 3840 | M | 38 | Amb a 1 | 0 | Cry j 1 | 2,39 | Cup a 1 | 24,49 |
| Italy - CAAM 3841 | M | 38 | Amb a 1 | 0 | Cry j 1 | 0 | Cup a 1 | 1,28 |
| Italy - CAAM 3842 | F | 38 | Amb a 1 | 0 | Cry j 1 | 0 | Cup a 1 | 2,17 |
| Italy - CAAM 3843 | F | 38 | Amb a 1 | 0 | Cry j 1 | 0,31 | Cup a 1 | 3,09 |
| Italy - CAAM 3844 | M | 38 | Amb a 1 | 0 | Cry j 1 | 6,18 | Cup a 1 | 13,71 |
| Italy - CAAM 3845 | F | 38 | Amb a 1 | 0 | Cry j 1 | 0,7 | Cup a 1 | 17,03 |
| Italy - CAAM 3846 | F | 38 | Amb a 1 | 0 | Cry j 1 | 0 | Cup a 1 | 0,07 |
| Italy - CAAM 3847 | M | 38 | Amb a 1 | 0 | Cry j 1 | 0,74 | Cup a 1 | 11,24 |
| Italy - CAAM 3848 | F | 38 | Amb a 1 | 0 | Cry j 1 | 0,33 | Cup a 1 | 2,03 |
| Italy - CAAM 3849 | M | 38 | Amb a 1 | 0 | Cry j 1 | 10,61 | Cup a 1 | 52,86 |
| Italy - CAAM 3850 | F | 38 | Amb a 1 | 0 | Cry j 1 | 0 | Cup a 1 | 1,68 |
| Italy - CAAM 3851 | M | 38 | Amb a 1 | 0 | Cry j 1 | 1,31 | Cup a 1 | 5,04 |
| Italy - CAAM 3852 | M | 38 | Amb a 1 | 0 | Cry j 1 | 0,37 | Cup a 1 | 3,02 |
| Italy - CAAM 3853 | M | 38 | Amb a 1 | 0 | Cry j 1 | 1,82 | Cup a 1 | 26,94 |
| Italy - CAAM 3854 | F | 38 | Amb a 1 | 0 | Cry j 1 | 0,8 | Cup a 1 | 42 |
| Italy - CAAM 3855 | M | 38 | Amb a 1 | 0 | Cry j 1 | 0,3 | Cup a 1 | 7,2 |
| Italy - CAAM 3856 | F | 38 | Amb a 1 | 0 | Cry j 1 | 1,3 | Cup a 1 | 15 |
| Italy - CAAM 3857 | M | 38 | Amb a 1 | 0 | Cry j 1 | 0,41 | Cup a 1 | 11,11 |
| Italy - CAAM 3858 | M | 38 | Amb a 1 | 0 | Cry j 1 | 0,16 | Cup a 1 | 8,97 |
| Italy - CAAM 3859 | F | 38 | Amb a 1 | 0 | Cry j 1 | 0 | Cup a 1 | 0,69 |
| Italy - CAAM 3860 | M | 38 | Amb a 1 | 0 | Cry j 1 | 0,38 | Cup a 1 | 0,51 |
| Italy - CAAM 3861 | M | 38 | Amb a 1 | 0 | Cry j 1 | 0,23 | Cup a 1 | 13,54 |
| Italy - CAAM 3862 | F | 38 | Amb a 1 | 0 | Cry j 1 | 2,04 | Cup a 1 | 4,62 |
| Italy - CAAM 3863 | F | 37 | Amb a 1 | 0 | Cry j 1 | 0,3 | Cup a 1 | 2,07 |
| Italy - CAAM 3864 | F | 37 | Amb a 1 | 0 | Cry j 1 | 1,23 | Cup a 1 | 4,58 |
| Italy - CAAM 3865 | F | 37 | Amb a 1 | 0 | Cry j 1 | 8,97 | Cup a 1 | 19,17 |
| Italy - CAAM 3866 | F | 37 | Amb a 1 | 0 | Cry j 1 | 3,29 | Cup a 1 | 25,22 |
| Italy - CAAM 3867 | F | 37 | Amb a 1 | 0 | Cry j 1 | 0,76 | Cup a 1 | 3,53 |
| Italy - CAAM 3868 | M | 37 | Amb a 1 | 0 | Cry j 1 | 0 | Cup a 1 | 1,69 |
| Italy - CAAM 3869 | F | 37 | Amb a 1 | 0 | Cry j 1 | 0 | Cup a 1 | 1,09 |
| Italy - CAAM 3870 | F | 37 | Amb a 1 | 0 | Cry j 1 | 0,21 | Cup a 1 | 0,87 |
| Italy - CAAM 3871 | F | 37 | Amb a 1 | 0 | Cry j 1 | 0,12 | Cup a 1 | 1,15 |
| Italy - CAAM 3872 | F | 37 | Amb a 1 | 0 | Cry j 1 | 0 | Cup a 1 | 0,2 |
| Italy - CAAM 3873 | M | 37 | Amb a 1 | 0 | Cry j 1 | 0,98 | Cup a 1 | 0,87 |
| Italy - CAAM 3874 | M | 37 | Amb a 1 | 0 | Cry j 1 | 0,61 | Cup a 1 | 1,44 |
| Italy - CAAM 3875 | F | 37 | Amb a 1 | 0 | Cry j 1 | 9,18 | Cup a 1 | 20,85 |
| Italy - CAAM 3876 | F | 37 | Amb a 1 | 0 | Cry j 1 | 2,7 | Cup a 1 | 7,63 |
| Italy - CAAM 3877 | F | 37 | Amb a 1 | 0 | Cry j 1 | 1,43 | Cup a 1 | 1,1 |
| Italy - CAAM 3878 | F | 37 | Amb a 1 | 0 | Cry j 1 | 0 | Cup a 1 | 2,22 |
| Italy - CAAM 3879 | M | 37 | Amb a 1 | 0 | Cry j 1 | 0,89 | Cup a 1 | 0,89 |
| Italy - CAAM 3880 | F | 37 | Amb a 1 | 0 | Cry j 1 | 3,87 | Cup a 1 | 9,26 |
| Italy - CAAM 3881 | M | 37 | Amb a 1 | 0 | Cry j 1 | 0 | Cup a 1 | 2,28 |
| Italy - CAAM 3882 | F | 37 | Amb a 1 | 0 | Cry j 1 | 24,28 | Cup a 1 | 49,9 |
| Italy - CAAM 3883 | M | 37 | Amb a 1 | 0 | Cry j 1 | 0,35 | Cup a 1 | 3,25 |
| Italy - CAAM 3884 | M | 37 | Amb a 1 | 0 | Cry j 1 | 0,17 | Cup a 1 | 0 |
| Italy - CAAM 3885 | F | 37 | Amb a 1 | 0 | Cry j 1 | 2,9 | Cup a 1 | 4,66 |
| Italy - CAAM 3886 | F | 37 | Amb a 1 | 0 | Cry j 1 | 8,04 | Cup a 1 | 17,31 |
| Italy - CAAM 3887 | M | 37 | Amb a 1 | 0 | Cry j 1 | 0,08 | Cup a 1 | 0,14 |
| Italy - CAAM 3888 | M | 37 | Amb a 1 | 0 | Cry j 1 | 0 | Cup a 1 | 0,06 |
| Italy - CAAM 3889 | F | 37 | Amb a 1 | 0 | Cry j 1 | 0 | Cup a 1 | 0,09 |
| Italy - CAAM 3890 | F | 37 | Amb a 1 | 0 | Cry j 1 | 0 | Cup a 1 | 0,49 |
| Italy - CAAM 3891 | M | 37 | Amb a 1 | 0 | Cry j 1 | 1,95 | Cup a 1 | 4,79 |
| Italy - CAAM 3892 | M | 37 | Amb a 1 | 0 | Cry j 1 | 0 | Cup a 1 | 0,23 |
| Italy - CAAM 3893 | F | 37 | Amb a 1 | 0 | Cry j 1 | 0,24 | Cup a 1 | 0,85 |
| Italy - CAAM 3894 | F | 37 | Amb a 1 | 0 | Cry j 1 | 0 | Cup a 1 | 2,2 |
| Italy - CAAM 3895 | F | 37 | Amb a 1 | 0 | Cry j 1 | 0 | Cup a 1 | 1,08 |
| Italy - CAAM 3896 | F | 37 | Amb a 1 | 0 | Cry j 1 | 0,19 | Cup a 1 | 0,59 |
| Italy - CAAM 3897 | F | 37 | Amb a 1 | 0 | Cry j 1 | 12,63 | Cup a 1 | 6,89 |
| Italy - CAAM 3898 | M | 37 | Amb a 1 | 0 | Cry j 1 | 3,92 | Cup a 1 | 2,79 |
| Italy - CAAM 3899 | M | 37 | Amb a 1 | 0 | Cry j 1 | 0 | Cup a 1 | 0,26 |
| Italy - CAAM 3900 | F | 37 | Amb a 1 | 0 | Cry j 1 | 2,21 | Cup a 1 | 5,8 |
| Italy - CAAM 3901 | M | 37 | Amb a 1 | 0 | Cry j 1 | 0,25 | Cup a 1 | 0,5 |
| Italy - CAAM 3902 | M | 37 | Amb a 1 | 0 | Cry j 1 | 0,13 | Cup a 1 | 0,99 |
| Italy - CAAM 3903 | F | 37 | Amb a 1 | 0 | Cry j 1 | 4,66 | Cup a 1 | 6,63 |
| Italy - CAAM 3904 | M | 37 | Amb a 1 | 0 | Cry j 1 | 1,04 | Cup a 1 | 1,6 |
| Italy - CAAM 3905 | M | 37 | Amb a 1 | 0 | Cry j 1 | 7,05 | Cup a 1 | 10,64 |
| Italy - CAAM 3906 | M | 37 | Amb a 1 | 0 | Cry j 1 | 18,7 | Cup a 1 | 9,71 |
| Italy - CAAM 3907 | M | 37 | Amb a 1 | 0 | Cry j 1 | 0,21 | Cup a 1 | 0,53 |
| Italy - CAAM 3908 | M | 37 | Amb a 1 | 0 | Cry j 1 | 0,77 | Cup a 1 | 3,87 |
| Italy - CAAM 3909 | F | 37 | Amb a 1 | 0 | Cry j 1 | 0,1 | Cup a 1 | 0,15 |
| Italy - CAAM 3910 | F | 37 | Amb a 1 | 0 | Cry j 1 | 1,4 | Cup a 1 | 2,72 |
| Italy - CAAM 3911 | F | 37 | Amb a 1 | 0 | Cry j 1 | 7,58 | Cup a 1 | 5,96 |
| Italy - CAAM 3912 | M | 37 | Amb a 1 | 0 | Cry j 1 | 0,68 | Cup a 1 | 0,54 |
| Italy - CAAM 3913 | F | 37 | Amb a 1 | 0 | Cry j 1 | 0,21 | Cup a 1 | 0,37 |
| Italy - CAAM 3914 | F | 37 | Amb a 1 | 0 | Cry j 1 | 1,37 | Cup a 1 | 2,13 |
| Italy - CAAM 3915 | M | 37 | Amb a 1 | 0 | Cry j 1 | 0,72 | Cup a 1 | 0,33 |
| Italy - CAAM 3916 | M | 37 | Amb a 1 | 0 | Cry j 1 | 2,29 | Cup a 1 | 0,8 |
| Italy - CAAM 3917 | M | 37 | Amb a 1 | 0 | Cry j 1 | 12,57 | Cup a 1 | 12,19 |
| Italy - CAAM 3918 | F | 37 | Amb a 1 | 0 | Cry j 1 | 0,83 | Cup a 1 | 2,65 |
| Italy - CAAM 3919 | M | 37 | Amb a 1 | 0 | Cry j 1 | 17,43 | Cup a 1 | 22,67 |
| Italy - CAAM 3920 | F | 37 | Amb a 1 | 0 | Cry j 1 | 0,2 | Cup a 1 | 0,7 |
| Italy - CAAM 3921 | F | 37 | Amb a 1 | 0 | Cry j 1 | 18,91 | Cup a 1 | 10,39 |
| Italy - CAAM 3922 | F | 37 | Amb a 1 | 0 | Cry j 1 | 0,13 | Cup a 1 | 3,88 |
| Italy - CAAM 3923 | M | 37 | Amb a 1 | 0 | Cry j 1 | 0,5 | Cup a 1 | 0,2 |
| Italy - CAAM 3924 | F | 37 | Amb a 1 | 0 | Cry j 1 | 0,83 | Cup a 1 | 1,72 |
| Italy - CAAM 3925 | F | 37 | Amb a 1 | 0 | Cry j 1 | 0,42 | Cup a 1 | 11,41 |
| Italy - CAAM 3926 | M | 37 | Amb a 1 | 0 | Cry j 1 | 0,72 | Cup a 1 | 1,63 |
| Italy - CAAM 3927 | F | 37 | Amb a 1 | 0 | Cry j 1 | 0,33 | Cup a 1 | 1,1 |
| Italy - CAAM 3928 | M | 37 | Amb a 1 | 0 | Cry j 1 | 0 | Cup a 1 | 0,17 |
| Italy - CAAM 3929 | M | 37 | Amb a 1 | 0 | Cry j 1 | 4,61 | Cup a 1 | 7,72 |
| Italy - CAAM 3930 | F | 37 | Amb a 1 | 0 | Cry j 1 | 0 | Cup a 1 | 0,56 |
| Italy - CAAM 3931 | M | 37 | Amb a 1 | 0 | Cry j 1 | 3,54 | Cup a 1 | 5,52 |
| Italy - CAAM 3932 | M | 37 | Amb a 1 | 0 | Cry j 1 | 0,53 | Cup a 1 | 3,06 |
| Italy - CAAM 3933 | M | 37 | Amb a 1 | 0 | Cry j 1 | 0,06 | Cup a 1 | 0 |
| Italy - CAAM 3934 | M | 37 | Amb a 1 | 0 | Cry j 1 | 0,72 | Cup a 1 | 1,45 |
| Italy - CAAM 3935 | M | 37 | Amb a 1 | 0 | Cry j 1 | 0,16 | Cup a 1 | 0,07 |
| Italy - CAAM 3936 | M | 37 | Amb a 1 | 0 | Cry j 1 | 0,1 | Cup a 1 | 0,18 |
| Italy - CAAM 3937 | F | 37 | Amb a 1 | 0 | Cry j 1 | 0 | Cup a 1 | 0,41 |
| Italy - CAAM 3938 | F | 37 | Amb a 1 | 0 | Cry j 1 | 7,61 | Cup a 1 | 6,24 |
| Italy - CAAM 3939 | F | 37 | Amb a 1 | 0 | Cry j 1 | 0,33 | Cup a 1 | 2,25 |
| Italy - CAAM 3940 | F | 37 | Amb a 1 | 0 | Cry j 1 | 0,27 | Cup a 1 | 0,7 |
| Italy - CAAM 3941 | F | 37 | Amb a 1 | 0 | Cry j 1 | 0,49 | Cup a 1 | 3,44 |
| Italy - CAAM 3942 | F | 37 | Amb a 1 | 0 | Cry j 1 | 0 | Cup a 1 | 0,06 |
| Italy - CAAM 3943 | M | 37 | Amb a 1 | 0 | Cry j 1 | 0,05 | Cup a 1 | 0,78 |
| Italy - CAAM 3944 | M | 37 | Amb a 1 | 0 | Cry j 1 | 0,57 | Cup a 1 | 5,06 |
| Italy - CAAM 3945 | M | 37 | Amb a 1 | 0 | Cry j 1 | 0 | Cup a 1 | 2,57 |
| Italy - CAAM 3946 | M | 37 | Amb a 1 | 0 | Cry j 1 | 4,49 | Cup a 1 | 10,43 |
| Italy - CAAM 3947 | F | 37 | Amb a 1 | 0 | Cry j 1 | 10,94 | Cup a 1 | 17,99 |
| Italy - CAAM 3948 | M | 37 | Amb a 1 | 0 | Cry j 1 | 2,53 | Cup a 1 | 6,31 |
| Italy - CAAM 3949 | M | 37 | Amb a 1 | 0 | Cry j 1 | 0 | Cup a 1 | 0,21 |
| Italy - CAAM 3950 | M | 37 | Amb a 1 | 0 | Cry j 1 | 1,74 | Cup a 1 | 7,41 |
| Italy - CAAM 3951 | F | 37 | Amb a 1 | 0 | Cry j 1 | 0,23 | Cup a 1 | 2,24 |
| Italy - CAAM 3952 | F | 37 | Amb a 1 | 0 | Cry j 1 | 0,3 | Cup a 1 | 3,7 |
| Italy - CAAM 3953 | M | 37 | Amb a 1 | 0 | Cry j 1 | 0,58 | Cup a 1 | 1,8 |
| Italy - CAAM 3954 | F | 37 | Amb a 1 | 0 | Cry j 1 | 0,06 | Cup a 1 | 2,42 |
| Italy - CAAM 3955 | F | 37 | Amb a 1 | 0 | Cry j 1 | 1,03 | Cup a 1 | 3,55 |
| Italy - CAAM 3956 | M | 37 | Amb a 1 | 0 | Cry j 1 | 0,74 | Cup a 1 | 7,79 |
| Italy - CAAM 3957 | M | 37 | Amb a 1 | 0 | Cry j 1 | 0 | Cup a 1 | 0,43 |
| Italy - CAAM 3958 | M | 37 | Amb a 1 | 0 | Cry j 1 | 0,04 | Cup a 1 | 1,29 |
| Italy - CAAM 3959 | F | 37 | Amb a 1 | 0 | Cry j 1 | 0 | Cup a 1 | 0,17 |
| Italy - CAAM 3960 | F | 37 | Amb a 1 | 0 | Cry j 1 | 0,06 | Cup a 1 | 0,22 |
| Italy - CAAM 3961 | F | 37 | Amb a 1 | 0 | Cry j 1 | 0 | Cup a 1 | 0,65 |
| Italy - CAAM 3962 | M | 37 | Amb a 1 | 0 | Cry j 1 | 0,05 | Cup a 1 | 1,69 |
| Italy - CAAM 3963 | F | 37 | Amb a 1 | 0 | Cry j 1 | 0 | Cup a 1 | 0,26 |
| Italy - CAAM 3964 | M | 37 | Amb a 1 | 0 | Cry j 1 | 0,1 | Cup a 1 | 1,41 |
| Italy - CAAM 3965 | F | 37 | Amb a 1 | 0 | Cry j 1 | 0 | Cup a 1 | 0,3 |
| Italy - CAAM 3966 | F | 37 | Amb a 1 | 0 | Cry j 1 | 0 | Cup a 1 | 0,42 |
| Italy - CAAM 3967 | F | 37 | Amb a 1 | 0 | Cry j 1 | 0 | Cup a 1 | 0,35 |
| Italy - CAAM 3968 | M | 37 | Amb a 1 | 0 | Cry j 1 | 0,67 | Cup a 1 | 0,84 |
| Italy - CAAM 3969 | M | 37 | Amb a 1 | 0 | Cry j 1 | 7,74 | Cup a 1 | 18,71 |
| Italy - CAAM 3970 | F | 37 | Amb a 1 | 0 | Cry j 1 | 0 | Cup a 1 | 0,02 |
| Italy - CAAM 3971 | F | 37 | Amb a 1 | 0 | Cry j 1 | 0,56 | Cup a 1 | 4,35 |
| Italy - CAAM 3972 | F | 37 | Amb a 1 | 0 | Cry j 1 | 1,7 | Cup a 1 | 4,14 |
| Italy - CAAM 3973 | M | 37 | Amb a 1 | 0 | Cry j 1 | 2,37 | Cup a 1 | 5,13 |
| Italy - CAAM 3974 | F | 37 | Amb a 1 | 0 | Cry j 1 | 0 | Cup a 1 | 0,9 |
| Italy - CAAM 3975 | M | 37 | Amb a 1 | 0 | Cry j 1 | 0 | Cup a 1 | 1,16 |
| Italy - CAAM 3976 | F | 37 | Amb a 1 | 0 | Cry j 1 | 3,82 | Cup a 1 | 8,14 |
| Italy - CAAM 3977 | M | 37 | Amb a 1 | 0 | Cry j 1 | 4,18 | Cup a 1 | 20,08 |
| Italy - CAAM 3978 | F | 37 | Amb a 1 | 0 | Cry j 1 | 2,01 | Cup a 1 | 2,2 |
| Italy - CAAM 3979 | M | 37 | Amb a 1 | 0 | Cry j 1 | 1,77 | Cup a 1 | 7,21 |
| Italy - CAAM 3980 | F | 37 | Amb a 1 | 0 | Cry j 1 | 1,39 | Cup a 1 | 7,98 |
| Italy - CAAM 3981 | F | 37 | Amb a 1 | 0 | Cry j 1 | 2,23 | Cup a 1 | 5,47 |
| Italy - CAAM 3982 | M | 37 | Amb a 1 | 0 | Cry j 1 | 0 | Cup a 1 | 1,87 |
| Italy - CAAM 3983 | F | 37 | Amb a 1 | 0 | Cry j 1 | 0 | Cup a 1 | 0,58 |
| Italy - CAAM 3984 | F | 37 | Amb a 1 | 0 | Cry j 1 | 1,88 | Cup a 1 | 12,06 |
| Italy - CAAM 3985 | F | 37 | Amb a 1 | 0 | Cry j 1 | 0 | Cup a 1 | 0,97 |
| Italy - CAAM 3986 | F | 37 | Amb a 1 | 0 | Cry j 1 | 1,1 | Cup a 1 | 5,79 |
| Italy - CAAM 3987 | M | 37 | Amb a 1 | 0 | Cry j 1 | 1,36 | Cup a 1 | 0 |
| Italy - CAAM 3988 | M | 37 | Amb a 1 | 0 | Cry j 1 | 0,84 | Cup a 1 | 2,54 |
| Italy - CAAM 3989 | F | 37 | Amb a 1 | 0 | Cry j 1 | 2,89 | Cup a 1 | 4,37 |
| Italy - CAAM 3990 | M | 37 | Amb a 1 | 0 | Cry j 1 | 0,99 | Cup a 1 | 2,84 |
| Italy - CAAM 3991 | F | 37 | Amb a 1 | 0 | Cry j 1 | 0 | Cup a 1 | 0,03 |
| Italy - CAAM 3992 | M | 37 | Amb a 1 | 0 | Cry j 1 | 0,49 | Cup a 1 | 5,01 |
| Italy - CAAM 3993 | F | 37 | Amb a 1 | 0 | Cry j 1 | 0,09 | Cup a 1 | 6,67 |
| Italy - CAAM 3994 | F | 37 | Amb a 1 | 0 | Cry j 1 | 0 | Cup a 1 | 1,44 |
| Italy - CAAM 3995 | F | 37 | Amb a 1 | 0 | Cry j 1 | 0 | Cup a 1 | 4,76 |
| Italy - CAAM 3996 | M | 37 | Amb a 1 | 0 | Cry j 1 | 0,98 | Cup a 1 | 7,4 |
| Italy - CAAM 3997 | M | 37 | Amb a 1 | 0 | Cry j 1 | 0 | Cup a 1 | 0,2 |
| Italy - CAAM 3998 | M | 37 | Amb a 1 | 0 | Cry j 1 | 0 | Cup a 1 | 0,76 |
| Italy - CAAM 3999 | M | 37 | Amb a 1 | 0 | Cry j 1 | 2,38 | Cup a 1 | 19,68 |
| Italy - CAAM 4000 | M | 37 | Amb a 1 | 0 | Cry j 1 | 1,77 | Cup a 1 | 18,12 |
| Italy - CAAM 4001 | M | 37 | Amb a 1 | 0 | Cry j 1 | 2,71 | Cup a 1 | 5,78 |
| Italy - CAAM 4002 | F | 37 | Amb a 1 | 0 | Cry j 1 | 4,34 | Cup a 1 | 5,9 |
| Italy - CAAM 4003 | F | 37 | Amb a 1 | 0 | Cry j 1 | 4,4 | Cup a 1 | 40 |
| Italy - CAAM 4004 | M | 37 | Amb a 1 | 0 | Cry j 1 | 3,47 | Cup a 1 | 6,19 |
| Italy - CAAM 4005 | F | 37 | Amb a 1 | 0 | Cry j 1 | 9,28 | Cup a 1 | 27,48 |
| Italy - CAAM 4006 | F | 37 | Amb a 1 | 0 | Cry j 1 | 0,3 | Cup a 1 | 1,09 |
| Italy - CAAM 4007 | F | 37 | Amb a 1 | 0 | Cry j 1 | 6,38 | Cup a 1 | 25,64 |
| Italy - CAAM 4008 | M | 37 | Amb a 1 | 0 | Cry j 1 | 1,77 | Cup a 1 | 6,72 |
| Italy - CAAM 4009 | F | 37 | Amb a 1 | 0 | Cry j 1 | 2,73 | Cup a 1 | 6,9 |
| Italy - CAAM 4010 | F | 37 | Amb a 1 | 0 | Cry j 1 | 0 | Cup a 1 | 0,53 |
| Italy - CAAM 4011 | F | 37 | Amb a 1 | 0 | Cry j 1 | 0,32 | Cup a 1 | 0,75 |
| Italy - CAAM 4012 | F | 37 | Amb a 1 | 0 | Cry j 1 | 7,83 | Cup a 1 | 31,96 |
| Italy - CAAM 4013 | M | 37 | Amb a 1 | 0 | Cry j 1 | 0,9 | Cup a 1 | 2,4 |
| Italy - CAAM 4014 | M | 37 | Amb a 1 | 0 | Cry j 1 | 9,97 | Cup a 1 | 27,32 |
| Italy - CAAM 4015 | M | 37 | Amb a 1 | 0 | Cry j 1 | 0,71 | Cup a 1 | 1,33 |
| Italy - CAAM 4016 | M | 37 | Amb a 1 | 0 | Cry j 1 | 0,11 | Cup a 1 | 3,46 |
| Italy - CAAM 4017 | F | 37 | Amb a 1 | 0 | Cry j 1 | 0 | Cup a 1 | 0,17 |
| Italy - CAAM 4018 | F | 37 | Amb a 1 | 0 | Cry j 1 | 0 | Cup a 1 | 2,52 |
| Italy - CAAM 4019 | F | 37 | Amb a 1 | 0 | Cry j 1 | 15,17 | Cup a 1 | 54,11 |
| Italy - CAAM 4020 | F | 37 | Amb a 1 | 0 | Cry j 1 | 0,48 | Cup a 1 | 4,3 |
| Italy - CAAM 4021 | M | 37 | Amb a 1 | 0 | Cry j 1 | 0,78 | Cup a 1 | 4 |
| Italy - CAAM 4022 | F | 37 | Amb a 1 | 0 | Cry j 1 | 0 | Cup a 1 | 0,58 |
| Italy - CAAM 4023 | M | 37 | Amb a 1 | 0 | Cry j 1 | 1,12 | Cup a 1 | 6,01 |
| Italy - CAAM 4024 | F | 37 | Amb a 1 | 0 | Cry j 1 | 0,09 | Cup a 1 | 2,19 |
| Italy - CAAM 4025 | F | 37 | Amb a 1 | 0 | Cry j 1 | 1,83 | Cup a 1 | 6 |
| Italy - CAAM 4026 | F | 37 | Amb a 1 | 0 | Cry j 1 | 0 | Cup a 1 | 0,28 |
| Italy - CAAM 4027 | M | 37 | Amb a 1 | 0 | Cry j 1 | 0,09 | Cup a 1 | 0 |
| Italy - CAAM 4028 | F | 37 | Amb a 1 | 0 | Cry j 1 | 2,6 | Cup a 1 | 9,32 |
| Italy - CAAM 4029 | F | 37 | Amb a 1 | 0 | Cry j 1 | 0 | Cup a 1 | 0,75 |
| Italy - CAAM 4030 | F | 37 | Amb a 1 | 0 | Cry j 1 | 1,51 | Cup a 1 | 6,71 |
| Italy - CAAM 4031 | F | 37 | Amb a 1 | 0 | Cry j 1 | 0,13 | Cup a 1 | 1,67 |
| Italy - CAAM 4032 | M | 37 | Amb a 1 | 0 | Cry j 1 | 0,57 | Cup a 1 | 7,29 |
| Italy - CAAM 4033 | M | 37 | Amb a 1 | 0 | Cry j 1 | 1,15 | Cup a 1 | 8,53 |
| Italy - CAAM 4034 | F | 37 | Amb a 1 | 0 | Cry j 1 | 2,81 | Cup a 1 | 19,07 |
| Italy - CAAM 4035 | M | 37 | Amb a 1 | 0 | Cry j 1 | 0 | Cup a 1 | 1,81 |
| Italy - CAAM 4036 | F | 37 | Amb a 1 | 0 | Cry j 1 | 0 | Cup a 1 | 0,4 |
| Italy - CAAM 4037 | F | 37 | Amb a 1 | 0 | Cry j 1 | 0,65 | Cup a 1 | 2,36 |
| Italy - CAAM 4038 | M | 37 | Amb a 1 | 0 | Cry j 1 | 0 | Cup a 1 | 0,15 |
| Italy - CAAM 4039 | F | 37 | Amb a 1 | 0 | Cry j 1 | 0,7 | Cup a 1 | 10,71 |
| Italy - CAAM 4040 | F | 37 | Amb a 1 | 0 | Cry j 1 | 0,41 | Cup a 1 | 14,35 |
| Italy - CAAM 4041 | M | 37 | Amb a 1 | 0 | Cry j 1 | 0 | Cup a 1 | 0,48 |
| Italy - CAAM 4042 | F | 37 | Amb a 1 | 0 | Cry j 1 | 0 | Cup a 1 | 0,44 |
| Italy - CAAM 4043 | M | 37 | Amb a 1 | 0 | Cry j 1 | 3,62 | Cup a 1 | 27,94 |
| Italy - CAAM 4044 | M | 37 | Amb a 1 | 0 | Cry j 1 | 0,79 | Cup a 1 | 15,49 |
| Italy - CAAM 4045 | M | 37 | Amb a 1 | 0 | Cry j 1 | 2,25 | Cup a 1 | 16,07 |
| Italy - CAAM 4046 | F | 37 | Amb a 1 | 0 | Cry j 1 | 0,39 | Cup a 1 | 1,57 |
| Italy - CAAM 4047 | M | 37 | Amb a 1 | 0 | Cry j 1 | 1,64 | Cup a 1 | 2,95 |
| Italy - CAAM 4048 | M | 37 | Amb a 1 | 0 | Cry j 1 | 0,12 | Cup a 1 | 8,55 |
| Italy - CAAM 4049 | F | 37 | Amb a 1 | 0 | Cry j 1 | 1,24 | Cup a 1 | 7,33 |
| Italy - CAAM 4050 | M | 37 | Amb a 1 | 0 | Cry j 1 | 0,72 | Cup a 1 | 5,17 |
| Italy - CAAM 4051 | M | 37 | Amb a 1 | 0 | Cry j 1 | 0 | Cup a 1 | 0,1 |
| Italy - CAAM 4052 | M | 37 | Amb a 1 | 0 | Cry j 1 | 1,22 | Cup a 1 | 13,65 |
| Italy - CAAM 4053 | F | 37 | Amb a 1 | 0 | Cry j 1 | 1,36 | Cup a 1 | 9,7 |
| Italy - CAAM 4054 | M | 37 | Amb a 1 | 0 | Cry j 1 | 0,43 | Cup a 1 | 2,73 |
| Italy - CAAM 4055 | M | 37 | Amb a 1 | 0 | Cry j 1 | 1,45 | Cup a 1 | 14,85 |
| Italy - CAAM 4056 | F | 37 | Amb a 1 | 0 | Cry j 1 | 0,09 | Cup a 1 | 0,12 |
| Italy - CAAM 4057 | F | 37 | Amb a 1 | 0 | Cry j 1 | 0 | Cup a 1 | 33 |
| Italy - CAAM 4058 | M | 37 | Amb a 1 | 0 | Cry j 1 | 1 | Cup a 1 | 28 |
| Italy - CAAM 4059 | F | 37 | Amb a 1 | 0 | Cry j 1 | 0 | Cup a 1 | 3,3 |
| Italy - CAAM 4060 | F | 37 | Amb a 1 | 0 | Cry j 1 | 4,2 | Cup a 1 | 17 |
| Italy - CAAM 4061 | M | 37 | Amb a 1 | 0 | Cry j 1 | 1,23 | Cup a 1 | 14,85 |
| Italy - CAAM 4062 | F | 37 | Amb a 1 | 0 | Cry j 1 | 0 | Cup a 1 | 3,66 |
| Italy - CAAM 4063 | F | 37 | Amb a 1 | 0 | Cry j 1 | 0,05 | Cup a 1 | 1,86 |
| Italy - CAAM 4064 | F | 36 | Amb a 1 | 0 | Cry j 1 | 0,75 | Cup a 1 | 0,11 |
| Italy - CAAM 4065 | M | 36 | Amb a 1 | 0 | Cry j 1 | 0 | Cup a 1 | 0,21 |
| Italy - CAAM 4066 | M | 36 | Amb a 1 | 0 | Cry j 1 | 0 | Cup a 1 | 9,38 |
| Italy - CAAM 4067 | F | 36 | Amb a 1 | 0 | Cry j 1 | 1,68 | Cup a 1 | 7,97 |
| Italy - CAAM 4068 | F | 36 | Amb a 1 | 0 | Cry j 1 | 0 | Cup a 1 | 1,41 |
| Italy - CAAM 4069 | M | 36 | Amb a 1 | 0 | Cry j 1 | 0 | Cup a 1 | 1,76 |
| Italy - CAAM 4070 | M | 36 | Amb a 1 | 0 | Cry j 1 | 4,09 | Cup a 1 | 3,72 |
| Italy - CAAM 4071 | F | 36 | Amb a 1 | 0 | Cry j 1 | 1,86 | Cup a 1 | 12,83 |
| Italy - CAAM 4072 | F | 36 | Amb a 1 | 0 | Cry j 1 | 2,05 | Cup a 1 | 2,08 |
| Italy - CAAM 4073 | F | 36 | Amb a 1 | 0 | Cry j 1 | 0 | Cup a 1 | 1,14 |
| Italy - CAAM 4074 | M | 36 | Amb a 1 | 0 | Cry j 1 | 1,94 | Cup a 1 | 4,54 |
| Italy - CAAM 4075 | M | 36 | Amb a 1 | 0 | Cry j 1 | 0,11 | Cup a 1 | 1,53 |
| Italy - CAAM 4076 | F | 36 | Amb a 1 | 0 | Cry j 1 | 4,19 | Cup a 1 | 11,05 |
| Italy - CAAM 4077 | M | 36 | Amb a 1 | 0 | Cry j 1 | 9,46 | Cup a 1 | 9,34 |
| Italy - CAAM 4078 | M | 36 | Amb a 1 | 0 | Cry j 1 | 0 | Cup a 1 | 1,66 |
| Italy - CAAM 4079 | F | 36 | Amb a 1 | 0 | Cry j 1 | 3,42 | Cup a 1 | 7,62 |
| Italy - CAAM 4080 | M | 36 | Amb a 1 | 0 | Cry j 1 | 0,85 | Cup a 1 | 2,74 |
| Italy - CAAM 4081 | M | 36 | Amb a 1 | 0 | Cry j 1 | 0 | Cup a 1 | 3,56 |
| Italy - CAAM 4082 | M | 36 | Amb a 1 | 0 | Cry j 1 | 0,19 | Cup a 1 | 0,42 |
| Italy - CAAM 4083 | M | 36 | Amb a 1 | 0 | Cry j 1 | 0,11 | Cup a 1 | 0,13 |
| Italy - CAAM 4084 | M | 36 | Amb a 1 | 0 | Cry j 1 | 0,03 | Cup a 1 | 0 |
| Italy - CAAM 4085 | M | 36 | Amb a 1 | 0 | Cry j 1 | 0 | Cup a 1 | 0,78 |
| Italy - CAAM 4086 | M | 36 | Amb a 1 | 0 | Cry j 1 | 0,03 | Cup a 1 | 0 |
| Italy - CAAM 4087 | F | 36 | Amb a 1 | 0 | Cry j 1 | 0,08 | Cup a 1 | 0 |
| Italy - CAAM 4088 | F | 36 | Amb a 1 | 0 | Cry j 1 | 2,57 | Cup a 1 | 8,6 |
| Italy - CAAM 4089 | F | 36 | Amb a 1 | 0 | Cry j 1 | 1,22 | Cup a 1 | 2,15 |
| Italy - CAAM 4090 | M | 36 | Amb a 1 | 0 | Cry j 1 | 4,19 | Cup a 1 | 5,87 |
| Italy - CAAM 4091 | F | 36 | Amb a 1 | 0 | Cry j 1 | 0,8 | Cup a 1 | 4,28 |
| Italy - CAAM 4092 | F | 36 | Amb a 1 | 0 | Cry j 1 | 1,15 | Cup a 1 | 3,44 |
| Italy - CAAM 4093 | F | 36 | Amb a 1 | 0 | Cry j 1 | 0,39 | Cup a 1 | 1,45 |
| Italy - CAAM 4094 | F | 36 | Amb a 1 | 0 | Cry j 1 | 0,52 | Cup a 1 | 0,51 |
| Italy - CAAM 4095 | M | 36 | Amb a 1 | 0 | Cry j 1 | 1,18 | Cup a 1 | 2,22 |
| Italy - CAAM 4096 | F | 36 | Amb a 1 | 0 | Cry j 1 | 0,34 | Cup a 1 | 0,83 |
| Italy - CAAM 4097 | M | 36 | Amb a 1 | 0 | Cry j 1 | 0 | Cup a 1 | 2,3 |
| Italy - CAAM 4098 | M | 36 | Amb a 1 | 0 | Cry j 1 | 0,62 | Cup a 1 | 0,37 |
| Italy - CAAM 4099 | F | 36 | Amb a 1 | 0 | Cry j 1 | 4,45 | Cup a 1 | 4,03 |
| Italy - CAAM 4100 | M | 36 | Amb a 1 | 0 | Cry j 1 | 13,71 | Cup a 1 | 17,82 |
| Italy - CAAM 4101 | F | 36 | Amb a 1 | 0 | Cry j 1 | 0,71 | Cup a 1 | 0,36 |
| Italy - CAAM 4102 | F | 36 | Amb a 1 | 0 | Cry j 1 | 0,64 | Cup a 1 | 3,87 |
| Italy - CAAM 4103 | F | 36 | Amb a 1 | 0 | Cry j 1 | 0,39 | Cup a 1 | 3,68 |
| Italy - CAAM 4104 | F | 36 | Amb a 1 | 0 | Cry j 1 | 2,03 | Cup a 1 | 6,01 |
| Italy - CAAM 4105 | F | 36 | Amb a 1 | 0 | Cry j 1 | 3,53 | Cup a 1 | 6,9 |
| Italy - CAAM 4106 | F | 36 | Amb a 1 | 0 | Cry j 1 | 0 | Cup a 1 | 4,27 |
| Italy - CAAM 4107 | F | 36 | Amb a 1 | 0 | Cry j 1 | 0,7 | Cup a 1 | 5,6 |
| Italy - CAAM 4108 | F | 36 | Amb a 1 | 0 | Cry j 1 | 1,71 | Cup a 1 | 9,59 |
| Italy - CAAM 4109 | M | 36 | Amb a 1 | 0 | Cry j 1 | 0,24 | Cup a 1 | 1,24 |
| Italy - CAAM 4110 | M | 36 | Amb a 1 | 0 | Cry j 1 | 0,34 | Cup a 1 | 1,64 |
| Italy - CAAM 4111 | M | 36 | Amb a 1 | 0 | Cry j 1 | 2,33 | Cup a 1 | 3,75 |
| Italy - CAAM 4112 | F | 36 | Amb a 1 | 0 | Cry j 1 | 10,06 | Cup a 1 | 18,25 |
| Italy - CAAM 4113 | F | 36 | Amb a 1 | 0 | Cry j 1 | 3,09 | Cup a 1 | 8,58 |
| Italy - CAAM 4114 | F | 36 | Amb a 1 | 0 | Cry j 1 | 4,56 | Cup a 1 | 30,42 |
| Italy - CAAM 4115 | M | 36 | Amb a 1 | 0 | Cry j 1 | 0,05 | Cup a 1 | 3,06 |
| Italy - CAAM 4116 | M | 36 | Amb a 1 | 0 | Cry j 1 | 0,26 | Cup a 1 | 0,04 |
| Italy - CAAM 4117 | M | 36 | Amb a 1 | 0 | Cry j 1 | 0,87 | Cup a 1 | 3,44 |
| Italy - CAAM 4118 | M | 36 | Amb a 1 | 0 | Cry j 1 | 3,62 | Cup a 1 | 18,06 |
| Italy - CAAM 4119 | F | 36 | Amb a 1 | 0 | Cry j 1 | 0,53 | Cup a 1 | 1,39 |
| Italy - CAAM 4120 | M | 36 | Amb a 1 | 0 | Cry j 1 | 0,05 | Cup a 1 | 0,81 |
| Italy - CAAM 4121 | F | 36 | Amb a 1 | 0 | Cry j 1 | 0 | Cup a 1 | 0,17 |
| Italy - CAAM 4122 | M | 36 | Amb a 1 | 0 | Cry j 1 | 0 | Cup a 1 | 0,23 |
| Italy - CAAM 4123 | M | 36 | Amb a 1 | 0 | Cry j 1 | 0,05 | Cup a 1 | 0,1 |
| Italy - CAAM 4124 | M | 36 | Amb a 1 | 0 | Cry j 1 | 0,11 | Cup a 1 | 0,44 |
| Italy - CAAM 4125 | M | 36 | Amb a 1 | 0 | Cry j 1 | 2,83 | Cup a 1 | 5,34 |
| Italy - CAAM 4126 | F | 36 | Amb a 1 | 0 | Cry j 1 | 7,71 | Cup a 1 | 30,86 |
| Italy - CAAM 4127 | F | 36 | Amb a 1 | 0 | Cry j 1 | 4,29 | Cup a 1 | 34,08 |
| Italy - CAAM 4128 | M | 36 | Amb a 1 | 0 | Cry j 1 | 0,29 | Cup a 1 | 0,54 |
| Italy - CAAM 4129 | F | 36 | Amb a 1 | 0 | Cry j 1 | 0,33 | Cup a 1 | 2,06 |
| Italy - CAAM 4130 | F | 36 | Amb a 1 | 0 | Cry j 1 | 0 | Cup a 1 | 0,52 |
| Italy - CAAM 4131 | F | 36 | Amb a 1 | 0 | Cry j 1 | 0,91 | Cup a 1 | 3,11 |
| Italy - CAAM 4132 | M | 36 | Amb a 1 | 0 | Cry j 1 | 0 | Cup a 1 | 3,57 |
| Italy - CAAM 4133 | M | 36 | Amb a 1 | 0 | Cry j 1 | 2,06 | Cup a 1 | 2,54 |
| Italy - CAAM 4134 | F | 36 | Amb a 1 | 0 | Cry j 1 | 0,09 | Cup a 1 | 0,39 |
| Italy - CAAM 4135 | F | 36 | Amb a 1 | 0 | Cry j 1 | 0,04 | Cup a 1 | 1,87 |
| Italy - CAAM 4136 | F | 36 | Amb a 1 | 0 | Cry j 1 | 2,78 | Cup a 1 | 7,04 |
| Italy - CAAM 4137 | F | 36 | Amb a 1 | 0 | Cry j 1 | 0 | Cup a 1 | 1,2 |
| Italy - CAAM 4138 | M | 36 | Amb a 1 | 0 | Cry j 1 | 1,09 | Cup a 1 | 4,97 |
| Italy - CAAM 4139 | F | 36 | Amb a 1 | 0 | Cry j 1 | 0,8 | Cup a 1 | 2,74 |
| Italy - CAAM 4140 | M | 36 | Amb a 1 | 0 | Cry j 1 | 1,03 | Cup a 1 | 3,68 |
| Italy - CAAM 4141 | F | 36 | Amb a 1 | 0 | Cry j 1 | 0,35 | Cup a 1 | 2,1 |
| Italy - CAAM 4142 | F | 36 | Amb a 1 | 0 | Cry j 1 | 0,18 | Cup a 1 | 0,89 |
| Italy - CAAM 4143 | F | 36 | Amb a 1 | 0 | Cry j 1 | 0,4 | Cup a 1 | 2,96 |
| Italy - CAAM 4144 | M | 36 | Amb a 1 | 0 | Cry j 1 | 12,46 | Cup a 1 | 25,22 |
| Italy - CAAM 4145 | M | 36 | Amb a 1 | 0 | Cry j 1 | 0 | Cup a 1 | 0,08 |
| Italy - CAAM 4146 | M | 36 | Amb a 1 | 0 | Cry j 1 | 3,01 | Cup a 1 | 17,16 |
| Italy - CAAM 4147 | F | 36 | Amb a 1 | 0 | Cry j 1 | 0 | Cup a 1 | 0,03 |
| Italy - CAAM 4148 | M | 36 | Amb a 1 | 0 | Cry j 1 | 0 | Cup a 1 | 0,19 |
| Italy - CAAM 4149 | F | 36 | Amb a 1 | 0 | Cry j 1 | 0,79 | Cup a 1 | 3,28 |
| Italy - CAAM 4150 | F | 36 | Amb a 1 | 0 | Cry j 1 | 0,8 | Cup a 1 | 4,28 |
| Italy - CAAM 4151 | M | 36 | Amb a 1 | 0 | Cry j 1 | 0,3 | Cup a 1 | 0,71 |
| Italy - CAAM 4152 | F | 36 | Amb a 1 | 0 | Cry j 1 | 0,48 | Cup a 1 | 0,46 |
| Italy - CAAM 4153 | F | 36 | Amb a 1 | 0 | Cry j 1 | 0,15 | Cup a 1 | 1,26 |
| Italy - CAAM 4154 | F | 36 | Amb a 1 | 0 | Cry j 1 | 0,14 | Cup a 1 | 0,69 |
| Italy - CAAM 4155 | F | 36 | Amb a 1 | 0 | Cry j 1 | 0,36 | Cup a 1 | 2,9 |
| Italy - CAAM 4156 | F | 36 | Amb a 1 | 0 | Cry j 1 | 1,54 | Cup a 1 | 1,68 |
| Italy - CAAM 4157 | M | 36 | Amb a 1 | 0 | Cry j 1 | 0,44 | Cup a 1 | 0,83 |
| Italy - CAAM 4158 | M | 36 | Amb a 1 | 0 | Cry j 1 | 3,32 | Cup a 1 | 6,88 |
| Italy - CAAM 4159 | F | 36 | Amb a 1 | 0 | Cry j 1 | 7,31 | Cup a 1 | 20,72 |
| Italy - CAAM 4160 | M | 36 | Amb a 1 | 0 | Cry j 1 | 2,59 | Cup a 1 | 7,33 |
| Italy - CAAM 4161 | F | 36 | Amb a 1 | 0 | Cry j 1 | 0 | Cup a 1 | 0,09 |
| Italy - CAAM 4162 | M | 36 | Amb a 1 | 0 | Cry j 1 | 0 | Cup a 1 | 2,02 |
| Italy - CAAM 4163 | M | 36 | Amb a 1 | 0 | Cry j 1 | 0 | Cup a 1 | 0,55 |
| Italy - CAAM 4164 | M | 36 | Amb a 1 | 0 | Cry j 1 | 1,44 | Cup a 1 | 2,65 |
| Italy - CAAM 4165 | F | 36 | Amb a 1 | 0 | Cry j 1 | 0,02 | Cup a 1 | 0,02 |
| Italy - CAAM 4166 | M | 36 | Amb a 1 | 0 | Cry j 1 | 0,88 | Cup a 1 | 2,76 |
| Italy - CAAM 4167 | M | 36 | Amb a 1 | 0 | Cry j 1 | 3,15 | Cup a 1 | 7,51 |
| Italy - CAAM 4168 | F | 36 | Amb a 1 | 0 | Cry j 1 | 1,11 | Cup a 1 | 3,7 |
| Italy - CAAM 4169 | F | 36 | Amb a 1 | 0 | Cry j 1 | 0,73 | Cup a 1 | 9,6 |
| Italy - CAAM 4170 | F | 36 | Amb a 1 | 0 | Cry j 1 | 0,13 | Cup a 1 | 1,21 |
| Italy - CAAM 4171 | F | 36 | Amb a 1 | 0 | Cry j 1 | 2,39 | Cup a 1 | 16,64 |
| Italy - CAAM 4172 | F | 36 | Amb a 1 | 0 | Cry j 1 | 1,07 | Cup a 1 | 8,64 |
| Italy - CAAM 4173 | F | 36 | Amb a 1 | 0 | Cry j 1 | 0 | Cup a 1 | 1,22 |
| Italy - CAAM 4174 | M | 36 | Amb a 1 | 0 | Cry j 1 | 0,04 | Cup a 1 | 3,65 |
| Italy - CAAM 4175 | F | 36 | Amb a 1 | 0 | Cry j 1 | 0 | Cup a 1 | 0,31 |
| Italy - CAAM 4176 | M | 36 | Amb a 1 | 0 | Cry j 1 | 3,1 | Cup a 1 | 6,81 |
| Italy - CAAM 4177 | F | 36 | Amb a 1 | 0 | Cry j 1 | 0 | Cup a 1 | 0,05 |
| Italy - CAAM 4178 | F | 36 | Amb a 1 | 0 | Cry j 1 | 3,11 | Cup a 1 | 21,23 |
| Italy - CAAM 4179 | F | 36 | Amb a 1 | 0 | Cry j 1 | 7,39 | Cup a 1 | 18,1 |
| Italy - CAAM 4180 | F | 36 | Amb a 1 | 0 | Cry j 1 | 6,06 | Cup a 1 | 10,54 |
| Italy - CAAM 4181 | M | 36 | Amb a 1 | 0 | Cry j 1 | 0,3 | Cup a 1 | 2,77 |
| Italy - CAAM 4182 | F | 36 | Amb a 1 | 0 | Cry j 1 | 0,03 | Cup a 1 | 2,28 |
| Italy - CAAM 4183 | F | 36 | Amb a 1 | 0 | Cry j 1 | 0,27 | Cup a 1 | 2 |
| Italy - CAAM 4184 | F | 36 | Amb a 1 | 0 | Cry j 1 | 8,51 | Cup a 1 | 16,44 |
| Italy - CAAM 4185 | M | 36 | Amb a 1 | 0 | Cry j 1 | 0 | Cup a 1 | 0,65 |
| Italy - CAAM 4186 | F | 36 | Amb a 1 | 0 | Cry j 1 | 0 | Cup a 1 | 4,47 |
| Italy - CAAM 4187 | F | 36 | Amb a 1 | 0 | Cry j 1 | 0,35 | Cup a 1 | 2,07 |
| Italy - CAAM 4188 | M | 36 | Amb a 1 | 0 | Cry j 1 | 2,21 | Cup a 1 | 12,16 |
| Italy - CAAM 4189 | F | 36 | Amb a 1 | 0 | Cry j 1 | 0 | Cup a 1 | 0,71 |
| Italy - CAAM 4190 | M | 36 | Amb a 1 | 0 | Cry j 1 | 1,15 | Cup a 1 | 2,12 |
| Italy - CAAM 4191 | F | 36 | Amb a 1 | 0 | Cry j 1 | 0,24 | Cup a 1 | 3,32 |
| Italy - CAAM 4192 | F | 36 | Amb a 1 | 0 | Cry j 1 | 10,65 | Cup a 1 | 20,36 |
| Italy - CAAM 4193 | F | 36 | Amb a 1 | 0 | Cry j 1 | 0,53 | Cup a 1 | 7,62 |
| Italy - CAAM 4194 | M | 36 | Amb a 1 | 0 | Cry j 1 | 3,09 | Cup a 1 | 10,48 |
| Italy - CAAM 4195 | F | 36 | Amb a 1 | 0 | Cry j 1 | 0 | Cup a 1 | 3,01 |
| Italy - CAAM 4196 | F | 36 | Amb a 1 | 0 | Cry j 1 | 1,05 | Cup a 1 | 2,11 |
| Italy - CAAM 4197 | F | 36 | Amb a 1 | 0 | Cry j 1 | 1,09 | Cup a 1 | 1,07 |
| Italy - CAAM 4198 | F | 36 | Amb a 1 | 0 | Cry j 1 | 1,32 | Cup a 1 | 5,94 |
| Italy - CAAM 4199 | F | 36 | Amb a 1 | 0 | Cry j 1 | 0 | Cup a 1 | 0,28 |
| Italy - CAAM 4200 | F | 36 | Amb a 1 | 0 | Cry j 1 | 0 | Cup a 1 | 1,41 |
| Italy - CAAM 4201 | F | 36 | Amb a 1 | 0 | Cry j 1 | 0,14 | Cup a 1 | 2,17 |
| Italy - CAAM 4202 | F | 36 | Amb a 1 | 0 | Cry j 1 | 0 | Cup a 1 | 1,93 |
| Italy - CAAM 4203 | M | 36 | Amb a 1 | 0 | Cry j 1 | 9,42 | Cup a 1 | 31,06 |
| Italy - CAAM 4204 | M | 36 | Amb a 1 | 0 | Cry j 1 | 1,44 | Cup a 1 | 14,45 |
| Italy - CAAM 4205 | F | 36 | Amb a 1 | 0 | Cry j 1 | 2,65 | Cup a 1 | 9,61 |
| Italy - CAAM 4206 | F | 36 | Amb a 1 | 0 | Cry j 1 | 0,19 | Cup a 1 | 4,88 |
| Italy - CAAM 4207 | F | 36 | Amb a 1 | 0 | Cry j 1 | 0 | Cup a 1 | 0,29 |
| Italy - CAAM 4208 | F | 36 | Amb a 1 | 0 | Cry j 1 | 0 | Cup a 1 | 1,3 |
| Italy - CAAM 4209 | F | 36 | Amb a 1 | 0 | Cry j 1 | 1,77 | Cup a 1 | 9,54 |
| Italy - CAAM 4210 | M | 36 | Amb a 1 | 0 | Cry j 1 | 1,69 | Cup a 1 | 20,71 |
| Italy - CAAM 4211 | F | 36 | Amb a 1 | 0 | Cry j 1 | 0 | Cup a 1 | 0,82 |
| Italy - CAAM 4212 | M | 36 | Amb a 1 | 0 | Cry j 1 | 0,86 | Cup a 1 | 6,79 |
| Italy - CAAM 4213 | F | 36 | Amb a 1 | 0 | Cry j 1 | 3,28 | Cup a 1 | 5,86 |
| Italy - CAAM 4214 | M | 36 | Amb a 1 | 0 | Cry j 1 | 0 | Cup a 1 | 0,18 |
| Italy - CAAM 4215 | M | 36 | Amb a 1 | 0 | Cry j 1 | 0,8 | Cup a 1 | 16,36 |
| Italy - CAAM 4216 | F | 36 | Amb a 1 | 0 | Cry j 1 | 0 | Cup a 1 | 0,66 |
| Italy - CAAM 4217 | F | 36 | Amb a 1 | 0 | Cry j 1 | 0,09 | Cup a 1 | 2,55 |
| Italy - CAAM 4218 | M | 36 | Amb a 1 | 0 | Cry j 1 | 0,16 | Cup a 1 | 0,28 |
| Italy - CAAM 4219 | F | 36 | Amb a 1 | 0 | Cry j 1 | 0,46 | Cup a 1 | 13,47 |
| Italy - CAAM 4220 | M | 36 | Amb a 1 | 0 | Cry j 1 | 0,61 | Cup a 1 | 6,1 |
| Italy - CAAM 4221 | M | 36 | Amb a 1 | 0 | Cry j 1 | 0,65 | Cup a 1 | 4,03 |
| Italy - CAAM 4222 | M | 36 | Amb a 1 | 0 | Cry j 1 | 0,94 | Cup a 1 | 3,43 |
| Italy - CAAM 4223 | F | 36 | Amb a 1 | 0 | Cry j 1 | 2,3 | Cup a 1 | 20,58 |
| Italy - CAAM 4224 | M | 36 | Amb a 1 | 0 | Cry j 1 | 1,37 | Cup a 1 | 12,51 |
| Italy - CAAM 4225 | F | 36 | Amb a 1 | 0 | Cry j 1 | 0 | Cup a 1 | 7 |
| Italy - CAAM 4226 | M | 36 | Amb a 1 | 0 | Cry j 1 | 0 | Cup a 1 | 4,56 |
| Italy - CAAM 4227 | F | 36 | Amb a 1 | 0 | Cry j 1 | 1,5 | Cup a 1 | 15 |
| Italy - CAAM 4228 | M | 36 | Amb a 1 | 0 | Cry j 1 | 0 | Cup a 1 | 0,5 |
| Italy - CAAM 4229 | F | 36 | Amb a 1 | 0 | Cry j 1 | 0 | Cup a 1 | 3,81 |
| Italy - CAAM 4230 | F | 36 | Amb a 1 | 0 | Cry j 1 | 0,08 | Cup a 1 | 0,36 |
| Italy - CAAM 4231 | M | 36 | Amb a 1 | 0 | Cry j 1 | 0,11 | Cup a 1 | 1,91 |
| Italy - CAAM 4232 | F | 35 | Amb a 1 | 0 | Cry j 1 | 0 | Cup a 1 | 4,26 |
| Italy - CAAM 4233 | M | 35 | Amb a 1 | 0 | Cry j 1 | 0 | Cup a 1 | 3,81 |
| Italy - CAAM 4234 | M | 35 | Amb a 1 | 0 | Cry j 1 | 1,46 | Cup a 1 | 5,96 |
| Italy - CAAM 4235 | M | 35 | Amb a 1 | 0 | Cry j 1 | 0,24 | Cup a 1 | 0,36 |
| Italy - CAAM 4236 | M | 35 | Amb a 1 | 0 | Cry j 1 | 15,26 | Cup a 1 | 9,49 |
| Italy - CAAM 4237 | F | 35 | Amb a 1 | 0 | Cry j 1 | 2,77 | Cup a 1 | 1,6 |
| Italy - CAAM 4238 | M | 35 | Amb a 1 | 0 | Cry j 1 | 0,6 | Cup a 1 | 1,79 |
| Italy - CAAM 4239 | F | 35 | Amb a 1 | 0 | Cry j 1 | 0 | Cup a 1 | 2,14 |
| Italy - CAAM 4240 | F | 35 | Amb a 1 | 0 | Cry j 1 | 0 | Cup a 1 | 1,44 |
| Italy - CAAM 4241 | M | 35 | Amb a 1 | 0 | Cry j 1 | 0,26 | Cup a 1 | 0,75 |
| Italy - CAAM 4242 | F | 35 | Amb a 1 | 0 | Cry j 1 | 1,97 | Cup a 1 | 12,43 |
| Italy - CAAM 4243 | F | 35 | Amb a 1 | 0 | Cry j 1 | 0,16 | Cup a 1 | 2,08 |
| Italy - CAAM 4244 | F | 35 | Amb a 1 | 0 | Cry j 1 | 0 | Cup a 1 | 2,18 |
| Italy - CAAM 4245 | F | 35 | Amb a 1 | 0 | Cry j 1 | 3,62 | Cup a 1 | 2,4 |
| Italy - CAAM 4246 | F | 35 | Amb a 1 | 0 | Cry j 1 | 0 | Cup a 1 | 0,13 |
| Italy - CAAM 4247 | F | 35 | Amb a 1 | 0 | Cry j 1 | 1,85 | Cup a 1 | 2,75 |
| Italy - CAAM 4248 | F | 35 | Amb a 1 | 0 | Cry j 1 | 0,76 | Cup a 1 | 2,38 |
| Italy - CAAM 4249 | M | 35 | Amb a 1 | 0 | Cry j 1 | 0,14 | Cup a 1 | 1,1 |
| Italy - CAAM 4250 | F | 35 | Amb a 1 | 0 | Cry j 1 | 0,38 | Cup a 1 | 1,42 |
| Italy - CAAM 4251 | F | 35 | Amb a 1 | 0 | Cry j 1 | 0,3 | Cup a 1 | 2,77 |
| Italy - CAAM 4252 | M | 35 | Amb a 1 | 0 | Cry j 1 | 0 | Cup a 1 | 2,2 |
| Italy - CAAM 4253 | F | 35 | Amb a 1 | 0 | Cry j 1 | 6,85 | Cup a 1 | 20,31 |
| Italy - CAAM 4254 | M | 35 | Amb a 1 | 0 | Cry j 1 | 0,21 | Cup a 1 | 0,34 |
| Italy - CAAM 4255 | F | 35 | Amb a 1 | 0 | Cry j 1 | 0,09 | Cup a 1 | 1,81 |
| Italy - CAAM 4256 | F | 35 | Amb a 1 | 0 | Cry j 1 | 0,13 | Cup a 1 | 1,87 |
| Italy - CAAM 4257 | F | 35 | Amb a 1 | 0 | Cry j 1 | 0,58 | Cup a 1 | 0,82 |
| Italy - CAAM 4258 | F | 35 | Amb a 1 | 0 | Cry j 1 | 0,27 | Cup a 1 | 0,34 |
| Italy - CAAM 4259 | F | 35 | Amb a 1 | 0 | Cry j 1 | 0 | Cup a 1 | 2,45 |
| Italy - CAAM 4260 | F | 35 | Amb a 1 | 0 | Cry j 1 | 0,11 | Cup a 1 | 3,86 |
| Italy - CAAM 4261 | M | 35 | Amb a 1 | 0 | Cry j 1 | 3,12 | Cup a 1 | 6,57 |
| Italy - CAAM 4262 | F | 35 | Amb a 1 | 0 | Cry j 1 | 4,56 | Cup a 1 | 13,71 |
| Italy - CAAM 4263 | F | 35 | Amb a 1 | 0 | Cry j 1 | 1,33 | Cup a 1 | 4,85 |
| Italy - CAAM 4264 | F | 35 | Amb a 1 | 0 | Cry j 1 | 1,3 | Cup a 1 | 6,96 |
| Italy - CAAM 4265 | F | 35 | Amb a 1 | 0 | Cry j 1 | 2,3 | Cup a 1 | 2,46 |
| Italy - CAAM 4266 | F | 35 | Amb a 1 | 0 | Cry j 1 | 0,38 | Cup a 1 | 2,73 |
| Italy - CAAM 4267 | M | 35 | Amb a 1 | 0 | Cry j 1 | 3,2 | Cup a 1 | 5,04 |
| Italy - CAAM 4268 | F | 35 | Amb a 1 | 0 | Cry j 1 | 0,58 | Cup a 1 | 1,66 |
| Italy - CAAM 4269 | M | 35 | Amb a 1 | 0 | Cry j 1 | 4,93 | Cup a 1 | 27,51 |
| Italy - CAAM 4270 | M | 35 | Amb a 1 | 0 | Cry j 1 | 0,73 | Cup a 1 | 0 |
| Italy - CAAM 4271 | M | 35 | Amb a 1 | 0 | Cry j 1 | 1,56 | Cup a 1 | 2,38 |
| Italy - CAAM 4272 | F | 35 | Amb a 1 | 0 | Cry j 1 | 0 | Cup a 1 | 0,11 |
| Italy - CAAM 4273 | M | 35 | Amb a 1 | 0 | Cry j 1 | 0,5 | Cup a 1 | 0,44 |
| Italy - CAAM 4274 | F | 35 | Amb a 1 | 0 | Cry j 1 | 0 | Cup a 1 | 0,7 |
| Italy - CAAM 4275 | M | 35 | Amb a 1 | 0 | Cry j 1 | 13,15 | Cup a 1 | 9,81 |
| Italy - CAAM 4276 | F | 35 | Amb a 1 | 0 | Cry j 1 | 2,72 | Cup a 1 | 3,25 |
| Italy - CAAM 4277 | M | 35 | Amb a 1 | 0 | Cry j 1 | 1,87 | Cup a 1 | 1,97 |
| Italy - CAAM 4278 | M | 35 | Amb a 1 | 0 | Cry j 1 | 2,16 | Cup a 1 | 1,66 |
| Italy - CAAM 4279 | M | 35 | Amb a 1 | 0 | Cry j 1 | 0,13 | Cup a 1 | 0,12 |
| Italy - CAAM 4280 | F | 35 | Amb a 1 | 0 | Cry j 1 | 0,84 | Cup a 1 | 0,47 |
| Italy - CAAM 4281 | M | 35 | Amb a 1 | 0 | Cry j 1 | 2,35 | Cup a 1 | 11 |
| Italy - CAAM 4282 | M | 35 | Amb a 1 | 0 | Cry j 1 | 0 | Cup a 1 | 0,08 |
| Italy - CAAM 4283 | M | 35 | Amb a 1 | 0 | Cry j 1 | 0,92 | Cup a 1 | 0,23 |
| Italy - CAAM 4284 | F | 35 | Amb a 1 | 0 | Cry j 1 | 2,68 | Cup a 1 | 0 |
| Italy - CAAM 4285 | F | 35 | Amb a 1 | 0 | Cry j 1 | 1,54 | Cup a 1 | 3,63 |
| Italy - CAAM 4286 | M | 35 | Amb a 1 | 0 | Cry j 1 | 5,02 | Cup a 1 | 5,37 |
| Italy - CAAM 4287 | F | 35 | Amb a 1 | 0 | Cry j 1 | 2,83 | Cup a 1 | 10,38 |
| Italy - CAAM 4288 | F | 35 | Amb a 1 | 0 | Cry j 1 | 0,16 | Cup a 1 | 1,36 |
| Italy - CAAM 4289 | M | 35 | Amb a 1 | 0 | Cry j 1 | 0 | Cup a 1 | 0,37 |
| Italy - CAAM 4290 | F | 35 | Amb a 1 | 0 | Cry j 1 | 0,93 | Cup a 1 | 1,22 |
| Italy - CAAM 4291 | F | 35 | Amb a 1 | 0 | Cry j 1 | 0,44 | Cup a 1 | 1,82 |
| Italy - CAAM 4292 | M | 35 | Amb a 1 | 0 | Cry j 1 | 0 | Cup a 1 | 0,1 |
| Italy - CAAM 4293 | F | 35 | Amb a 1 | 0 | Cry j 1 | 0,11 | Cup a 1 | 0,55 |
| Italy - CAAM 4294 | M | 35 | Amb a 1 | 0 | Cry j 1 | 0 | Cup a 1 | 0,11 |
| Italy - CAAM 4295 | F | 35 | Amb a 1 | 0 | Cry j 1 | 0 | Cup a 1 | 0,03 |
| Italy - CAAM 4296 | F | 35 | Amb a 1 | 0 | Cry j 1 | 0,33 | Cup a 1 | 1,08 |
| Italy - CAAM 4297 | M | 35 | Amb a 1 | 0 | Cry j 1 | 33,18 | Cup a 1 | 63,79 |
| Italy - CAAM 4298 | F | 35 | Amb a 1 | 0 | Cry j 1 | 3,32 | Cup a 1 | 6,16 |
| Italy - CAAM 4299 | M | 35 | Amb a 1 | 0 | Cry j 1 | 0,77 | Cup a 1 | 1,48 |
| Italy - CAAM 4300 | F | 35 | Amb a 1 | 0 | Cry j 1 | 0,29 | Cup a 1 | 0,63 |
| Italy - CAAM 4301 | M | 35 | Amb a 1 | 0 | Cry j 1 | 1,09 | Cup a 1 | 1,2 |
| Italy - CAAM 4302 | F | 35 | Amb a 1 | 0 | Cry j 1 | 0 | Cup a 1 | 0,65 |
| Italy - CAAM 4303 | F | 35 | Amb a 1 | 0 | Cry j 1 | 6,64 | Cup a 1 | 24,32 |
| Italy - CAAM 4304 | F | 35 | Amb a 1 | 0 | Cry j 1 | 0 | Cup a 1 | 9,75 |
| Italy - CAAM 4305 | F | 35 | Amb a 1 | 0 | Cry j 1 | 0,27 | Cup a 1 | 0,77 |
| Italy - CAAM 4306 | F | 35 | Amb a 1 | 0 | Cry j 1 | 1,87 | Cup a 1 | 5,2 |
| Italy - CAAM 4307 | F | 35 | Amb a 1 | 0 | Cry j 1 | 0,08 | Cup a 1 | 0,83 |
| Italy - CAAM 4308 | M | 35 | Amb a 1 | 0 | Cry j 1 | 0,11 | Cup a 1 | 5,96 |
| Italy - CAAM 4309 | M | 35 | Amb a 1 | 0 | Cry j 1 | 1,12 | Cup a 1 | 2,36 |
| Italy - CAAM 4310 | M | 35 | Amb a 1 | 0 | Cry j 1 | 0 | Cup a 1 | 0,53 |
| Italy - CAAM 4311 | F | 35 | Amb a 1 | 0 | Cry j 1 | 0 | Cup a 1 | 0,1 |
| Italy - CAAM 4312 | M | 35 | Amb a 1 | 0 | Cry j 1 | 2,24 | Cup a 1 | 13,01 |
| Italy - CAAM 4313 | M | 35 | Amb a 1 | 0 | Cry j 1 | 7,77 | Cup a 1 | 6,09 |
| Italy - CAAM 4314 | M | 35 | Amb a 1 | 0 | Cry j 1 | 2,85 | Cup a 1 | 8,91 |
| Italy - CAAM 4315 | F | 35 | Amb a 1 | 0 | Cry j 1 | 7,1 | Cup a 1 | 39,13 |
| Italy - CAAM 4316 | F | 35 | Amb a 1 | 0 | Cry j 1 | 19,25 | Cup a 1 | 42,79 |
| Italy - CAAM 4317 | F | 35 | Amb a 1 | 0 | Cry j 1 | 0,39 | Cup a 1 | 14,93 |
| Italy - CAAM 4318 | M | 35 | Amb a 1 | 0 | Cry j 1 | 2,33 | Cup a 1 | 21,51 |
| Italy - CAAM 4319 | F | 35 | Amb a 1 | 0 | Cry j 1 | 0,32 | Cup a 1 | 1,41 |
| Italy - CAAM 4320 | M | 35 | Amb a 1 | 0 | Cry j 1 | 0 | Cup a 1 | 0,95 |
| Italy - CAAM 4321 | M | 35 | Amb a 1 | 0 | Cry j 1 | 2,51 | Cup a 1 | 14,16 |
| Italy - CAAM 4322 | M | 35 | Amb a 1 | 0 | Cry j 1 | 5,5 | Cup a 1 | 14,36 |
| Italy - CAAM 4323 | F | 35 | Amb a 1 | 0 | Cry j 1 | 0,85 | Cup a 1 | 10,14 |
| Italy - CAAM 4324 | M | 35 | Amb a 1 | 0 | Cry j 1 | 0,04 | Cup a 1 | 0,55 |
| Italy - CAAM 4325 | M | 35 | Amb a 1 | 0 | Cry j 1 | 0,92 | Cup a 1 | 2,85 |
| Italy - CAAM 4326 | F | 35 | Amb a 1 | 0 | Cry j 1 | 0,67 | Cup a 1 | 12,25 |
| Italy - CAAM 4327 | M | 35 | Amb a 1 | 0 | Cry j 1 | 0 | Cup a 1 | 1,8 |
| Italy - CAAM 4328 | F | 35 | Amb a 1 | 0 | Cry j 1 | 2,47 | Cup a 1 | 8,54 |
| Italy - CAAM 4329 | M | 35 | Amb a 1 | 0 | Cry j 1 | 1,56 | Cup a 1 | 13,97 |
| Italy - CAAM 4330 | F | 35 | Amb a 1 | 0 | Cry j 1 | 3,86 | Cup a 1 | 4,95 |
| Italy - CAAM 4331 | M | 35 | Amb a 1 | 0 | Cry j 1 | 4,23 | Cup a 1 | 22,74 |
| Italy - CAAM 4332 | F | 35 | Amb a 1 | 0 | Cry j 1 | 0 | Cup a 1 | 0,77 |
| Italy - CAAM 4333 | F | 35 | Amb a 1 | 0 | Cry j 1 | 0,83 | Cup a 1 | 3,57 |
| Italy - CAAM 4334 | M | 35 | Amb a 1 | 0 | Cry j 1 | 0,24 | Cup a 1 | 0,98 |
| Italy - CAAM 4335 | F | 35 | Amb a 1 | 0 | Cry j 1 | 0 | Cup a 1 | 8,09 |
| Italy - CAAM 4336 | M | 35 | Amb a 1 | 0 | Cry j 1 | 0 | Cup a 1 | 2,78 |
| Italy - CAAM 4337 | F | 35 | Amb a 1 | 0 | Cry j 1 | 1,35 | Cup a 1 | 15,61 |
| Italy - CAAM 4338 | F | 35 | Amb a 1 | 0 | Cry j 1 | 0,06 | Cup a 1 | 0,27 |
| Italy - CAAM 4339 | F | 35 | Amb a 1 | 0 | Cry j 1 | 0,07 | Cup a 1 | 0,04 |
| Italy - CAAM 4340 | F | 35 | Amb a 1 | 0 | Cry j 1 | 1,03 | Cup a 1 | 1,47 |
| Italy - CAAM 4341 | F | 35 | Amb a 1 | 0 | Cry j 1 | 1,87 | Cup a 1 | 3,51 |
| Italy - CAAM 4342 | F | 35 | Amb a 1 | 0 | Cry j 1 | 0 | Cup a 1 | 0,2 |
| Italy - CAAM 4343 | F | 35 | Amb a 1 | 0 | Cry j 1 | 0,73 | Cup a 1 | 19,6 |
| Italy - CAAM 4344 | M | 35 | Amb a 1 | 0 | Cry j 1 | 0,58 | Cup a 1 | 3,7 |
| Italy - CAAM 4345 | M | 35 | Amb a 1 | 0 | Cry j 1 | 0,22 | Cup a 1 | 0 |
| Italy - CAAM 4346 | F | 35 | Amb a 1 | 0 | Cry j 1 | 0,7 | Cup a 1 | 3,6 |
| Italy - CAAM 4347 | F | 35 | Amb a 1 | 0 | Cry j 1 | 1,15 | Cup a 1 | 15,25 |
| Italy - CAAM 4348 | F | 35 | Amb a 1 | 0 | Cry j 1 | 0,53 | Cup a 1 | 7,52 |
| Italy - CAAM 4349 | M | 35 | Amb a 1 | 0 | Cry j 1 | 14,82 | Cup a 1 | 20,96 |
| Italy - CAAM 4350 | M | 35 | Amb a 1 | 0 | Cry j 1 | 0,23 | Cup a 1 | 1,23 |
| Italy - CAAM 4351 | M | 35 | Amb a 1 | 0 | Cry j 1 | 0,75 | Cup a 1 | 3,04 |
| Italy - CAAM 4352 | F | 35 | Amb a 1 | 0 | Cry j 1 | 3,68 | Cup a 1 | 17,15 |
| Italy - CAAM 4353 | F | 35 | Amb a 1 | 0 | Cry j 1 | 6,64 | Cup a 1 | 27,78 |
| Italy - CAAM 4354 | F | 35 | Amb a 1 | 0 | Cry j 1 | 18,99 | Cup a 1 | 34,56 |
| Italy - CAAM 4355 | M | 35 | Amb a 1 | 0 | Cry j 1 | 0,07 | Cup a 1 | 1,57 |
| Italy - CAAM 4356 | M | 35 | Amb a 1 | 0 | Cry j 1 | 6,4 | Cup a 1 | 17,25 |
| Italy - CAAM 4357 | M | 35 | Amb a 1 | 0 | Cry j 1 | 0,15 | Cup a 1 | 0,96 |
| Italy - CAAM 4358 | M | 35 | Amb a 1 | 0 | Cry j 1 | 0,86 | Cup a 1 | 9,45 |
| Italy - CAAM 4359 | M | 35 | Amb a 1 | 0 | Cry j 1 | 0,11 | Cup a 1 | 14,66 |
| Italy - CAAM 4360 | F | 35 | Amb a 1 | 0 | Cry j 1 | 2,67 | Cup a 1 | 21,88 |
| Italy - CAAM 4361 | F | 35 | Amb a 1 | 0 | Cry j 1 | 0,04 | Cup a 1 | 0,64 |
| Italy - CAAM 4362 | M | 35 | Amb a 1 | 0 | Cry j 1 | 0,1 | Cup a 1 | 1,36 |
| Italy - CAAM 4363 | F | 35 | Amb a 1 | 0 | Cry j 1 | 0,17 | Cup a 1 | 2,94 |
| Italy - CAAM 4364 | M | 35 | Amb a 1 | 0 | Cry j 1 | 0 | Cup a 1 | 3,3 |
| Italy - CAAM 4365 | F | 35 | Amb a 1 | 0 | Cry j 1 | 0 | Cup a 1 | 2,46 |
| Italy - CAAM 4366 | F | 35 | Amb a 1 | 0 | Cry j 1 | 0,1 | Cup a 1 | 7,45 |
| Italy - CAAM 4367 | M | 35 | Amb a 1 | 0 | Cry j 1 | 1,08 | Cup a 1 | 18,67 |
| Italy - CAAM 4368 | M | 35 | Amb a 1 | 0 | Cry j 1 | 0 | Cup a 1 | 2,37 |
| Italy - CAAM 4369 | F | 35 | Amb a 1 | 0 | Cry j 1 | 0 | Cup a 1 | 3,09 |
| Italy - CAAM 4370 | F | 35 | Amb a 1 | 0 | Cry j 1 | 3,15 | Cup a 1 | 5,6 |
| Italy - CAAM 4371 | M | 35 | Amb a 1 | 0 | Cry j 1 | 0,4 | Cup a 1 | 0,35 |
| Italy - CAAM 4372 | F | 35 | Amb a 1 | 0 | Cry j 1 | 9,13 | Cup a 1 | 25,55 |
| Italy - CAAM 4373 | F | 35 | Amb a 1 | 0 | Cry j 1 | 0,38 | Cup a 1 | 7,5 |
| Italy - CAAM 4374 | F | 35 | Amb a 1 | 0 | Cry j 1 | 0,53 | Cup a 1 | 2,2 |
| Italy - CAAM 4375 | M | 35 | Amb a 1 | 0 | Cry j 1 | 0 | Cup a 1 | 5,02 |
| Italy - CAAM 4376 | M | 35 | Amb a 1 | 0 | Cry j 1 | 0,09 | Cup a 1 | 0,79 |
| Italy - CAAM 4377 | M | 35 | Amb a 1 | 0 | Cry j 1 | 0 | Cup a 1 | 0,17 |
| Italy - CAAM 4378 | F | 35 | Amb a 1 | 0 | Cry j 1 | 2,12 | Cup a 1 | 9,16 |
| Italy - CAAM 4379 | F | 35 | Amb a 1 | 0 | Cry j 1 | 0 | Cup a 1 | 0,07 |
| Italy - CAAM 4380 | F | 35 | Amb a 1 | 0 | Cry j 1 | 0 | Cup a 1 | 1,37 |
| Italy - CAAM 4381 | M | 35 | Amb a 1 | 0 | Cry j 1 | 0,54 | Cup a 1 | 5,15 |
| Italy - CAAM 4382 | F | 35 | Amb a 1 | 0 | Cry j 1 | 0 | Cup a 1 | 1,65 |
| Italy - CAAM 4383 | M | 35 | Amb a 1 | 0 | Cry j 1 | 0,61 | Cup a 1 | 16,39 |
| Italy - CAAM 4384 | F | 35 | Amb a 1 | 0 | Cry j 1 | 5,21 | Cup a 1 | 16,37 |
| Italy - CAAM 4385 | M | 35 | Amb a 1 | 0 | Cry j 1 | 2,5 | Cup a 1 | 15,38 |
| Italy - CAAM 4386 | F | 35 | Amb a 1 | 0 | Cry j 1 | 0 | Cup a 1 | 0,69 |
| Italy - CAAM 4387 | F | 35 | Amb a 1 | 0 | Cry j 1 | 3,5 | Cup a 1 | 18,99 |
| Italy - CAAM 4388 | M | 35 | Amb a 1 | 0 | Cry j 1 | 0 | Cup a 1 | 0,87 |
| Italy - CAAM 4389 | F | 35 | Amb a 1 | 0 | Cry j 1 | 0 | Cup a 1 | 6,6 |
| Italy - CAAM 4390 | F | 35 | Amb a 1 | 0 | Cry j 1 | 0 | Cup a 1 | 0,09 |
| Italy - CAAM 4391 | M | 35 | Amb a 1 | 0 | Cry j 1 | 0 | Cup a 1 | 0,31 |
| Italy - CAAM 4392 | M | 35 | Amb a 1 | 0 | Cry j 1 | 1,72 | Cup a 1 | 8,16 |
| Italy - CAAM 4393 | M | 35 | Amb a 1 | 0 | Cry j 1 | 5,74 | Cup a 1 | 52,82 |
| Italy - CAAM 4394 | F | 35 | Amb a 1 | 0 | Cry j 1 | 1 | Cup a 1 | 17,26 |
| Italy - CAAM 4395 | M | 35 | Amb a 1 | 0 | Cry j 1 | 0 | Cup a 1 | 0,5 |
| Italy - CAAM 4396 | F | 35 | Amb a 1 | 0 | Cry j 1 | 0 | Cup a 1 | 0,1 |
| Italy - CAAM 4397 | F | 35 | Amb a 1 | 0 | Cry j 1 | 0,34 | Cup a 1 | 7,97 |
| Italy - CAAM 4398 | F | 35 | Amb a 1 | 0 | Cry j 1 | 0,24 | Cup a 1 | 12,52 |
| Italy - CAAM 4399 | F | 35 | Amb a 1 | 0 | Cry j 1 | 4,21 | Cup a 1 | 49,83 |
| Italy - CAAM 4400 | F | 35 | Amb a 1 | 0 | Cry j 1 | 0,83 | Cup a 1 | 10,52 |
| Italy - CAAM 4401 | M | 35 | Amb a 1 | 0 | Cry j 1 | 4,7 | Cup a 1 | 36 |
| Italy - CAAM 4402 | F | 35 | Amb a 1 | 0 | Cry j 1 | 0 | Cup a 1 | 0,6 |
| Italy - CAAM 4403 | F | 35 | Amb a 1 | 0 | Cry j 1 | 0 | Cup a 1 | 0,17 |
| Italy - CAAM 4404 | F | 35 | Amb a 1 | 0 | Cry j 1 | 0 | Cup a 1 | 0,06 |
| Italy - CAAM 4405 | F | 35 | Amb a 1 | 0 | Cry j 1 | 1,2 | Cup a 1 | 6,4 |
| Italy - CAAM 4406 | F | 35 | Amb a 1 | 0 | Cry j 1 | 0,58 | Cup a 1 | 3,32 |
| Italy - CAAM 4407 | M | 35 | Amb a 1 | 0 | Cry j 1 | 0 | Cup a 1 | 5,6 |
| Italy - CAAM 4408 | F | 35 | Amb a 1 | 0 | Cry j 1 | 0 | Cup a 1 | 0,14 |
| Italy - CAAM 4409 | M | 35 | Amb a 1 | 0 | Cry j 1 | 0 | Cup a 1 | 0,24 |
| Italy - CAAM 4410 | M | 34 | Amb a 1 | 0 | Cry j 1 | 0,11 | Cup a 1 | 0,45 |
| Italy - CAAM 4411 | F | 34 | Amb a 1 | 0 | Cry j 1 | 1,89 | Cup a 1 | 20,84 |
| Italy - CAAM 4412 | M | 34 | Amb a 1 | 0 | Cry j 1 | 3,23 | Cup a 1 | 6,77 |
| Italy - CAAM 4413 | M | 34 | Amb a 1 | 0 | Cry j 1 | 0 | Cup a 1 | 0,51 |
| Italy - CAAM 4414 | M | 34 | Amb a 1 | 0 | Cry j 1 | 0 | Cup a 1 | 1,73 |
| Italy - CAAM 4415 | F | 34 | Amb a 1 | 0 | Cry j 1 | 0 | Cup a 1 | 3,03 |
| Italy - CAAM 4416 | M | 34 | Amb a 1 | 0 | Cry j 1 | 0,54 | Cup a 1 | 9,2 |
| Italy - CAAM 4417 | F | 34 | Amb a 1 | 0 | Cry j 1 | 1,64 | Cup a 1 | 18,17 |
| Italy - CAAM 4418 | F | 34 | Amb a 1 | 0 | Cry j 1 | 3,25 | Cup a 1 | 30,18 |
| Italy - CAAM 4419 | F | 34 | Amb a 1 | 0 | Cry j 1 | 0 | Cup a 1 | 7,88 |
| Italy - CAAM 4420 | M | 34 | Amb a 1 | 0 | Cry j 1 | 0,98 | Cup a 1 | 0,85 |
| Italy - CAAM 4421 | M | 34 | Amb a 1 | 0 | Cry j 1 | 1 | Cup a 1 | 8,59 |
| Italy - CAAM 4422 | M | 34 | Amb a 1 | 0 | Cry j 1 | 1,44 | Cup a 1 | 1,63 |
| Italy - CAAM 4423 | F | 34 | Amb a 1 | 0 | Cry j 1 | 0,81 | Cup a 1 | 25,07 |
| Italy - CAAM 4424 | F | 34 | Amb a 1 | 0 | Cry j 1 | 1,85 | Cup a 1 | 1,38 |
| Italy - CAAM 4425 | M | 34 | Amb a 1 | 0 | Cry j 1 | 0,03 | Cup a 1 | 0 |
| Italy - CAAM 4426 | F | 34 | Amb a 1 | 0 | Cry j 1 | 0,05 | Cup a 1 | 0,4 |
| Italy - CAAM 4427 | M | 34 | Amb a 1 | 0 | Cry j 1 | 1,42 | Cup a 1 | 5,84 |
| Italy - CAAM 4428 | F | 34 | Amb a 1 | 0 | Cry j 1 | 9,74 | Cup a 1 | 14,72 |
| Italy - CAAM 4429 | M | 34 | Amb a 1 | 0 | Cry j 1 | 0,29 | Cup a 1 | 0,82 |
| Italy - CAAM 4430 | F | 34 | Amb a 1 | 0 | Cry j 1 | 0,21 | Cup a 1 | 0,5 |
| Italy - CAAM 4431 | F | 34 | Amb a 1 | 0 | Cry j 1 | 0,84 | Cup a 1 | 1,53 |
| Italy - CAAM 4432 | M | 34 | Amb a 1 | 0 | Cry j 1 | 0,6 | Cup a 1 | 2,28 |
| Italy - CAAM 4433 | M | 34 | Amb a 1 | 0 | Cry j 1 | 0 | Cup a 1 | 0,29 |
| Italy - CAAM 4434 | F | 34 | Amb a 1 | 0 | Cry j 1 | 1,28 | Cup a 1 | 4,03 |
| Italy - CAAM 4435 | F | 34 | Amb a 1 | 0 | Cry j 1 | 7,05 | Cup a 1 | 20,53 |
| Italy - CAAM 4436 | M | 34 | Amb a 1 | 0 | Cry j 1 | 0 | Cup a 1 | 0,83 |
| Italy - CAAM 4437 | F | 34 | Amb a 1 | 0 | Cry j 1 | 0,11 | Cup a 1 | 0,26 |
| Italy - CAAM 4438 | M | 34 | Amb a 1 | 0 | Cry j 1 | 0,89 | Cup a 1 | 0,4 |
| Italy - CAAM 4439 | M | 34 | Amb a 1 | 0 | Cry j 1 | 0,94 | Cup a 1 | 7,45 |
| Italy - CAAM 4440 | F | 34 | Amb a 1 | 0 | Cry j 1 | 2,89 | Cup a 1 | 7,2 |
| Italy - CAAM 4441 | F | 34 | Amb a 1 | 0 | Cry j 1 | 0,27 | Cup a 1 | 2,14 |
| Italy - CAAM 4442 | M | 34 | Amb a 1 | 0 | Cry j 1 | 1,36 | Cup a 1 | 2,37 |
| Italy - CAAM 4443 | F | 34 | Amb a 1 | 0 | Cry j 1 | 1,79 | Cup a 1 | 0 |
| Italy - CAAM 4444 | M | 34 | Amb a 1 | 0 | Cry j 1 | 0 | Cup a 1 | 0,45 |
| Italy - CAAM 4445 | M | 34 | Amb a 1 | 0 | Cry j 1 | 14,19 | Cup a 1 | 2,47 |
| Italy - CAAM 4446 | F | 34 | Amb a 1 | 0 | Cry j 1 | 2,91 | Cup a 1 | 0 |
| Italy - CAAM 4447 | M | 34 | Amb a 1 | 0 | Cry j 1 | 8,99 | Cup a 1 | 6,12 |
| Italy - CAAM 4448 | F | 34 | Amb a 1 | 0 | Cry j 1 | 3,97 | Cup a 1 | 2,2 |
| Italy - CAAM 4449 | F | 34 | Amb a 1 | 0 | Cry j 1 | 0,48 | Cup a 1 | 0,94 |
| Italy - CAAM 4450 | F | 34 | Amb a 1 | 0 | Cry j 1 | 0,13 | Cup a 1 | 0,61 |
| Italy - CAAM 4451 | M | 34 | Amb a 1 | 0 | Cry j 1 | 6,42 | Cup a 1 | 5,76 |
| Italy - CAAM 4452 | F | 34 | Amb a 1 | 0 | Cry j 1 | 0,31 | Cup a 1 | 0,11 |
| Italy - CAAM 4453 | F | 34 | Amb a 1 | 0 | Cry j 1 | 0,31 | Cup a 1 | 0,4 |
| Italy - CAAM 4454 | F | 34 | Amb a 1 | 0 | Cry j 1 | 0,15 | Cup a 1 | 0,35 |
| Italy - CAAM 4455 | F | 34 | Amb a 1 | 0 | Cry j 1 | 4,93 | Cup a 1 | 34,15 |
| Italy - CAAM 4456 | M | 34 | Amb a 1 | 0 | Cry j 1 | 0,32 | Cup a 1 | 2,53 |
| Italy - CAAM 4457 | F | 34 | Amb a 1 | 0 | Cry j 1 | 2,25 | Cup a 1 | 7,45 |
| Italy - CAAM 4458 | F | 34 | Amb a 1 | 0 | Cry j 1 | 0 | Cup a 1 | 0,32 |
| Italy - CAAM 4459 | F | 34 | Amb a 1 | 0 | Cry j 1 | 1,53 | Cup a 1 | 15,36 |
| Italy - CAAM 4460 | F | 34 | Amb a 1 | 0 | Cry j 1 | 0,41 | Cup a 1 | 0,7 |
| Italy - CAAM 4461 | M | 34 | Amb a 1 | 0 | Cry j 1 | 0,53 | Cup a 1 | 2,11 |
| Italy - CAAM 4462 | M | 34 | Amb a 1 | 0 | Cry j 1 | 4,03 | Cup a 1 | 11,49 |
| Italy - CAAM 4463 | F | 34 | Amb a 1 | 0 | Cry j 1 | 0,1 | Cup a 1 | 0,8 |
| Italy - CAAM 4464 | M | 34 | Amb a 1 | 0 | Cry j 1 | 1,39 | Cup a 1 | 4,92 |
| Italy - CAAM 4465 | F | 34 | Amb a 1 | 0 | Cry j 1 | 2,31 | Cup a 1 | 24,76 |
| Italy - CAAM 4466 | M | 34 | Amb a 1 | 0 | Cry j 1 | 1,51 | Cup a 1 | 5,49 |
| Italy - CAAM 4467 | M | 34 | Amb a 1 | 0 | Cry j 1 | 0 | Cup a 1 | 0,07 |
| Italy - CAAM 4468 | F | 34 | Amb a 1 | 0 | Cry j 1 | 1,12 | Cup a 1 | 2,15 |
| Italy - CAAM 4469 | M | 34 | Amb a 1 | 0 | Cry j 1 | 6,88 | Cup a 1 | 28,61 |
| Italy - CAAM 4470 | F | 34 | Amb a 1 | 0 | Cry j 1 | 0,92 | Cup a 1 | 5,21 |
| Italy - CAAM 4471 | M | 34 | Amb a 1 | 0 | Cry j 1 | 6,5 | Cup a 1 | 10,22 |
| Italy - CAAM 4472 | M | 34 | Amb a 1 | 0 | Cry j 1 | 1,1 | Cup a 1 | 3,08 |
| Italy - CAAM 4473 | M | 34 | Amb a 1 | 0 | Cry j 1 | 1,21 | Cup a 1 | 4,33 |
| Italy - CAAM 4474 | F | 34 | Amb a 1 | 0 | Cry j 1 | 1,41 | Cup a 1 | 4,22 |
| Italy - CAAM 4475 | M | 34 | Amb a 1 | 0 | Cry j 1 | 0 | Cup a 1 | 2,69 |
| Italy - CAAM 4476 | F | 34 | Amb a 1 | 0 | Cry j 1 | 0,58 | Cup a 1 | 1,64 |
| Italy - CAAM 4477 | M | 34 | Amb a 1 | 0 | Cry j 1 | 3,69 | Cup a 1 | 9,49 |
| Italy - CAAM 4478 | M | 34 | Amb a 1 | 0 | Cry j 1 | 0,28 | Cup a 1 | 1,73 |
| Italy - CAAM 4479 | M | 34 | Amb a 1 | 0 | Cry j 1 | 0 | Cup a 1 | 0,39 |
| Italy - CAAM 4480 | F | 34 | Amb a 1 | 0 | Cry j 1 | 5,58 | Cup a 1 | 21,07 |
| Italy - CAAM 4481 | M | 34 | Amb a 1 | 0 | Cry j 1 | 0 | Cup a 1 | 0,03 |
| Italy - CAAM 4482 | F | 34 | Amb a 1 | 0 | Cry j 1 | 0,16 | Cup a 1 | 6,34 |
| Italy - CAAM 4483 | F | 34 | Amb a 1 | 0 | Cry j 1 | 0,05 | Cup a 1 | 3,03 |
| Italy - CAAM 4484 | F | 34 | Amb a 1 | 0 | Cry j 1 | 0,16 | Cup a 1 | 0,85 |
| Italy - CAAM 4485 | M | 34 | Amb a 1 | 0 | Cry j 1 | 2,23 | Cup a 1 | 4,69 |
| Italy - CAAM 4486 | M | 34 | Amb a 1 | 0 | Cry j 1 | 0 | Cup a 1 | 1,11 |
| Italy - CAAM 4487 | F | 34 | Amb a 1 | 0 | Cry j 1 | 8,4 | Cup a 1 | 19,55 |
| Italy - CAAM 4488 | M | 34 | Amb a 1 | 0 | Cry j 1 | 0,54 | Cup a 1 | 1,3 |
| Italy - CAAM 4489 | F | 34 | Amb a 1 | 0 | Cry j 1 | 1,11 | Cup a 1 | 10,71 |
| Italy - CAAM 4490 | M | 34 | Amb a 1 | 0 | Cry j 1 | 4,38 | Cup a 1 | 10,79 |
| Italy - CAAM 4491 | F | 34 | Amb a 1 | 0 | Cry j 1 | 0,77 | Cup a 1 | 5,57 |
| Italy - CAAM 4492 | M | 34 | Amb a 1 | 0 | Cry j 1 | 1,78 | Cup a 1 | 10,2 |
| Italy - CAAM 4493 | M | 34 | Amb a 1 | 0 | Cry j 1 | 1,33 | Cup a 1 | 3,37 |
| Italy - CAAM 4494 | F | 34 | Amb a 1 | 0 | Cry j 1 | 0 | Cup a 1 | 1,69 |
| Italy - CAAM 4495 | M | 34 | Amb a 1 | 0 | Cry j 1 | 0,32 | Cup a 1 | 2,65 |
| Italy - CAAM 4496 | F | 34 | Amb a 1 | 0 | Cry j 1 | 0,79 | Cup a 1 | 1,23 |
| Italy - CAAM 4497 | F | 34 | Amb a 1 | 0 | Cry j 1 | 0 | Cup a 1 | 0,48 |
| Italy - CAAM 4498 | F | 34 | Amb a 1 | 0 | Cry j 1 | 1,59 | Cup a 1 | 5,75 |
| Italy - CAAM 4499 | F | 34 | Amb a 1 | 0 | Cry j 1 | 1,97 | Cup a 1 | 7,06 |
| Italy - CAAM 4500 | F | 34 | Amb a 1 | 0 | Cry j 1 | 0 | Cup a 1 | 0,11 |
| Italy - CAAM 4501 | M | 34 | Amb a 1 | 0 | Cry j 1 | 0,24 | Cup a 1 | 2,27 |
| Italy - CAAM 4502 | F | 34 | Amb a 1 | 0 | Cry j 1 | 3,7 | Cup a 1 | 5,95 |
| Italy - CAAM 4503 | F | 34 | Amb a 1 | 0 | Cry j 1 | 0 | Cup a 1 | 1,96 |
| Italy - CAAM 4504 | M | 34 | Amb a 1 | 0 | Cry j 1 | 0 | Cup a 1 | 1,25 |
| Italy - CAAM 4505 | F | 34 | Amb a 1 | 0 | Cry j 1 | 0 | Cup a 1 | 0,42 |
| Italy - CAAM 4506 | F | 34 | Amb a 1 | 0 | Cry j 1 | 0,2 | Cup a 1 | 5,08 |
| Italy - CAAM 4507 | F | 34 | Amb a 1 | 0 | Cry j 1 | 1,4 | Cup a 1 | 0 |
| Italy - CAAM 4508 | F | 34 | Amb a 1 | 0 | Cry j 1 | 0,91 | Cup a 1 | 6,95 |
| Italy - CAAM 4509 | M | 34 | Amb a 1 | 0 | Cry j 1 | 0 | Cup a 1 | 1,01 |
| Italy - CAAM 4510 | F | 34 | Amb a 1 | 0 | Cry j 1 | 1,26 | Cup a 1 | 6,03 |
| Italy - CAAM 4511 | M | 34 | Amb a 1 | 0 | Cry j 1 | 0,82 | Cup a 1 | 1,37 |
| Italy - CAAM 4512 | F | 34 | Amb a 1 | 0 | Cry j 1 | 0 | Cup a 1 | 0,08 |
| Italy - CAAM 4513 | F | 34 | Amb a 1 | 0 | Cry j 1 | 3,69 | Cup a 1 | 8,95 |
| Italy - CAAM 4514 | F | 34 | Amb a 1 | 0 | Cry j 1 | 0 | Cup a 1 | 0,07 |
| Italy - CAAM 4515 | F | 34 | Amb a 1 | 0 | Cry j 1 | 0 | Cup a 1 | 2,77 |
| Italy - CAAM 4516 | M | 34 | Amb a 1 | 0 | Cry j 1 | 0 | Cup a 1 | 0,16 |
| Italy - CAAM 4517 | M | 34 | Amb a 1 | 0 | Cry j 1 | 2,14 | Cup a 1 | 3,33 |
| Italy - CAAM 4518 | F | 34 | Amb a 1 | 0 | Cry j 1 | 0,63 | Cup a 1 | 3,8 |
| Italy - CAAM 4519 | M | 34 | Amb a 1 | 0 | Cry j 1 | 0 | Cup a 1 | 7,09 |
| Italy - CAAM 4520 | F | 34 | Amb a 1 | 0 | Cry j 1 | 0,07 | Cup a 1 | 6,89 |
| Italy - CAAM 4521 | F | 34 | Amb a 1 | 0 | Cry j 1 | 0,13 | Cup a 1 | 0,11 |
| Italy - CAAM 4522 | F | 34 | Amb a 1 | 0 | Cry j 1 | 0,32 | Cup a 1 | 2,14 |
| Italy - CAAM 4523 | F | 34 | Amb a 1 | 0 | Cry j 1 | 0 | Cup a 1 | 0,9 |
| Italy - CAAM 4524 | F | 34 | Amb a 1 | 0 | Cry j 1 | 1,31 | Cup a 1 | 8,65 |
| Italy - CAAM 4525 | M | 34 | Amb a 1 | 0 | Cry j 1 | 5,03 | Cup a 1 | 28,31 |
| Italy - CAAM 4526 | M | 34 | Amb a 1 | 0 | Cry j 1 | 0,08 | Cup a 1 | 1,06 |
| Italy - CAAM 4527 | M | 34 | Amb a 1 | 0 | Cry j 1 | 0 | Cup a 1 | 1,57 |
| Italy - CAAM 4528 | F | 34 | Amb a 1 | 0 | Cry j 1 | 0 | Cup a 1 | 0,67 |
| Italy - CAAM 4529 | M | 34 | Amb a 1 | 0 | Cry j 1 | 1,31 | Cup a 1 | 1,49 |
| Italy - CAAM 4530 | F | 34 | Amb a 1 | 0 | Cry j 1 | 0 | Cup a 1 | 0,73 |
| Italy - CAAM 4531 | F | 34 | Amb a 1 | 0 | Cry j 1 | 0 | Cup a 1 | 0,62 |
| Italy - CAAM 4532 | F | 34 | Amb a 1 | 0 | Cry j 1 | 0,89 | Cup a 1 | 16,79 |
| Italy - CAAM 4533 | F | 34 | Amb a 1 | 0 | Cry j 1 | 0,23 | Cup a 1 | 4,85 |
| Italy - CAAM 4534 | F | 34 | Amb a 1 | 0 | Cry j 1 | 0 | Cup a 1 | 12,15 |
| Italy - CAAM 4535 | F | 34 | Amb a 1 | 0 | Cry j 1 | 0,15 | Cup a 1 | 1,96 |
| Italy - CAAM 4536 | M | 34 | Amb a 1 | 0 | Cry j 1 | 1,13 | Cup a 1 | 9,21 |
| Italy - CAAM 4537 | M | 34 | Amb a 1 | 0 | Cry j 1 | 0 | Cup a 1 | 2,82 |
| Italy - CAAM 4538 | F | 34 | Amb a 1 | 0 | Cry j 1 | 6,81 | Cup a 1 | 36,97 |
| Italy - CAAM 4539 | F | 34 | Amb a 1 | 0 | Cry j 1 | 0,07 | Cup a 1 | 1,58 |
| Italy - CAAM 4540 | M | 34 | Amb a 1 | 0 | Cry j 1 | 1,48 | Cup a 1 | 37,1 |
| Italy - CAAM 4541 | F | 34 | Amb a 1 | 0 | Cry j 1 | 0 | Cup a 1 | 0,69 |
| Italy - CAAM 4542 | F | 34 | Amb a 1 | 0 | Cry j 1 | 0 | Cup a 1 | 38,1 |
| Italy - CAAM 4543 | F | 34 | Amb a 1 | 0 | Cry j 1 | 0 | Cup a 1 | 1,15 |
| Italy - CAAM 4544 | F | 34 | Amb a 1 | 0 | Cry j 1 | 1,14 | Cup a 1 | 5,95 |
| Italy - CAAM 4545 | M | 34 | Amb a 1 | 0 | Cry j 1 | 0,97 | Cup a 1 | 4,7 |
| Italy - CAAM 4546 | M | 34 | Amb a 1 | 0 | Cry j 1 | 0 | Cup a 1 | 0,22 |
| Italy - CAAM 4547 | F | 34 | Amb a 1 | 0 | Cry j 1 | 0,25 | Cup a 1 | 7,57 |
| Italy - CAAM 4548 | M | 34 | Amb a 1 | 0 | Cry j 1 | 0 | Cup a 1 | 0,41 |
| Italy - CAAM 4549 | F | 34 | Amb a 1 | 0 | Cry j 1 | 0,97 | Cup a 1 | 7,77 |
| Italy - CAAM 4550 | M | 34 | Amb a 1 | 0 | Cry j 1 | 29,8 | Cup a 1 | 49,11 |
| Italy - CAAM 4551 | M | 34 | Amb a 1 | 0 | Cry j 1 | 0 | Cup a 1 | 5,46 |
| Italy - CAAM 4552 | F | 34 | Amb a 1 | 0 | Cry j 1 | 1,04 | Cup a 1 | 8,67 |
| Italy - CAAM 4553 | F | 34 | Amb a 1 | 0 | Cry j 1 | 1,12 | Cup a 1 | 4,52 |
| Italy - CAAM 4554 | M | 34 | Amb a 1 | 0 | Cry j 1 | 2,38 | Cup a 1 | 11,36 |
| Italy - CAAM 4555 | F | 34 | Amb a 1 | 0 | Cry j 1 | 0,8 | Cup a 1 | 3,7 |
| Italy - CAAM 4556 | M | 34 | Amb a 1 | 0 | Cry j 1 | 3,8 | Cup a 1 | 29 |
| Italy - CAAM 4557 | M | 34 | Amb a 1 | 0 | Cry j 1 | 7 | Cup a 1 | 51 |
| Italy - CAAM 4558 | F | 34 | Amb a 1 | 0 | Cry j 1 | 0 | Cup a 1 | 1,2 |
| Italy - CAAM 4559 | F | 34 | Amb a 1 | 0 | Cry j 1 | 0,5 | Cup a 1 | 11 |
| Italy - CAAM 4560 | F | 34 | Amb a 1 | 0 | Cry j 1 | 0,4 | Cup a 1 | 5,5 |
| Italy - CAAM 4561 | M | 34 | Amb a 1 | 0 | Cry j 1 | 1 | Cup a 1 | 16 |
| Italy - CAAM 4562 | M | 34 | Amb a 1 | 0 | Cry j 1 | 0,98 | Cup a 1 | 5,41 |
| Italy - CAAM 4563 | M | 34 | Amb a 1 | 0 | Cry j 1 | 0 | Cup a 1 | 1,6 |
| Italy - CAAM 4564 | F | 34 | Amb a 1 | 0 | Cry j 1 | 0 | Cup a 1 | 0,88 |
| Italy - CAAM 4565 | M | 34 | Amb a 1 | 0 | Cry j 1 | 11,1 | Cup a 1 | 48,09 |
| Italy - CAAM 4566 | M | 34 | Amb a 1 | 0 | Cry j 1 | 0 | Cup a 1 | 0,13 |
| Italy - CAAM 4567 | F | 34 | Amb a 1 | 0 | Cry j 1 | 1,7 | Cup a 1 | 27 |
| Italy - CAAM 4568 | F | 34 | Amb a 1 | 0 | Cry j 1 | 0 | Cup a 1 | 3,57 |
| Italy - CAAM 4569 | F | 33 | Amb a 1 | 0 | Cry j 1 | 0,36 | Cup a 1 | 1,92 |
| Italy - CAAM 4570 | M | 33 | Amb a 1 | 0 | Cry j 1 | 0,23 | Cup a 1 | 1,71 |
| Italy - CAAM 4571 | M | 33 | Amb a 1 | 0 | Cry j 1 | 0,03 | Cup a 1 | 0,5 |
| Italy - CAAM 4572 | M | 33 | Amb a 1 | 0 | Cry j 1 | 0,76 | Cup a 1 | 0 |
| Italy - CAAM 4573 | F | 33 | Amb a 1 | 0 | Cry j 1 | 0,05 | Cup a 1 | 0 |
| Italy - CAAM 4574 | M | 33 | Amb a 1 | 0 | Cry j 1 | 0,07 | Cup a 1 | 0,1 |
| Italy - CAAM 4575 | F | 33 | Amb a 1 | 0 | Cry j 1 | 0,08 | Cup a 1 | 0,27 |
| Italy - CAAM 4576 | F | 33 | Amb a 1 | 0 | Cry j 1 | 0 | Cup a 1 | 5,11 |
| Italy - CAAM 4577 | M | 33 | Amb a 1 | 0 | Cry j 1 | 6,05 | Cup a 1 | 4,67 |
| Italy - CAAM 4578 | M | 33 | Amb a 1 | 0 | Cry j 1 | 0,1 | Cup a 1 | 2,75 |
| Italy - CAAM 4579 | F | 33 | Amb a 1 | 0 | Cry j 1 | 0 | Cup a 1 | 1,8 |
| Italy - CAAM 4580 | F | 33 | Amb a 1 | 0 | Cry j 1 | 0,45 | Cup a 1 | 5,25 |
| Italy - CAAM 4581 | F | 33 | Amb a 1 | 0 | Cry j 1 | 0 | Cup a 1 | 2,16 |
| Italy - CAAM 4582 | F | 33 | Amb a 1 | 0 | Cry j 1 | 2,22 | Cup a 1 | 2,77 |
| Italy - CAAM 4583 | F | 33 | Amb a 1 | 0 | Cry j 1 | 11,6 | Cup a 1 | 12,67 |
| Italy - CAAM 4584 | M | 33 | Amb a 1 | 0 | Cry j 1 | 0,07 | Cup a 1 | 0,92 |
| Italy - CAAM 4585 | F | 33 | Amb a 1 | 0 | Cry j 1 | 0 | Cup a 1 | 0,09 |
| Italy - CAAM 4586 | M | 33 | Amb a 1 | 0 | Cry j 1 | 2,25 | Cup a 1 | 16,37 |
| Italy - CAAM 4587 | M | 33 | Amb a 1 | 0 | Cry j 1 | 3,74 | Cup a 1 | 5,08 |
| Italy - CAAM 4588 | F | 33 | Amb a 1 | 0 | Cry j 1 | 0 | Cup a 1 | 0,1 |
| Italy - CAAM 4589 | F | 33 | Amb a 1 | 0 | Cry j 1 | 0,1 | Cup a 1 | 0,85 |
| Italy - CAAM 4590 | F | 33 | Amb a 1 | 0 | Cry j 1 | 0 | Cup a 1 | 0,05 |
| Italy - CAAM 4591 | M | 33 | Amb a 1 | 0 | Cry j 1 | 3,85 | Cup a 1 | 7,67 |
| Italy - CAAM 4592 | M | 33 | Amb a 1 | 0 | Cry j 1 | 3,28 | Cup a 1 | 4,06 |
| Italy - CAAM 4593 | F | 33 | Amb a 1 | 0 | Cry j 1 | 15,35 | Cup a 1 | 21,1 |
| Italy - CAAM 4594 | M | 33 | Amb a 1 | 0 | Cry j 1 | 0,18 | Cup a 1 | 0,41 |
| Italy - CAAM 4595 | F | 33 | Amb a 1 | 0 | Cry j 1 | 2,68 | Cup a 1 | 4,96 |
| Italy - CAAM 4596 | F | 33 | Amb a 1 | 0 | Cry j 1 | 0,55 | Cup a 1 | 1,96 |
| Italy - CAAM 4597 | M | 33 | Amb a 1 | 0 | Cry j 1 | 0 | Cup a 1 | 0,22 |
| Italy - CAAM 4598 | F | 33 | Amb a 1 | 0 | Cry j 1 | 0,05 | Cup a 1 | 0,07 |
| Italy - CAAM 4599 | F | 33 | Amb a 1 | 0 | Cry j 1 | 0,42 | Cup a 1 | 1,29 |
| Italy - CAAM 4600 | M | 33 | Amb a 1 | 0 | Cry j 1 | 0,52 | Cup a 1 | 1,6 |
| Italy - CAAM 4601 | M | 33 | Amb a 1 | 0 | Cry j 1 | 2,65 | Cup a 1 | 2,34 |
| Italy - CAAM 4602 | M | 33 | Amb a 1 | 0 | Cry j 1 | 2,2 | Cup a 1 | 2,01 |
| Italy - CAAM 4603 | M | 33 | Amb a 1 | 0 | Cry j 1 | 1,4 | Cup a 1 | 25,15 |
| Italy - CAAM 4604 | M | 33 | Amb a 1 | 0 | Cry j 1 | 3,76 | Cup a 1 | 3,76 |
| Italy - CAAM 4605 | M | 33 | Amb a 1 | 0 | Cry j 1 | 30,3 | Cup a 1 | 2,63 |
| Italy - CAAM 4606 | F | 33 | Amb a 1 | 0 | Cry j 1 | 4,97 | Cup a 1 | 5,77 |
| Italy - CAAM 4607 | F | 33 | Amb a 1 | 0 | Cry j 1 | 0,77 | Cup a 1 | 0,96 |
| Italy - CAAM 4608 | M | 33 | Amb a 1 | 0 | Cry j 1 | 5,4 | Cup a 1 | 5,56 |
| Italy - CAAM 4609 | F | 33 | Amb a 1 | 0 | Cry j 1 | 1,53 | Cup a 1 | 0,43 |
| Italy - CAAM 4610 | M | 33 | Amb a 1 | 0 | Cry j 1 | 0,2 | Cup a 1 | 1,24 |
| Italy - CAAM 4611 | F | 33 | Amb a 1 | 0 | Cry j 1 | 1,73 | Cup a 1 | 2 |
| Italy - CAAM 4612 | F | 33 | Amb a 1 | 0 | Cry j 1 | 0 | Cup a 1 | 0,71 |
| Italy - CAAM 4613 | M | 33 | Amb a 1 | 0 | Cry j 1 | 0,15 | Cup a 1 | 0,44 |
| Italy - CAAM 4614 | F | 33 | Amb a 1 | 0 | Cry j 1 | 6,29 | Cup a 1 | 2,75 |
| Italy - CAAM 4615 | F | 33 | Amb a 1 | 0 | Cry j 1 | 0,6 | Cup a 1 | 2,02 |
| Italy - CAAM 4616 | M | 33 | Amb a 1 | 0 | Cry j 1 | 2,28 | Cup a 1 | 11,03 |
| Italy - CAAM 4617 | F | 33 | Amb a 1 | 0 | Cry j 1 | 0,39 | Cup a 1 | 0,8 |
| Italy - CAAM 4618 | M | 33 | Amb a 1 | 0 | Cry j 1 | 0,3 | Cup a 1 | 0,47 |
| Italy - CAAM 4619 | F | 33 | Amb a 1 | 0 | Cry j 1 | 2,06 | Cup a 1 | 1,25 |
| Italy - CAAM 4620 | F | 33 | Amb a 1 | 0 | Cry j 1 | 1,41 | Cup a 1 | 5,83 |
| Italy - CAAM 4621 | M | 33 | Amb a 1 | 0 | Cry j 1 | 0 | Cup a 1 | 0,87 |
| Italy - CAAM 4622 | M | 33 | Amb a 1 | 0 | Cry j 1 | 1,44 | Cup a 1 | 8,05 |
| Italy - CAAM 4623 | F | 33 | Amb a 1 | 0 | Cry j 1 | 1,85 | Cup a 1 | 7,88 |
| Italy - CAAM 4624 | F | 33 | Amb a 1 | 0 | Cry j 1 | 0,22 | Cup a 1 | 0,45 |
| Italy - CAAM 4625 | F | 33 | Amb a 1 | 0 | Cry j 1 | 0 | Cup a 1 | 0,19 |
| Italy - CAAM 4626 | F | 33 | Amb a 1 | 0 | Cry j 1 | 0,32 | Cup a 1 | 0,9 |
| Italy - CAAM 4627 | M | 33 | Amb a 1 | 0 | Cry j 1 | 9,92 | Cup a 1 | 22,6 |
| Italy - CAAM 4628 | M | 33 | Amb a 1 | 0 | Cry j 1 | 1,04 | Cup a 1 | 4,65 |
| Italy - CAAM 4629 | F | 33 | Amb a 1 | 0 | Cry j 1 | 0,5 | Cup a 1 | 6,22 |
| Italy - CAAM 4630 | M | 33 | Amb a 1 | 0 | Cry j 1 | 1,66 | Cup a 1 | 17,24 |
| Italy - CAAM 4631 | M | 33 | Amb a 1 | 0 | Cry j 1 | 1,42 | Cup a 1 | 3,1 |
| Italy - CAAM 4632 | M | 33 | Amb a 1 | 0 | Cry j 1 | 0,57 | Cup a 1 | 6,18 |
| Italy - CAAM 4633 | F | 33 | Amb a 1 | 0 | Cry j 1 | 0 | Cup a 1 | 0,26 |
| Italy - CAAM 4634 | F | 33 | Amb a 1 | 0 | Cry j 1 | 2,42 | Cup a 1 | 28,55 |
| Italy - CAAM 4635 | F | 33 | Amb a 1 | 0 | Cry j 1 | 0,09 | Cup a 1 | 0,15 |
| Italy - CAAM 4636 | F | 33 | Amb a 1 | 0 | Cry j 1 | 0 | Cup a 1 | 1,22 |
| Italy - CAAM 4637 | F | 33 | Amb a 1 | 0 | Cry j 1 | 0,05 | Cup a 1 | 5,66 |
| Italy - CAAM 4638 | F | 33 | Amb a 1 | 0 | Cry j 1 | 0,34 | Cup a 1 | 0,89 |
| Italy - CAAM 4639 | F | 33 | Amb a 1 | 0 | Cry j 1 | 0,24 | Cup a 1 | 3,06 |
| Italy - CAAM 4640 | M | 33 | Amb a 1 | 0 | Cry j 1 | 0,04 | Cup a 1 | 0,13 |
| Italy - CAAM 4641 | F | 33 | Amb a 1 | 0 | Cry j 1 | 0 | Cup a 1 | 1,09 |
| Italy - CAAM 4642 | F | 33 | Amb a 1 | 0 | Cry j 1 | 0,19 | Cup a 1 | 0,09 |
| Italy - CAAM 4643 | F | 33 | Amb a 1 | 0 | Cry j 1 | 0,08 | Cup a 1 | 0,66 |
| Italy - CAAM 4644 | F | 33 | Amb a 1 | 0 | Cry j 1 | 0 | Cup a 1 | 1,37 |
| Italy - CAAM 4645 | M | 33 | Amb a 1 | 0 | Cry j 1 | 0 | Cup a 1 | 1,08 |
| Italy - CAAM 4646 | F | 33 | Amb a 1 | 0 | Cry j 1 | 0,59 | Cup a 1 | 4,28 |
| Italy - CAAM 4647 | M | 33 | Amb a 1 | 0 | Cry j 1 | 0,43 | Cup a 1 | 5,59 |
| Italy - CAAM 4648 | F | 33 | Amb a 1 | 0 | Cry j 1 | 0 | Cup a 1 | 1,27 |
| Italy - CAAM 4649 | F | 33 | Amb a 1 | 0 | Cry j 1 | 0 | Cup a 1 | 0,12 |
| Italy - CAAM 4650 | M | 33 | Amb a 1 | 0 | Cry j 1 | 1,33 | Cup a 1 | 5,24 |
| Italy - CAAM 4651 | M | 33 | Amb a 1 | 0 | Cry j 1 | 0,47 | Cup a 1 | 0,25 |
| Italy - CAAM 4652 | M | 33 | Amb a 1 | 0 | Cry j 1 | 0,11 | Cup a 1 | 1,44 |
| Italy - CAAM 4653 | F | 33 | Amb a 1 | 0 | Cry j 1 | 1,87 | Cup a 1 | 8,74 |
| Italy - CAAM 4654 | F | 33 | Amb a 1 | 0 | Cry j 1 | 0,16 | Cup a 1 | 14,1 |
| Italy - CAAM 4655 | F | 33 | Amb a 1 | 0 | Cry j 1 | 2,6 | Cup a 1 | 7,41 |
| Italy - CAAM 4656 | M | 33 | Amb a 1 | 0 | Cry j 1 | 0 | Cup a 1 | 0,4 |
| Italy - CAAM 4657 | F | 33 | Amb a 1 | 0 | Cry j 1 | 0,23 | Cup a 1 | 1,9 |
| Italy - CAAM 4658 | F | 33 | Amb a 1 | 0 | Cry j 1 | 0,04 | Cup a 1 | 2,82 |
| Italy - CAAM 4659 | F | 33 | Amb a 1 | 0 | Cry j 1 | 7,95 | Cup a 1 | 25,59 |
| Italy - CAAM 4660 | F | 33 | Amb a 1 | 0 | Cry j 1 | 15,66 | Cup a 1 | 23,88 |
| Italy - CAAM 4661 | M | 33 | Amb a 1 | 0 | Cry j 1 | 1,66 | Cup a 1 | 15,54 |
| Italy - CAAM 4662 | F | 33 | Amb a 1 | 0 | Cry j 1 | 3,58 | Cup a 1 | 14,61 |
| Italy - CAAM 4663 | F | 33 | Amb a 1 | 0 | Cry j 1 | 1,06 | Cup a 1 | 10,47 |
| Italy - CAAM 4664 | F | 33 | Amb a 1 | 0 | Cry j 1 | 6,92 | Cup a 1 | 11,37 |
| Italy - CAAM 4665 | M | 33 | Amb a 1 | 0 | Cry j 1 | 3,32 | Cup a 1 | 11,2 |
| Italy - CAAM 4666 | F | 33 | Amb a 1 | 0 | Cry j 1 | 0,47 | Cup a 1 | 1,78 |
| Italy - CAAM 4667 | F | 33 | Amb a 1 | 0 | Cry j 1 | 0,64 | Cup a 1 | 3,43 |
| Italy - CAAM 4668 | M | 33 | Amb a 1 | 0 | Cry j 1 | 0,03 | Cup a 1 | 0,67 |
| Italy - CAAM 4669 | M | 33 | Amb a 1 | 0 | Cry j 1 | 0,75 | Cup a 1 | 2,92 |
| Italy - CAAM 4670 | M | 33 | Amb a 1 | 0 | Cry j 1 | 0,14 | Cup a 1 | 1,59 |
| Italy - CAAM 4671 | M | 33 | Amb a 1 | 0 | Cry j 1 | 0,07 | Cup a 1 | 0,24 |
| Italy - CAAM 4672 | F | 33 | Amb a 1 | 0 | Cry j 1 | 0,06 | Cup a 1 | 0,28 |
| Italy - CAAM 4673 | F | 33 | Amb a 1 | 0 | Cry j 1 | 0 | Cup a 1 | 3,97 |
| Italy - CAAM 4674 | M | 33 | Amb a 1 | 0 | Cry j 1 | 0 | Cup a 1 | 7,67 |
| Italy - CAAM 4675 | F | 33 | Amb a 1 | 0 | Cry j 1 | 0,02 | Cup a 1 | 0 |
| Italy - CAAM 4676 | M | 33 | Amb a 1 | 0 | Cry j 1 | 1,04 | Cup a 1 | 20,49 |
| Italy - CAAM 4677 | F | 33 | Amb a 1 | 0 | Cry j 1 | 0 | Cup a 1 | 0,24 |
| Italy - CAAM 4678 | F | 33 | Amb a 1 | 0 | Cry j 1 | 0 | Cup a 1 | 0,13 |
| Italy - CAAM 4679 | M | 33 | Amb a 1 | 0 | Cry j 1 | 0,09 | Cup a 1 | 0,92 |
| Italy - CAAM 4680 | F | 33 | Amb a 1 | 0 | Cry j 1 | 25,48 | Cup a 1 | 54,86 |
| Italy - CAAM 4681 | F | 33 | Amb a 1 | 0 | Cry j 1 | 2,96 | Cup a 1 | 6,98 |
| Italy - CAAM 4682 | M | 33 | Amb a 1 | 0 | Cry j 1 | 0 | Cup a 1 | 0,32 |
| Italy - CAAM 4683 | F | 33 | Amb a 1 | 0 | Cry j 1 | 5,67 | Cup a 1 | 12,87 |
| Italy - CAAM 4684 | M | 33 | Amb a 1 | 0 | Cry j 1 | 3,9 | Cup a 1 | 10,37 |
| Italy - CAAM 4685 | M | 33 | Amb a 1 | 0 | Cry j 1 | 3,38 | Cup a 1 | 13,32 |
| Italy - CAAM 4686 | M | 33 | Amb a 1 | 0 | Cry j 1 | 1,23 | Cup a 1 | 0 |
| Italy - CAAM 4687 | F | 33 | Amb a 1 | 0 | Cry j 1 | 3,74 | Cup a 1 | 15,41 |
| Italy - CAAM 4688 | M | 33 | Amb a 1 | 0 | Cry j 1 | 0 | Cup a 1 | 0,38 |
| Italy - CAAM 4689 | M | 33 | Amb a 1 | 0 | Cry j 1 | 0 | Cup a 1 | 0,29 |
| Italy - CAAM 4690 | F | 33 | Amb a 1 | 0 | Cry j 1 | 1,62 | Cup a 1 | 2,18 |
| Italy - CAAM 4691 | F | 33 | Amb a 1 | 0 | Cry j 1 | 0,38 | Cup a 1 | 4,31 |
| Italy - CAAM 4692 | F | 33 | Amb a 1 | 0 | Cry j 1 | 2,13 | Cup a 1 | 10,01 |
| Italy - CAAM 4693 | F | 33 | Amb a 1 | 0 | Cry j 1 | 4,13 | Cup a 1 | 7,18 |
| Italy - CAAM 4694 | M | 33 | Amb a 1 | 0 | Cry j 1 | 0,56 | Cup a 1 | 7,41 |
| Italy - CAAM 4695 | M | 33 | Amb a 1 | 0 | Cry j 1 | 1,32 | Cup a 1 | 5,5 |
| Italy - CAAM 4696 | F | 33 | Amb a 1 | 0 | Cry j 1 | 6,23 | Cup a 1 | 35,74 |
| Italy - CAAM 4697 | M | 33 | Amb a 1 | 0 | Cry j 1 | 1,44 | Cup a 1 | 6,57 |
| Italy - CAAM 4698 | M | 33 | Amb a 1 | 0 | Cry j 1 | 0,15 | Cup a 1 | 0,47 |
| Italy - CAAM 4699 | M | 33 | Amb a 1 | 0 | Cry j 1 | 8,38 | Cup a 1 | 16,93 |
| Italy - CAAM 4700 | F | 33 | Amb a 1 | 0 | Cry j 1 | 0,4 | Cup a 1 | 4,2 |
| Italy - CAAM 4701 | M | 33 | Amb a 1 | 0 | Cry j 1 | 0,04 | Cup a 1 | 0,64 |
| Italy - CAAM 4702 | F | 33 | Amb a 1 | 0 | Cry j 1 | 4,03 | Cup a 1 | 17,61 |
| Italy - CAAM 4703 | F | 33 | Amb a 1 | 0 | Cry j 1 | 2,48 | Cup a 1 | 3,27 |
| Italy - CAAM 4704 | F | 33 | Amb a 1 | 0 | Cry j 1 | 0 | Cup a 1 | 0,88 |
| Italy - CAAM 4705 | F | 33 | Amb a 1 | 0 | Cry j 1 | 3,28 | Cup a 1 | 25,6 |
| Italy - CAAM 4706 | F | 33 | Amb a 1 | 0 | Cry j 1 | 0,09 | Cup a 1 | 1,97 |
| Italy - CAAM 4707 | F | 33 | Amb a 1 | 0 | Cry j 1 | 0,04 | Cup a 1 | 1,53 |
| Italy - CAAM 4708 | M | 33 | Amb a 1 | 0 | Cry j 1 | 0,22 | Cup a 1 | 1,19 |
| Italy - CAAM 4709 | F | 33 | Amb a 1 | 0 | Cry j 1 | 0,68 | Cup a 1 | 5,7 |
| Italy - CAAM 4710 | F | 33 | Amb a 1 | 0 | Cry j 1 | 0 | Cup a 1 | 1,11 |
| Italy - CAAM 4711 | F | 33 | Amb a 1 | 0 | Cry j 1 | 0 | Cup a 1 | 0,19 |
| Italy - CAAM 4712 | F | 33 | Amb a 1 | 0 | Cry j 1 | 0 | Cup a 1 | 0,54 |
| Italy - CAAM 4713 | F | 33 | Amb a 1 | 0 | Cry j 1 | 2,41 | Cup a 1 | 12,81 |
| Italy - CAAM 4714 | F | 33 | Amb a 1 | 0 | Cry j 1 | 2 | Cup a 1 | 11,25 |
| Italy - CAAM 4715 | F | 33 | Amb a 1 | 0 | Cry j 1 | 1,71 | Cup a 1 | 23,8 |
| Italy - CAAM 4716 | F | 33 | Amb a 1 | 0 | Cry j 1 | 0,74 | Cup a 1 | 10,75 |
| Italy - CAAM 4717 | F | 33 | Amb a 1 | 0 | Cry j 1 | 5,73 | Cup a 1 | 26,41 |
| Italy - CAAM 4718 | F | 33 | Amb a 1 | 0 | Cry j 1 | 0,46 | Cup a 1 | 2,03 |
| Italy - CAAM 4719 | F | 33 | Amb a 1 | 0 | Cry j 1 | 0 | Cup a 1 | 0,54 |
| Italy - CAAM 4720 | F | 33 | Amb a 1 | 0 | Cry j 1 | 0,3 | Cup a 1 | 14,44 |
| Italy - CAAM 4721 | F | 33 | Amb a 1 | 0 | Cry j 1 | 0,43 | Cup a 1 | 12,19 |
| Italy - CAAM 4722 | F | 33 | Amb a 1 | 0 | Cry j 1 | 0,68 | Cup a 1 | 7,34 |
| Italy - CAAM 4723 | F | 33 | Amb a 1 | 0 | Cry j 1 | 0,23 | Cup a 1 | 1,52 |
| Italy - CAAM 4724 | M | 33 | Amb a 1 | 0 | Cry j 1 | 2,98 | Cup a 1 | 14,35 |
| Italy - CAAM 4725 | F | 33 | Amb a 1 | 0 | Cry j 1 | 1,5 | Cup a 1 | 7,13 |
| Italy - CAAM 4726 | F | 33 | Amb a 1 | 0 | Cry j 1 | 0 | Cup a 1 | 0,81 |
| Italy - CAAM 4727 | F | 33 | Amb a 1 | 0 | Cry j 1 | 0 | Cup a 1 | 0,24 |
| Italy - CAAM 4728 | F | 33 | Amb a 1 | 0 | Cry j 1 | 1,1 | Cup a 1 | 28,79 |
| Italy - CAAM 4729 | F | 33 | Amb a 1 | 0 | Cry j 1 | 0,63 | Cup a 1 | 14,69 |
| Italy - CAAM 4730 | F | 33 | Amb a 1 | 0 | Cry j 1 | 1,63 | Cup a 1 | 17,56 |
| Italy - CAAM 4731 | F | 33 | Amb a 1 | 0 | Cry j 1 | 5,69 | Cup a 1 | 55,71 |
| Italy - CAAM 4732 | M | 33 | Amb a 1 | 0 | Cry j 1 | 1,03 | Cup a 1 | 14,62 |
| Italy - CAAM 4733 | M | 33 | Amb a 1 | 0 | Cry j 1 | 0 | Cup a 1 | 0,56 |
| Italy - CAAM 4734 | M | 33 | Amb a 1 | 0 | Cry j 1 | 3,68 | Cup a 1 | 26,12 |
| Italy - CAAM 4735 | F | 33 | Amb a 1 | 0 | Cry j 1 | 1,42 | Cup a 1 | 6,21 |
| Italy - CAAM 4736 | M | 33 | Amb a 1 | 0 | Cry j 1 | 0,86 | Cup a 1 | 9,11 |
| Italy - CAAM 4737 | M | 33 | Amb a 1 | 0 | Cry j 1 | 0 | Cup a 1 | 0,46 |
| Italy - CAAM 4738 | F | 33 | Amb a 1 | 0 | Cry j 1 | 0 | Cup a 1 | 0,79 |
| Italy - CAAM 4739 | F | 33 | Amb a 1 | 0 | Cry j 1 | 5,5 | Cup a 1 | 20 |
| Italy - CAAM 4740 | F | 33 | Amb a 1 | 0 | Cry j 1 | 5,43 | Cup a 1 | 14,56 |
| Italy - CAAM 4741 | F | 33 | Amb a 1 | 0 | Cry j 1 | 0 | Cup a 1 | 1,06 |
| Italy - CAAM 4742 | F | 33 | Amb a 1 | 0 | Cry j 1 | 0,97 | Cup a 1 | 13,71 |
| Italy - CAAM 4743 | F | 33 | Amb a 1 | 0 | Cry j 1 | 0 | Cup a 1 | 27 |
| Italy - CAAM 4744 | F | 33 | Amb a 1 | 0 | Cry j 1 | 1,6 | Cup a 1 | 14 |
| Italy - CAAM 4745 | M | 33 | Amb a 1 | 0 | Cry j 1 | 0,5 | Cup a 1 | 3,3 |
| Italy - CAAM 4746 | F | 33 | Amb a 1 | 0 | Cry j 1 | 0 | Cup a 1 | 3,4 |
| Italy - CAAM 4747 | F | 33 | Amb a 1 | 0 | Cry j 1 | 0,13 | Cup a 1 | 1,3 |
| Italy - CAAM 4748 | F | 33 | Amb a 1 | 0 | Cry j 1 | 19,58 | Cup a 1 | 30,32 |
| Italy - CAAM 4749 | F | 32 | Amb a 1 | 0 | Cry j 1 | 9,56 | Cup a 1 | 42,99 |
| Italy - CAAM 4750 | M | 32 | Amb a 1 | 0 | Cry j 1 | 0,34 | Cup a 1 | 2,94 |
| Italy - CAAM 4751 | F | 32 | Amb a 1 | 0 | Cry j 1 | 1,96 | Cup a 1 | 9,49 |
| Italy - CAAM 4752 | F | 32 | Amb a 1 | 0 | Cry j 1 | 3,16 | Cup a 1 | 13,24 |
| Italy - CAAM 4753 | F | 32 | Amb a 1 | 0 | Cry j 1 | 0,09 | Cup a 1 | 0,55 |
| Italy - CAAM 4754 | F | 32 | Amb a 1 | 0 | Cry j 1 | 0,31 | Cup a 1 | 1,3 |
| Italy - CAAM 4755 | F | 32 | Amb a 1 | 0 | Cry j 1 | 0 | Cup a 1 | 1,53 |
| Italy - CAAM 4756 | F | 32 | Amb a 1 | 0 | Cry j 1 | 21,28 | Cup a 1 | 14,87 |
| Italy - CAAM 4757 | M | 32 | Amb a 1 | 0 | Cry j 1 | 1,01 | Cup a 1 | 0 |
| Italy - CAAM 4758 | F | 32 | Amb a 1 | 0 | Cry j 1 | 1,19 | Cup a 1 | 1,93 |
| Italy - CAAM 4759 | F | 32 | Amb a 1 | 0 | Cry j 1 | 0 | Cup a 1 | 5 |
| Italy - CAAM 4760 | F | 32 | Amb a 1 | 0 | Cry j 1 | 0,1 | Cup a 1 | 1,81 |
| Italy - CAAM 4761 | F | 32 | Amb a 1 | 0 | Cry j 1 | 0,37 | Cup a 1 | 1,43 |
| Italy - CAAM 4762 | M | 32 | Amb a 1 | 0 | Cry j 1 | 3,93 | Cup a 1 | 2,38 |
| Italy - CAAM 4763 | F | 32 | Amb a 1 | 0 | Cry j 1 | 0 | Cup a 1 | 2,66 |
| Italy - CAAM 4764 | F | 32 | Amb a 1 | 0 | Cry j 1 | 2,48 | Cup a 1 | 0 |
| Italy - CAAM 4765 | M | 32 | Amb a 1 | 0 | Cry j 1 | 0 | Cup a 1 | 2,84 |
| Italy - CAAM 4766 | M | 32 | Amb a 1 | 0 | Cry j 1 | 0 | Cup a 1 | 4,98 |
| Italy - CAAM 4767 | F | 32 | Amb a 1 | 0 | Cry j 1 | 1,51 | Cup a 1 | 3,69 |
| Italy - CAAM 4768 | F | 32 | Amb a 1 | 0 | Cry j 1 | 5,56 | Cup a 1 | 9,51 |
| Italy - CAAM 4769 | F | 32 | Amb a 1 | 0 | Cry j 1 | 0 | Cup a 1 | 2,48 |
| Italy - CAAM 4770 | F | 32 | Amb a 1 | 0 | Cry j 1 | 0 | Cup a 1 | 2,38 |
| Italy - CAAM 4771 | F | 32 | Amb a 1 | 0 | Cry j 1 | 0,14 | Cup a 1 | 2,13 |
| Italy - CAAM 4772 | F | 32 | Amb a 1 | 0 | Cry j 1 | 0 | Cup a 1 | 0,33 |
| Italy - CAAM 4773 | M | 32 | Amb a 1 | 0 | Cry j 1 | 0 | Cup a 1 | 0,78 |
| Italy - CAAM 4774 | M | 32 | Amb a 1 | 0 | Cry j 1 | 0 | Cup a 1 | 0,19 |
| Italy - CAAM 4775 | M | 32 | Amb a 1 | 0 | Cry j 1 | 0,61 | Cup a 1 | 2,45 |
| Italy - CAAM 4776 | M | 32 | Amb a 1 | 0 | Cry j 1 | 2,01 | Cup a 1 | 5,24 |
| Italy - CAAM 4777 | M | 32 | Amb a 1 | 0 | Cry j 1 | 1,56 | Cup a 1 | 4,45 |
| Italy - CAAM 4778 | F | 32 | Amb a 1 | 0 | Cry j 1 | 0,08 | Cup a 1 | 0,29 |
| Italy - CAAM 4779 | F | 32 | Amb a 1 | 0 | Cry j 1 | 0,99 | Cup a 1 | 2,17 |
| Italy - CAAM 4780 | F | 32 | Amb a 1 | 0 | Cry j 1 | 0,07 | Cup a 1 | 0,61 |
| Italy - CAAM 4781 | M | 32 | Amb a 1 | 0 | Cry j 1 | 1,8 | Cup a 1 | 5,07 |
| Italy - CAAM 4782 | F | 32 | Amb a 1 | 0 | Cry j 1 | 4,15 | Cup a 1 | 1,65 |
| Italy - CAAM 4783 | M | 32 | Amb a 1 | 0 | Cry j 1 | 1,55 | Cup a 1 | 3,02 |
| Italy - CAAM 4784 | F | 32 | Amb a 1 | 0 | Cry j 1 | 1,52 | Cup a 1 | 3,38 |
| Italy - CAAM 4785 | F | 32 | Amb a 1 | 0 | Cry j 1 | 7,33 | Cup a 1 | 34,26 |
| Italy - CAAM 4786 | F | 32 | Amb a 1 | 0 | Cry j 1 | 2,18 | Cup a 1 | 6,52 |
| Italy - CAAM 4787 | F | 32 | Amb a 1 | 0 | Cry j 1 | 0,06 | Cup a 1 | 0,14 |
| Italy - CAAM 4788 | M | 32 | Amb a 1 | 0 | Cry j 1 | 0,16 | Cup a 1 | 0,32 |
| Italy - CAAM 4789 | M | 32 | Amb a 1 | 0 | Cry j 1 | 0 | Cup a 1 | 0,1 |
| Italy - CAAM 4790 | F | 32 | Amb a 1 | 0 | Cry j 1 | 1,41 | Cup a 1 | 2,02 |
| Italy - CAAM 4791 | F | 32 | Amb a 1 | 0 | Cry j 1 | 0 | Cup a 1 | 0,35 |
| Italy - CAAM 4792 | M | 32 | Amb a 1 | 0 | Cry j 1 | 8,87 | Cup a 1 | 22,4 |
| Italy - CAAM 4793 | F | 32 | Amb a 1 | 0 | Cry j 1 | 0,21 | Cup a 1 | 0,32 |
| Italy - CAAM 4794 | F | 32 | Amb a 1 | 0 | Cry j 1 | 0,51 | Cup a 1 | 2,54 |
| Italy - CAAM 4795 | M | 32 | Amb a 1 | 0 | Cry j 1 | 0,56 | Cup a 1 | 0,17 |
| Italy - CAAM 4796 | F | 32 | Amb a 1 | 0 | Cry j 1 | 0,68 | Cup a 1 | 1,11 |
| Italy - CAAM 4797 | F | 32 | Amb a 1 | 0 | Cry j 1 | 0,79 | Cup a 1 | 11,63 |
| Italy - CAAM 4798 | F | 32 | Amb a 1 | 0 | Cry j 1 | 0,69 | Cup a 1 | 5,27 |
| Italy - CAAM 4799 | F | 32 | Amb a 1 | 0 | Cry j 1 | 0 | Cup a 1 | 0,8 |
| Italy - CAAM 4800 | M | 32 | Amb a 1 | 0 | Cry j 1 | 4,31 | Cup a 1 | 0 |
| Italy - CAAM 4801 | M | 32 | Amb a 1 | 0 | Cry j 1 | 3,57 | Cup a 1 | 0 |
| Italy - CAAM 4802 | M | 32 | Amb a 1 | 0 | Cry j 1 | 0,31 | Cup a 1 | 0,28 |
| Italy - CAAM 4803 | F | 32 | Amb a 1 | 0 | Cry j 1 | 0 | Cup a 1 | 0,38 |
| Italy - CAAM 4804 | F | 32 | Amb a 1 | 0 | Cry j 1 | 0 | Cup a 1 | 0,33 |
| Italy - CAAM 4805 | F | 32 | Amb a 1 | 0 | Cry j 1 | 1,64 | Cup a 1 | 2,41 |
| Italy - CAAM 4806 | F | 32 | Amb a 1 | 0 | Cry j 1 | 0 | Cup a 1 | 1,26 |
| Italy - CAAM 4807 | M | 32 | Amb a 1 | 0 | Cry j 1 | 7,72 | Cup a 1 | 6,8 |
| Italy - CAAM 4808 | F | 32 | Amb a 1 | 0 | Cry j 1 | 0 | Cup a 1 | 0,16 |
| Italy - CAAM 4809 | F | 32 | Amb a 1 | 0 | Cry j 1 | 16 | Cup a 1 | 17,51 |
| Italy - CAAM 4810 | F | 32 | Amb a 1 | 0 | Cry j 1 | 0,57 | Cup a 1 | 0 |
| Italy - CAAM 4811 | F | 32 | Amb a 1 | 0 | Cry j 1 | 0,96 | Cup a 1 | 1,65 |
| Italy - CAAM 4812 | F | 32 | Amb a 1 | 0 | Cry j 1 | 0,02 | Cup a 1 | 0 |
| Italy - CAAM 4813 | M | 32 | Amb a 1 | 0 | Cry j 1 | 6,94 | Cup a 1 | 2,91 |
| Italy - CAAM 4814 | F | 32 | Amb a 1 | 0 | Cry j 1 | 0,56 | Cup a 1 | 5,14 |
| Italy - CAAM 4815 | F | 32 | Amb a 1 | 0 | Cry j 1 | 9,33 | Cup a 1 | 27,3 |
| Italy - CAAM 4816 | M | 32 | Amb a 1 | 0 | Cry j 1 | 1,53 | Cup a 1 | 1,86 |
| Italy - CAAM 4817 | M | 32 | Amb a 1 | 0 | Cry j 1 | 0 | Cup a 1 | 3,09 |
| Italy - CAAM 4818 | F | 32 | Amb a 1 | 0 | Cry j 1 | 18,26 | Cup a 1 | 37,55 |
| Italy - CAAM 4819 | F | 32 | Amb a 1 | 0 | Cry j 1 | 3,85 | Cup a 1 | 5,84 |
| Italy - CAAM 4820 | M | 32 | Amb a 1 | 0 | Cry j 1 | 2,82 | Cup a 1 | 3,28 |
| Italy - CAAM 4821 | F | 32 | Amb a 1 | 0 | Cry j 1 | 0 | Cup a 1 | 0,2 |
| Italy - CAAM 4822 | M | 32 | Amb a 1 | 0 | Cry j 1 | 0,44 | Cup a 1 | 1,1 |
| Italy - CAAM 4823 | F | 32 | Amb a 1 | 0 | Cry j 1 | 0 | Cup a 1 | 0,34 |
| Italy - CAAM 4824 | F | 32 | Amb a 1 | 0 | Cry j 1 | 2,34 | Cup a 1 | 3,06 |
| Italy - CAAM 4825 | M | 32 | Amb a 1 | 0 | Cry j 1 | 4,21 | Cup a 1 | 9,5 |
| Italy - CAAM 4826 | F | 32 | Amb a 1 | 0 | Cry j 1 | 0,04 | Cup a 1 | 0 |
| Italy - CAAM 4827 | F | 32 | Amb a 1 | 0 | Cry j 1 | 0,23 | Cup a 1 | 2,9 |
| Italy - CAAM 4828 | F | 32 | Amb a 1 | 0 | Cry j 1 | 0,38 | Cup a 1 | 2,04 |
| Italy - CAAM 4829 | F | 32 | Amb a 1 | 0 | Cry j 1 | 0,05 | Cup a 1 | 0 |
| Italy - CAAM 4830 | F | 32 | Amb a 1 | 0 | Cry j 1 | 0,2 | Cup a 1 | 2,91 |
| Italy - CAAM 4831 | F | 32 | Amb a 1 | 0 | Cry j 1 | 0,16 | Cup a 1 | 0,3 |
| Italy - CAAM 4832 | F | 32 | Amb a 1 | 0 | Cry j 1 | 0,75 | Cup a 1 | 4,46 |
| Italy - CAAM 4833 | F | 32 | Amb a 1 | 0 | Cry j 1 | 0,58 | Cup a 1 | 0 |
| Italy - CAAM 4834 | F | 32 | Amb a 1 | 0 | Cry j 1 | 0,66 | Cup a 1 | 1,33 |
| Italy - CAAM 4835 | M | 32 | Amb a 1 | 0 | Cry j 1 | 0 | Cup a 1 | 0,14 |
| Italy - CAAM 4836 | F | 32 | Amb a 1 | 0 | Cry j 1 | 1,4 | Cup a 1 | 11 |
| Italy - CAAM 4837 | F | 32 | Amb a 1 | 0 | Cry j 1 | 0,52 | Cup a 1 | 3,51 |
| Italy - CAAM 4838 | F | 32 | Amb a 1 | 0 | Cry j 1 | 0 | Cup a 1 | 0,9 |
| Italy - CAAM 4839 | M | 32 | Amb a 1 | 0 | Cry j 1 | 1,49 | Cup a 1 | 3,62 |
| Italy - CAAM 4840 | F | 32 | Amb a 1 | 0 | Cry j 1 | 0 | Cup a 1 | 6,11 |
| Italy - CAAM 4841 | M | 32 | Amb a 1 | 0 | Cry j 1 | 0,04 | Cup a 1 | 0,33 |
| Italy - CAAM 4842 | F | 32 | Amb a 1 | 0 | Cry j 1 | 0,44 | Cup a 1 | 8,66 |
| Italy - CAAM 4843 | F | 32 | Amb a 1 | 0 | Cry j 1 | 7,57 | Cup a 1 | 0,94 |
| Italy - CAAM 4844 | M | 32 | Amb a 1 | 0 | Cry j 1 | 3,2 | Cup a 1 | 5,89 |
| Italy - CAAM 4845 | M | 32 | Amb a 1 | 0 | Cry j 1 | 4,71 | Cup a 1 | 25,47 |
| Italy - CAAM 4846 | F | 32 | Amb a 1 | 0 | Cry j 1 | 0,12 | Cup a 1 | 0,43 |
| Italy - CAAM 4847 | F | 32 | Amb a 1 | 0 | Cry j 1 | 0,19 | Cup a 1 | 2,33 |
| Italy - CAAM 4848 | M | 32 | Amb a 1 | 0 | Cry j 1 | 1,22 | Cup a 1 | 11,45 |
| Italy - CAAM 4849 | M | 32 | Amb a 1 | 0 | Cry j 1 | 0 | Cup a 1 | 1,91 |
| Italy - CAAM 4850 | F | 32 | Amb a 1 | 0 | Cry j 1 | 4,39 | Cup a 1 | 13,19 |
| Italy - CAAM 4851 | F | 32 | Amb a 1 | 0 | Cry j 1 | 0,26 | Cup a 1 | 0,62 |
| Italy - CAAM 4852 | M | 32 | Amb a 1 | 0 | Cry j 1 | 0 | Cup a 1 | 1,34 |
| Italy - CAAM 4853 | F | 32 | Amb a 1 | 0 | Cry j 1 | 0 | Cup a 1 | 1,22 |
| Italy - CAAM 4854 | F | 32 | Amb a 1 | 0 | Cry j 1 | 0,2 | Cup a 1 | 2,05 |
| Italy - CAAM 4855 | M | 32 | Amb a 1 | 0 | Cry j 1 | 0,14 | Cup a 1 | 0,64 |
| Italy - CAAM 4856 | M | 32 | Amb a 1 | 0 | Cry j 1 | 31,47 | Cup a 1 | 36,42 |
| Italy - CAAM 4857 | F | 32 | Amb a 1 | 0 | Cry j 1 | 0,69 | Cup a 1 | 4,43 |
| Italy - CAAM 4858 | F | 32 | Amb a 1 | 0 | Cry j 1 | 1,95 | Cup a 1 | 5,32 |
| Italy - CAAM 4859 | F | 32 | Amb a 1 | 0 | Cry j 1 | 5,86 | Cup a 1 | 6,14 |
| Italy - CAAM 4860 | M | 32 | Amb a 1 | 0 | Cry j 1 | 0 | Cup a 1 | 0,24 |
| Italy - CAAM 4861 | M | 32 | Amb a 1 | 0 | Cry j 1 | 1,78 | Cup a 1 | 5,49 |
| Italy - CAAM 4862 | F | 32 | Amb a 1 | 0 | Cry j 1 | 1,09 | Cup a 1 | 4,38 |
| Italy - CAAM 4863 | F | 32 | Amb a 1 | 0 | Cry j 1 | 0,67 | Cup a 1 | 3,49 |
| Italy - CAAM 4864 | M | 32 | Amb a 1 | 0 | Cry j 1 | 2,76 | Cup a 1 | 7,76 |
| Italy - CAAM 4865 | M | 32 | Amb a 1 | 0 | Cry j 1 | 0 | Cup a 1 | 3,8 |
| Italy - CAAM 4866 | M | 32 | Amb a 1 | 0 | Cry j 1 | 1,71 | Cup a 1 | 1 |
| Italy - CAAM 4867 | M | 32 | Amb a 1 | 0 | Cry j 1 | 0,53 | Cup a 1 | 3,89 |
| Italy - CAAM 4868 | F | 32 | Amb a 1 | 0 | Cry j 1 | 0 | Cup a 1 | 7,91 |
| Italy - CAAM 4869 | M | 32 | Amb a 1 | 0 | Cry j 1 | 3,26 | Cup a 1 | 11,61 |
| Italy - CAAM 4870 | F | 32 | Amb a 1 | 0 | Cry j 1 | 0 | Cup a 1 | 0,35 |
| Italy - CAAM 4871 | M | 32 | Amb a 1 | 0 | Cry j 1 | 2,44 | Cup a 1 | 4,96 |
| Italy - CAAM 4872 | F | 32 | Amb a 1 | 0 | Cry j 1 | 0 | Cup a 1 | 3,6 |
| Italy - CAAM 4873 | M | 32 | Amb a 1 | 0 | Cry j 1 | 0,14 | Cup a 1 | 0,75 |
| Italy - CAAM 4874 | M | 32 | Amb a 1 | 0 | Cry j 1 | 3,24 | Cup a 1 | 5,44 |
| Italy - CAAM 4875 | M | 32 | Amb a 1 | 0 | Cry j 1 | 0,06 | Cup a 1 | 0,22 |
| Italy - CAAM 4876 | M | 32 | Amb a 1 | 0 | Cry j 1 | 0,14 | Cup a 1 | 0,2 |
| Italy - CAAM 4877 | F | 32 | Amb a 1 | 0 | Cry j 1 | 5,62 | Cup a 1 | 5,75 |
| Italy - CAAM 4878 | F | 32 | Amb a 1 | 0 | Cry j 1 | 0,02 | Cup a 1 | 1,87 |
| Italy - CAAM 4879 | M | 32 | Amb a 1 | 0 | Cry j 1 | 0,86 | Cup a 1 | 5,09 |
| Italy - CAAM 4880 | F | 32 | Amb a 1 | 0 | Cry j 1 | 0 | Cup a 1 | 0,12 |
| Italy - CAAM 4881 | M | 32 | Amb a 1 | 0 | Cry j 1 | 1,91 | Cup a 1 | 4,41 |
| Italy - CAAM 4882 | M | 32 | Amb a 1 | 0 | Cry j 1 | 0,17 | Cup a 1 | 1,64 |
| Italy - CAAM 4883 | M | 32 | Amb a 1 | 0 | Cry j 1 | 0,06 | Cup a 1 | 0,73 |
| Italy - CAAM 4884 | F | 32 | Amb a 1 | 0 | Cry j 1 | 0,76 | Cup a 1 | 1,71 |
| Italy - CAAM 4885 | F | 32 | Amb a 1 | 0 | Cry j 1 | 15,45 | Cup a 1 | 79,7 |
| Italy - CAAM 4886 | F | 32 | Amb a 1 | 0 | Cry j 1 | 0 | Cup a 1 | 1,63 |
| Italy - CAAM 4887 | M | 32 | Amb a 1 | 0 | Cry j 1 | 1,14 | Cup a 1 | 11,03 |
| Italy - CAAM 4888 | M | 32 | Amb a 1 | 0 | Cry j 1 | 5,2 | Cup a 1 | 19 |
| Italy - CAAM 4889 | F | 32 | Amb a 1 | 0 | Cry j 1 | 1,37 | Cup a 1 | 9,5 |
| Italy - CAAM 4890 | M | 32 | Amb a 1 | 0 | Cry j 1 | 12,14 | Cup a 1 | 36,45 |
| Italy - CAAM 4891 | M | 32 | Amb a 1 | 0 | Cry j 1 | 0 | Cup a 1 | 5,59 |
| Italy - CAAM 4892 | M | 32 | Amb a 1 | 0 | Cry j 1 | 0,75 | Cup a 1 | 11,72 |
| Italy - CAAM 4893 | F | 32 | Amb a 1 | 0 | Cry j 1 | 0 | Cup a 1 | 0,29 |
| Italy - CAAM 4894 | F | 32 | Amb a 1 | 0 | Cry j 1 | 2,93 | Cup a 1 | 20,15 |
| Italy - CAAM 4895 | M | 32 | Amb a 1 | 0 | Cry j 1 | 0,26 | Cup a 1 | 1,17 |
| Italy - CAAM 4896 | M | 32 | Amb a 1 | 0 | Cry j 1 | 0,61 | Cup a 1 | 8,82 |
| Italy - CAAM 4897 | M | 32 | Amb a 1 | 0 | Cry j 1 | 0 | Cup a 1 | 2,6 |
| Italy - CAAM 4898 | M | 32 | Amb a 1 | 0 | Cry j 1 | 0 | Cup a 1 | 2,57 |
| Italy - CAAM 4899 | M | 32 | Amb a 1 | 0 | Cry j 1 | 0,67 | Cup a 1 | 5,63 |
| Italy - CAAM 4900 | F | 32 | Amb a 1 | 0 | Cry j 1 | 1,64 | Cup a 1 | 11,33 |
| Italy - CAAM 4901 | F | 32 | Amb a 1 | 0 | Cry j 1 | 0 | Cup a 1 | 3,58 |
| Italy - CAAM 4902 | M | 32 | Amb a 1 | 0 | Cry j 1 | 1,89 | Cup a 1 | 9,59 |
| Italy - CAAM 4903 | F | 32 | Amb a 1 | 0 | Cry j 1 | 0,74 | Cup a 1 | 3,61 |
| Italy - CAAM 4904 | M | 32 | Amb a 1 | 0 | Cry j 1 | 3,84 | Cup a 1 | 11,21 |
| Italy - CAAM 4905 | F | 32 | Amb a 1 | 0 | Cry j 1 | 0,18 | Cup a 1 | 2,85 |
| Italy - CAAM 4906 | F | 32 | Amb a 1 | 0 | Cry j 1 | 0,58 | Cup a 1 | 3,37 |
| Italy - CAAM 4907 | F | 32 | Amb a 1 | 0 | Cry j 1 | 3,03 | Cup a 1 | 10,02 |
| Italy - CAAM 4908 | M | 32 | Amb a 1 | 0 | Cry j 1 | 2,91 | Cup a 1 | 8,15 |
| Italy - CAAM 4909 | M | 32 | Amb a 1 | 0 | Cry j 1 | 0 | Cup a 1 | 0,4 |
| Italy - CAAM 4910 | F | 32 | Amb a 1 | 0 | Cry j 1 | 0 | Cup a 1 | 0,11 |
| Italy - CAAM 4911 | M | 32 | Amb a 1 | 0 | Cry j 1 | 2,66 | Cup a 1 | 9,29 |
| Italy - CAAM 4912 | F | 32 | Amb a 1 | 0 | Cry j 1 | 0,59 | Cup a 1 | 7,92 |
| Italy - CAAM 4913 | M | 32 | Amb a 1 | 0 | Cry j 1 | 0,12 | Cup a 1 | 1,36 |
| Italy - CAAM 4914 | F | 32 | Amb a 1 | 0 | Cry j 1 | 0 | Cup a 1 | 0,77 |
| Italy - CAAM 4915 | M | 32 | Amb a 1 | 0 | Cry j 1 | 0,29 | Cup a 1 | 3,35 |
| Italy - CAAM 4916 | M | 32 | Amb a 1 | 0 | Cry j 1 | 0,21 | Cup a 1 | 1,34 |
| Italy - CAAM 4917 | F | 32 | Amb a 1 | 0 | Cry j 1 | 0 | Cup a 1 | 2,12 |
| Italy - CAAM 4918 | M | 32 | Amb a 1 | 0 | Cry j 1 | 0,34 | Cup a 1 | 2,11 |
| Italy - CAAM 4919 | F | 32 | Amb a 1 | 0 | Cry j 1 | 0 | Cup a 1 | 0,12 |
| Italy - CAAM 4920 | F | 32 | Amb a 1 | 0 | Cry j 1 | 0,12 | Cup a 1 | 7,87 |
| Italy - CAAM 4921 | F | 32 | Amb a 1 | 0 | Cry j 1 | 1,02 | Cup a 1 | 5,34 |
| Italy - CAAM 4922 | M | 32 | Amb a 1 | 0 | Cry j 1 | 5,94 | Cup a 1 | 25,71 |
| Italy - CAAM 4923 | F | 32 | Amb a 1 | 0 | Cry j 1 | 0,35 | Cup a 1 | 12,55 |
| Italy - CAAM 4924 | M | 32 | Amb a 1 | 0 | Cry j 1 | 0 | Cup a 1 | 1,35 |
| Italy - CAAM 4925 | F | 32 | Amb a 1 | 0 | Cry j 1 | 5,2 | Cup a 1 | 12,07 |
| Italy - CAAM 4926 | F | 32 | Amb a 1 | 0 | Cry j 1 | 0,72 | Cup a 1 | 23,3 |
| Italy - CAAM 4927 | M | 32 | Amb a 1 | 0 | Cry j 1 | 0,29 | Cup a 1 | 4,34 |
| Italy - CAAM 4928 | F | 32 | Amb a 1 | 0 | Cry j 1 | 1,71 | Cup a 1 | 12,54 |
| Italy - CAAM 4929 | M | 32 | Amb a 1 | 0 | Cry j 1 | 2,17 | Cup a 1 | 19,89 |
| Italy - CAAM 4930 | F | 32 | Amb a 1 | 0 | Cry j 1 | 0,79 | Cup a 1 | 5,96 |
| Italy - CAAM 4931 | F | 32 | Amb a 1 | 0 | Cry j 1 | 0,69 | Cup a 1 | 2,78 |
| Italy - CAAM 4932 | M | 32 | Amb a 1 | 0 | Cry j 1 | 2,02 | Cup a 1 | 9,33 |
| Italy - CAAM 4933 | F | 32 | Amb a 1 | 0 | Cry j 1 | 1,36 | Cup a 1 | 10,91 |
| Italy - CAAM 4934 | M | 32 | Amb a 1 | 0 | Cry j 1 | 0 | Cup a 1 | 1,22 |
| Italy - CAAM 4935 | M | 32 | Amb a 1 | 0 | Cry j 1 | 0 | Cup a 1 | 0,6 |
| Italy - CAAM 4936 | F | 32 | Amb a 1 | 0 | Cry j 1 | 0 | Cup a 1 | 0,29 |
| Italy - CAAM 4937 | M | 32 | Amb a 1 | 0 | Cry j 1 | 0 | Cup a 1 | 5,4 |
| Italy - CAAM 4938 | M | 32 | Amb a 1 | 0 | Cry j 1 | 0,1 | Cup a 1 | 3,03 |
| Italy - CAAM 4939 | F | 32 | Amb a 1 | 0 | Cry j 1 | 0 | Cup a 1 | 6,5 |
| Italy - CAAM 4940 | F | 32 | Amb a 1 | 0 | Cry j 1 | 0,15 | Cup a 1 | 2,1 |
| Italy - CAAM 4941 | F | 32 | Amb a 1 | 0 | Cry j 1 | 1,38 | Cup a 1 | 5,48 |
| Italy - CAAM 4942 | F | 32 | Amb a 1 | 0 | Cry j 1 | 0,97 | Cup a 1 | 0,25 |
| Italy - CAAM 4943 | F | 32 | Amb a 1 | 0 | Cry j 1 | 0 | Cup a 1 | 18 |
| Italy - CAAM 4944 | F | 32 | Amb a 1 | 0 | Cry j 1 | 0 | Cup a 1 | 0,24 |
| Italy - CAAM 4945 | F | 32 | Amb a 1 | 0 | Cry j 1 | 0 | Cup a 1 | 1,32 |
| Italy - CAAM 4946 | M | 32 | Amb a 1 | 0 | Cry j 1 | 0,46 | Cup a 1 | 4,73 |
| Italy - CAAM 4947 | M | 32 | Amb a 1 | 0 | Cry j 1 | 0 | Cup a 1 | 0,94 |
| Italy - CAAM 4948 | F | 32 | Amb a 1 | 0 | Cry j 1 | 0 | Cup a 1 | 0,51 |
| Italy - CAAM 4949 | M | 31 | Amb a 1 | 0 | Cry j 1 | 3,36 | Cup a 1 | 3,46 |
| Italy - CAAM 4950 | F | 31 | Amb a 1 | 0 | Cry j 1 | 3,79 | Cup a 1 | 5,97 |
| Italy - CAAM 4951 | F | 31 | Amb a 1 | 0 | Cry j 1 | 1,24 | Cup a 1 | 4,72 |
| Italy - CAAM 4952 | M | 31 | Amb a 1 | 0 | Cry j 1 | 0 | Cup a 1 | 1,91 |
| Italy - CAAM 4953 | M | 31 | Amb a 1 | 0 | Cry j 1 | 0,33 | Cup a 1 | 0 |
| Italy - CAAM 4954 | F | 31 | Amb a 1 | 0 | Cry j 1 | 3,16 | Cup a 1 | 19,82 |
| Italy - CAAM 4955 | M | 31 | Amb a 1 | 0 | Cry j 1 | 2,22 | Cup a 1 | 4,29 |
| Italy - CAAM 4956 | F | 31 | Amb a 1 | 0 | Cry j 1 | 0 | Cup a 1 | 1,15 |
| Italy - CAAM 4957 | F | 31 | Amb a 1 | 0 | Cry j 1 | 1,5 | Cup a 1 | 4,07 |
| Italy - CAAM 4958 | F | 31 | Amb a 1 | 0 | Cry j 1 | 0,12 | Cup a 1 | 0,34 |
| Italy - CAAM 4959 | M | 31 | Amb a 1 | 0 | Cry j 1 | 0,88 | Cup a 1 | 1,07 |
| Italy - CAAM 4960 | F | 31 | Amb a 1 | 0 | Cry j 1 | 0 | Cup a 1 | 0,8 |
| Italy - CAAM 4961 | M | 31 | Amb a 1 | 0 | Cry j 1 | 0 | Cup a 1 | 3,17 |
| Italy - CAAM 4962 | F | 31 | Amb a 1 | 0 | Cry j 1 | 0 | Cup a 1 | 1,12 |
| Italy - CAAM 4963 | M | 31 | Amb a 1 | 0 | Cry j 1 | 0,17 | Cup a 1 | 0,22 |
| Italy - CAAM 4964 | M | 31 | Amb a 1 | 0 | Cry j 1 | 0,85 | Cup a 1 | 8,78 |
| Italy - CAAM 4965 | F | 31 | Amb a 1 | 0 | Cry j 1 | 0,41 | Cup a 1 | 1,65 |
| Italy - CAAM 4966 | F | 31 | Amb a 1 | 0 | Cry j 1 | 0 | Cup a 1 | 0,12 |
| Italy - CAAM 4967 | M | 31 | Amb a 1 | 0 | Cry j 1 | 1,65 | Cup a 1 | 8,14 |
| Italy - CAAM 4968 | M | 31 | Amb a 1 | 0 | Cry j 1 | 0,12 | Cup a 1 | 0,09 |
| Italy - CAAM 4969 | M | 31 | Amb a 1 | 0 | Cry j 1 | 0,13 | Cup a 1 | 0 |
| Italy - CAAM 4970 | F | 31 | Amb a 1 | 0 | Cry j 1 | 0,33 | Cup a 1 | 2,09 |
| Italy - CAAM 4971 | F | 31 | Amb a 1 | 0 | Cry j 1 | 0,07 | Cup a 1 | 0,86 |
| Italy - CAAM 4972 | M | 31 | Amb a 1 | 0 | Cry j 1 | 0 | Cup a 1 | 1,03 |
| Italy - CAAM 4973 | M | 31 | Amb a 1 | 0 | Cry j 1 | 4,94 | Cup a 1 | 16,39 |
| Italy - CAAM 4974 | F | 31 | Amb a 1 | 0 | Cry j 1 | 0,84 | Cup a 1 | 1,48 |
| Italy - CAAM 4975 | F | 31 | Amb a 1 | 0 | Cry j 1 | 1,82 | Cup a 1 | 5,99 |
| Italy - CAAM 4976 | F | 31 | Amb a 1 | 0 | Cry j 1 | 0,67 | Cup a 1 | 0,77 |
| Italy - CAAM 4977 | F | 31 | Amb a 1 | 0 | Cry j 1 | 9,36 | Cup a 1 | 9,45 |
| Italy - CAAM 4978 | F | 31 | Amb a 1 | 0 | Cry j 1 | 2,16 | Cup a 1 | 6,37 |
| Italy - CAAM 4979 | F | 31 | Amb a 1 | 0 | Cry j 1 | 0,59 | Cup a 1 | 1,95 |
| Italy - CAAM 4980 | M | 31 | Amb a 1 | 0 | Cry j 1 | 7,4 | Cup a 1 | 20,24 |
| Italy - CAAM 4981 | F | 31 | Amb a 1 | 0 | Cry j 1 | 0,21 | Cup a 1 | 0 |
| Italy - CAAM 4982 | F | 31 | Amb a 1 | 0 | Cry j 1 | 0,05 | Cup a 1 | 0,2 |
| Italy - CAAM 4983 | F | 31 | Amb a 1 | 0 | Cry j 1 | 29,83 | Cup a 1 | 27,6 |
| Italy - CAAM 4984 | F | 31 | Amb a 1 | 0 | Cry j 1 | 0 | Cup a 1 | 1,1 |
| Italy - CAAM 4985 | F | 31 | Amb a 1 | 0 | Cry j 1 | 0,36 | Cup a 1 | 8,4 |
| Italy - CAAM 4986 | M | 31 | Amb a 1 | 0 | Cry j 1 | 2,67 | Cup a 1 | 3,08 |
| Italy - CAAM 4987 | F | 31 | Amb a 1 | 0 | Cry j 1 | 1,97 | Cup a 1 | 3,05 |
| Italy - CAAM 4988 | M | 31 | Amb a 1 | 0 | Cry j 1 | 9,44 | Cup a 1 | 23,72 |
| Italy - CAAM 4989 | F | 31 | Amb a 1 | 0 | Cry j 1 | 0,03 | Cup a 1 | 0,03 |
| Italy - CAAM 4990 | M | 31 | Amb a 1 | 0 | Cry j 1 | 0 | Cup a 1 | 0,31 |
| Italy - CAAM 4991 | F | 31 | Amb a 1 | 0 | Cry j 1 | 8,34 | Cup a 1 | 13,27 |
| Italy - CAAM 4992 | F | 31 | Amb a 1 | 0 | Cry j 1 | 0,94 | Cup a 1 | 1,58 |
| Italy - CAAM 4993 | F | 31 | Amb a 1 | 0 | Cry j 1 | 0,22 | Cup a 1 | 2,75 |
| Italy - CAAM 4994 | F | 31 | Amb a 1 | 0 | Cry j 1 | 5,7 | Cup a 1 | 4,62 |
| Italy - CAAM 4995 | M | 31 | Amb a 1 | 0 | Cry j 1 | 1 | Cup a 1 | 2,08 |
| Italy - CAAM 4996 | F | 31 | Amb a 1 | 0 | Cry j 1 | 3,51 | Cup a 1 | 1,42 |
| Italy - CAAM 4997 | M | 31 | Amb a 1 | 0 | Cry j 1 | 1,18 | Cup a 1 | 21,34 |
| Italy - CAAM 4998 | F | 31 | Amb a 1 | 0 | Cry j 1 | 7,89 | Cup a 1 | 0 |
| Italy - CAAM 4999 | M | 31 | Amb a 1 | 0 | Cry j 1 | 30,66 | Cup a 1 | 6,45 |
| Italy - CAAM 5000 | F | 31 | Amb a 1 | 0 | Cry j 1 | 0,11 | Cup a 1 | 1,75 |
| Italy - CAAM 5001 | M | 31 | Amb a 1 | 0 | Cry j 1 | 0,96 | Cup a 1 | 1,68 |
| Italy - CAAM 5002 | F | 31 | Amb a 1 | 0 | Cry j 1 | 8,2 | Cup a 1 | 6,31 |
| Italy - CAAM 5003 | M | 31 | Amb a 1 | 0 | Cry j 1 | 1,25 | Cup a 1 | 3,84 |
| Italy - CAAM 5004 | F | 31 | Amb a 1 | 0 | Cry j 1 | 0,6 | Cup a 1 | 0,73 |
| Italy - CAAM 5005 | F | 31 | Amb a 1 | 0 | Cry j 1 | 0 | Cup a 1 | 0,89 |
| Italy - CAAM 5006 | F | 31 | Amb a 1 | 0 | Cry j 1 | 0,33 | Cup a 1 | 0,78 |
| Italy - CAAM 5007 | F | 31 | Amb a 1 | 0 | Cry j 1 | 1,03 | Cup a 1 | 8,24 |
| Italy - CAAM 5008 | F | 31 | Amb a 1 | 0 | Cry j 1 | 1,57 | Cup a 1 | 2,17 |
| Italy - CAAM 5009 | F | 31 | Amb a 1 | 0 | Cry j 1 | 5,96 | Cup a 1 | 10,01 |
[truncated: 416,595 more chars]
